# South African Abietane Diterpenoids and Their Analogs as Potential Antimalarials: Novel Insights from Hybrid Computational Approaches

**DOI:** 10.3390/molecules24224036

**Published:** 2019-11-07

**Authors:** Thommas Musyoka, Özlem Tastan Bishop

**Affiliations:** Research Unit in Bioinformatics (RUBi), Department of Biochemistry and Microbiology, Rhodes University, Grahamstown 6140, South Africa; mutemibiochemistry@gmail.com

**Keywords:** falcipains, docking, molecular dynamics simulation, dynamic residue interaction network, binding free energy

## Abstract

The hemoglobin degradation process in *Plasmodium* parasites is vital for nutrient acquisition required for their growth and proliferation. In *P. falciparum*, falcipains (FP-2 and FP-3) are the major hemoglobinases, and remain attractive antimalarial drug targets. Other *Plasmodium* species also possess highly homologous proteins to FP-2 and FP-3. Although several inhibitors have been designed against these proteins, none has been commercialized due to associated toxicity on human cathepsins (Cat-K, Cat-L and Cat-S). Despite the two enzyme groups sharing a common structural fold and catalytic mechanism, distinct active site variations have been identified, and can be exploited for drug development. Here, we utilize in silico approaches to screen 628 compounds from the South African natural sources to identify potential hits that can selectively inhibit the plasmodial proteases. Using docking studies, seven abietane diterpenoids, binding strongly to the plasmodial proteases, and three additional analogs from PubChem were identified. Important residues involved in ligand stabilization were identified for all potential hits through binding pose analysis and their energetic contribution determined by binding free energy calculations. The identified compounds present important scaffolds that could be further developed as plasmodial protease inhibitors. Previous laboratory assays showed the effect of the seven diterpenoids as antimalarials. Here, for the first time, we demonstrate that their possible mechanism of action could be by interacting with falcipains and their plasmodial homologs. Dynamic residue network (DRN) analysis on the plasmodial proteases identified functionally important residues, including a region with high *betweenness centrality*, which had previously been proposed as a potential allosteric site in FP-2.

## 1. Introduction

The implementation of artemisinin-based combination therapy (ACT) as the first line drug in the treatment of uncomplicated malaria cases has led to a substantial reduction in the global malaria burden in recent years [1]. However, the disease still remains a major public health concern in the tropical areas of Africa, Southern East Asia and Eastern Mediterranean [1]. According to the 2018 malaria report by the World Health Organization (WHO), 219 million malaria cases were reported in 2017, resulting in 435,000 deaths [1]. With recent disease surveillance reports indicating the development and spread of plasmodia strains with ACT drug resistance in South East Asia, the current combined efforts towards malaria control and elimination could be derailed [2,3,4,5]. While this is an anticipated phenomenon considering the parasite’s inherent ability of overcoming the therapeutic effect of almost all known antimalarial drugs [6,7], a major health concern remains how global disease control systems are prepared should ACTs become completely ineffective. Consequently, the search for next generation antimalarial drugs that can interrupt essential molecular pathways, crucial for parasite growth and multiplication, remains a top priority.

The hemoglobin degradation pathway, a multistage process occurring in an acidic compartment, the food vacuole, is essential for the survival of the parasite [8,9]. During the intraerythrocytic stage, plasmodia employ an array of proteases to degrade almost 75% of hemoglobin inside the host’s red blood cells to obtain essential amino acids with an exception of isoleucine [10,11,12]. Due to the significance of this pathway, the plasmodial hemoglobin degrading proteases present attractive and promising drug targets.

**Figure 1:**
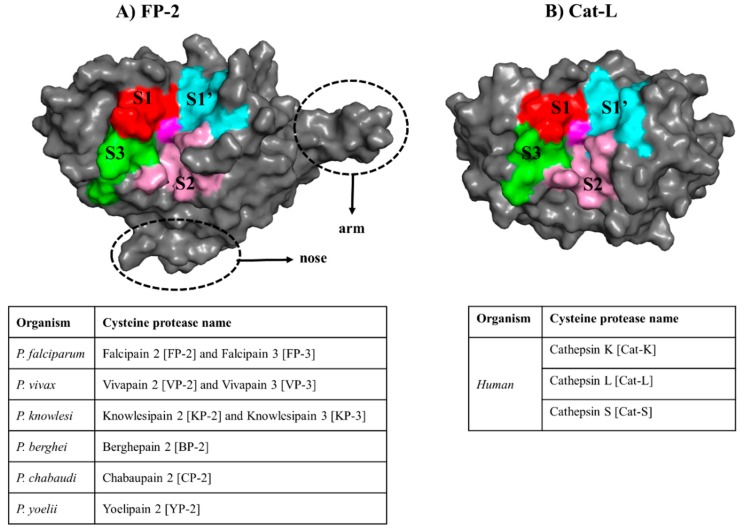
The general structural fold of (**A**) falcipains (FPs) and plasmodial homologs and (**B**) human cysteine proteases. The different subsites forming the “trench-like” active pocket are shown in red (S1), pink (S2), green (S3) and cyan (S1′). The central catalytic Cys residue is colored in magenta. The unique structural features (nose and arm) found only in plasmodial proteases are marked with a broken line. Tables present the name of the homologous FP-2 and FP-3 proteins from other *Plasmodium* species as well as the human host.

In *Plasmodium falciparum*, two cysteine proteases viz. Falcipain 2 (FP-2) and Falcipain 3 (FP-3) have been identified as the major hemoglobinases [13,14,15], which initiate the process by cleaving hemoglobin at multiple sites [16]. Enzyme assay studies reveal that the abundance and activity of FP-2 is at peak during the early phases of trophozoite development, in contrast to that of FP-3, which is mainly expressed at the lateral stages of maturation and release [15,17]. Similarly, other *Plasmodium* species also possess highly homologous proteins to FP-2 and FP-3 [18,19,20,21]. A major limitation with targeting these plasmodial proteases is their close homology with human cathepsin proteins (Cathepsin K (Cat-K), Cathepsin L (Cat-L) and Cathepsin S (Cat-S)). The two protein groups have a similar structural fold except that plasmodial proteases possess longer prodomain regions and two distinct inserts within their mature (catalytic) domain: a “nose” and “arm” of ~17 and ~14 residues, respectively [13,22,23] (Figure 1).

Both the plasmodial and human proteins belong to the Clan CA of the C1 family of enzymes, which are characterized by the Cys-His-Asn catalytic triad centrally located in a cleft at the junction between left and right domains [24]. They all share a common catalytic mechanism in which the triad’s His residue deprotonates the thiol group of the catalytic Cys residue priming it for a nucleophilic attack under reducing and acidic pH environment [13,16,17,24]. Additional residues around these catalytic triad centers also play important roles during the substrate hydrolysis process, and are grouped into four subsites namely S1, S2, S3 and S1′ [25] (Figure 1). In spite of their similarities, our previous studies have revealed distinctive phylogenetic clustering between the two protein groups [26] as well as important subsite residue composition differences, which could be exploited for inhibitor design [27]. The significance of FP-2 as a key drug target has previously been demonstrated through in vitro inhibition studies using chemical compounds or genetic manipulation [15,28]. The interference of FP-2 activity triggered food vacuole swelling due to the accumulation of undigested hemoglobin resulting in an altered parasite growth pattern. Moreover, a recent study by Siddiqui et al. showed a possible association of the presence of non-synonymous mutations in the FP-2 gene with decreased artemisinin sensitivity [29].

In an ongoing effort to identify natural inhibitors against FP-2, FP-3 proteins and their homologs from other *Plasmodium* species, the current work uses computational approaches, including compound docking, molecular dynamics (MD) and binding free energy (BFE) calculations to identify possible hits from the South African Natural Compound Database (SANCDB) [30]. This is a continuation of our previous work using a small subset of 23 SANCDB compounds where we identified a compound, 5α-Pregna-1,20-dien-3-one (5PGA), as a potential hit against the plasmodial proteases while showing selectivity towards the human cathepsins [31]. Together, these studies are inspired by the significant role of secondary metabolites from nature in drug development as a source of important scaffolds as they have unmatched chemical diversity, physicochemical properties and structural complexity [32,33,34,35]. In addition, antimalarial chemotherapy has greatly been influenced by natural products, including artemisinin. From the literature, several studies have identified a set of non-peptidic chemical compounds from natural sources with inhibitory potency of up to the micromolar level against FP-2 [36,37]. A major limitation of these studies is that they focus only on FP-2 and FP-3. However, for successful drug development against these plasmodial cysteine enzymes, the broad activity against the other homologs as well as the concurrent inhibition of both FP-2 and FP-3 is necessary due to redundancy in their function [10].

From the 628 SANCDB compounds screened, seven abietane diterpenoids, namely SANC00364, SANC00365, SANC00367, SANC00369, SANC00371, SANC00372 and SANC00373 bound strongly to the plasmodial hemoglobinases. From the literature, these compounds have shown to possess antimalarial properties [38]. Based on the binding energy, SANC00369, SANC00371, SANC00372 and SANC00373 exhibited selectivity towards human cathepsins. A >80% structure similarity search using the identified SANCDB hits yielded three additional hits from PubChem (126461286, 126462623, 126465495) with stronger binding affinities on plasmodial proteases possibly due to their extended chemical structures. The key interactions between the different ligands and the various proteins were identified. These were mainly hydrophobic contacts, which were mainly influenced by the size and chemical groups present in the ligands. Besides the known functional catalytic subsite residues, dynamic residue network (DRN) analysis revealed several functionally important residues that are distal from the active pocket in the plasmodial proteases. In FP-2, some of these residues have been linked to its allosteric modulation by heme although the mechanism still remains unclear [39]. Collectively, the current results provide novel insights on the possible antimalarial action of seven South African compounds through the inhibition of the hemoglobin degradation pathway. These compounds, together with their analogs from PubChem, present important scaffolds that can be optimized further for the development of potent inhibitors against FP-2, FP-3 and their plasmodial homologs.

## 2. Results and Discussion

### 2.1. Accurate Ligand Docking Parameters are Established and Validated

Several computational approaches for the identification of bioactive molecules from compound libraries already exist [40,41]. Docking, an approach that predicts the receptor binding mode of small molecules, has become an indispensable method in the computer-aided drug design (CADD) process. Through combination and optimization of hydrophobic, steric and electrostatic interactions, the method estimates the interaction energy between a protein and a ligand. During screening, accurate parameters are required for the sampling and scoring process to prioritize potential binders from non-binders [42]. Re-docking of co-crystallized ligands is a common approach for the selection and calibration of docking parameters. Depending on the size of the ligand, successful validation is considered if the re-docked pose has a root mean square deviation (RMSD) value ≤ 2.0 Å [43]. As a benchmarking process, a set of decoys (compounds which are presumed to be inactive against the target under investigation) are seeded with a fraction of compounds with known activity and then subjected to the docking process. Ranking is then performed and the enrichment of the docking process determined through Receiver Operating Characteristic (ROC) curves and Area Under Curve (AUC) plots [44]. ROC utilizes a binary classifier to discriminate binders from non-binders, thus determining the sensitivity and specificity of the docking process. AUC is a measure of the reliability of the classifier with values <0.5, indicating a random process. Several hit screening studies have successfully applied this method in the past [45,46,47,48,49].

**Figure 2:**
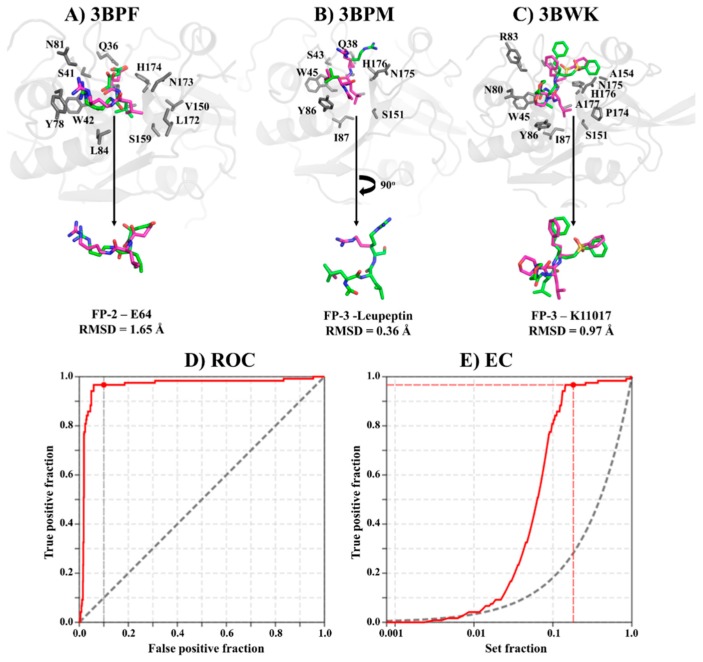
Docking protocol validation. (**A**–**C**) Root mean square deviation (RMSD) comparison between crystallographic ligand pose (pink) and the lowest energy redocked conformation (green) for E64, Leupeptin and K11017 in FP-2 and FP-3. Benchmarking result of our docking process as shown by (**D**) Receiver Operating Characteristic (ROC) and (**E**) an enrichment curve using a library of active compounds against FP-2 and their decoys.

Conformational deviation of re-docked co-crystallized ligands in FP-2 (PDB: 3BPF, E64) and FP-3 (PDB: 3BPM, Leupeptin and 3BWK, K11017) showed that all the ligands had RMSD values < 2.0 Å, an indication that our parameters were suitable for the structure-based screening process (Figure 2A–C). This was despite the three compounds having numerous rotatable bonds. A benchmarking process of our protocol using 30 active compounds against FP-2 (from the literature with IC_50_ ≤ 10 μM) and their decoys (1500) achieved early true positive recognition with 96% of the active compounds being identified within 10% of the testing library search. Moreover, a Boltzmann-Enhanced Discrimination of the Receiver Operating Characteristic (BEDROC) and AUC of 0.75 and 0.96 were achieved. A similar result was obtained by Mugumbate et al. using a collection of 13 active compounds against FP-2 and 506 decoys from the National Cancer Institute open database [46].

### 2.2. Potential Plasmodial Cysteine Protease Inhibitors Are Identified from SANCDB

**Figure 3:**
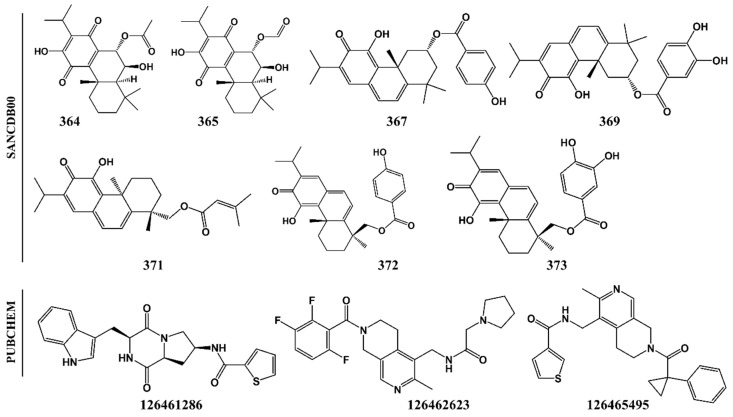
2D representation of identified South African Natural Compound Database (SANCDB) hits and analog compounds from PubChem.

Using these validated parameters, the binding mode of 628 SANCDB compounds on the “trench-like” active site of FPs and its homologs from other *Plasmodium* species as well as the human cathepsins was evaluated. Based on the binding energy scores, seven abietane diterpene compounds (SANC00364, SANC00365, SANC00367, SANC00369, SANC00371, SANC00372 and SANC00373) showing strong interactions with majority of the plasmodial proteases were identified (Figure 3). A previous in vitro study [38] showed that the identified SANCDB compounds exhibited strong anti-plasmodial activities against chloroquine-resistant (CQ^R^) *P. falciparum* strain(s), and inhibited *β*-hematic formation in a synergistic or additive manner when used together with chloroquine or quinine. This demonstrated that these compounds interfered with the hemoglobin degradation pathway.

The total interaction energy in AutoDock is determined by a semiempirical free energy scoring function consisting of two components (intramolecular and intermolecular energy) and is proportional to the number of interactions between a protein receptor and the ligand. Thus, ligands with extended structures tend to establish more contacts with receptor residues, resulting in stronger interaction energies. SANC00364 and SANC00365 are the smallest ligands in the compound set, a possible explanation of their weaker interaction energies compared to others. Despite SANC00364 and SANC00365 having weak binding affinity on the different plasmodial proteins compared to the other SANCDB hits, we also selected them for further in silico studies to elucidate if they could potentially inhibit the hemoglobinases. The seven selected compounds were all isolated from plant species belonging to *Plectranthus* [30,38]. To ensure that the correct ligand binding mode was chosen, a post-docking analysis procedure was performed, and clusters with a pose occupancy of >70% were selected.

**Figure 4:**
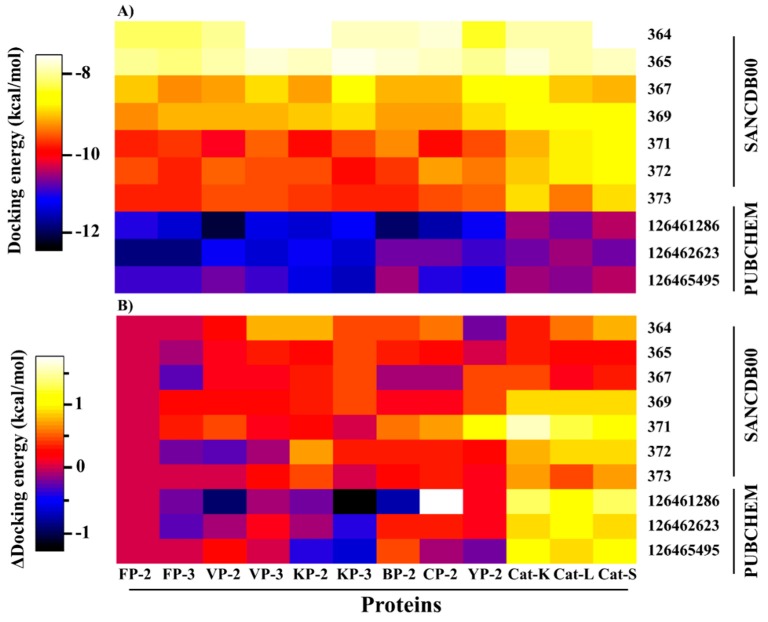
A heatmap representation of (**A**) interaction energies between the selected hits and the plasmodial proteases as well as human cathepsins and (**B**) interaction energy difference of the various complexes to the corresponding FP-2-hit complex. Weak interactions are shown in white while in black are complexes with the strongest affinities.

Even with the high structural similarity of both the plasmodial proteases and cathepsins active site pockets, four of the identified hits (SANC00369, SANC00371, SANC00372 and SANC373) exhibited a differential affinity binding profiles against the human cathepsins, a probable indication of selectivity (Figure 4).

**Figure 5:**
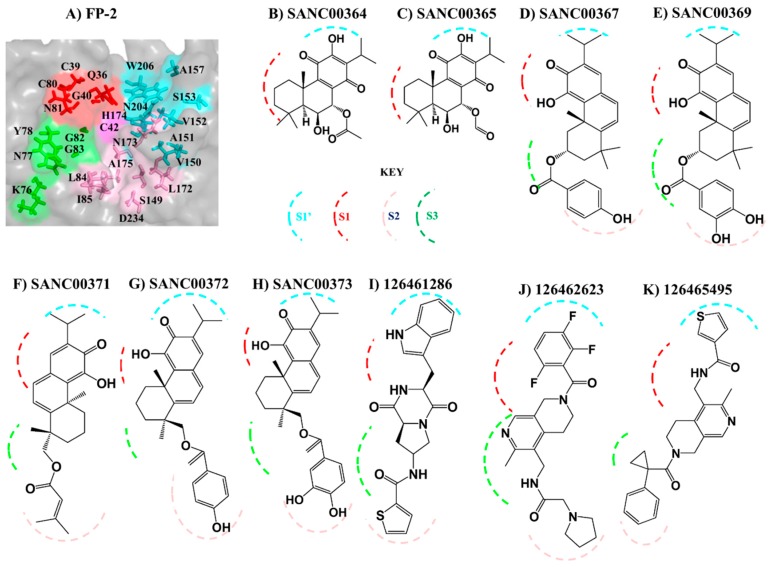
Ligand binding mode in plasmodial proteases. (**A**) A zoomed surface representation of the FP-2 active site. Shown in sticks are the different subsite residues (S1 = Q36, C39, G40, C80, N81; S2 = L84, I85, S149, L172, N173, A175, D234; S3 K76, N77, Y78, G82, G83; S1′ = V150, A151, A157, H174, N204, W206). (**B**–**K**) Schematic representation of the interactions of the different compounds with the four subsites in the plasmodial proteins. S1 = Red, S2 = Pink, S3 = Green and S1′ = Pink broken lines.

In our previous study [27], we identified the amino acid composition of the four different subsite pockets in both the plasmodial and human cathepsin proteins (Figure 1 and Figure 5A). Here, to determine the key residues (numbering according to the catalytic domain of each protein—Table S1) stabilizing the ligands inside of the subsite pockets, a protein–ligand interaction analysis was performed on each complex using LIGPLOT^+^ [50]. Structural analysis of the different subsites shows that both S2 and S1′ (formed by 7 and 8 residues, respectively) can accommodate large chemical groups as they are broader compared to the other subsites. However, the access to S2 is regulated by a narrow mouth formed by two “gate-keeper” residues. The interactions with S1 and S3 are mainly with the residues that form the lining of the “trench-like” active pocket. From subsite composition analysis, a high variation in S2 and S1′ [27] residues exists between human cathepsins and plasmodial proteases, a key aspect which can be utilized to achieve drug selectivity. Thus, ligands with a propensity of interacting with these subsites should be prioritized for inhibitor design. Previous studies have showed that the S2 that forms the bottom end of the “trench-like” active site is known to be a critical determinant of substrate specificity for this group of enzymes [25]. Unique to the human plasmodial proteases, with an exception of VP-3, is the presence of a negatively charged residue (mostly Glu) at the deep end of S2 as compared to the human cathepsins, which have a non-polar (hydrophobic) residue. From the residue interaction fingerprint analysis, the ligands had a differential subsite binding pattern with the various proteins and was dependent on their size and chemical composition (Table S2).

A close look at the identified hit structures (Figure 3 and Figure 5) shows that these ligands have a short central core consisting of a quinone moiety fused together with two cyclohexane rings. A detailed docking pose analysis showed that the quinone moiety formed interactions with the highly conserved Trp residue located at the S1′ subsite floor in all the proteins studied. Additional S1′ interactions were mediated by an isopropyl chemical group attached to the quinone moiety. The remaining nearby interactions were mainly between S1 residues and the double-fused cyclohexane rings, which mainly formed hydrophobic interactions with neighboring non-subsite residues around the S1 and S1′ subsites. Due to the absence of tail groups, both SANC00364 and SANC00365 lacked interactions with the other subsite residues. Besides the interactions with these two compounds, both SANC00367 and SANC00369 had additional interactions mainly with the highly conserved Gly residues lining the S3 wall. Furthermore, these two ligands made limited contact with residues forming the S2 entrance but not with those at the pocket’s deep end as they had a short central ester linker group (Table S1, Figure 3 and Figure 5). Due to these additional interactions, the compounds registered stronger binding interactions compared to SANC00364 and SANC00365. SANC00371-373 compounds effectively spanned the entire active pocket and interacted with most of the important residues forming the different subsites through hydrophobic effects and hydrogen bonds. This sufficiently explains why these set of compounds had stronger interaction energy compared to the rest of the other SANCDB hits. These compounds had an extended ester linker group compared to SANC00367 and SANC00369 (Figure 3). From the results, additional hit modifications on the SANCDB hits may result in highly potent and specific plasmodial cysteine protease inhibitors. Considering the interaction profile made with S1′ and S1 residues by SANC00364 and 365, these two compounds also offer important starting scaffolds, which may require additional modification experiments mainly involving the addition of extra tail groups aimed at interacting with S2 residues. This would boost their binding affinity against the plasmodial proteases and may lead to novel derivatives with potential of becoming inhibitors against the plasmodial proteases. Several modelling studies have identified the key pharmacophoric elements that a ligand should possess for effective FP-2 and FP-3 inhibitory activity [51,52,53]. Nevertheless, none of these has provided details of integral aspects necessary for achieving selectivity on human cathepsins. A significant feature for optimum plasmodial protease inhibition is the presence of chemical groups that favorably interact with the protein’s bulky electronegative and hydrophilic preferred regions of the S2 and S1′, respectively. Although some studies have suggested the presence of a hydrogen bond forming chemical groups with S3 residues increases ligand stabilization, our previous BFE calculations revealed that these interactions impaired binding and should be avoided [27]. For stronger interactions with S1′ and neighboring S1 residues, substitutions of the head group with strong electronegative groups like the halides (F, Cl and Br), especially at the *para* and *ortho* positions, are essential [53,54]. Additionally, the presence of hydroxyl groups in the groups interacting with the S2, which is mainly hydrophobic, should be avoided. To find additional hits that meet most of these requirements, a ligand-based virtual screening was performed using the identified hits on the PubChem [55] and MolPort chemical databases [56]. Where necessary, additional substitutions of the hydroxyl groups in the head and tail groups of SANC00371-373 were performed. In case of SANC00371, the tail methyl groups were also substituted with different bulky cyclic groups. Out of 153 analogs obtained, three exhibited strong interactions with most of the plasmodial proteases whilst showing selectivity (~1.0 kcal/mol interaction energy difference) on the human cathepsins (Figure 4B). The selected analogs namely 126461286, 126462623 and 126465495 were all from PubChem. Attached to the ends of their extended structures were bulky groups which favored the establishment of strong interactions with all the four subsites in the plasmodial proteases (Table S1, Figure S1). 126462623 had the majority of the necessary pharmacophoric features for a desirable inhibition of plasmodial proteases, including the presence of strong electronegative groups (F) necessary for S1′ interactions of notable strength. In human cathepsins, the compound had a different binding pose orientation with the head group with fluorine (F) groups facing away from S1′. This may be due to volume differences in the S1′ subsite of plasmodial and human proteases. The remaining two PubChem analogs, 126461286 and 126465495, which had narrower head groups, fitted in the S1′ site and interacted with the majority of its residues. Combining the results obtained with SANCDB and PubChem, important structural features necessary for designing novel inhibitors against the plasmodial proteases were identified.

### 2.3. Drug-Like Properties of the Identified Hits and Analogs are Studied

A major factor in reducing attrition rate in the drug development process is the early detection and filtering of hits that have unfavorable structural and physicochemical properties that may result in promiscuous activity on multiple protein targets and poor ADMET (absorption, distribution, metabolism, excretion, toxicity) profiles. To increase the probability of identifying compounds with desirable features, various metrics were checked. To determine if the identified hits had the acceptable properties, they were all evaluated for drug-likeness using the Lipinski’s rule of five (Ro5) [57]. In addition, the presence of substructure features associated with promiscuous binding on protein targets was evaluated using the pan assay interference compounds (PAINS) filter [58] using the FAF-Drugs2 ADMET filtering webserver [59]. As presented in Table 1, all the selected hits had acceptable drug-like scores with molecular weights below 500 Daltons. However, only SANC00369, SANC00371, SANC00372 and the PubChem hits passed the PAINS filter. The PAINS sub-structural motif in both SANC00364 and SANC00365 was the quinone moiety while in SANC00369 and SANC00373 it was the catechol group. Although the PAINS metric has become a common tool in pre-filtering large chemical libraries leading to the identification of compounds with unattractive pharmacokinetic properties, up to 5% of the current number of FDA (U.S. Food and Drug Administration) drugs have been found to contain PAINS-recognized features [60,61]. Thus, additional biological assays may be necessary to confirm if indeed the four flagged SANCDB compounds have promiscuous interaction profiles with off targets. Where necessary, additional hit modifications followed up by chemical assays may be undertaken to improve their pharmacokinetic properties.

### 2.4. Protein-Compound Stability for Each System is Assessed Through Molecular Dynamic Studies

Molecular dynamics simulations remain one of the most common and versatile computational method in CADD through which the dynamic, stability and energetic properties of biological complexes can be deciphered [62,63,64]. Through MD, all the components in a system are treated in a flexible manner over time, thus overcoming the limitations of docking. To further understand the conformational changes and stability of complexes selected from docking studies, all-atom MD simulations of 100 ns were performed both on the ligand-free and ligand-bound protein systems using the GROMACS simulation package [65]. The global and local conformational changes in the different systems were then determined by the backbone root mean square deviation (RMSD) and root mean square fluctuations (RMSF), respectively, over the MD trajectory.

From the RMSD plots, all proteins systems (apo and complexes) achieved stable RMSD profiles after an equilibration time of 30 ns (Figure S2A–S13A). In the RStudio statistical package (Version 1.1.442, RStudio Team 2016), a comparison of the RMSD distribution of the apo vs. the corresponding ligand-bound complexes was performed for the last 70 ns of simulation. The z-test and Kolmogorov–Smirnov (KS) [66] statistical tests were applied to establish if there was any significant difference between the RMSD population means and the distribution of the apo and ligand-bound systems as performed by Penkler et al. [67]. Likewise, the conformational fluctuations of the selected hits during simulations were also evaluated by the same approach. In the apo form, the plasmodial proteases have Cα backbone fluctuations ranging between 0.19 and 0.29 nm while that of human cathepsins is between 0.17 and 0.23 nm (Figure S14 and Table S3). All the apo forms had RMSD values with a normal distribution around their means with an exception of FP-2, VP-2, KP-3 and Cat-L, which displayed a bimodal distribution. A close analysis of the trajectories of these four proteins revealed several structural elements, mostly loops (FP-2 = 100–125, VP-2 = 220–227, KP-3 = 220–227 and Cat-L = 96–107), having higher fluctuations than the rest of the proteins as displayed by the RMSF profiles (Figure S2B–S13B). However, addition of the various ligands led to the stabilization of these regions with resulting complexes displaying normal distribution profiles around their means (Figure S14). From the statistical tests, there was a significant difference in the RMSD means and its distribution between the apo and ligand-bound complexes (Table S3).

Analysis of the RMSD distribution of ligands when complexed with the various proteins after equilibration showed different distribution patterns, which may be linked to their binding modes within the active site pockets. SANC00364 and SANC00365 displayed minimal conformational changes with normal distribution means with a standard deviation of ≤ 0.01 nm (Figure S15). This was expected behavior as these ligands had only three rotatable bonds, none of which was in the central core of their structures making them extremely rigid. This can also be seen from their trajectory profiles (Figure S2A–S13A). Ligands showing bimodal distribution experienced changes in their binding modes around the rotatable bonds, causing fluctuations in their RMSD profiles as well as the residues flipping portions of their structures during simulation. Despite SANC00371-373 and PubChem hits having more than five rotatable bonds, they exhibited stable conformations, especially with the plasmodial proteases (Figure S15 and Figure S2A–S13A).

To assess how ligand addition influenced the residue fluctuations in each protein, RMSF analysis was performed (Figure S16 and Figure S2B–S13B). From the results, ligand binding significantly reduced the conformational fluctuation of residues located in loop regions around the active site in most of the proteins. However, no changes were observed with residues found on the arm region (loop f) of plasmodial proteases, which is a distance away from the active pocket. In human cathepsins, minimal fluctuations were observed in this region as they possess a shorter arm. The greatest stabilization in the various loops was witnessed in BP-2 with the different ligands. The catalytic triad (Cys-His-Asn) in both the plasmodial and human proteins experienced minimal fluctuations in both the ligand bound and free systems (Figure S16 and Figure S2B–S13B). These residues are located in highly stable *β*-sheets regions at the core of the proteins. An important contributor to ligand stability is the presence of stable interactions with the active site pocket residues. Using LIGPLOT^+^, the hydrophobic and hydrogen contacts stabilizing the ligands were evaluated at different MD simulation time steps. Key hydrophobic *pi-pi* stack and hydrogen bonds identified at the docking level were maintained during the simulations, a possible explanation of the minimal fluctuations observed during simulations (Table S4). Just like in the docking studies, the plasmodial proteases had more interactions, especially with the PubChem compounds (Table 2 and Table S4).

### 2.5. Key Residues Involved in the Protein Stabilization of Selected Compounds Are Identified via Binding Free Energy

**Figure 6:**
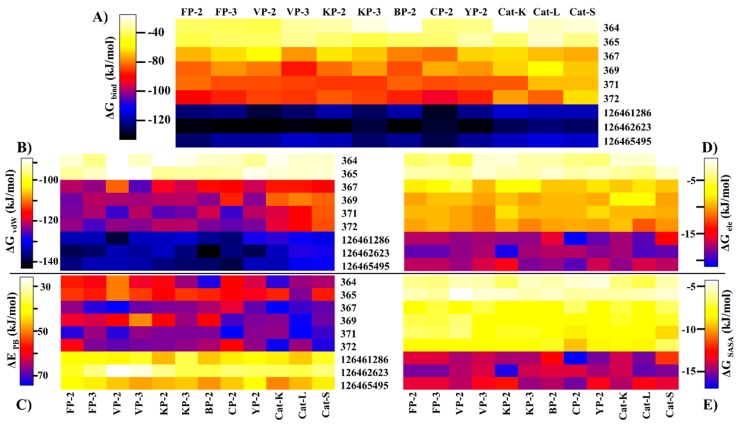
Heatmaps representing the (**A**) binding free energies of the protein-ligand complexes and the contributions from individual energetic components: (**B**) van der Waals (vdW); (**C**) polar solvation (PB); (**D**) electrostatics (ele); and (**E**) solvation energy (SASA).

The determination of the binding free energy of protein–ligand complexes remains an important aspect in structure-based drug design [68,69]. Despite the incapacity to determine the entropic contribution of ligand binding, several computational approaches that estimate the affinity of small molecules with proteins have been developed [70,71]. In the current work, the MMPBSA method [72] was used to estimate the binding affinity of the identified hits with the different proteins. A high correlation between docking results and *ΔG_bind_* energy was observed (Figure 6A). As observed with the docking studies, both SANC00364 and SANC00365 had the lowest affinity with the different proteins due to the limited residue interactions they formed. Strong binding affinities were observed with the rest of the SANCDB hits, especially SANC00369, SANC00371 and SANC00372. These compounds had a high number of hydrophobic contacts and several hydrogen bonds with important active pocket residues of the various proteins due to their size. Additionally, the compounds had a differential affinity of >20.0 kJ/mol between the plasmodial and human proteins. The strongest binding affinity was recorded with the PubChem compounds, which had *ΔG_bind_* energy < −100.0 kJ/mol in both the plasmodial and human proteins. However, they showed minimal selectivity on the human cathepsins.

Decomposition of the net affinity of each protein–ligand complex to the various energetic components revealed that binding was mainly promoted by vdW while polar solvation was the greatest impediment (Figure 6B,C). The magnitude of the vdW component on a protein is directly influenced by the number of hydrophobic effects a ligand has with its residues as determined by its size and chemical composition. Due to the limited contacts by SANC00364 and SANC00365 with the various proteins, the vdW contribution was the least compared to the rest of the compounds. The SANCDB exhibited weaker electrostatic and SASA contributions as compared to the PubChem analogs (Figure 6B,C and Table S5).

**Figure 7:**
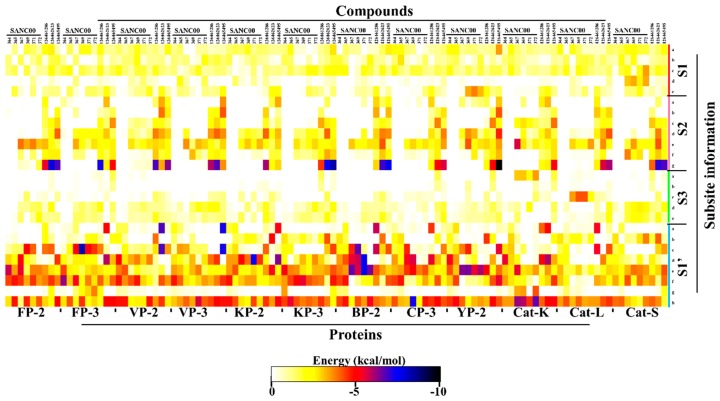
Per-residue decomposition of the net binding free energy (BFE) showing the contributions from each subsite residue towards ligand binding. The details (name and number) of the subsite residues (shown in letters) are listed in Table S6.

To gain more insight on the important residues contributing to the binding process, *ΔG_bind_* energy was further decomposed to gain an energy contribution from each residue. The obtained results were compared with the interaction fingerprint for each ligand obtained from the docking results (Table S2) in order to identify the key residues involved in stabilization of the ligands. From Table S2, it was observed that all the ligands established several interactions with S1 polar residues. However, per-residue energy decomposition results (Figure 7) revealed that most of these interactions were either unfavorable or resulted in bond energy of > −3.0 kcal/mol. Uniquely, the highly conserved Glu^a^ and Gly^c^ residues formed contacts of energy order < −3.0 kcal/mol in most of the proteins. In S1′, most of the compounds established strong contacts (< −4.0 kcal/mol) with the highly conserved His (S1′^f^) and hydrophobic Trp (S1′^h^) residues located at the floor of the pocket (Figure 7). The ring structure in these compounds facilitated the formation of strong interactions with the Trp side chain. The details (name and position) of the different subsite residues represented in letters (Figure 7) are found in Table S6.

All the other compounds, with an exception of SANC00364 and SANC00365, formed contacts of varied magnitude with S2 residues. This was because of the small chemical structure of these two compounds. The rest of the SANCDB compounds had limited contacts with S2, especially with only the residues forming its entrance. However, due to the extended nature of the PubChem compounds, strong contacts were observed with the residues forming the deep end of S2, especially in the plasmodial proteases. Several contacts of lesser magnitude and unfavorable energy contributions were observed with S1 and S3 residues and depended on the size of the ligands. For S2, the highly conserved catalytic triad Asn^e^ residues formed interactions with most of the ligands mainly through hydrogen bonds. In S3, the main contribution (weak) was through the highly conserved Gly^d,e^ residues. The current results are in agreement with our previous work using 2-cyanopyridine nitrile derivatives (CPs) where we identified that residues in S1 and S3 tend to form unfavorable contacts and should be avoided so as to increase the binding affinity. Moreover, to effectively inhibit the plasmodial proteases while maintaining a differential affinity binding with the human proteins, the designing of potential hits should target S2 residues and the highly varied portion of S1′ ^(a–e)^ (see Table S6).

### 2.6. Important Residues in Protein Communication and More Evidence for Allosteric Sutes are Identified via Dynamic Residue Network Analysis

To determine the important residues that are crucial for protein communication the *average L* and *average BC* of each complex and the corresponding apo form were calculated using MD-TASK [73]. Additionally, the effect of ligand binding on *average BC* and *average L* (*ΔBC* and *ΔL*) per each residue was also calculated by determining the difference between that of the apo and ligand-bound complexes (apo-complex). Recently, this approach has been successfully applied in several studies resulting in the identification of key residues that are important communication hubs in biological systems as well as those that mediate allosteric regulation [67,73,74].

**Figure 8:**
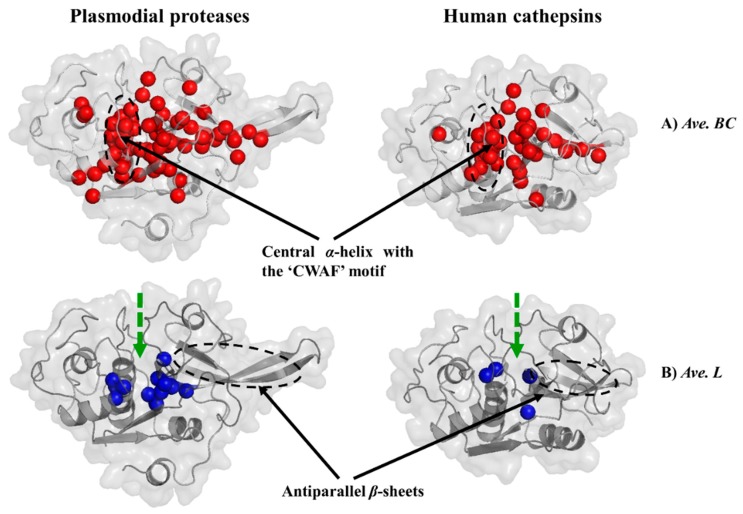
Key communication hubs in plasmodial proteases and their human homologs. Structural mapping of residues with significantly high *average BC* (**A**, red) and low *average L* (**B**, blue) values in plasmodial proteases and human cathepsins in both ligand-bound and ligand-free states. Active site location is shown by the thick green broken line.

*Average L* of a residue describes its ability to communicate with other residues within a network [75]. *Average BC* describes how frequently a residue is utilized in the shortest path navigation, thus reflecting on its influence and role in a network. Nodes with a high *BC* are of interest as they regulate information flow within important communication paths. A cut-off value of one and half times standard deviation away from the mean was used to determine residues with significantly high *average BC* and low *average L* values. In both apo and ligand bound systems, a cluster of conserved residues in the plasmodial as well human proteases and located at the central *α*-helix containing the “CWAF” motif (residues 42–55 (FP-2 catalytic domain numbering)) surrounding the active site were found to possess high *BC* values (Figure 8A, Figure S16–S28 and Table 3A). Additionally, a portion of the antiparallel *β*-sheets was also found to have residues with high *BC* values. However, human cathepsins had fewer number of residues in this region compared to the plasmodial proteases, which may be linked to the shorter *β*-sheets as they lack the arm structure (*β*-hairpin). A distinct feature is the high variation of the residues forming the *β*-sheets between the plasmodial and human homologs. However, in each group, these residues are highly conserved. Most of the catalytic triad residues as well as a few of the subsite residues had high *BC* scores, possibly due to their integral catalytic function. These included: C42, S149, A151, S153, N204, W206 (FP-2); C44, S151, H176, N206, W208 (FP-3); C43, S150, N205, W207 (VP-2); C42, S149, A175, N204, W206 (VP-3); C43, S150, A176, N205, W207 KP-2); C41, N148, A174, N203, W205 (KP-3); C43, N148, A176, N205, W207 (BP-2); C43, A150, A176, N205, W207 (CP-2); C43, A150, A176, N205, W207 (YP-2); C25, Y67, A134, H162, N182, W184 (Cat-K); C26, A136, A137, D138, G140, G165, N188, W190 (Cat-L); C25, G137, N138, D139, R141, G165, N184, W1186 (Cat-S). All the identified residues displaying high *BC* values experienced minimal fluctuations (as determined by RMSF. Figure S16–S28). The inverse correlation between two metrics, RMSF and *BC*, especially in relatively rigid proteins, was previously shown by Penkler et al. [74]. The majority of these residues were found in and around the binding active pocket (Figure S16–S28). For *average L*, several residues deeply buried within the central core were identified to possess significantly lower values (Figure 8B, Figure S16–S28 and Table 3B). In terms of the cluster size, the human cathepsins had a fewer number of these residues as compared to the plasmodial homologs. Both the apo and ligand-bound systems showed a similar topology, an indication of little or a null effect of the presence of ligands on the active site.

However, by determining the change of *average BC* and *average L* (*ΔBC* and *ΔL*) between the apo and corresponding receptor ligand complexes, a range of residues were observed that experienced significant changes (increase and decrease) in the *average shortest path* and *betweenness of centrality* (Figure S29, Table S7 and Figure S30–S41). These residues were mainly in the *β*-sheet and the region between S1 residues and the central *α*-helix containing the “CWAF” motif.

From these results, ligand binding seems to affect the communication network not only on the residues around the active site but also those that are distal. This finding may be linked to the possible existence of allosteric signals between the active site and other regions of the proteins. Allosteric modulation in the papain-like group of enzymes (which includes the proteins under this study) has been previously reported. A recent study by Alvarez et al. reported the existence of two possible sites in cruzipain, a homolog of FP-2 from *Trypanosoma cruzi* [76]. Another study by Novinec et al. reported conformational variability of human Cat-K and related cathepsins using computational approaches and how chondroitin sulfate and two other small molecules (NSC13345 and NSC94914) modulate allosteric regulation on these proteins [77]. A recent study by Marques et al. suggested that heme, a toxic by-product of hemoglobin degradation may induce conformational changes forming a secondary binding pocket between the “trench-like” primary active pocket and *β*-hairpin [39]. Binding of heme to this potential allosteric site causes modifications in the primary site inhibiting substrate processing resulting to an allosteric regulation on FP-2 [39]. The formation of this shallow heme site is highly depended on conformational changes induced by the highly dynamic *β*-hairpin. Our DRN analysis showed that this region in plasmodial proteases had several residues with high *BC* values (Table 3) and corresponding low RMSF. In FP-2, these included A175-M183 and K196-W206. A similar observation was done in corresponding regions in the other plasmodial proteases. Additionally, two separate studies by Marques et al. [78] and Bertoldo et al. [79] have tried to identify inhibitors which can mimic heme activity. Using experimental techniques, the team by Bertoldo identified a chalcone natural compound that exhibited non-competitive inhibitory mechanism against FP-2. Using Trp intrinsic fluorescence assays, they suggested that the observed FP-2 inhibition was possibly through the disruption of the Trp206 and Trp211 interaction leading to conformational changes in the active site. From the current results, only Trp206, a highly conserved S1′ residue, showed high *BC* values. Through structure activity relationship studies using polysulfonated polyaromatic symmetrical urea (suramin) and analogs, Marques et al. identified a possible conformational change involving E171, N173-K184 and H197, resulting in the formation of a secondary binding site. From our results, these residues had high *BC* values accompanied by minimal fluctuations, an indication that these residues may be of a significant allosteric effect. Collectively, residues located in the *β*-sheet of plasmodial proteases may be involved in allosteric communication.

A strong correlation between *BC* and *L^−1^*, *BC* and *RMSF^−1^*, *L* and *RMSF* and *L^−1^* and *RMSF^−1^* was observed in all the systems (Table S8). The reverse correlation between *BC* and *RMSF* as well as *L* was, for the first time, shown in the Penkler et. al. study [74]. This reverse correlation is more prominent in the protein structures with a relatively rigid nature as it is observed in the case here.

## 3. Materials and Methods

**Figure 9:**
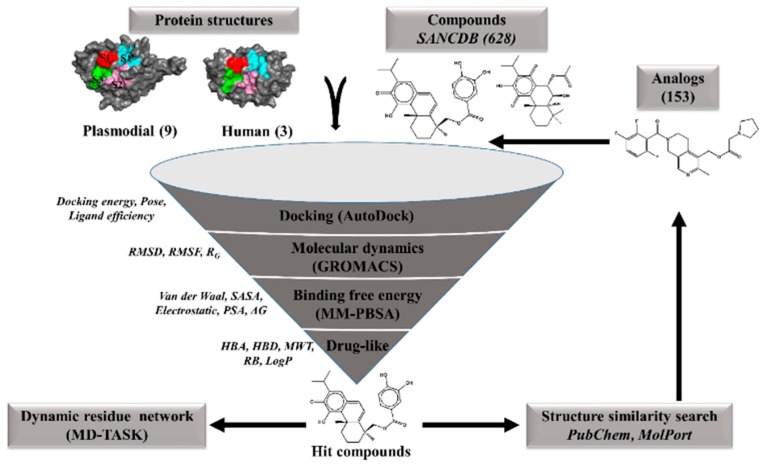
A graphical workflow of the computational approaches used in the identification of potential hits against *P. falciparum* FP-2 and FP-3 proteins and their homologs from other plasmodial species.

A graphical representation of the approaches used in this study is shown in Figure 9.

### 3.1. Identification of Hit Compounds from the South African Natural Compounds Database (SANCDB)

A total of 628 natural compounds from SANCDB were retrieved in the minimized format for docking studies. Following a previously reported approach [27,31], 7536 docking runs involving 12 (nine plasmodial and three human cathepsin—Figure 1) proteins were performed by AutoDock4.2 [80] on a Linux cluster. The catalytic domain protein structures of falcipains and human cathepsins (FP-2: 2OUL), (FP-3: 3BWK), (Cat-K: 3OVZ), (Cat-L: 3OF8) and (Cat-S: 1NPZ) were retrieved from the Brookhaven Protein Data Bank (PDB) [81]. The remaining plasmodial proteins were constructed using homology approaches as detailed in our previous study [27]. Initially, the accuracy of AutoDock was first determined by re-docking E-64, Leupeptin as well as K11017, the co-crystallized ligands in FP-2 (PDB: 3BPF) and FP-3 (PDB: 3BPM and 3BWK), respectively. The Gasteiger–Huckel method in the AutoDock tools (ADT version 1.5.6) was used to assign partial charges on the ligands. A cubic box of grid points 70, 70 and 65 along the x, y and z directions with a grid spacing of 0.375 Å was generated on the catalytic Cys residue of each receptor. Docking conformational search was performed using the Larmarckian genetic algorithm with each ligand being subjected to 100 simulations, maximum number of generations set at 27,000 and maximum energy evaluations of 450,000. Subsequently, the RMSD of the heavy atoms in the crystallographic ligands and the best re-docked pose were determined. The interaction fingerprint for each re-docked pose was also evaluated for consistency. To determine the sensitivity and specificity of the docking process, 30 compounds of diverse chemical nature and with IC_50_ ≤ 10 μM against FP-2 were compiled from the literature [37,52,54,79,82,83,84,85,86,87]. Fifty decoys per each active compound were generated from the directory of useful decoys (DUD-E) database [88] and together with the active compounds submitted to the docking process (Table S9). A Receiver Operating Characteristic (ROC) was established using the screening explorer webserver [49]. Finally, a structure-based virtual screening was performed on the SANCDB ligands. Based on the docking energy score, the best pose per ligand was selected and visualized using PyMOL. In house scripts using LigPlot^+^ subroutines [50] was utilized to determine protein–ligand interactions per each complex. Other drug-like filtering metrics, including ligand efficiency, rule of five (Ro5) and differential affinity binding between the plasmodial and human proteins, were applied leading to the identification of eight potential hits. A >80% structure similarity search within the PubChem and MolPort chemical databases to identify analogs using the identified hits was also performed. From 153 analogs obtained, a total of 1836 docking experiments were performed in a similar way as with the SANCDB compounds.

### 3.2. Molecular Dynamics and Trajectory Analysis

To analyze the conformational changes in the different protein–ligand complexes, all atom MD simulations of 100 nanoseconds (ns) were performed using GROMACS suite 2016.1 [65] with AMBER99 force field [89]. Using AnteChamber Python Parser interface (ACPYPE) [90], amber force field compatible topologies with correct atom types and charges for the different ligands (ACPYPE) were generated. The generated protein–ligand topology files were merged to form complexes which were solvated using the SPC water model in a triclinic box of dimension with a minimal distance of 1.0 nm between the complex and box edges. Resulting infinite systems were neutralized by replacing water molecules with a 0.15 M solution of NaCl and then relaxed using the steepest descent energy minimization algorithm without any constraints until a tolerance of 1000.0 kJ mol^−1^ nm^−1^ was obtained. A bi-phase (each 200 ps) canonical ensemble was used to equilibrate the systems; first under the NVT ensemble set at 300 K using Berendsen temperature coupling and then NPT ensemble at 1 atm in all directions at constant temperature of 300 K using the Parrinello–Rahman barostat algorithm [91]. Equilibrated systems were subjected to 100 ns production runs with an integration time step of 2 femtoseconds (fs) at a constant temperature and pressure. LINCS algorithm [92] was used to apply bond length constraints during equilibration and production runs. A particle-mesh Ewald algorithm [93] with a Fourier grid spacing of 0.16 nm was used for long-range electrostatics forces while cut-off distances for Coulomb and van der Waal interactions were set at 1.4 nm. Using the GROMACS tools *gmx rms* and *gmx rmsf*, the global stability and local residue fluctuations for both the protein–ligand complexes and ligand-free protein systems were evaluated. Using the *gmx trjconv* tool, system snapshots were generated at 5 ns intervals and the protein–ligand interactions in each complex determined using LigPlot^+^. Statistical analysis and plotting of the various properties were performed using R.

### 3.3. Binding Free Energy Calculations

To gain a further understanding about the stability of each of the various protein–ligand complexes, molecular mechanics Poisson–Boltzmann surface area (MM/PBSA) calculations were determined using the pre-compiled g_mmpbsa tool (version 1.6) [72] as previously described [27,31]. In summary, 1000 snapshot structures were extracted from the last 20 ns per each protein–ligand trajectory and the BFE (*ΔG_bind_*) per individual complex calculated. This approach applies a thermodynamic cycle using the single trajectory approach to approximate *ΔG_bind_* where the polar contribution is modelled by the Poisson–Boltzmann continuous model while the non-electrostatic solvation component through the solvent accessible surface area (SASA). The solvent, solute and vacuum dielectric constants were set as 80, 2 and 1, respectively. As outlined by Kollman et al. [94], the following set of equations are applied:

(1) $$\Delta G_{bind}\;=\;G_{complex}-\left(G_{receptor}+G_{ligand}\right)$$

(2) $$\Delta G_{bind}\;=\;E_{gas}+G_{sol}-T\Delta S$$

(3) $$E_{gas}\;=\;E_{int}+E_{vdw}+E_{ele}$$

(4) $$G_{sol}\;=\;G_{pol}+G_{SA}$$

(5) $$G_{SA}\;=\;\gamma_{SASA}+b$$

where *G_complex_*, *G_receptor_* and *G_ligand_* denotes the absolute free energies for each complex, the ligand-free protein and ligand-only, respectively. The *ΔG_bind_* individual contributions include three components: A gas-phase energy (*E_gas_*), which is a sum of bonded (*E_int_*) and nonbonded terms (*E_vdw_* and *E_ele_*); the solvation free energy (*G_sol_*), which is sum of polar (*G_pol_*) plus nonpolar (*G_SA_*) solvation energy components; and a conformational entropy term (*TΔS*) computed through normal-mode analysis of the conformational snapshots harvested from the simulation trajectory. The SASA model used to calculate *G_SA_* utilized an offset value (*b*) of 3.84928 kJ·mol^−1^ and surface tension proportionality (γ) of 0.0226778 kJ·mol^−1^·Å^−2^. The individual contributions of the protein residues to the three energetic components were determined through per-residue decomposition.

### 3.4. Dynamic Residue Network Analysis

To determine the effect of ligand binding on the residue communication flow in each protein–ligand complex during simulations, MD-TASK [73] was used to calculate the dynamic residue network (DRN) graphs by using every 100th frame in both apo and ligand bound protein system MD trajectories. In this network approach, the C*_α_* (for Gly) and C*_β_* for other residues are treated as nodes and the inter-node edge distance represent residue interactions. From the resulting DRN, a *N × N* matrix was calculated for each case, and the *shortest path length* (*L*) and *betweenness centrality* (*BC*) determined using the default cut-off off edge distance connecting two nodes of 6.7 Å. *L* represents the number of nodes that need to be traversed between two residues *i* and *j* while *BC* is a measure of how frequent a node occurs in the shortest path length between two nodes. Using a custom algorithm in MD-TASK, *average L* per each node is calculated by determining the sum of the shortest paths to that reference residue from all the other residues divided by the total number of residues minus one. By calculating the average of these metrics, important residues controlling communication flow within communication paths can be determined over an MD simulation. Data normalization for *BC* and *L* was performed using the following equations:

(6) $$Z_{Apo\;}=\;\frac{Apo-min\left(Apo,Complex\right)}{max\left(Apo,Complex\right)-min\left(Apo,Complex\right)}$$

(7) $$Z_{\mathrm{Complex}}\;=\;\frac{\mathrm{Complex}-min\left(Apo,Complex\right)}{max\left(Apo,Complex\right)-min\left(Apo,Complex\right)}$$

where *Z_Apo_* and *Z_Complex_* is the normalized *average L* and *average BC* results for the apo and complex systems, respectively.

To identify the effect of ligand binding on proteins, *ΔL* and *ΔBC* metrics were calculated using the corresponding apo receptors as the reference (*average L* (apo) − *average L* (corresponding ligand bound system); *average BC* (apo) − *average BC* (corresponding ligand bound system)). Residues showing a significant change (>1.5 times the standard deviation away from the mean) were determined. The relationship between the *average L* and *average BC* with the local per residue fluctuation (RMSF from trajectory analysis) was determined by calculating the Pearson correlation coefficient values between these three metrics. Analysis was performed using trajectories of 100 ns for both the ligand-bound and corresponding ligand-free systems.

## 4. Conclusions

The continued search for novel antimalarial compounds has been warranted by constant emergence of drug-resistant plasmodia strains [95]. The current work uses computational approaches to screen natural compounds from South Africa for potential hits against *Plasmodium* proteases responsible for hemoglobin degradation. We identified seven diterpenoids that have interesting interaction profiles with the different subsite residues in plasmodial hemoglobinases. Although a previous study had shown these compounds to possess strong antimalarial properties [38], their specific target protein and mechanism had not been elucidated yet. Five of these hits exhibited strong binding affinities against plasmodial proteases involved in hemoglobin degradation with SANC00369, with SANC00371-373 showing some level of selectivity towards human cathepsins. Compared to 2-cyanopyrimidine nitriles [52] (the best known non-peptide inhibitors developed hitherto), the current set of hits lacked the essential pharmacophoric features necessary for effective inhibition as outlined in previous studies [51,52]. To enhance the hemoglobinases inhibitory potency of the identified natural compounds, further hit modifications to align with the essential features of already known inhibitors are necessary. Considering the shape and physicochemical properties of the different subsite pockets as influenced by their residue composition, derivatives of these compounds should be able to fit well and establish strong interactions with key residues across the S2 and S1′ subsites of the plasmodial proteases. As a proof of concept, we utilized this information and performed minor modifications on SANC00371-373, where we introduced various groups known to promote interactions with S1′ and S2 of the plasmodial proteases. Ligand-based virtual screening with the modified SANCDB hits lead to the identification of three PubChem analogs with stronger binding affinities and residue interaction profiles similar to those seen with 2-cyanopyrimidines [27].

As the first study to apply DRN in the identification of important residue for communication in plasmodial hemoglobinases, the current study determined a cluster of residues between the primary subsite and *β*-hairpin structure of plasmodial proteases with significantly high *BC* values and minimal fluctuations. A previous study using in vitro techniques showed this region to be involved in the formation of a secondary site where heme binds and modulates the hemoglobinases activity via allosteric modulation [39]. This may hold the key to the development of selective antimalarial drugs, mimicking the allosteric effect of heme on the plasmodial hemoglobinases. This observation confirms the importance of DRN analysis in the identification of additional protein binding sites other than the main orthosteric sites that could be of biological importance. Recently, the search for allosteric modulators has drawn intensified research interest as they tend to be more specific compared to primary binding site inhibitors [96,97,98]. The existence of allosteric modulation in the papain-like group of enzymes has been reported in the recent past thus supporting a possible allosteric modulation of the proteases studied herein. Using the findings of the current study, further studies are necessary to identify potential allosteric modulators against the plasmodial parasite to determine their mode of action.

In conclusion, the identified natural compounds, especially SANC00371-373 and the PUBCHEM analogs, could provide potential templates for the development of novel antimalarial drugs. Additional hit optimization studies, mainly targeting the substitution of the hydroxyl groups with functional chemical groups, complying with the chemical properties as well as size of the different subsites, are necessary. Moreover, the elongation of the central ester group linker of the SANCDB hits might be critical to ensure the establishment of interactions with S2 residues, which are known to be important for selectivity. To ensure the stability of SANC00371-373 hits in vivo, additional modification by substituting the ester linkage with stronger groups such as amide linkages may be undertaken. The hit optimization for PUBCHEM hits should mainly focus on increasing their selectivity against human cathepsins.

## Figures and Tables

**Table 1:** Drug-like properties and pan assay interference compounds (PAINS) filtering of identified hits from the SANCDB and analogs from the PubChem database.

Compound ID	Chemical Formula	Lipinski’s Rule of Five (RO5)					PAINS
		Mol. wt	HBA	HBD	nRB	LogP	
SANC00364	C_22_H_30_O_6_	390.2	6	2	3	3.9	Fail
SANC00365	C_21_H_28_O_6_	376.2	6	2	3	3.6	Fail
SANC00367	C_27_H_30_O_5_	434.2	5	2	4	5.0	Pass
SANC00369	C_27_H_30_O_6_	450.2	6	3	4	5.4	Fail
SANC00371	C_25_H_32_O_4_	396.2	4	1	5	5.2	Pass
SANC00372	C_27_H_30_O_5_	434.2	5	2	5	4.7	Pass
SANC00373	C_27_H_30_O_6_	450.2	6	3	5	5.0	Fail
CID126461286	C_21_H_20_N_4_O_3_S	408.1	7	3	5	0.8	Pass
CID126462623	C_23_H_25_F_3_N_4_O_2_	446.2	6	1	7	-0.4	Pass
CID126465495	C_25_H_25_N_3_O_2_S	431.2	5	1	7	1.8	Pass

**Table 2:** Main hydrogen-forming residues during molecular dynamics in different proteins. Residue numbering is based on the catalytic domain (Table S1).

Protein	Compound	Residues
FP-2	SANC00364	C42, W206
	SANC00367	K37, C39, W207
	SANC00371	Q36, N38, C39, W206
	126462623	Q36, N81, G83, N173, W206
VP-2	SANC00364	D36, W207
	SANC00367	A38, H175, W207
	SANC00371	Q37, A38, W207
	126462623	Q37, G84, N174, W207
BP-2	SANC00364	Q37, W207
	SANC00367	Q37, E82, N174
	SANC00371	Q37, G84, W207
	126462623	Q37, G84, N174, W207
Cat-K	SANC00364	W184
	SANC00367	Q19, W184
	SANC00371	Q19, W184
	126462623	Q19, W184
Cat-L	SANC00364	Q20
	SANC00367	N19, W190
	SANC00371	G21, W190
	126462623	Q20, G21

**Table 3:** Residues with high *average BC* values (**A**) and low *average L* (**B**) in both apo and ligand-bound systems. Residue numbering based on the catalytic domain length indicated in Table S1. Bold red shows cluster of residues with high *BC* values in both human and plasmodial proteins while bold black those only in plasmodial proteases.

Protein	Residues
***(A) Average BC***	
FP-2	**W24**, **T31**, **C42-Y55**, S66, Q68, V71, **N86, F89, E90**, I93, K126, P132, R141, **G144-S149**, A151, S153, **A175-M183, K196-W206**, N217, E219, D221, I237, P238
FP-3	**W26**, **T33**, **C44-Y57**, S68, Q70, V73, **T88, F91, D92**, I95, K128, I133, L142, R143, P147, **S149-S151**, **A176-G182**, G184, K186, **K198-W208**, N219, E221, D223, Y227, V239, P240
VP-2	**W25**, **T32**, **C43-Y56**, S67, Q69, V71, **P87, F90, E91**, I94, I126, I132, I141, R142, **G145-S150**, **A176-E185**, **K197-W207**, R218, E220, D222, Y226, V238, A239
VP-3	**W24**, **T31**, **C42-Y55**, S66, Q68, V71, **P86, F89, E90**, L93, I125, I131, I140, K141, **G144-S149**, **A175-E184**, **K196-W206**, K217, Q219, Y223, V237, A238
KP-2	**W25**, **T32**, **C43-Y56**, S67, Q69, V72, **P87, F90, E91**, I94, E131, I141, K142, **G145-S150**, **A176-E185**, **K197-W207**, K218, Q220, V238, A239
KP-3	**W23**, **T30**, **C41-Y54**, S65, Q67, V70, **P85, I88, E89**, I92, E129, V139, R140, **G143-N148**, **A174-E184**, **K195-W205**, R216, E218, D220, V237, L238
BP-2	**W25**, **I32**, **C43-Y56**, S67, Q69, V72, **P87, F90, E91**, S127, P133, Q142, **G145-V151**, **A176-V184**, **K197-W207**, R218, K220, N222, A237, P238
CP-2	**W25**, **I32**, **C43-Y56**, S67, Q69, V72, **P87, F90, E91**, I127, P133, Q142, **G145-V151**, **A176-V184**, **D197-W207**, R218, K220, D222, V237, P238
YP-2	W**25**, **I32**, **C43-Y56**, S67, Q69, V72, **P87, F90, E91**, S127, P133, Q142, **G145-V151**, **A176-V184**, **K197-W207**, R218, K220, N222, A237, P238
Cat-K	**C25-E35**, N52, Y67, N70, A124, **G129-S132**, A134, **N161-A166**, **K176-W184**, Y192
Cat-L	**C26-E36**, Q52, V55, D72, F75, Q76, A128, **S133-D138**, G140, **G165-G170**, **K182-W190**, Y199
Cat-S	**C25-E35**, Q51, V54, T72, F75, Q76, A129, **S135-D139**, R141, **G165-170**, **Y179-186**, R197
***(B) Average L***	
FP-2	S47, I48, S50, V51, **I146-S149, M177-G180, K203**
FP-3	S49, V50, S52, V53, **I148-S151, I179-G181, K205**
VP-2	T48, V49, V51, V52, **P146-V149, V177-V180, K204**
VP-3	T47, V48, V50, V51, **I146-S149, I177-G180, K203**
KP-2	T48, V49, V51, V52, **I147-S150, I178-G181, R204**
KP-3	T46, V47, V49, V50, **I145-N148, I176-G179, K202**
BP-2	T48, A49, V51, V52, **I147-A150, M178-G181, R204**
CP-2	T48, A49, V51, I52, **L147-A150, I178-G181, R204**
YP-2	T48, A49, V51, V52, **I147-A150, M178-G181, R204**
Cat-K	A27, F28, V131, V164
Cat-L	A31, T32, V135, V168
Cat-S	A30, V31, V136, V168

## Supplementary Materials

Supplementary materials can be found at https://www.mdpi.com/1420-3049/24/22/4036/s1. **Figure S1.** Binding modes and key residues involved in the stabilization of the different compounds inside the active pockets of the different proteins. Residue names are colour coded based on the subsite information (Red = S1, Pink= S2, Green = S3, Cyan = S1′ and Black = non-subsite). **Figure S2:** Time dependent evolution of RMSD (A) and RMSF (B) plots of FP-2 during 100 ns simulation. Color code: Black = Apo; Blue = Complex and Green = Ligand only. **Figure S3:** Time dependent evolution of RMSD (A) and RMSF (B) plots of FP-3 during 100 ns simulation. Color code: Black = Apo; Blue= Complex and Green = Ligand only. **Figure S4:** Time dependent evolution of RMSD (A) and RMSF (B) plots of VP-2 during 100 ns simulation. Color code: Black = Apo; Blue= Complex and Green = Ligand only. **Figure S5:** Time dependent evolution of RMSD (A) and RMSF (B) plots of VP-3 during 100 ns simulation. Color code: Black = Apo; Blue= Complex and Green = Ligand only. **Figure S6:** Time dependent evolution of RMSD (A) and RMSF (B) plots of KP-2 during 100 ns simulation. Color code: Black = Apo; Blue= Complex and Green = Ligand only. **Figure S7:** Time dependent evolution of RMSD (A) and RMSF (B) plots of KP-3 during 100 ns simulation. Color code: Black = Apo; Blue= Complex and Green = Ligand only. **Figure S8:** Time dependent evolution of RMSD (A) and RMSF (B) plots of BP-2 during 100 ns simulation. Color code: Black = Apo; Blue= Complex and Green = Ligand only. **Figure S9:** Time dependent evolution of RMSD (A) and RMSF (B) plots of CP-2 during 100 ns simulation. Color code: Black = Apo; Blue= Complex and Green = Ligand only. **Figure S10:** Time dependent evolution of RMSD (A) and RMSF (B) plots of YP-2 during 100 ns simulation. Color code: Black = Apo; Blue= Complex and Green = Ligand only. **Figure S11:** Time dependent evolution of RMSD (A) and RMSF (B) plots of Cat-K during 100 ns simulation. Color code: Black = Apo; Blue= Complex and Green = Ligand only. **Figure S12:** Time dependent evolution of RMSD (A) and RMSF (B) plots of Cat-L during 100 ns simulation. Color code: Black = Apo; Blue= Complex and Green = Ligand only. **Figure S13:** Time dependent evolution of RMSD (A) and RMSF (B) plots of Cat-S during 100 ns simulation. Color code: Black = Apo; Blue= Complex and Green = Ligand only. **Figure S14.** Cα protein backbone RMSD distribution histogram plots for apo and ligand bound protein systems. The effect of ligand binding on the global conformational variation for each protein can be determined by comparing the mean (μ) of the complex (dashed red line) and that of the corresponding apo system. σ denotes the conformational distribution standard deviation over the last 70 ns of simulation. **Figure S15.** Ligand backbone RMSD distribution plots for the various ligands bound to plasmodial and human proteins. The dashed red line indicates the RMSD mean (μ) per each ligand while σ denotes the standard deviation during the last 70 ns of simulation. **Figure S16.** The effect of ligand binding on the fluctuations of each protein residue as determined by Root Mean Square Fluctuations (RMSF). Marked in red wedges are the catalytic triad (Cys-His-Asn) residues of each protein. The colour bars at the top correspond to the protein loops as shown on the right. **Figure S17:** Dynamic residue network analysis (*betweenness of centrality* and *average shortest path*) of FP-2 in the presence of different ligands. Color code: Black = Apo; Red= Complex. The location of residues with significant high *average BC* and low *average L* score are shown in red and blue on the protein structure. **Figure S18:** Dynamic residue network analysis (*betweenness of centrality* and *average shortest path*) of FP-3 in the presence of different ligands. Color code: Black = Apo; Red= Complex. The location of residues with significant high *average BC* and low *average L* score are shown in red and blue on the protein structure. **Figure S19:** Dynamic residue network analysis (*betweenness of centrality* and *average shortest path*) of VP-2 in the presence of different ligands. Color code: Black = Apo; Red= Complex. The location of residues with significant high *average BC* and low *average L* score are shown in red and blue on the protein structure. **Figure S20:** Dynamic residue network analysis (*betweenness of centrality* and *average shortest path*) of VP-3 in the presence of different ligands. Color code: Black = Apo; Red= Complex. The location of residues with significant high *average BC* and low *average L* score are shown in red and blue on the protein structure. **Figure S21:** Dynamic residue network analysis (*betweenness of centrality* and *average shortest path*) of KP-2 in the presence of different ligands. Color code: Black = Apo; Red= Complex. The location of residues with significant high *average BC* and low *average L* score are shown in red and blue on the protein structure. **Figure S22:** Dynamic residue network analysis (*betweenness of centrality* and *average shortest path*) of KP-3 in the presence of different ligands. Color code: Black = Apo; Red= Complex. The location of residues with significant high *average BC* and low *average L* score are shown in red and blue on the protein structure. **Figure S23:** Dynamic residue network analysis (*betweenness of centrality* and *average shortest path*) of BP-2 in the presence of different ligands. Color code: Black = Apo; Red= Complex. The location of residues with significant high *average BC* and low *average L* score are shown in red and blue on the protein structure. **Figure S24:** Dynamic residue network analysis (*betweenness of centrality* and *average shortest path*) of CP-2 in the presence of different ligands. Color code: Black = Apo; Red= Complex. The location of residues with significant high *average BC* and low *average L* score are shown in red and blue on the protein structure. **Figure S25:** Dynamic residue network analysis (*betweenness of centrality* and *average shortest path*) of YP-2 in the presence of different ligands. Color code: Black = Apo; Red= Complex. The location of residues with significant high *average BC* and low *average L* score are shown in red and blue on the protein structure. **Figure S26:** Dynamic residue network analysis (*betweenness of centrality* and *average shortest path*) of Cat-K in the presence of different ligands. Color code: Black = Apo; Red= Complex. The location of residues with significant high *average BC* and low *average L* score are shown in red and blue on the protein structure. **Figure S27:** Dynamic residue network analysis (*betweenness of centrality* and *average shortest path*) of Cat-L in the presence of different ligands. Color code: Black = Apo; Red= Complex. The location of residues with significant high *average BC* and low *average L* score are shown in red and blue on the protein structure. **Figure S28:** Dynamic residue network analysis (*betweenness of centrality* and *average shortest path*) of Cat-S in the presence of different ligands. Color code: Black = Apo; Red= Complex. The location of residues with significant high *average BC* and low *average L* score are shown in red and blue on the protein structure. **Figure S29.** Effect of ligand binding on *BC* (A) and *L* (B) using the apo structures as the reference. Shaded are regions of the protein that showed significant changes (one and half times away from the means of the ligand bound systems) in *BC* (red) and *L* (blue). **Figure S30:** Plots showing FP-2 residues with significant changes in *average* a) *BC* and B) *L* upon ligand binding. **Figure S31:** Plots showing FP-3 residues with significant changes in *average* a) *BC* and B) *L* upon ligand binding. **Figure S32:** Plots showing VP-2 residues with significant changes in *average* a) *BC* and B) *L* upon ligand binding. **Figure S33:** Plots showing VP-3 residues with significant changes in *average* a) *BC* and B) *L* upon ligand binding. **Figure S34:** Plots showing KP-2 residues with significant changes in *average* a) *BC* and B) *L* upon ligand binding. **Figure S35:** Plots showing KP-3 residues with significant changes in *average* a) *BC* and B) *L* upon ligand binding. **Figure S36:** Plots showing BP-2 residues with significant changes in *average* a) *BC* and B) *L* upon ligand binding. **Figure S37:** Plots showing CP-2 residues with significant changes in *average* a) *BC* and B) *L* upon ligand binding. **Figure S38:** Plots showing YP-2 residues with significant changes in *average* a) *BC* and B) *L* upon ligand binding. **Figure S39:** Plots showing Cat-K residues with significant changes in *average* a) *BC* and B) *L* upon ligand binding. **Figure S40:** Plots showing Cat-L residues with significant changes in *average* a) *BC* and B) *L* upon ligand binding. **Figure S41:** Plots showing Cat-S residues with significant changes in *average* a) *BC* and B) *L* upon ligand binding. **Table S1.** Position of the catalytic domain of all proteins used and the corresponding domain numbering. **Table S2.** Residue interaction fingerprint between the different proteins and ligands. Shown in red, pink, green and cyan are residues interacting with S1, S2, S3 and S1′ respectively. In black represent non-subsite residues. Residues are numbered according to the catalytic domain (**Table S1**). **Table S3.** RMSD distribution statistics. Apo and ligand bound RMSD means were compared using the z-test with α = 0.05 and a H_0_ of *μ1* –*μ2* = 0. The two-sample KS-test was used to compare the shapes of the distributions between the apo and ligand bound systems *μ* = Mean, σ = Standard deviation and σ^2^ = Variance. **Table S4:** Ligand-residue interaction fingerprint at different MD simulation time steps. VDR and HBR indicate the number of residues involved in van der Waals (hydrophobic) interactions and those involved in hydrogen (HBR) bonding respectively. Residues are numbered based on the catalytic domain length of each protein (**Table S1**). **Table S5:** Protein-ligand complex binding free energy terms in kJ/mol as determined by molecular mechanics Poisson-Boltzmann surface area (MM-PBSA) analysis. vdW=van der Waals forces, ele=electrostatics, PB=polar solvation energy, SASA=Soluble Accessible Surface Area, bind=binding free energy. **Table S6:** Subsite residue composition information (details and position). Residue numbering based on the catalytic domain length of individual proteins as indicated in **Table S1**. **Table S7.** Residues with significant *ΔBC* (A) and *ΔL* (B). Apo systems were used as the reference structures. A cut off value of one and half times standard deviation of the means of the difference with the various ligands used to define residues with significant changes in *average BC* and *average L*. Residue numbering based on the catalytic domain length indicated in **Table S1**. **Table S8.** Association of the various protein dynamic residue network metrics for proteins bound to different compounds as determined by Pearson’s correlation coefficient. **Table S9.** Active compounds against FP-2 and decoys from DUD-E used for docking protocol validation.
