# Supplementary material for: South African Abietane Diterpenoids and Their Analogs as Potential Antimalarials: Novel Insights from Hybrid Computational Approaches
Source: Molecules. 2019 Nov 7;24(22):4036. doi: 10.3390/molecules24224036 (PMC6891524; doi:10.3390/molecules24224036)
Supplement: Supplementary file 1 [file molecules-24-04036-s001.pdf]

Supplementary Material

South African abietene diterpenoids and their analogs as potential antimalarials: Novel insights from hybrid computational approaches

Thommas M. Musyoka & Özlem Tastan Bishop*

Research Unit in Bioinformatics (RUBi), Department of Biochemistry and Microbiology, Rhodes University, Grahamstown, 6140, South Africa

Author e-mail address:

Thommas Mutemi Musyoka: mutemibiochemistry@gmail.com

Özlem Tastan Bishop: O.TastanBishop@ru.ac.za

Corresponding author details:

Özlem Tastan Bishop: +27-466-038-072, O.TastanBishop@ru.ac.za

Table of contents

Figure S1. Binding modes and key residues involved in the stabilization of the different compounds inside the active pockets of the different proteins. Residue names are colour coded based on the subsite information (Red = S1, Pink= S2, Green = S3, Cyan = S1' and Black = non-subsite). 6

Figure S2: Time dependent evolution of RMSD (A) and RMSF (B) plots of FP-2 during 100 ns simulation. Color code: Black = Apo; Blue= Complex and Green = Ligand only. 7

Figure S3: Time dependent evolution of RMSD (A) and RMSF (B) plots of FP-3 during 100 ns simulation. Color code: Black = Apo; Blue= Complex and Green = Ligand only. 8

Figure S4: Time dependent evolution of RMSD (A) and RMSF (B) plots of VP-2 during 100 ns simulation. Color code: Black = Apo; Blue= Complex and Green = Ligand only. 9

Figure S5: Time dependent evolution of RMSD (A) and RMSF (B) plots of VP-3 during 100 ns simulation. Color code: Black = Apo; Blue= Complex and Green = Ligand only. 10

Figure S6: Time dependent evolution of RMSD (A) and RMSF (B) plots of KP-2 during 100 ns simulation. Color code: Black = Apo; Blue= Complex and Green = Ligand only. 11

Figure S7: Time dependent evolution of RMSD (A) and RMSF (B) plots of KP-3 during 100 ns simulation. Color code: Black = Apo; Blue= Complex and Green = Ligand only. 12

Figure S8: Time dependent evolution of RMSD (A) and RMSF (B) plots of BP-2 during 100 ns simulation. Color code: Black = Apo; Blue= Complex and Green = Ligand only. 13

Figure S9: Time dependent evolution of RMSD (A) and RMSF (B) plots of CP-2 during 100 ns simulation. Color code: Black = Apo; Blue= Complex and Green = Ligand only. 14

Figure S10: Time dependent evolution of RMSD (A) and RMSF (B) plots of YP-2 during 100 ns simulation. Color code: Black = Apo; Blue= Complex and Green = Ligand only. 15

Figure S11: Time dependent evolution of RMSD (A) and RMSF (B) plots of Cat-K during 100 ns simulation. Color code: Black = Apo; Blue= Complex and Green = Ligand only. 16

Figure S12: Time dependent evolution of RMSD (A) and RMSF (B) plots of Cat-L during 100 ns simulation. Color code: Black = Apo; Blue= Complex and Green = Ligand only. 17

Figure S13: Time dependent evolution of RMSD (A) and RMSF (B) plots of Cat-S during 100 ns simulation. Color code: Black = Apo; Blue= Complex and Green = Ligand only. 18

Figure S14. Cα protein backbone RMSD distribution histogram plots for apo and ligand bound protein systems. The effect of ligand binding on the global conformational variation for each protein can be determined by comparing the mean (μ) of the complex (dashed red line) and that of the corresponding apo system. σ denotes the conformational distribution standard deviation over the last 70 ns of simulation. 19

Figure S15. Ligand backbone RMSD distribution plots for the various ligands bound to plasmodial and human proteins. The dashed red line indicates the RMSD mean (μ) per each ligand while σ denotes the standard deviation during the last 70 ns of simulation. 20

Figure S16. The effect of ligand binding on the fluctuations of each protein residue as determined by Root Mean Square Fluctuations (RMSF). Marked in red wedges are the catalytic triad (Cys-His-Asn) residues of each protein. The colour bars at the top correspond to the protein loops as shown on the right. 21

Figure S17: Dynamic residue network analysis (*betweenness of centrality* and *average shortest path*) of FP-2 in the presence of different ligands. Color code: Black = Apo; Red= Complex. The location of

residues with significant high *average BC* and low *average L* score are shown in red and blue on the protein structure. 22

Figure S18: Dynamic residue network analysis (*betweenness of centrality* and *average shortest path*) of FP-3 in the presence of different ligands. Color code: Black = Apo; Red= Complex. The location of residues with significant high *average BC* and low *average L* score are shown in red and blue on the protein structure. 23

Figure S19: Dynamic residue network analysis (*betweenness of centrality* and *average shortest path*) of VP-2 in the presence of different ligands. Color code: Black = Apo; Red= Complex. The location of residues with significant high *average BC* and low *average L* score are shown in red and blue on the protein structure. 24

Figure S20: Dynamic residue network analysis (*betweenness of centrality* and *average shortest path*) of VP-3 in the presence of different ligands. Color code: Black = Apo; Red= Complex. The location of residues with significant high *average BC* and low *average L* score are shown in red and blue on the protein structure. 25

Figure S21: Dynamic residue network analysis (*betweenness of centrality* and *average shortest path*) of KP-2 in the presence of different ligands. Color code: Black = Apo; Red= Complex. The location of residues with significant high *average BC* and low *average L* score are shown in red and blue on the protein structure. 26

Figure S22: Dynamic residue network analysis (*betweenness of centrality* and *average shortest path*) of KP-3 in the presence of different ligands. Color code: Black = Apo; Red= Complex. The location of residues with significant high *average BC* and low *average L* score are shown in red and blue on the protein structure. 27

Figure S23: Dynamic residue network analysis (*betweenness of centrality* and *average shortest path*) of BP-2 in the presence of different ligands. Color code: Black = Apo; Red= Complex. The location of residues with significant high *average BC* and low *average L* score are shown in red and blue on the protein structure. 28

Figure S24: Dynamic residue network analysis (*betweenness of centrality* and *average shortest path*) of CP-2 in the presence of different ligands. Color code: Black = Apo; Red= Complex. The location of residues with significant high *average BC* and low *average L* score are shown in red and blue on the protein structure. 29

Figure S25: Dynamic residue network analysis (*betweenness of centrality* and *average shortest path*) of YP-2 in the presence of different ligands. Color code: Black = Apo; Red= Complex. The location of residues with significant high *average BC* and low *average L* score are shown in red and blue on the protein structure. 30

Figure S26: Dynamic residue network analysis (*betweenness of centrality* and *average shortest path*) of Cat-K in the presence of different ligands. Color code: Black = Apo; Red= Complex. The location of residues with significant high *average BC* and low *average L* score are shown in red and blue on the protein structure. 31

Figure S27: Dynamic residue network analysis (*betweenness of centrality* and *average shortest path*) of Cat-L in the presence of different ligands. Color code: Black = Apo; Red= Complex. The location of residues with significant high *average BC* and low *average L* score are shown in red and blue on the protein structure. 32

Figure S28: Dynamic residue network analysis (*betweenness of centrality* and *average shortest path*) of Cat-S in the presence of different ligands. Color code: Black = Apo; Red= Complex. The location of residues with significant high *average BC* and low *average L* score are shown in red and blue on the protein structure. 33

Figure S29. Effect of ligand binding on *BC* (A) and *L* (B) using the apo structures as the reference. Shaded are regions of the protein that showed significant changes (one and half times away from the means of the ligand bound systems) in *BC* (red) and *L* (blue). 34

Figure S30: Plots showing FP-2 residues with significant changes in *average* a) *BC* and B) *L* upon ligand binding. 35

Figure S31: Plots showing FP-3 residues with significant changes in *average* a) *BC* and B) *L* upon ligand binding. 36

Figure S32: Plots showing VP-2 residues with significant changes in *average* a) *BC* and B) *L* upon ligand binding. 37

Figure S33: Plots showing VP-3 residues with significant changes in *average* a) *BC* and B) *L* upon ligand binding. 38

Figure S34: Plots showing KP-2 residues with significant changes in *average* a) *BC* and B) *L* upon ligand binding. 39

Figure S35: Plots showing KP-3 residues with significant changes in *average* a) *BC* and B) *L* upon ligand binding. 40

Figure S36: Plots showing BP-2 residues with significant changes in *average* a) *BC* and B) *L* upon ligand binding. 41

Figure S37: Plots showing CP-2 residues with significant changes in *average* a) *BC* and B) *L* upon ligand binding. 42

Figure S38: Plots showing YP-2 residues with significant changes in *average* a) *BC* and B) *L* upon ligand binding. 43

Figure S39: Plots showing Cat-K residues with significant changes in *average* a) *BC* and B) *L* upon ligand binding. 44

Figure S40: Plots showing Cat-L residues with significant changes in *average* a) *BC* and B) *L* upon ligand binding. 45

Figure S41: Plots showing Cat-S residues with significant changes in *average* a) *BC* and B) *L* upon ligand binding. 46

Table S1. Position of the catalytic domain of all proteins used and the corresponding domain numbering. 47

Table S2. Residue interaction fingerprint between the different proteins and ligands. Shown in red, pink, green and cyan are residues interacting with S1, S2, S3 and S1' respectively. In black represent non-subsite residues. Residues are numbered according to the catalytic domain (**Table S1**). 48

Table S3. RMSD distribution statistics. Apo and ligand bound RMSD means were compared using the z-test with $\alpha = 0.05$ and a H_0 of $\mu 1 - \mu 2 = 0$. The two-sample KS-test was used to compare the shapes of the distributions between the apo and ligand bound systems μ = Mean, σ = Standard deviation and σ^2 = Variance. 52

Table S4: Ligand-residue interaction fingerprint at different MD simulation time steps. VDR and HBR indicate the number of residues involved in van der Waals (hydrophobic) interactions and those involved in hydrogen (HBR) bonding respectively. Residue are numbered based on the catalytic domain length of each protein (**Table S1**). 57

Table S5: Protein-ligand complex binding free energy terms in kJ/mol as determined by molecular mechanics Poisson-Boltzmann surface area (MM-PBSA) analysis. vdW=van der Waals forces,

ele=electrostatics, PB=polar solvation energy, SASA=Soluble Accessible Surface Area, bind=binding free energy. 62

Table S6: Subsite residue composition information (details and position). Residue numbering based on the catalytic domain length of individual proteins as indicated in **Table S1**. 64

Table S7. Residues with significant *ΔBC* (A) and *ΔL* (B). Apo systems were used as the reference structures. A cut off value of one and half times standard deviation of the means of the difference with the various ligands used to define residues with significant changes in *average BC* and *average L*. Residue numbering based on the catalytic domain length indicated in **Table S1**. 65

Table S8. Association of the various protein dynamic residue network metrics for proteins bound to different compounds as determined by Pearson's correlation coefficient. 67

Table S9. Active compounds against FP-2 and decoys from DUD-E used for docking protocol validation. 69

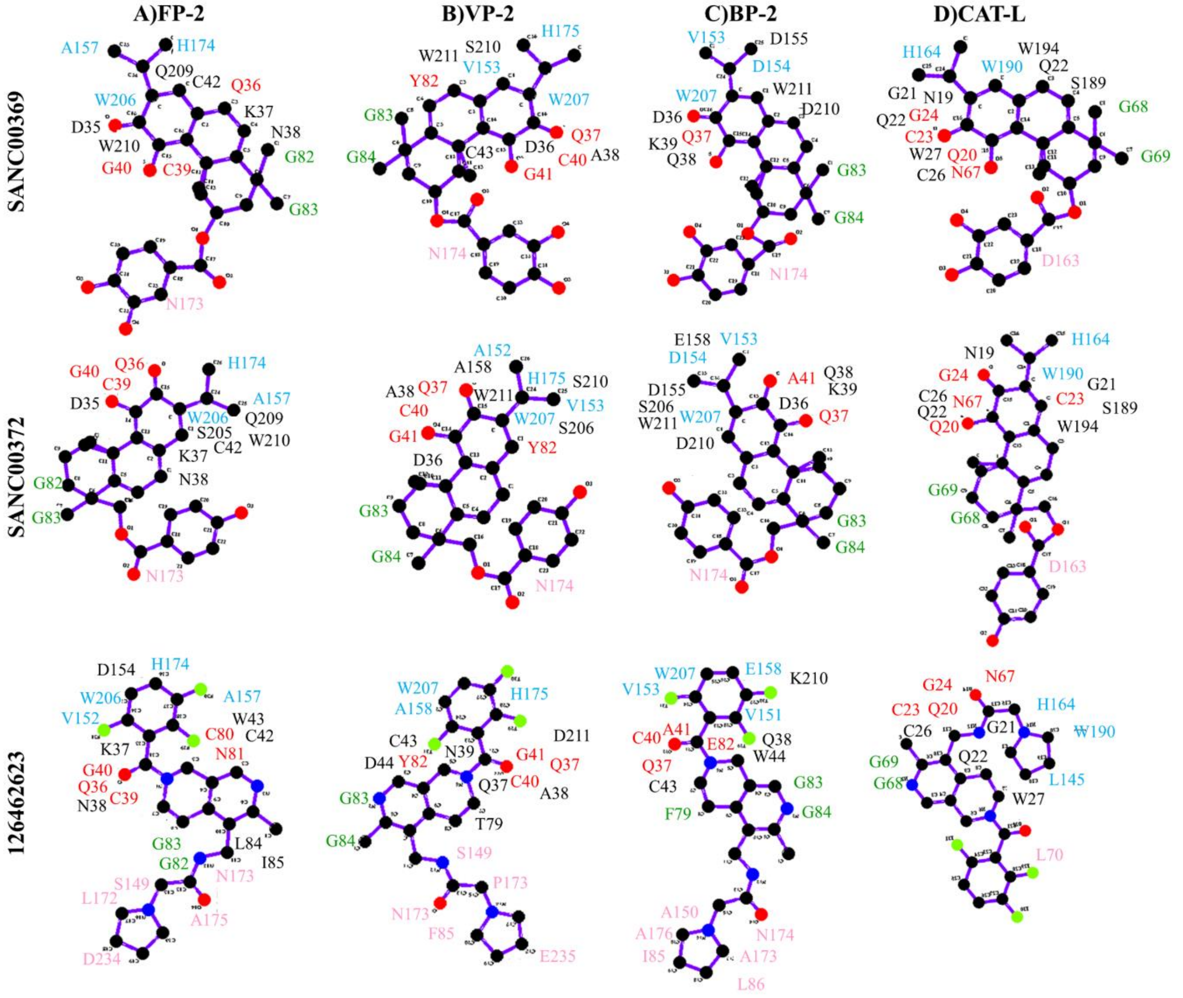

Figure S1. Binding modes and key residues involved in the stabilization of the different compounds inside the active pockets of the different proteins. Residue names are colour coded based on the subsite information (Red = S1, Pink= S2, Green = S3, Cyan = S1' and Black = non-subsite).

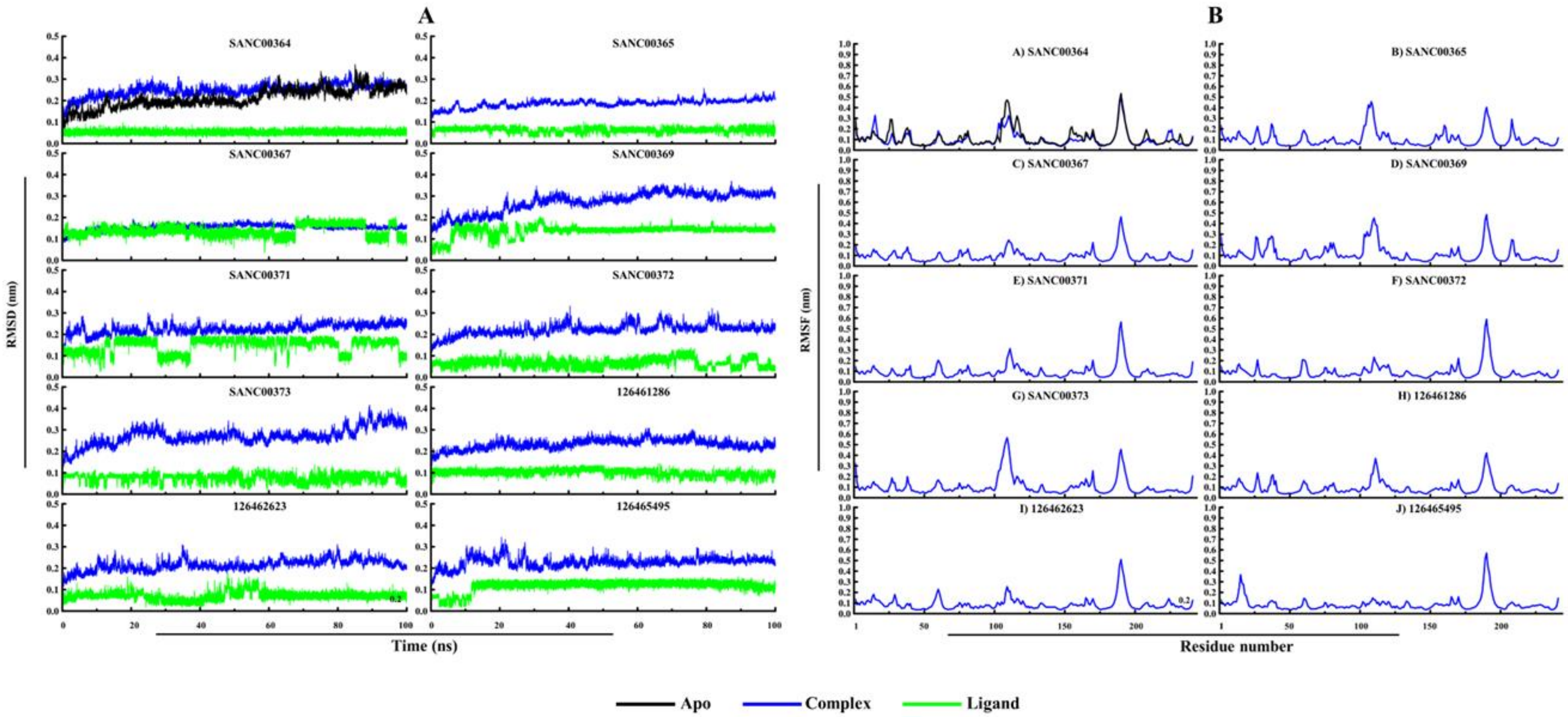

Figure S2: Time dependent evolution of RMSD (A) and RMSF (B) plots of FP-2 during 100 ns simulation. Color code: Black = Apo; Blue= Complex and Green = Ligand only.

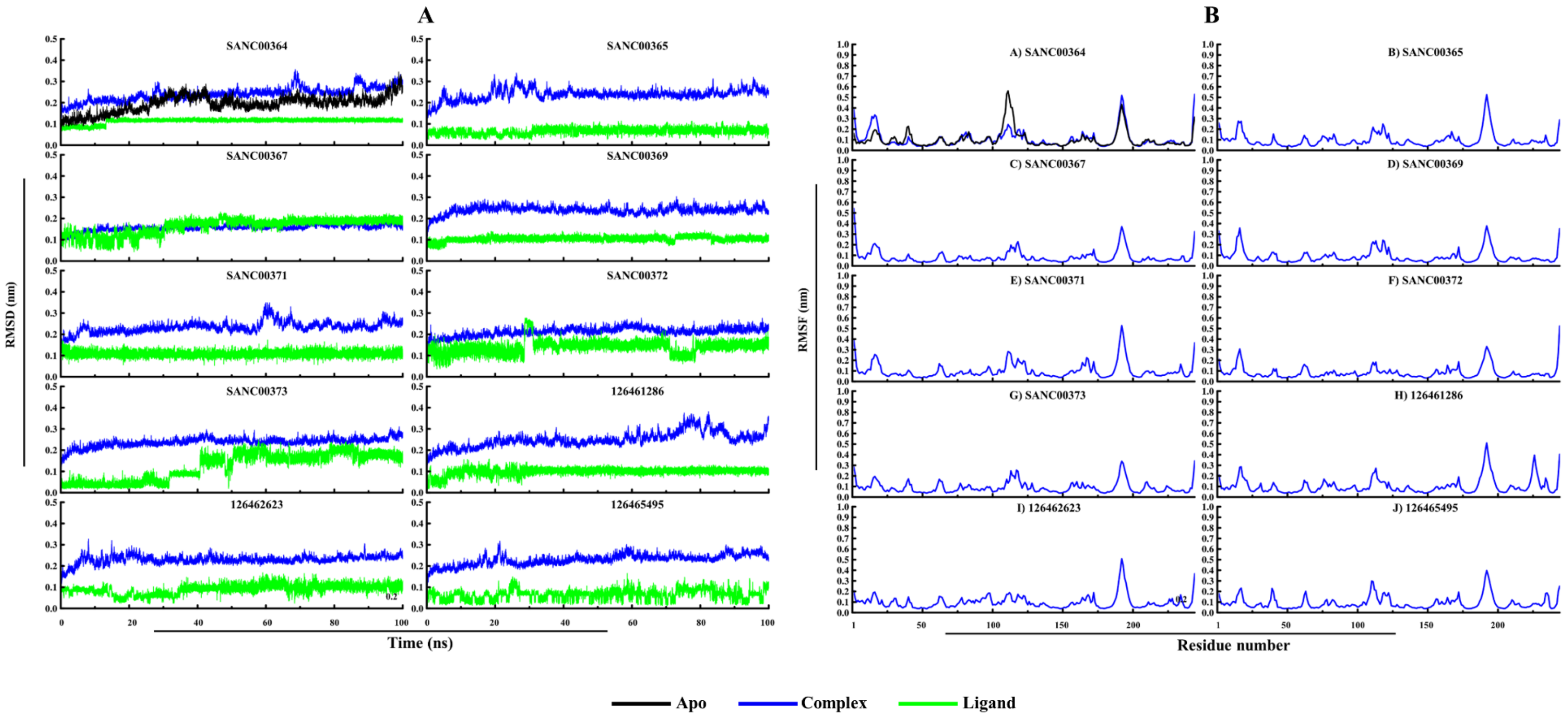

Figure S3: Time dependent evolution of RMSD (A) and RMSF (B) plots of FP-3 during 100 ns simulation. Color code: Black = Apo; Blue= Complex and Green = Ligand only.

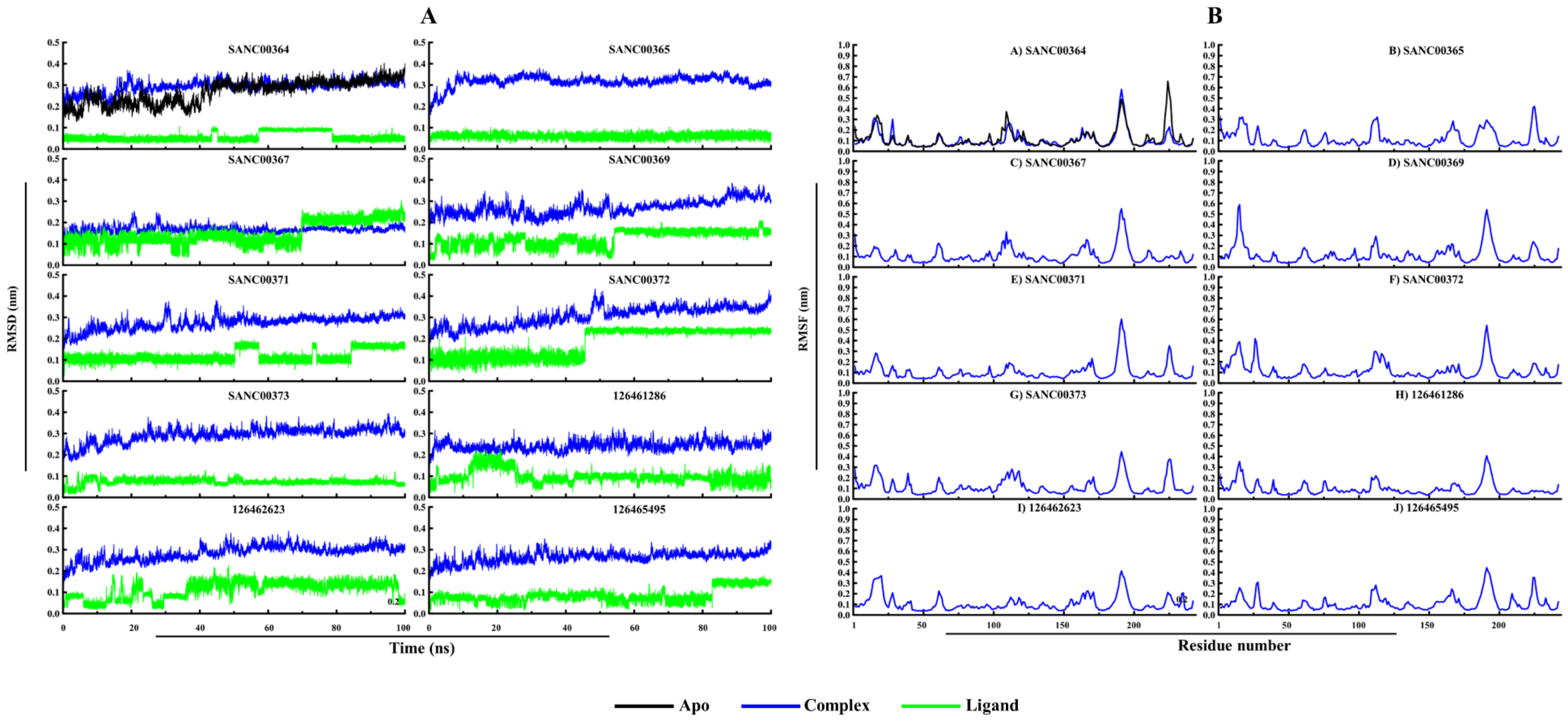

Figure S4: Time dependent evolution of RMSD (A) and RMSF (B) plots of VP-2 during 100 ns simulation. Color code: Black = Apo; Blue= Complex and Green = Ligand only.

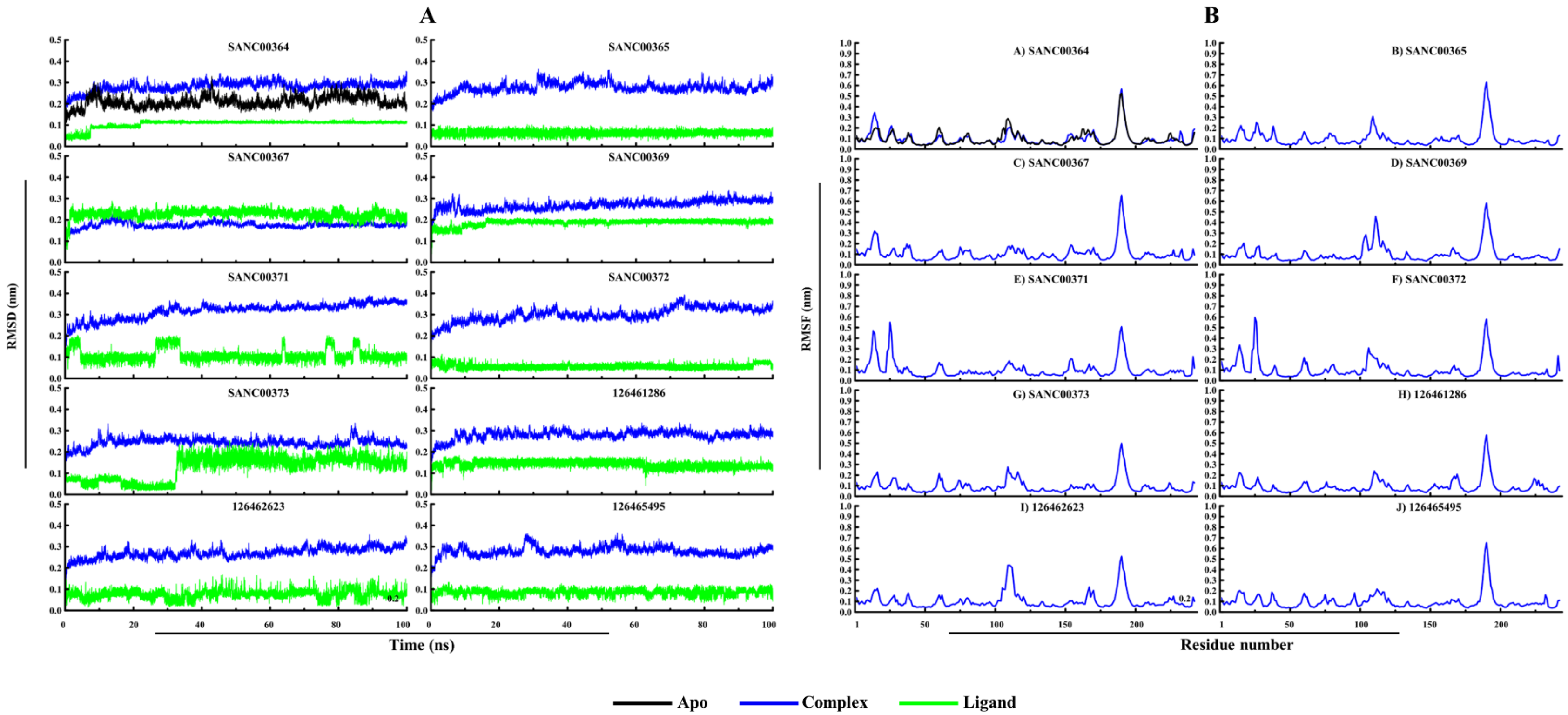

Figure S5: Time dependent evolution of RMSD (A) and RMSF (B) plots of VP-3 during 100 ns simulation. Color code: Black = Apo; Blue= Complex and Green = Ligand only.

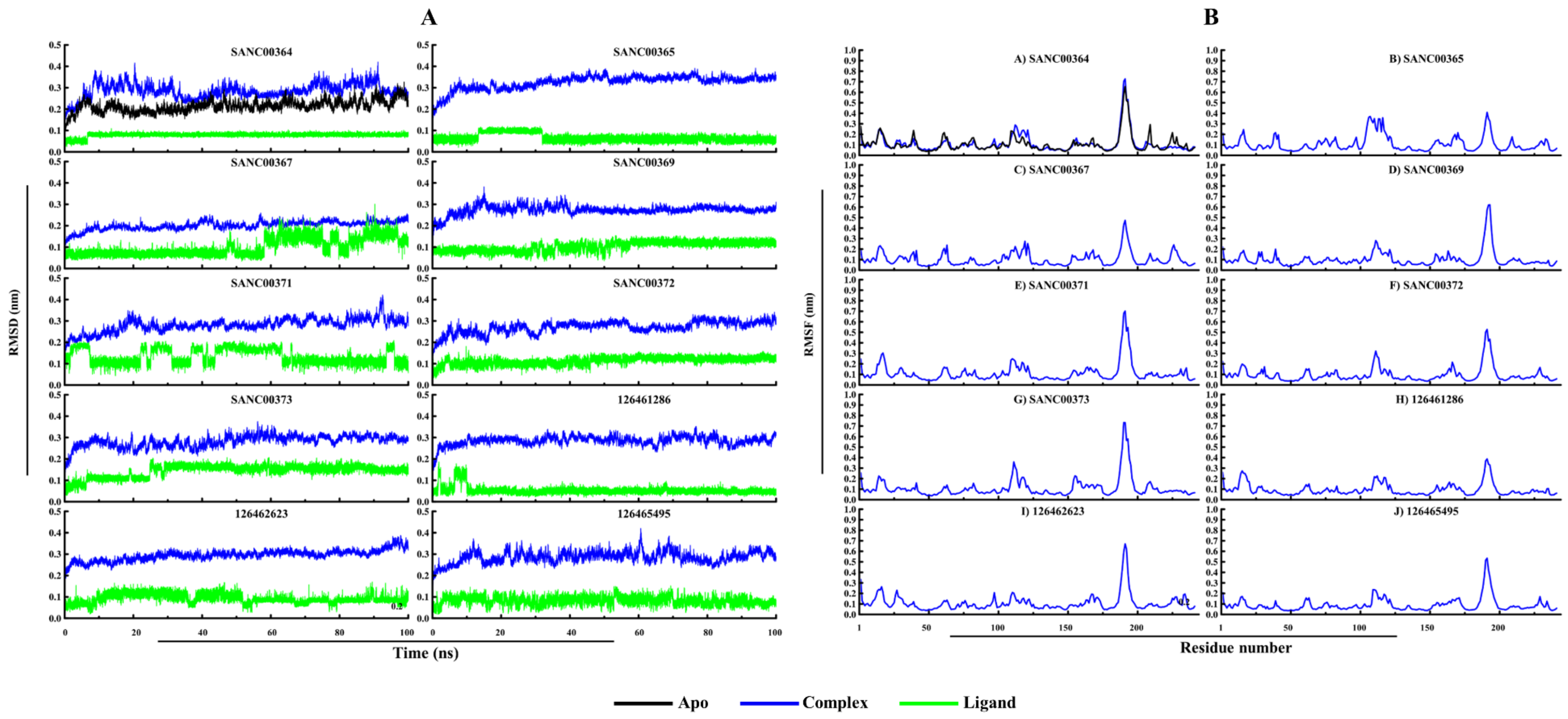

Figure S6: Time dependent evolution of RMSD (A) and RMSF (B) plots of KP-2 during 100 ns simulation. Color code: Black = Apo; Blue= Complex and Green = Ligand only.

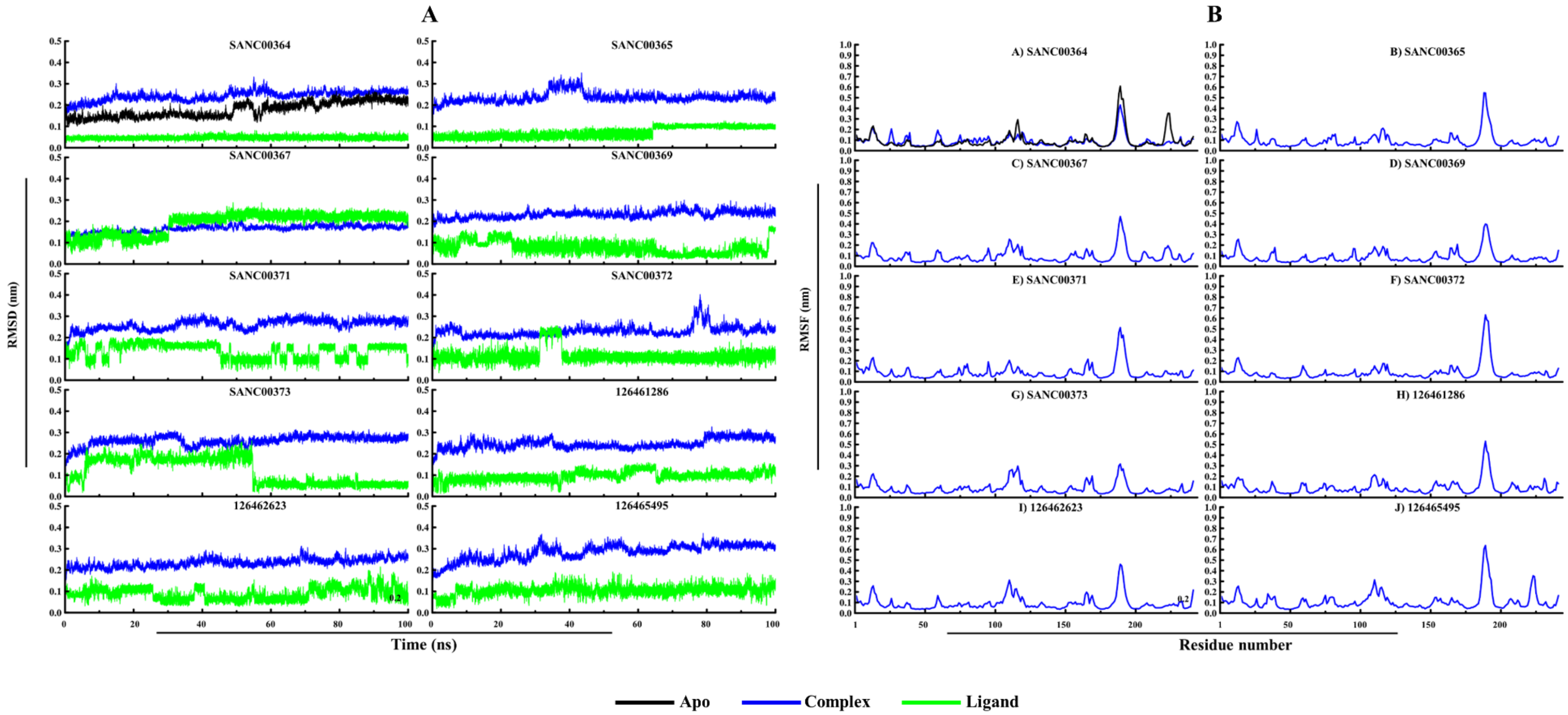

Figure S7: Time dependent evolution of RMSD (A) and RMSF (B) plots of KP-3 during 100 ns simulation. Color code: Black = Apo; Blue= Complex and Green = Ligand only.

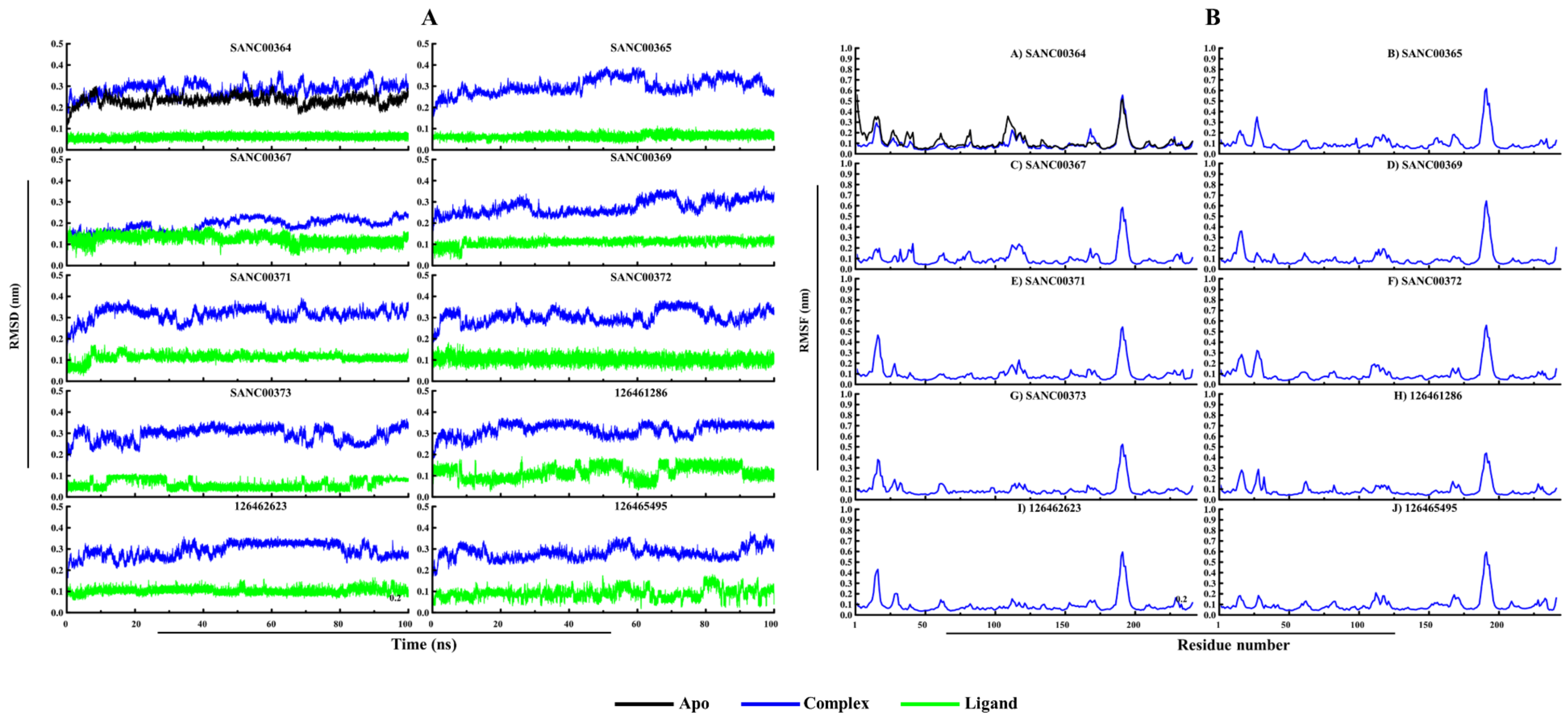

Figure S8: Time dependent evolution of RMSD (A) and RMSF (B) plots of BP-2 during 100 ns simulation. Color code: Black = Apo; Blue= Complex and Green = Ligand only.

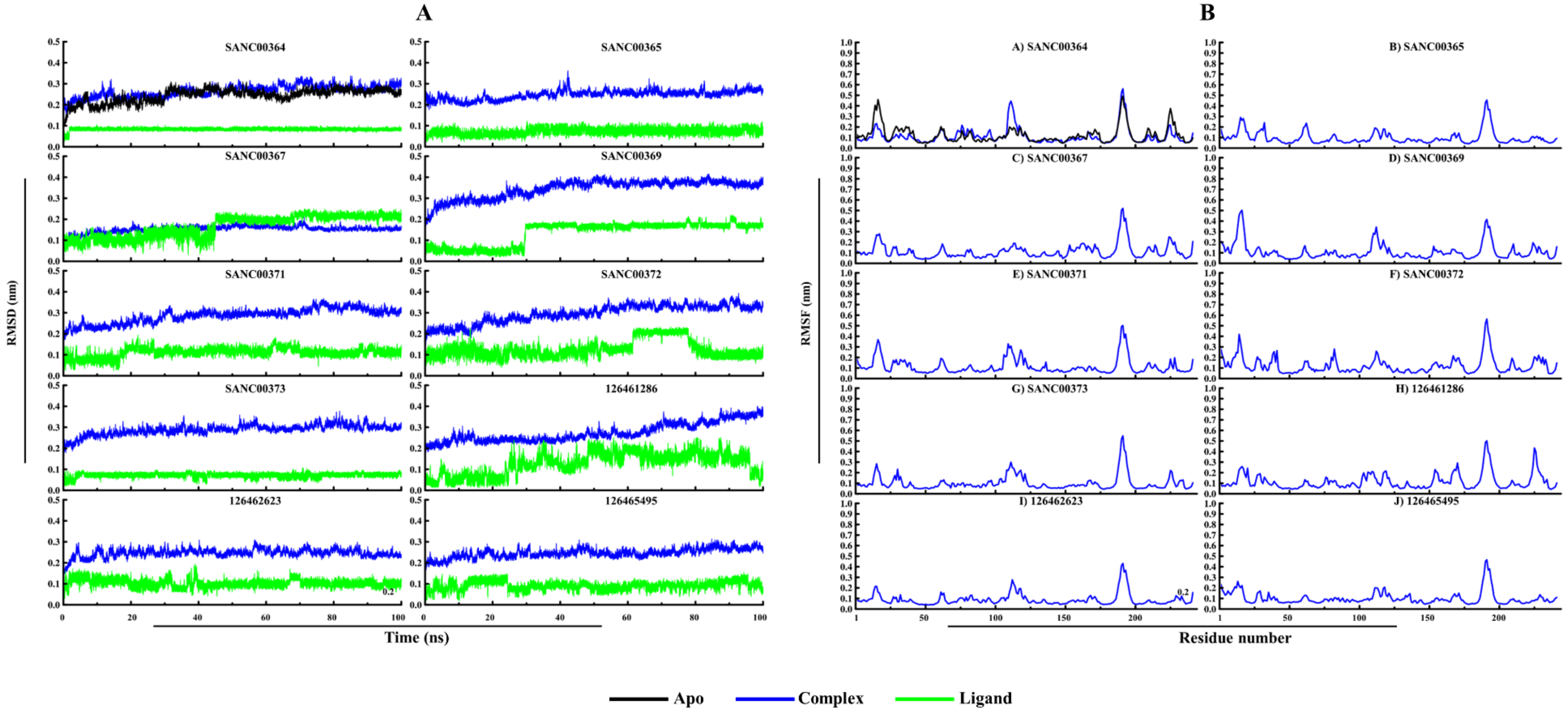

Figure S9: Time dependent evolution of RMSD (A) and RMSF (B) plots of CP-2 during 100 ns simulation. Color code: Black = Apo; Blue= Complex and Green = Ligand only.

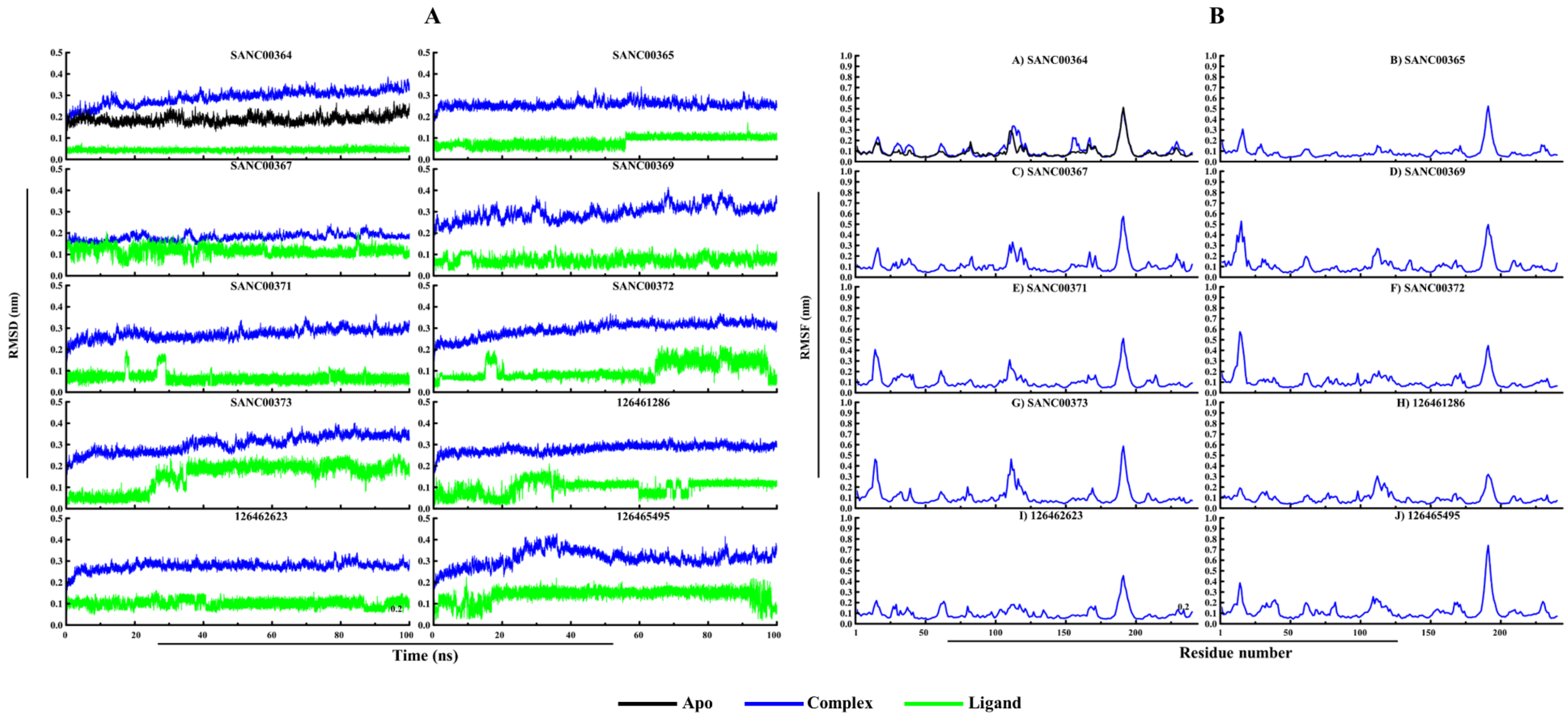

Figure S10: Time dependent evolution of RMSD (A) and RMSF (B) plots of YP-2 during 100 ns simulation. Color code: Black = Apo; Blue= Complex and Green = Ligand only.

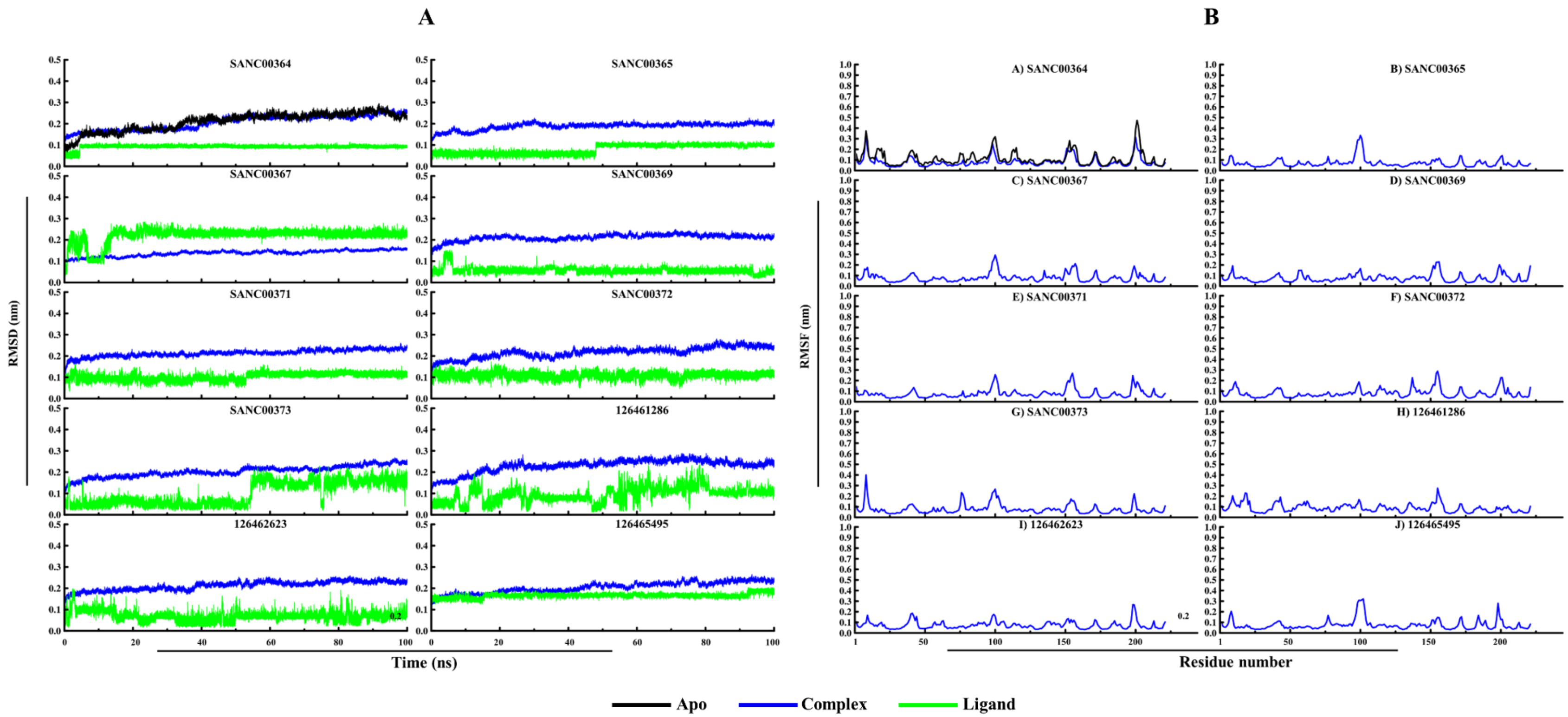

Figure S11: Time dependent evolution of RMSD (A) and RMSF (B) plots of Cat-K during 100 ns simulation. Color code: Black = Apo; Blue= Complex and Green = Ligand only.

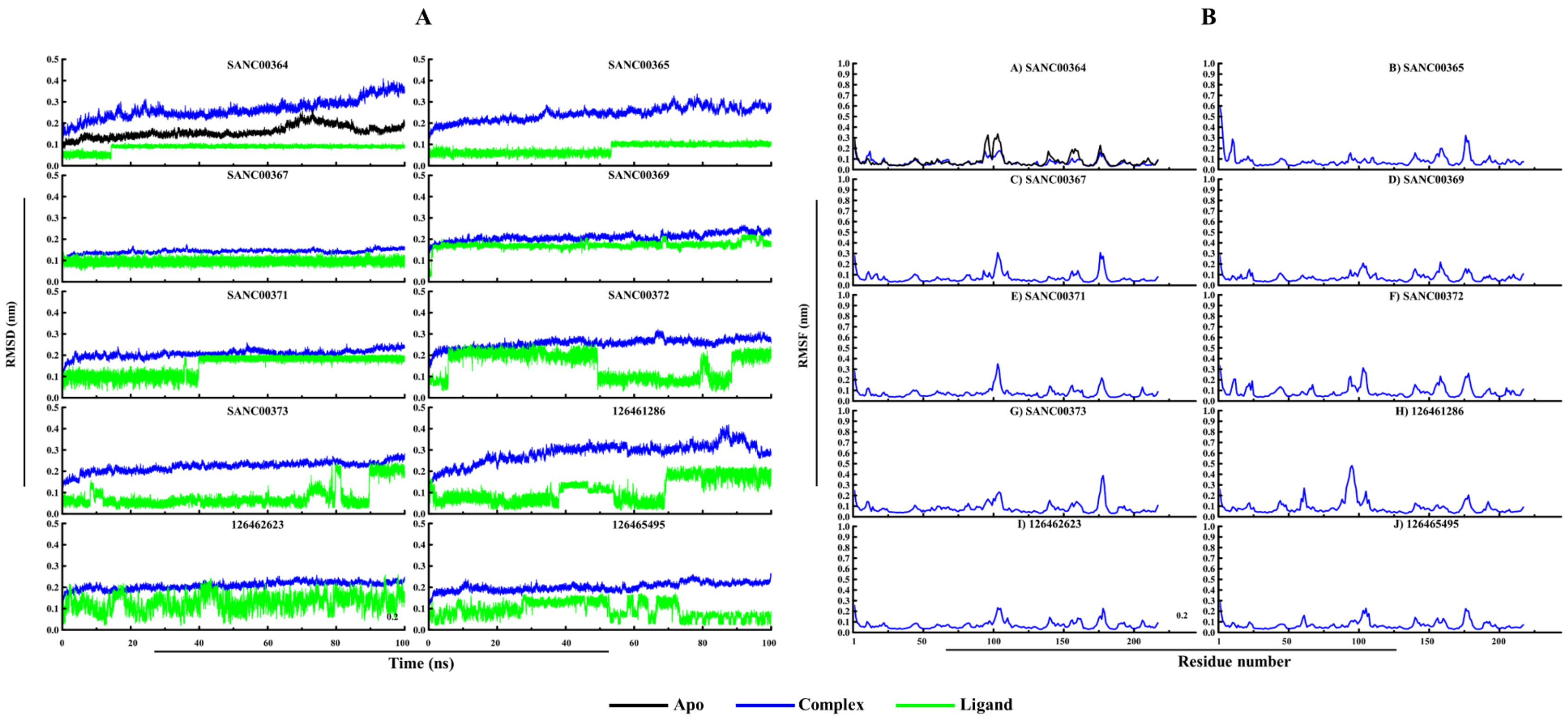

Figure S12: Time dependent evolution of RMSD (A) and RMSF (B) plots of Cat-L during 100 ns simulation. Color code: Black = Apo; Blue= Complex and Green = Ligand only.

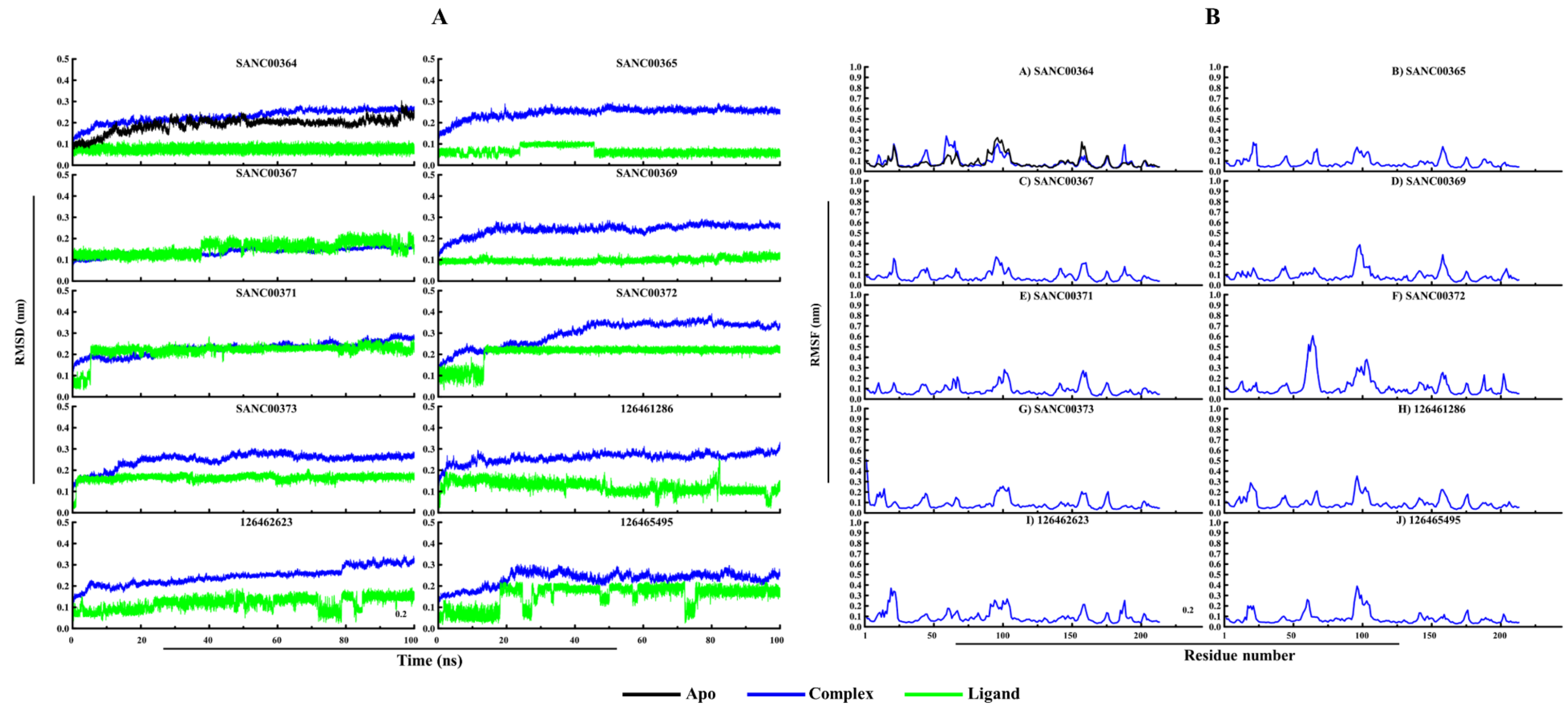

Figure S13: Time dependent evolution of RMSD (A) and RMSF (B) plots of Cat-S during 100 ns simulation. Color code: Black = Apo; Blue= Complex and Green = Ligand only.

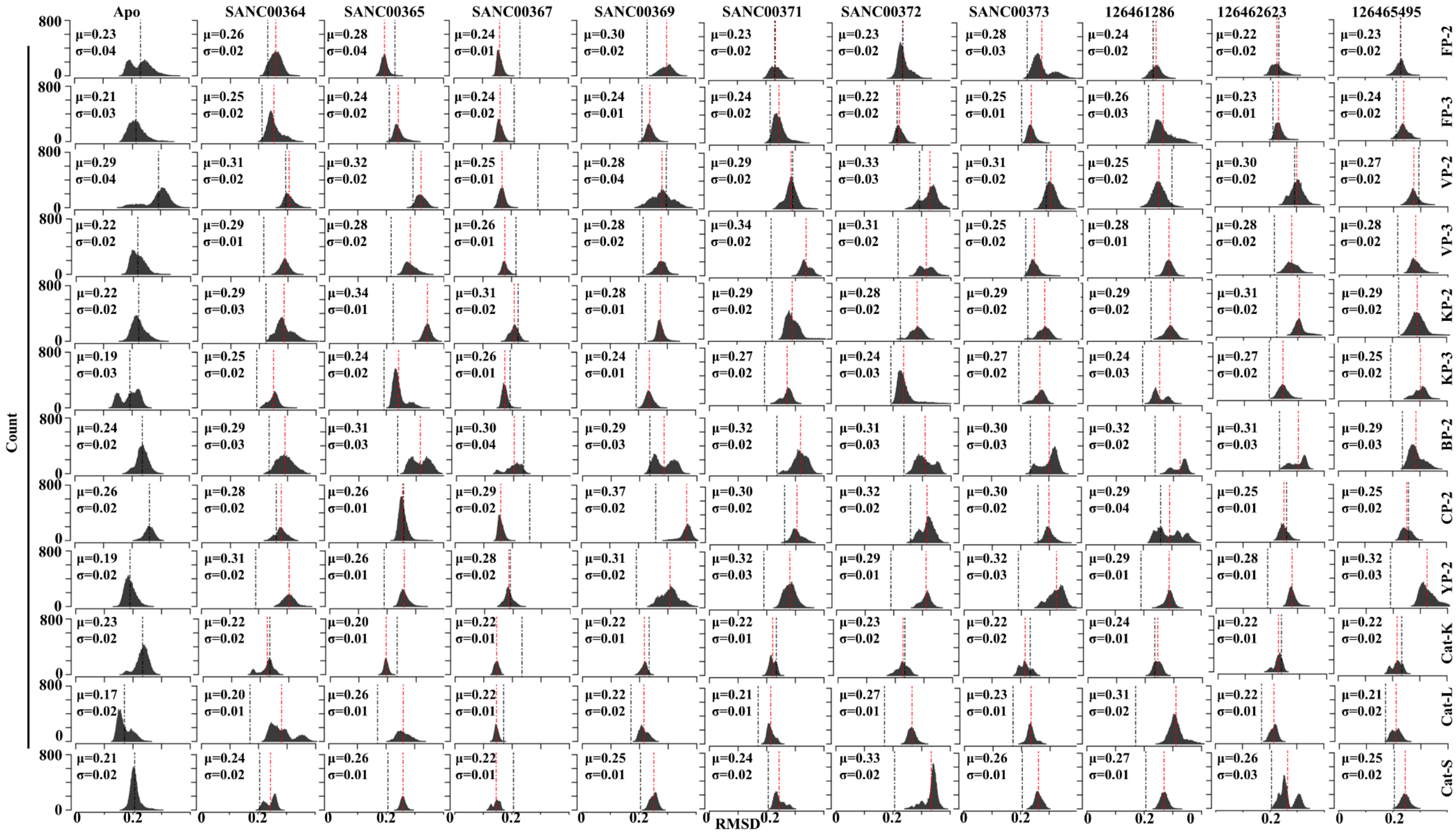

Figure S14. Cα protein backbone RMSD distribution histogram plots for apo and ligand bound protein systems. The effect of ligand binding on the global conformational variation for each protein can be determined by comparing the mean (μ) of the complex (dashed red line) and that of the corresponding apo system. σ denotes the conformational distribution standard deviation over the last 70 ns of simulation.

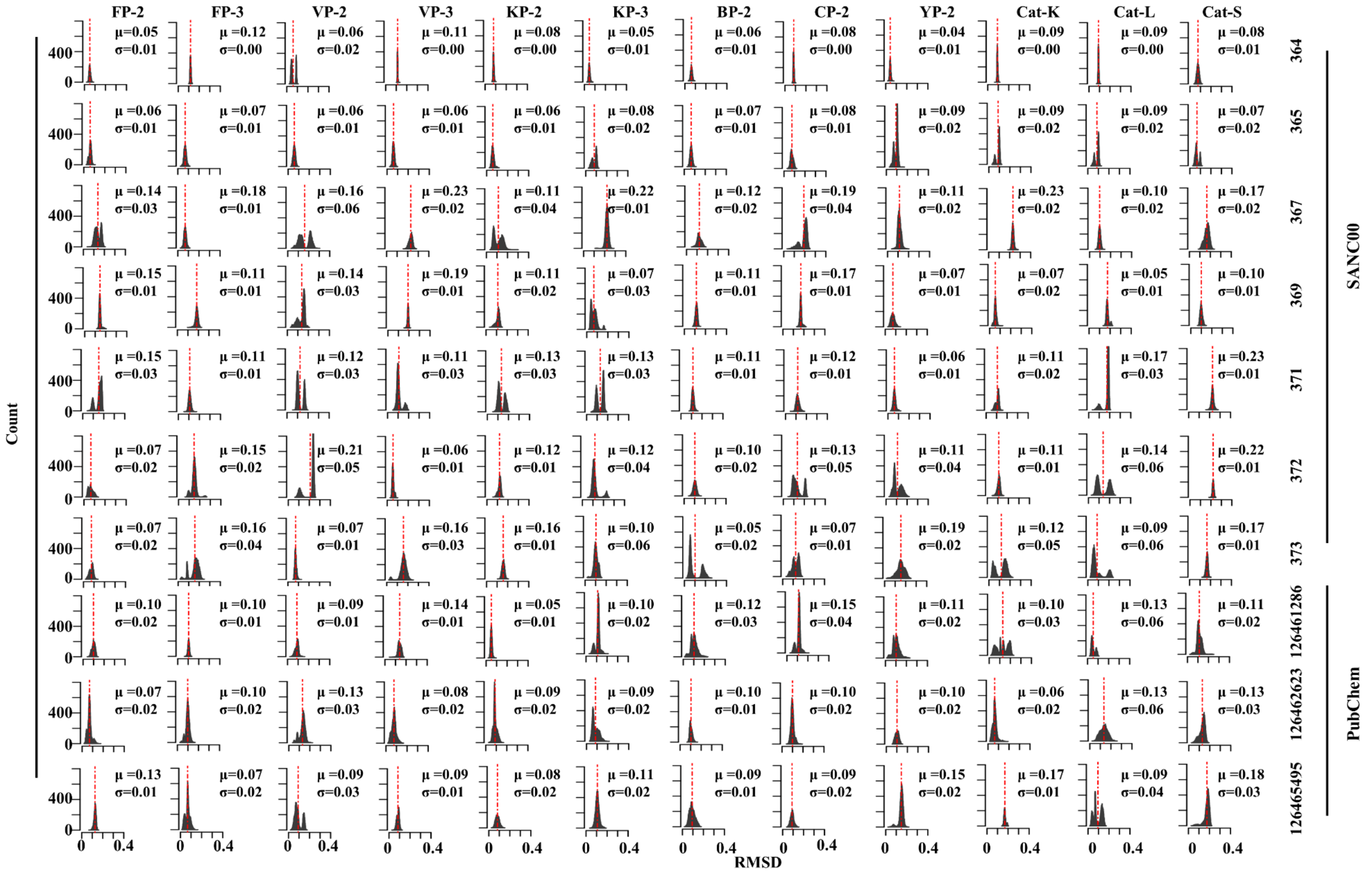

Figure S15. Ligand backbone RMSD distribution plots for the various ligands bound to plasmodial and human proteins. The dashed red line indicates the RMSD mean (μ) per each ligand while σ denotes the standard deviation during the last 70 ns of simulation.

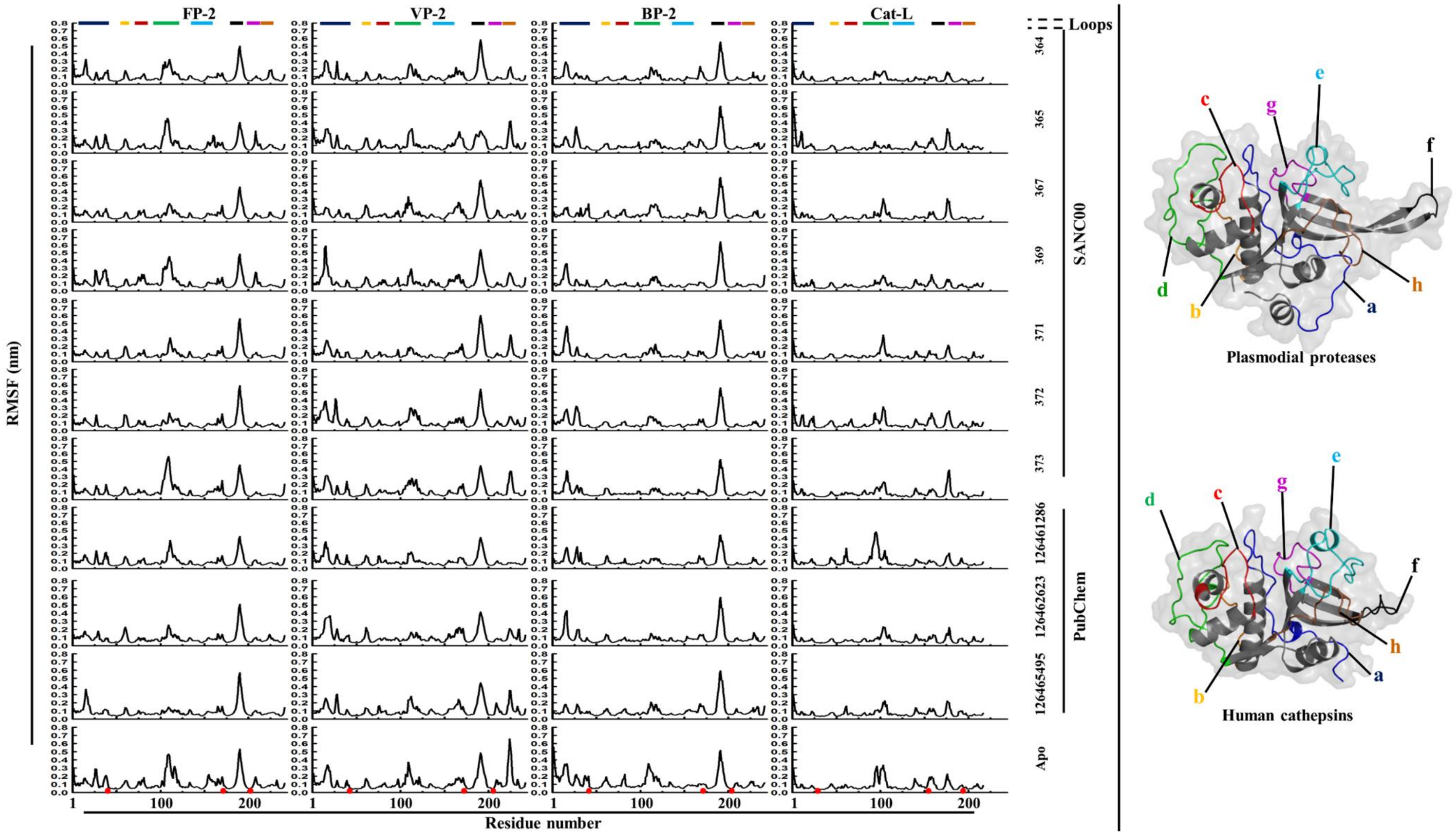

Figure S16. The effect of ligand binding on the fluctuations of each protein residue as determined by Root Mean Square Fluctuations (RMSF). Marked in red wedges are the catalytic triad (Cys-His-Asn) residues of each protein. The colour bars at the top correspond to the protein loops as shown on the right.

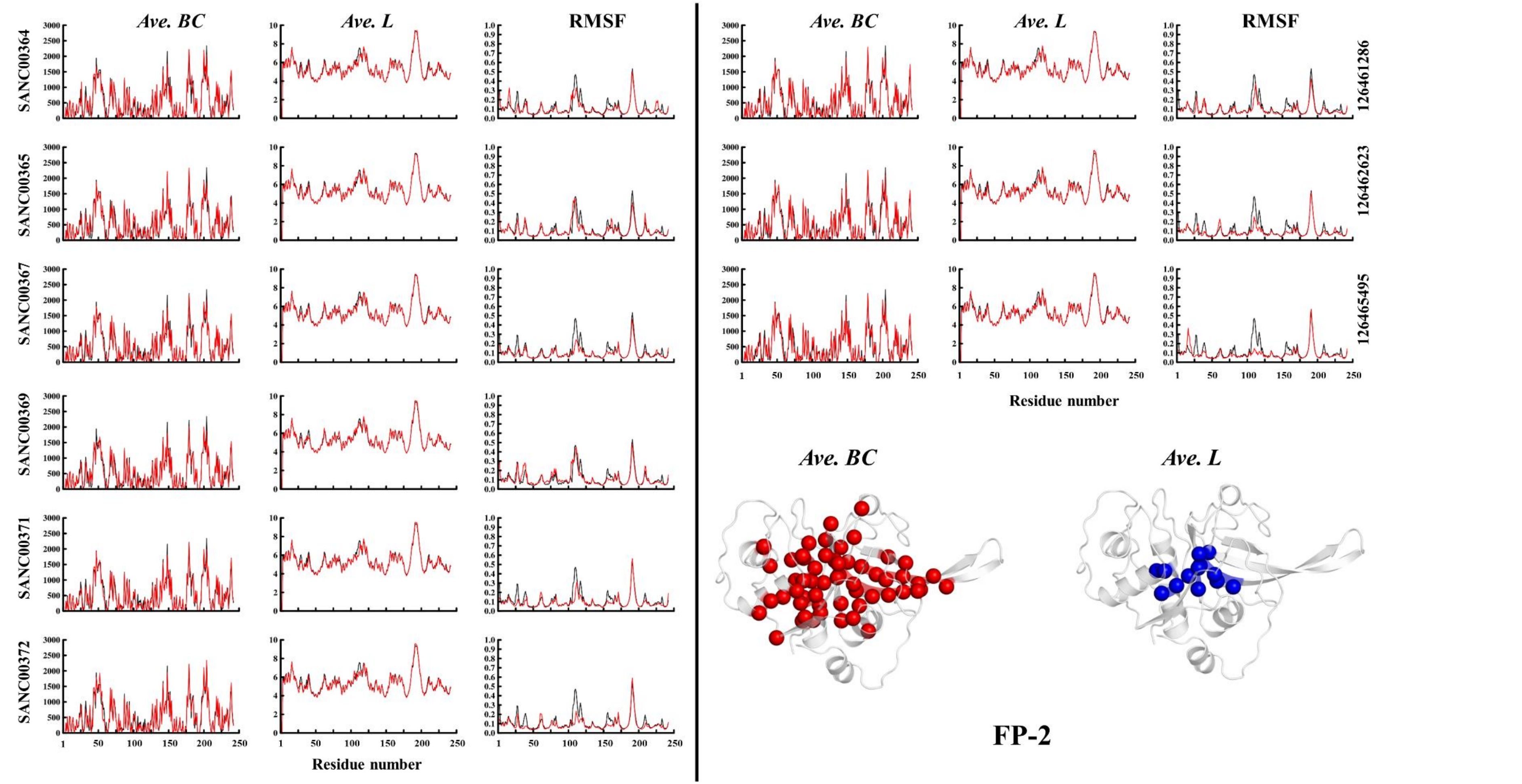

Figure S17: Dynamic residue network analysis (*betweenness of centrality* and *average shortest path*) of FP-2 in the presence of different ligands. Color code: Black = Apo; Red= Complex. The location of residues with significant high *average BC* and low *average L* score are shown in red and blue on the protein structure.

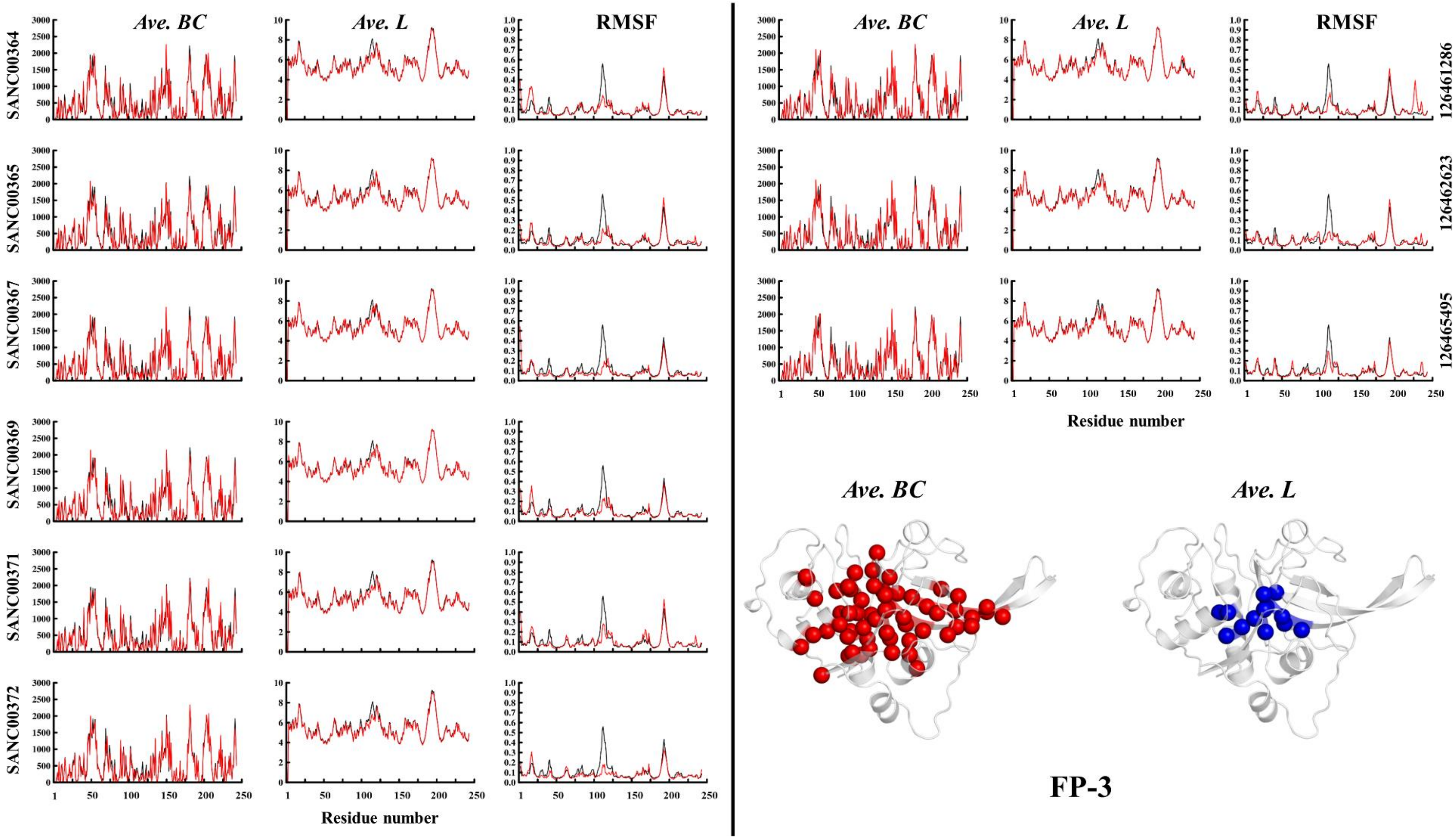

Figure S18: Dynamic residue network analysis (*betweenness of centrality* and *average shortest path*) of FP-3 in the presence of different ligands. Color code: Black = Apo; Red= Complex. The location of residues with significant high *average BC* and low *average L* score are shown in red and blue on the protein structure.

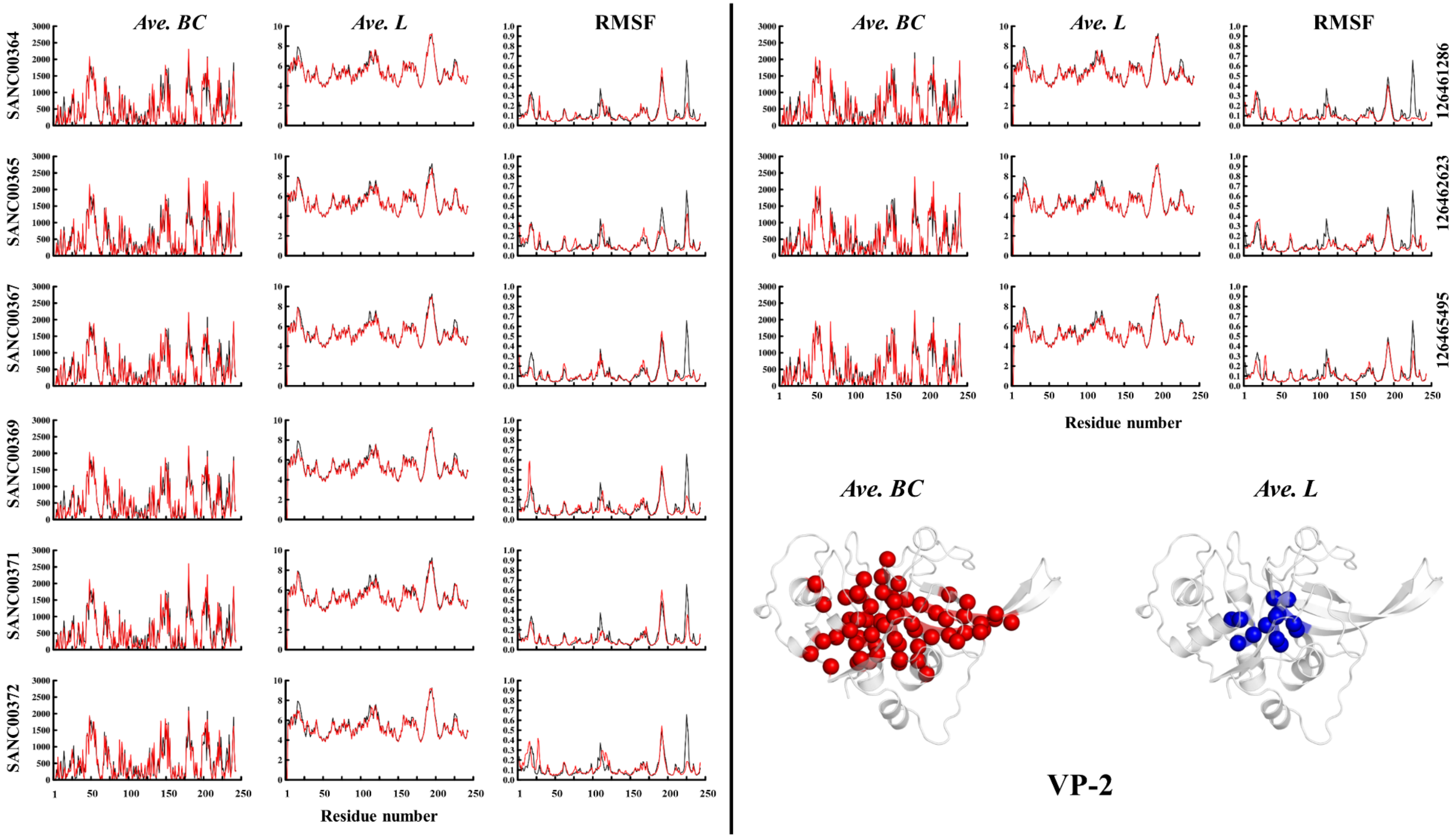

Figure S19: Dynamic residue network analysis (*betweenness of centrality* and *average shortest path*) of VP-2 in the presence of different ligands. Color code: Black = Apo; Red= Complex. The location of residues with significant high *average BC* and low *average L* score are shown in red and blue on the protein structure.

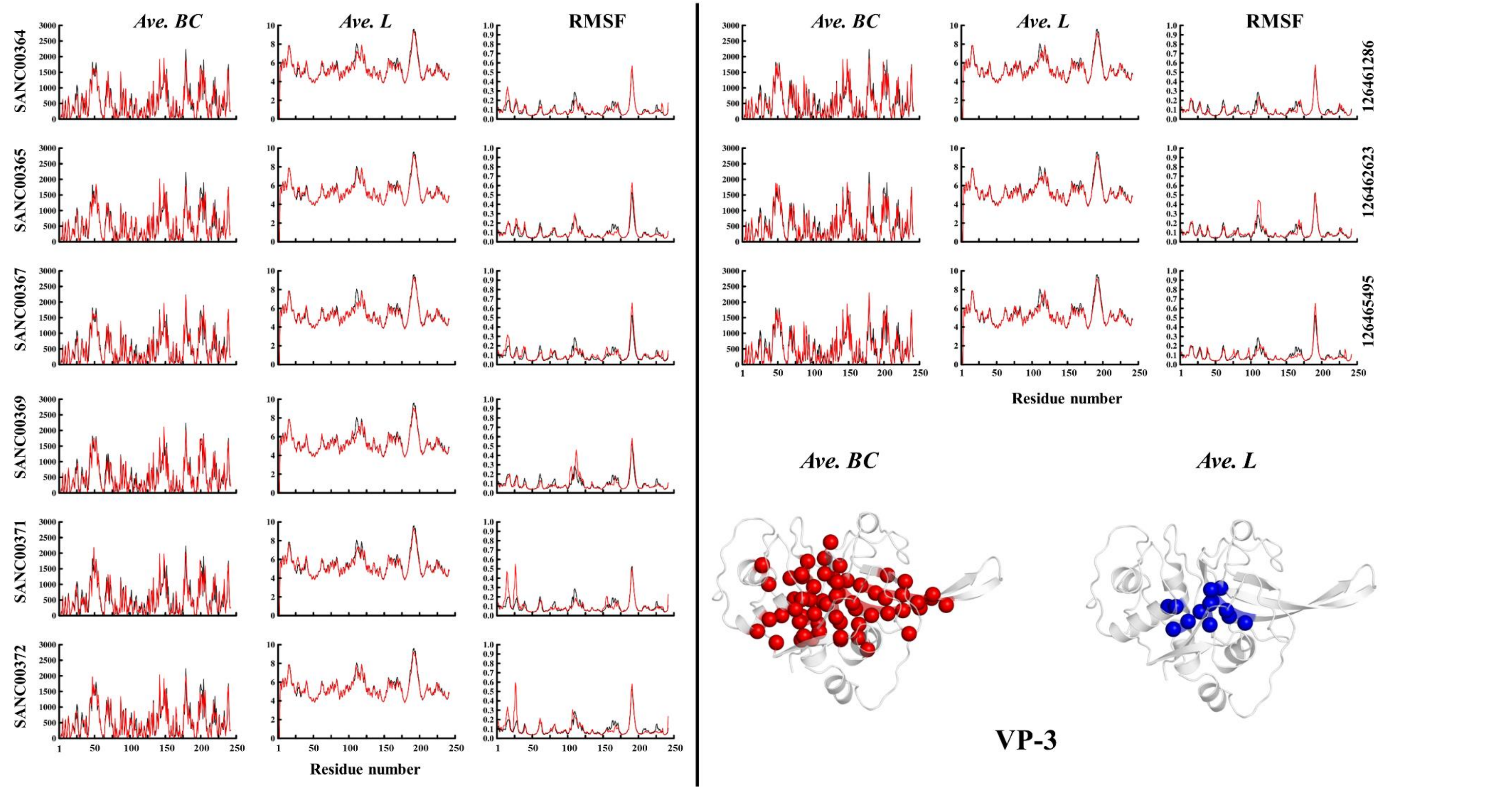

Figure S20: Dynamic residue network analysis (*betweenness of centrality* and *average shortest path*) of VP-3 in the presence of different ligands. Color code: Black = Apo; Red= Complex. The location of residues with significant high *average BC* and low *average L* score are shown in red and blue on the protein structure.

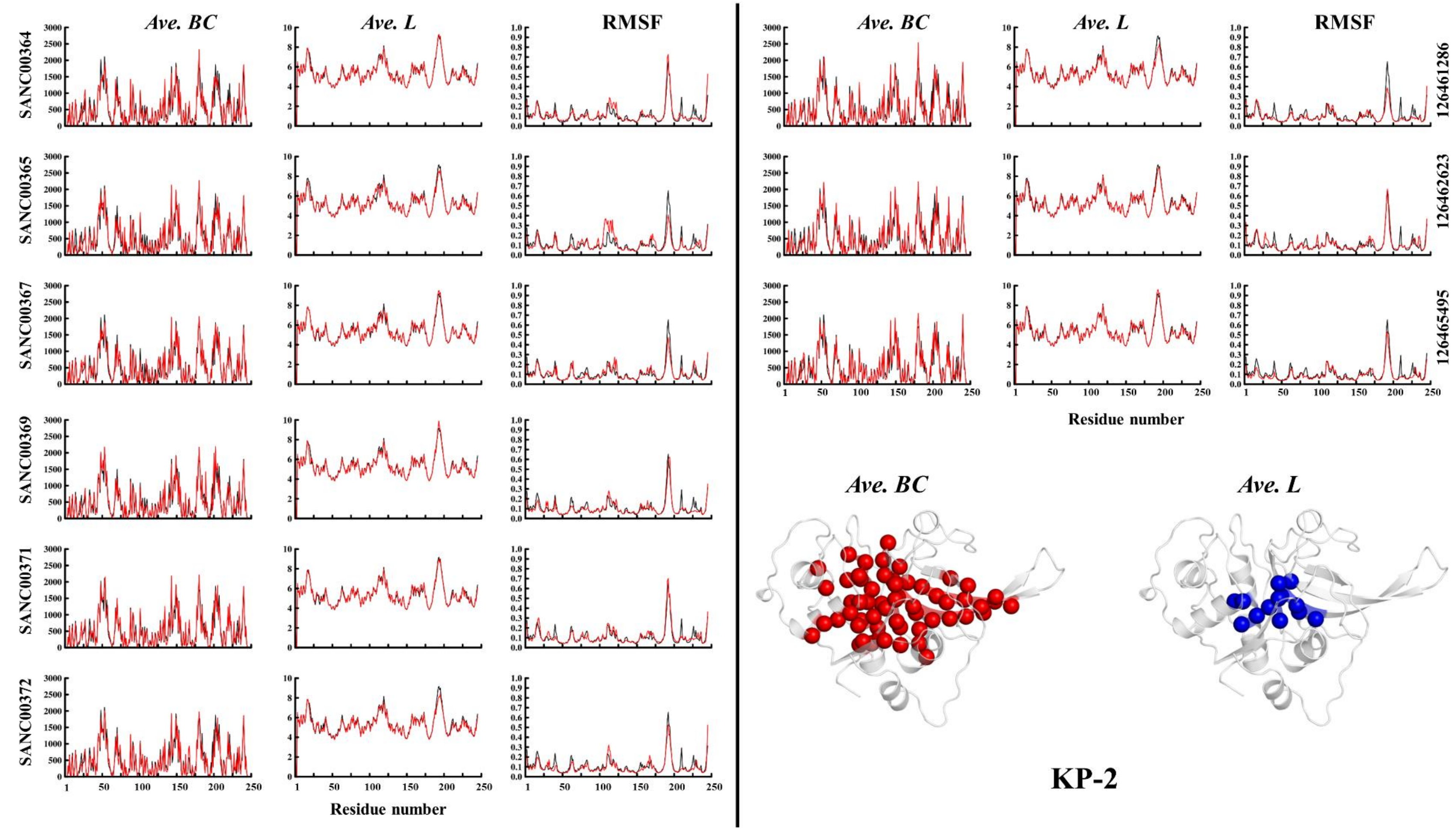

Figure S21: Dynamic residue network analysis (*betweenness of centrality* and *average shortest path*) of KP-2 in the presence of different ligands. Color code: Black = Apo; Red= Complex. The location of residues with significant high *average BC* and low *average L* score are shown in red and blue on the protein structure.

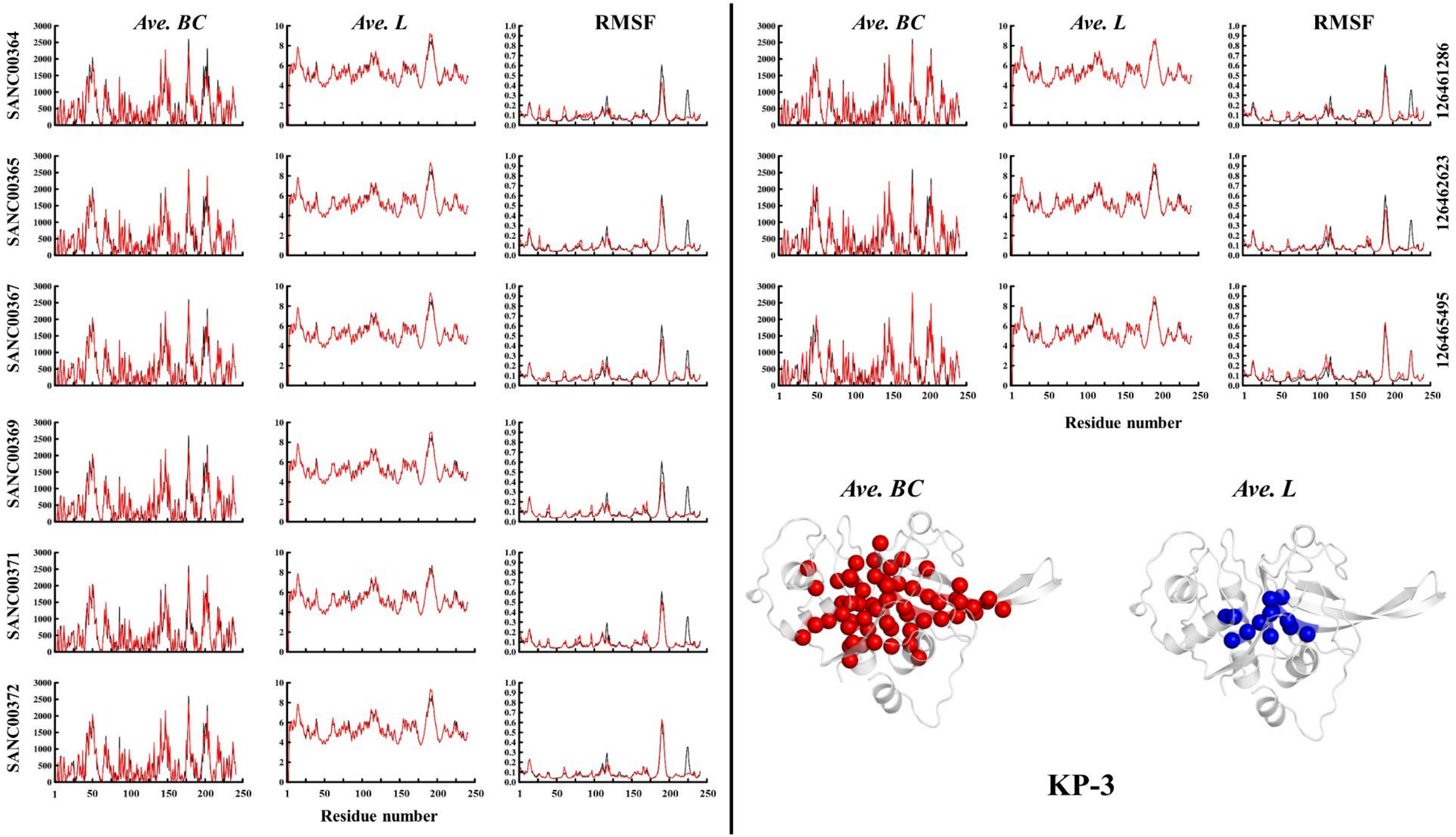

Figure S22: Dynamic residue network analysis (*betweenness of centrality* and *average shortest path*) of KP-3 in the presence of different ligands. Color code: Black = Apo; Red= Complex. The location of residues with significant high *average BC* and low *average L* score are shown in red and blue on the protein structure.

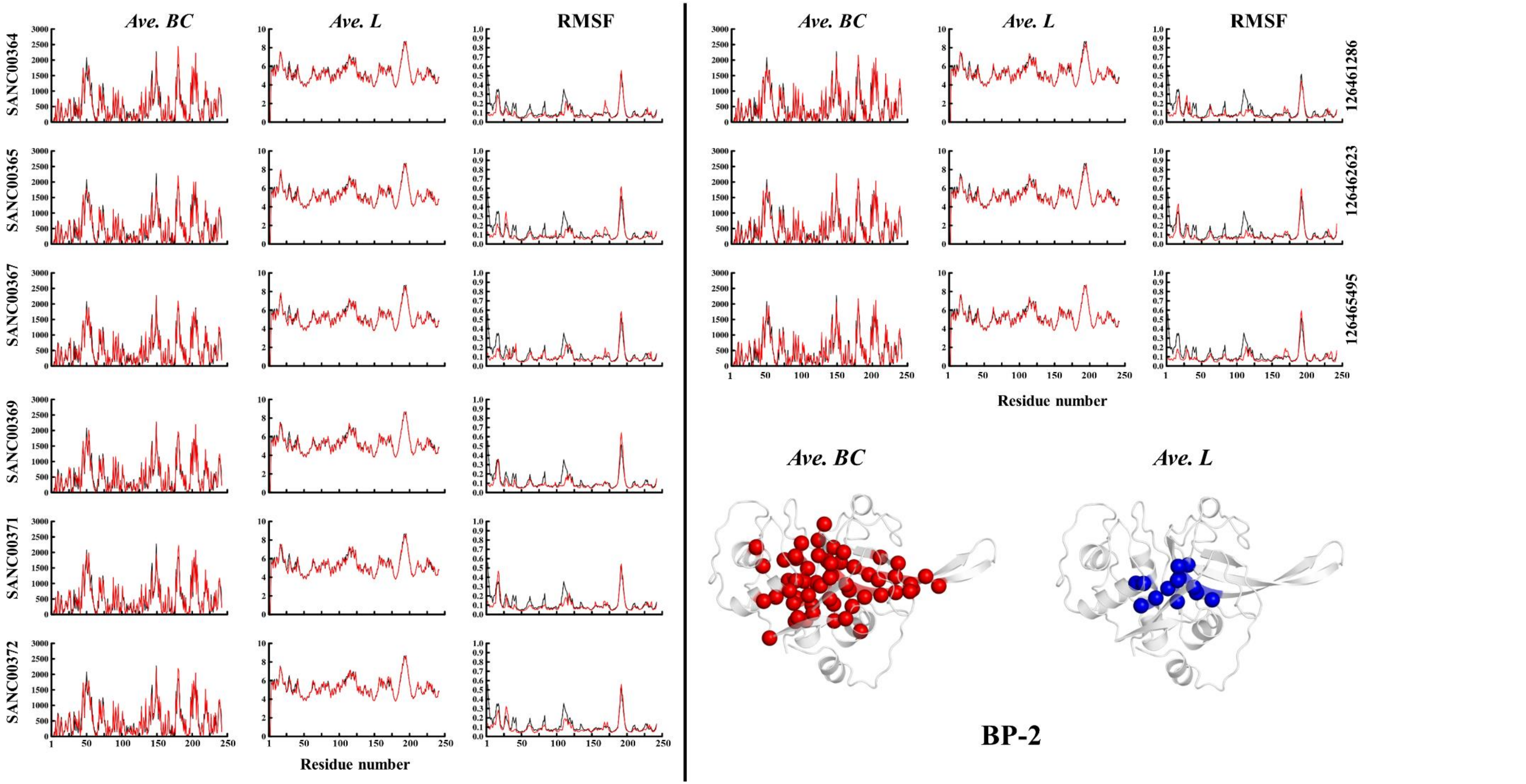

Figure S23: Dynamic residue network analysis (*betweenness of centrality* and *average shortest path*) of BP-2 in the presence of different ligands. Color code: Black = Apo; Red= Complex. The location of residues with significant high *average BC* and low *average L* score are shown in red and blue on the protein structure.

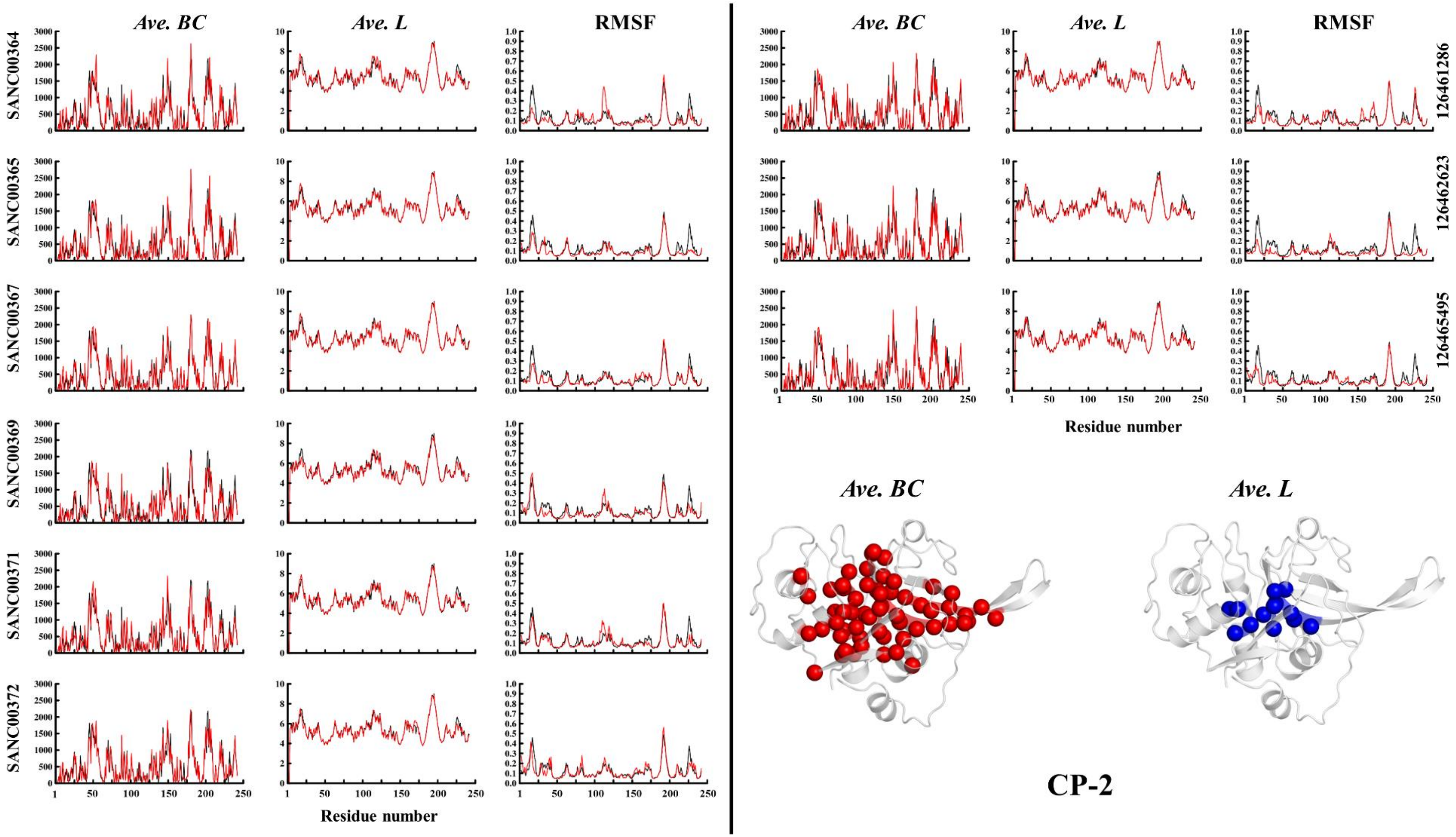

Figure S24: Dynamic residue network analysis (*betweenness of centrality* and *average shortest path*) of CP-2 in the presence of different ligands. Color code: Black = Apo; Red= Complex. The location of residues with significant high *average BC* and low *average L* score are shown in red and blue on the protein structure.

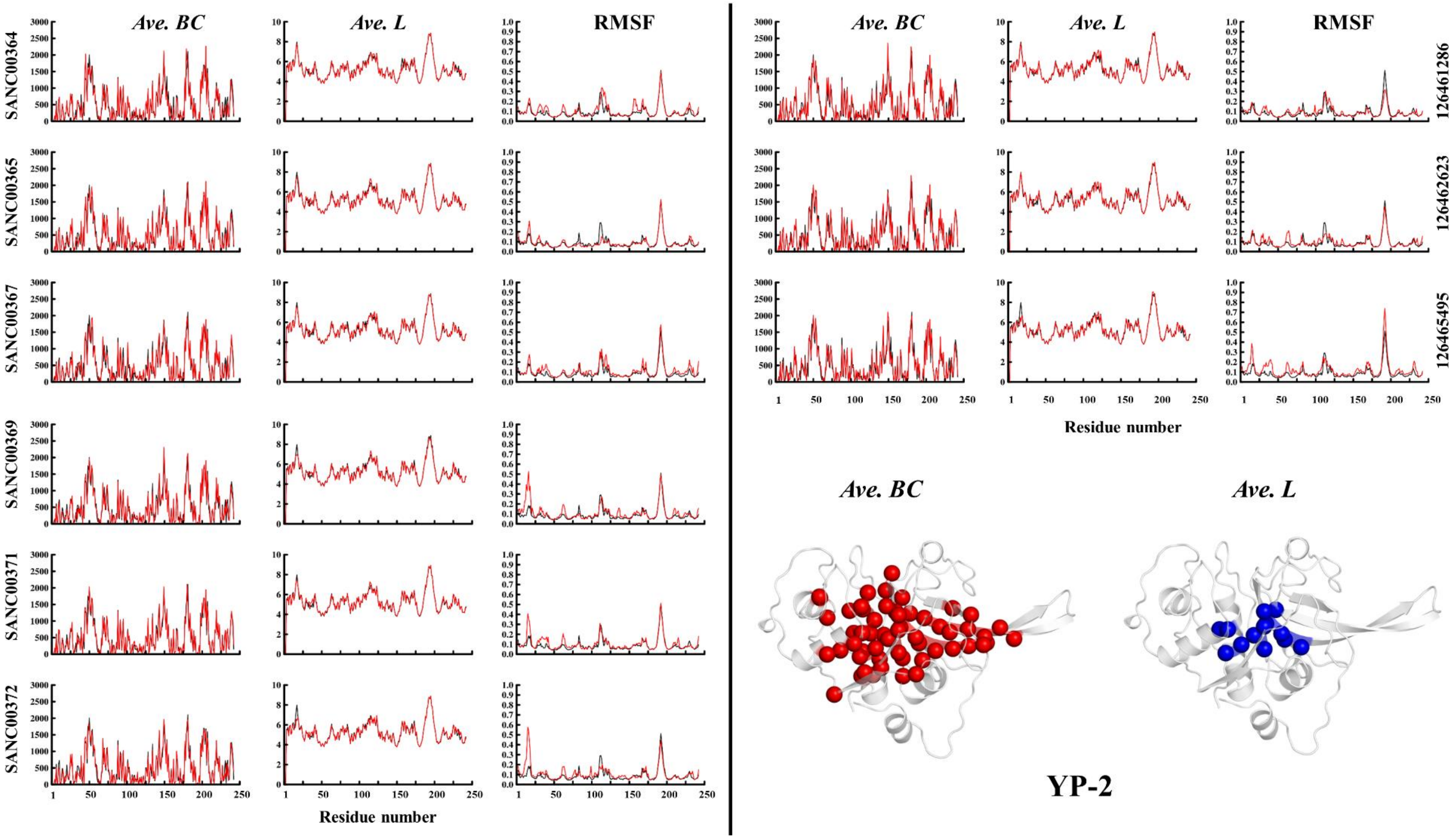

Figure S25: Dynamic residue network analysis (*betweenness of centrality* and *average shortest path*) of YP-2 in the presence of different ligands. Color code: Black = Apo; Red= Complex. The location of residues with significant high *average BC* and low *average L* score are shown in red and blue on the protein structure.

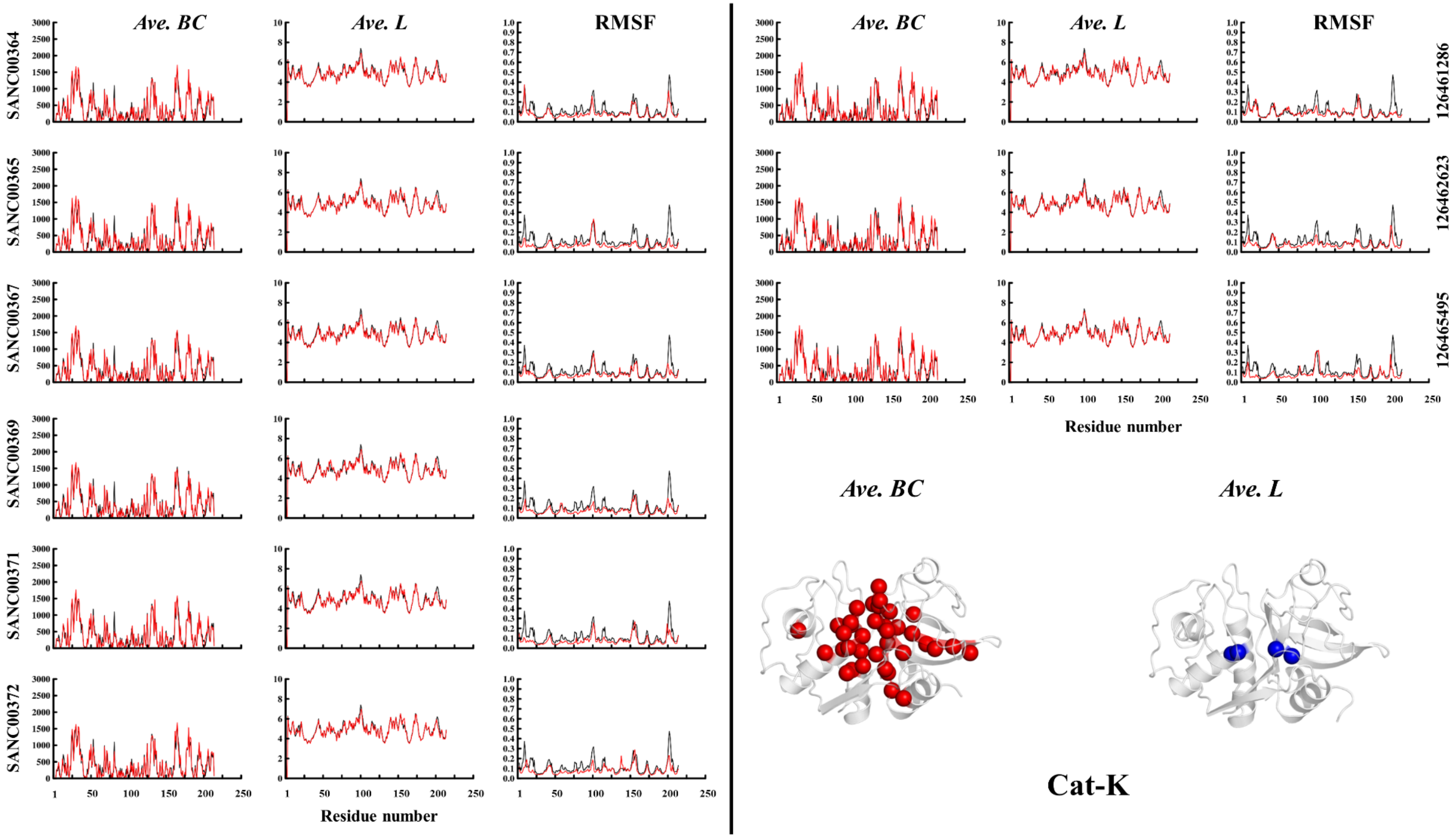

Figure S26: Dynamic residue network analysis (*betweenness of centrality* and *average shortest path*) of Cat-K in the presence of different ligands. Color code: Black = Apo; Red= Complex. The location of residues with significant high *average BC* and low *average L* score are shown in red and blue on the protein structure.

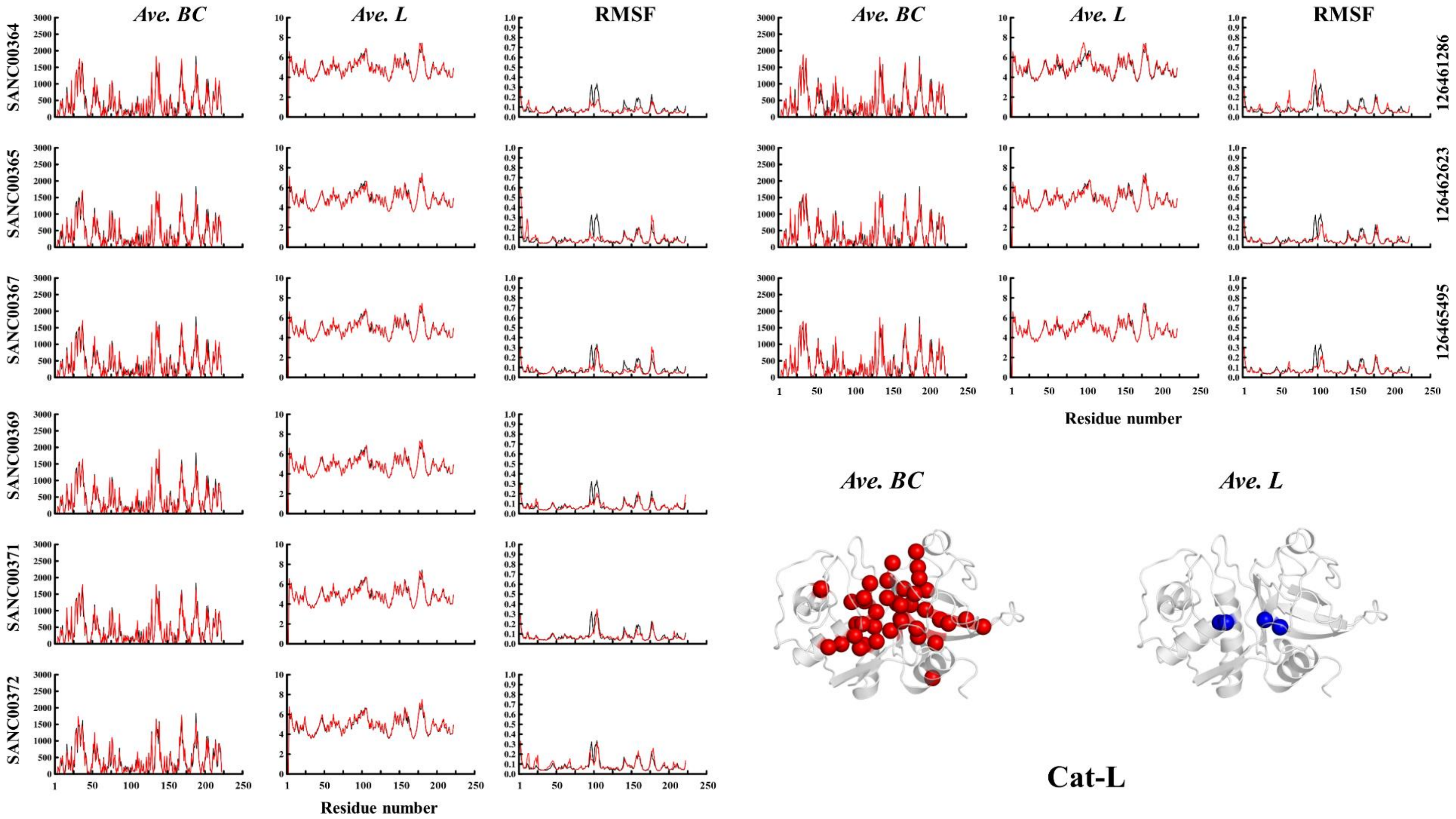

306

307 **Figure S27:** Dynamic residue network analysis (*betweenness of centrality* and *average shortest path*) of Cat-L in the presence of different
308 ligands. Color code: Black = Apo; Red= Complex. The location of residues with significant high *average BC* and low *average L* score are shown
309 in red and blue on the protein structure.

310

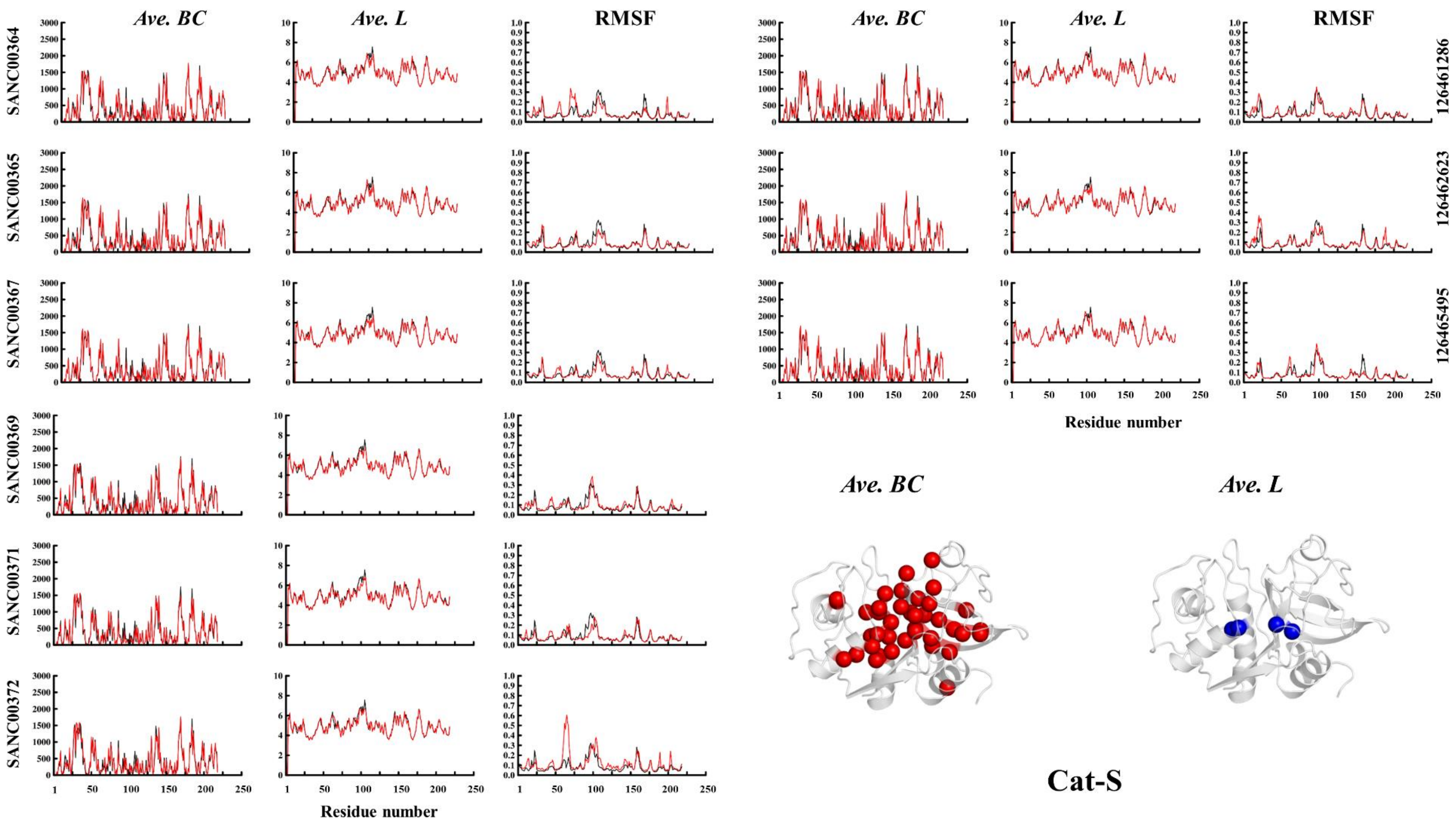

Figure S28: Dynamic residue network analysis (*betweenness of centrality* and *average shortest path*) of Cat-S in the presence of different ligands. Color code: Black = Apo; Red= Complex. The location of residues with significant high *average BC* and low *average L* score are shown in red and blue on the protein structure.

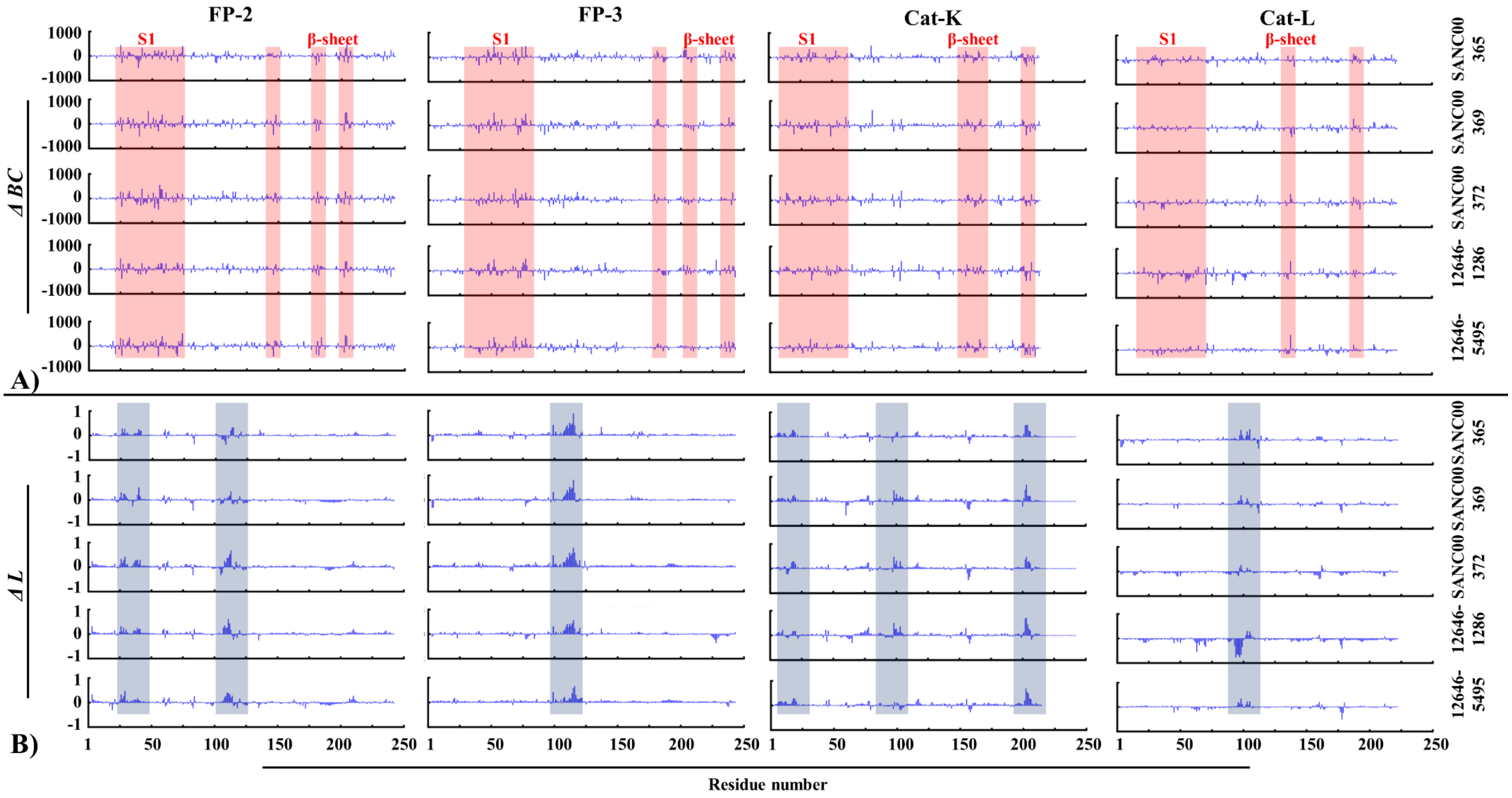

Figure S29. Effect of ligand binding on *BC* (A) and *L* (B) using the apo structures as the reference. Shaded are regions of the protein that showed significant changes (one and half times away from the means of the ligand bound systems) in *BC* (red) and *L* (blue).

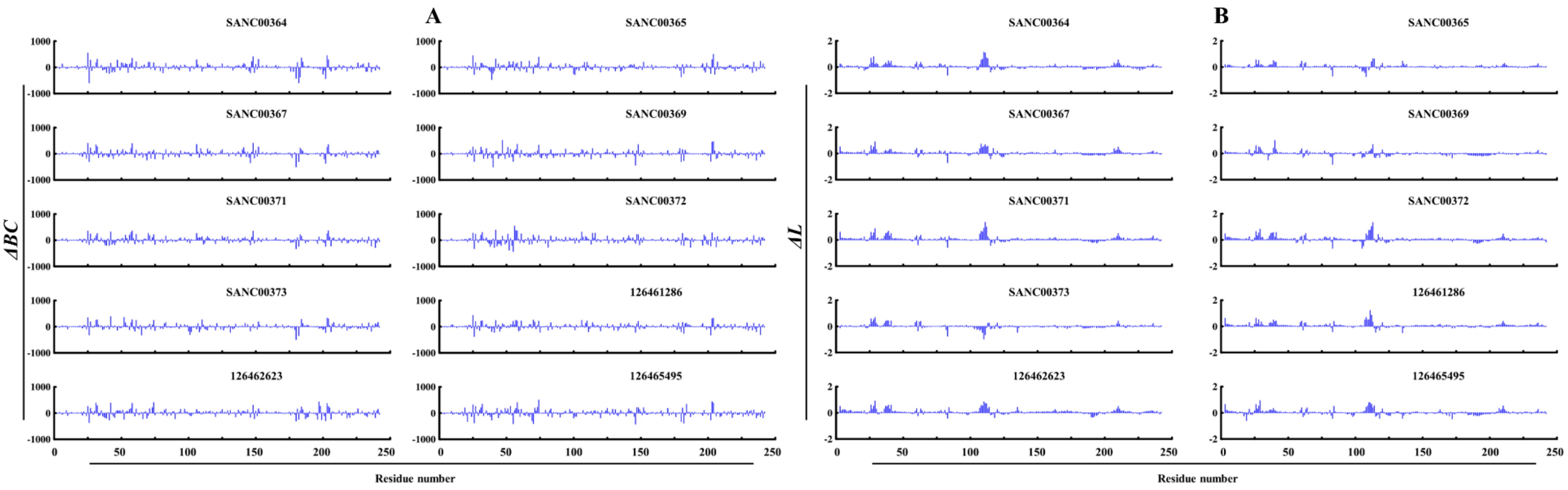

Figure S30: Plots showing FP-2 residues with significant changes in *average* a) *BC* and B) *L* upon ligand binding.

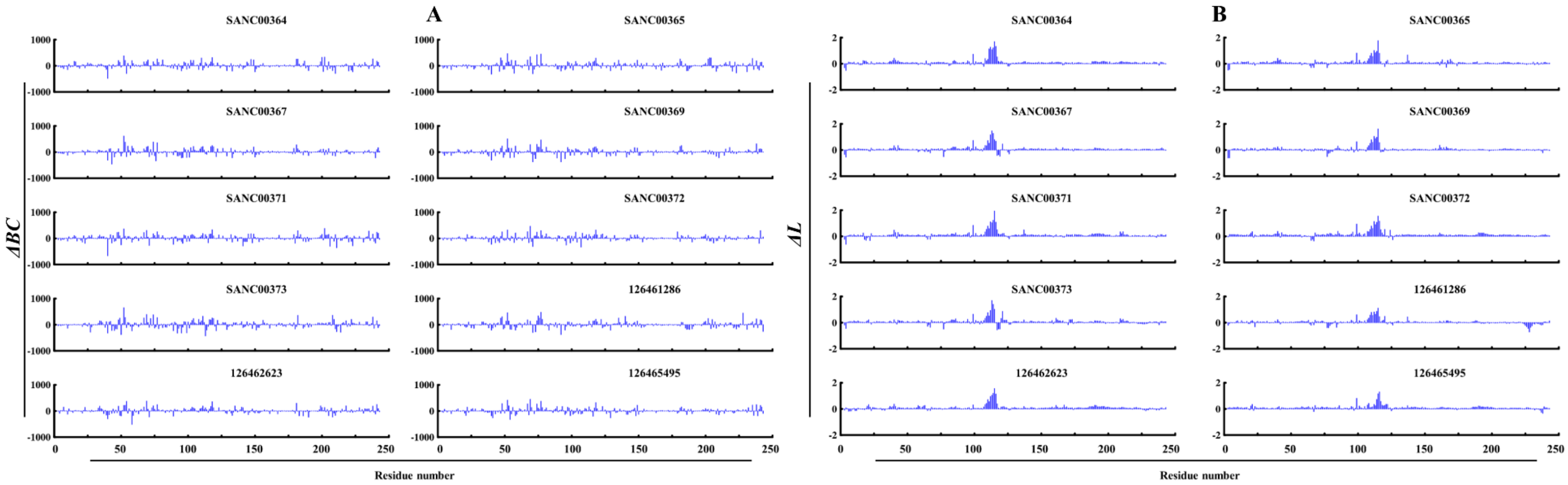

Figure S31: Plots showing FP-3 residues with significant changes in *average* a) *BC* and B) *L* upon ligand binding.

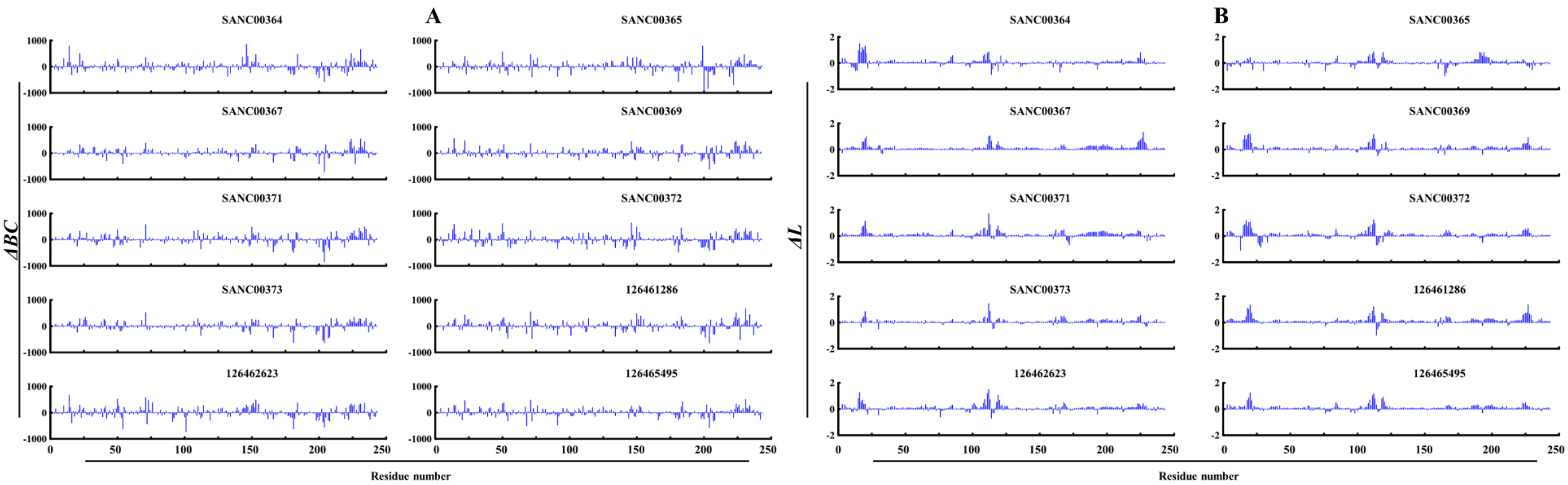

Figure S32: Plots showing VP-2 residues with significant changes in *average* a) *BC* and B) *L* upon ligand binding.

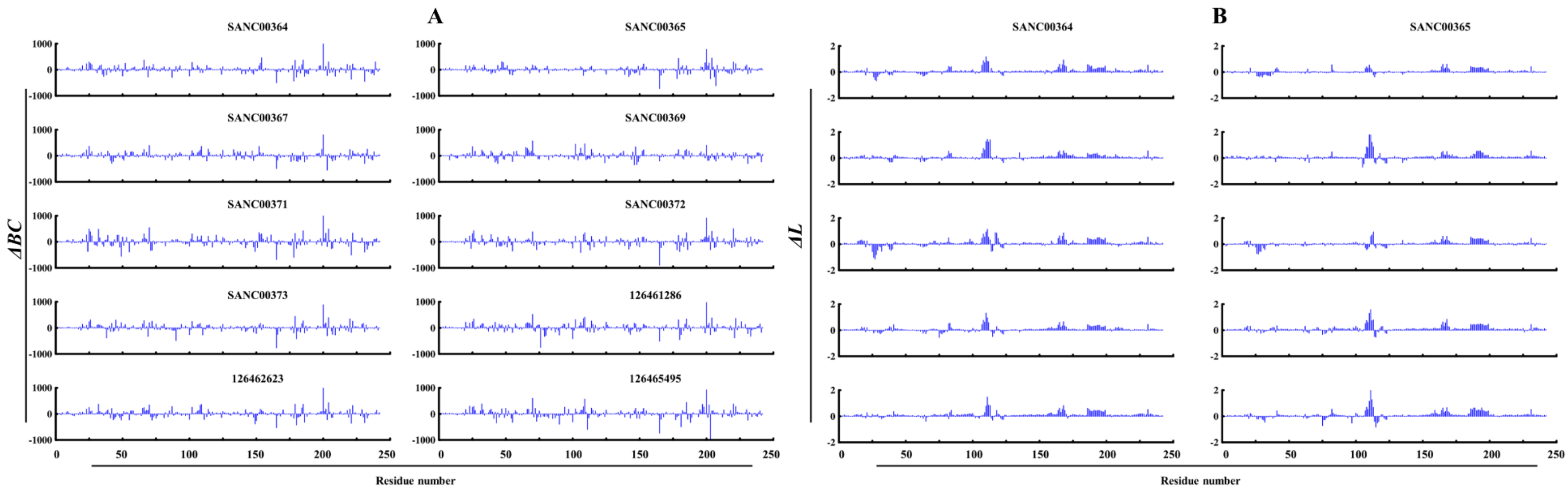

Figure S33: Plots showing VP-3 residues with significant changes in *average* a) *BC* and B) *L* upon ligand binding.

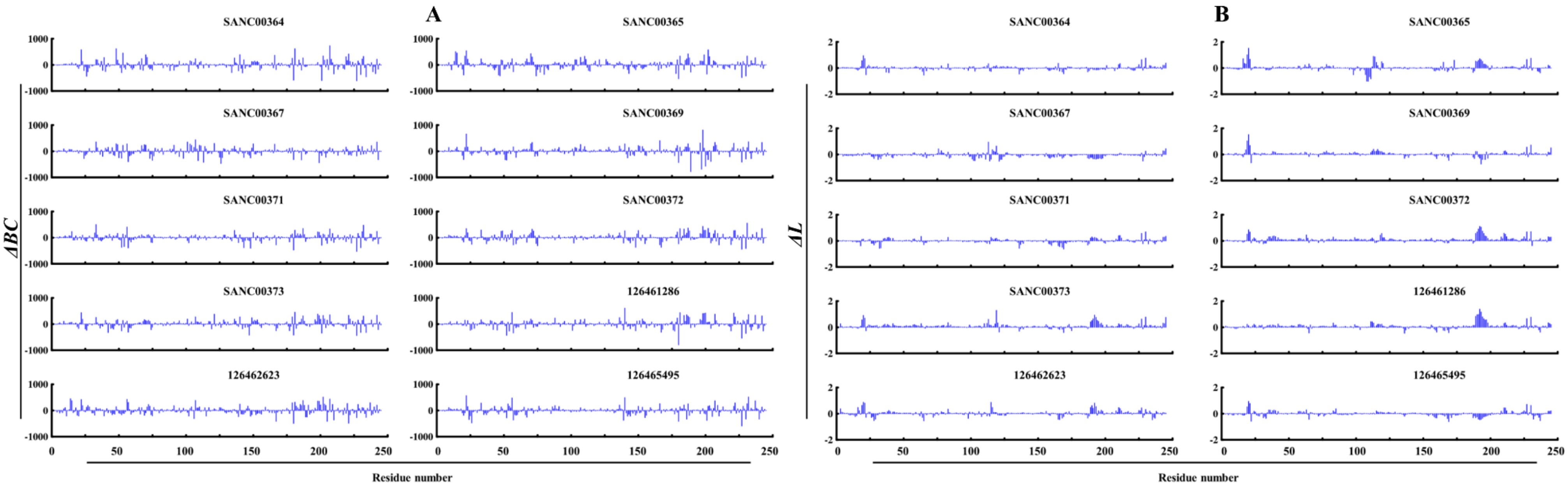

Figure S34: Plots showing KP-2 residues with significant changes in *average* a) *BC* and B) *L* upon ligand binding.

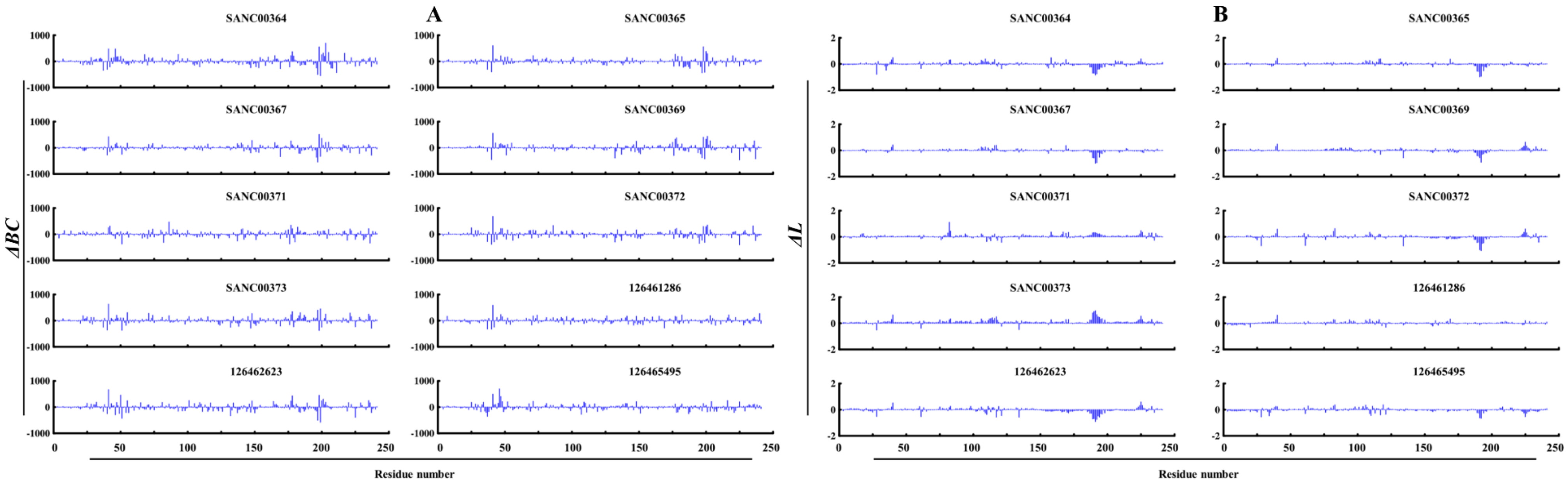

Figure S35: Plots showing KP-3 residues with significant changes in *average* a) *BC* and B) *L* upon ligand binding.

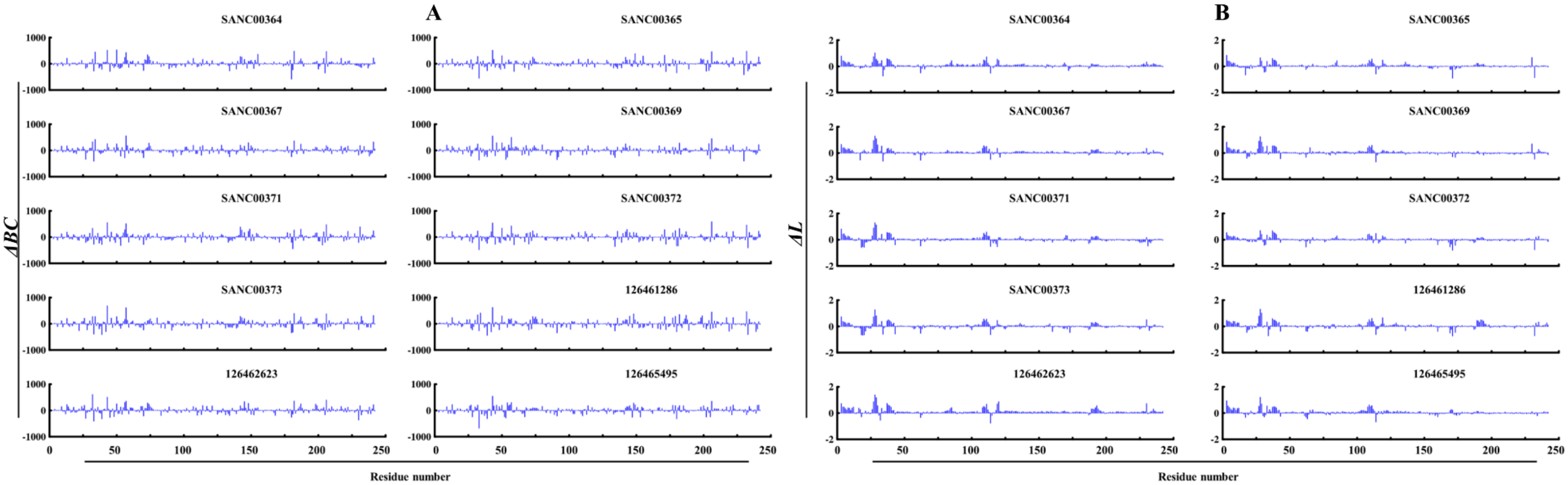

Figure S36: Plots showing BP-2 residues with significant changes in *average* a) *BC* and B) *L* upon ligand binding.

341

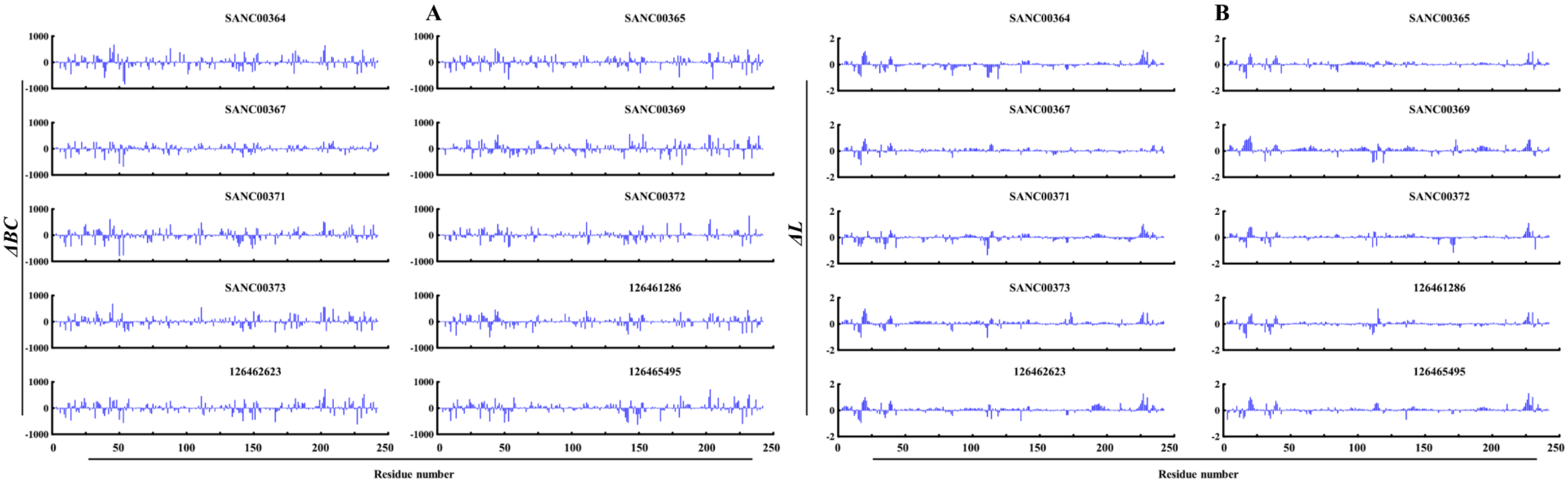

342 **Figure S37:** Plots showing CP-2 residues with significant changes in *average* a) *BC* and B) *L* upon ligand binding.

343

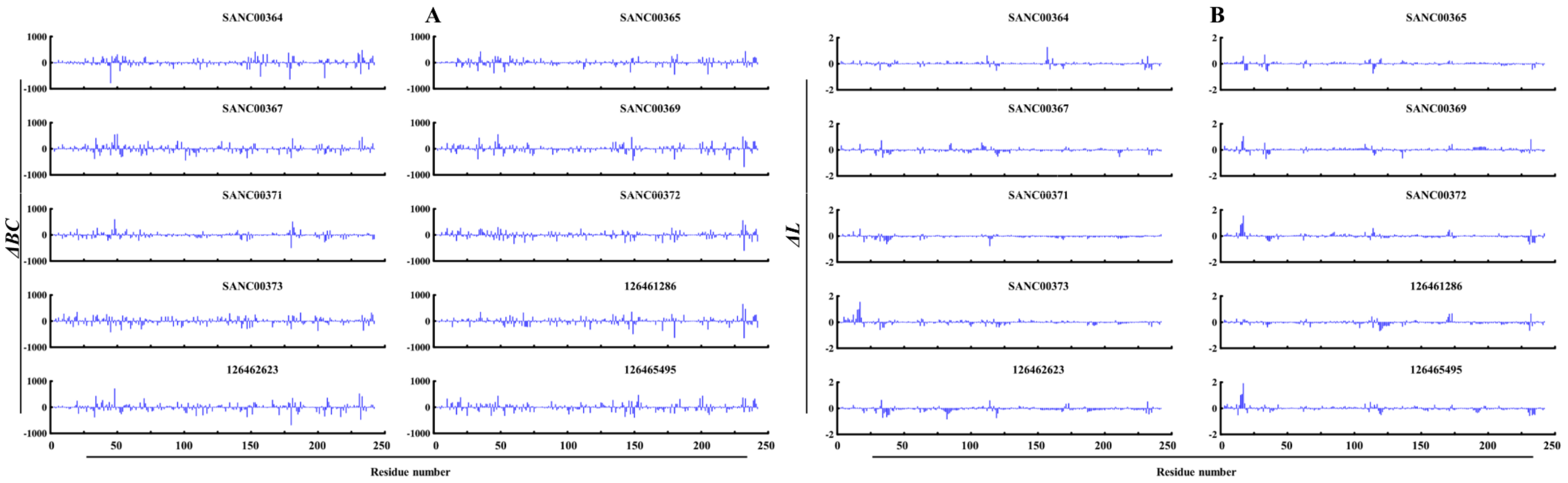

Figure S38: Plots showing YP-2 residues with significant changes in *average* a) *BC* and B) *L* upon ligand binding.

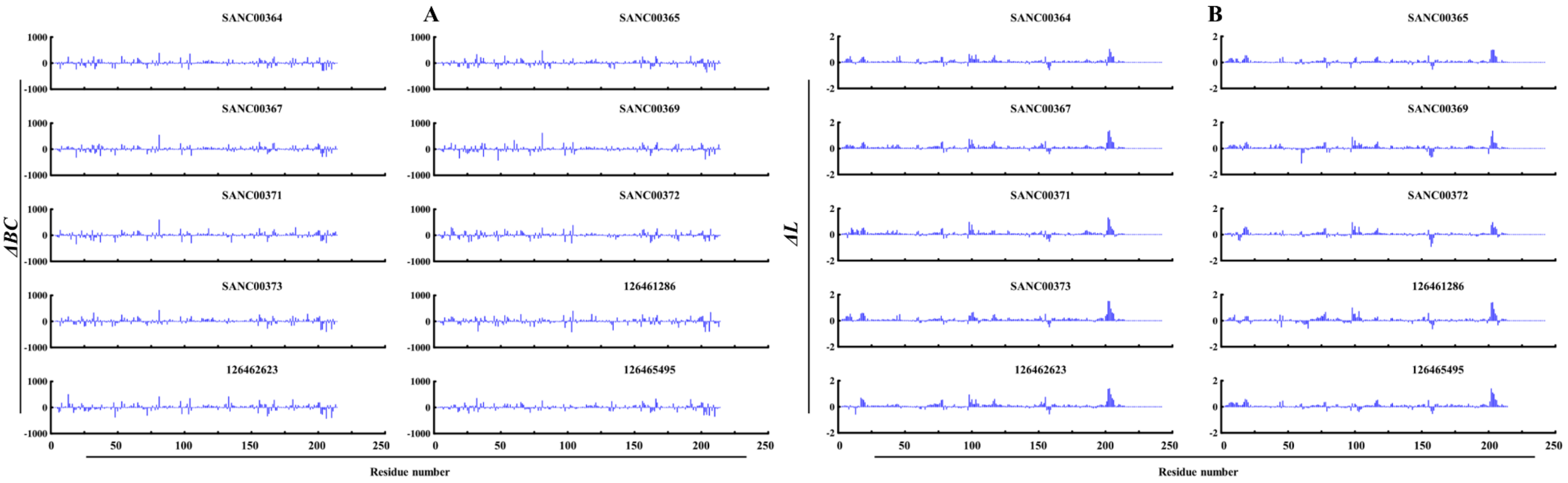

Figure S39: Plots showing Cat-K residues with significant changes in *average* a) *BC* and B) *L* upon ligand binding.

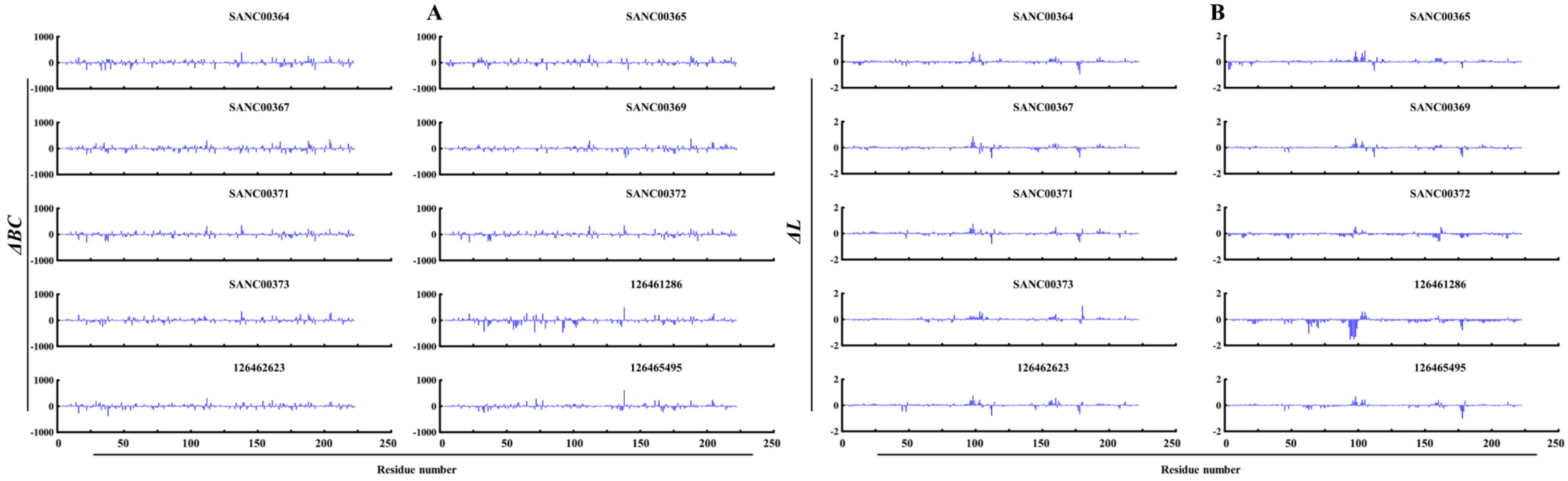

Figure S40: Plots showing Cat-L residues with significant changes in *average* a) *BC* and B) *L* upon ligand binding.

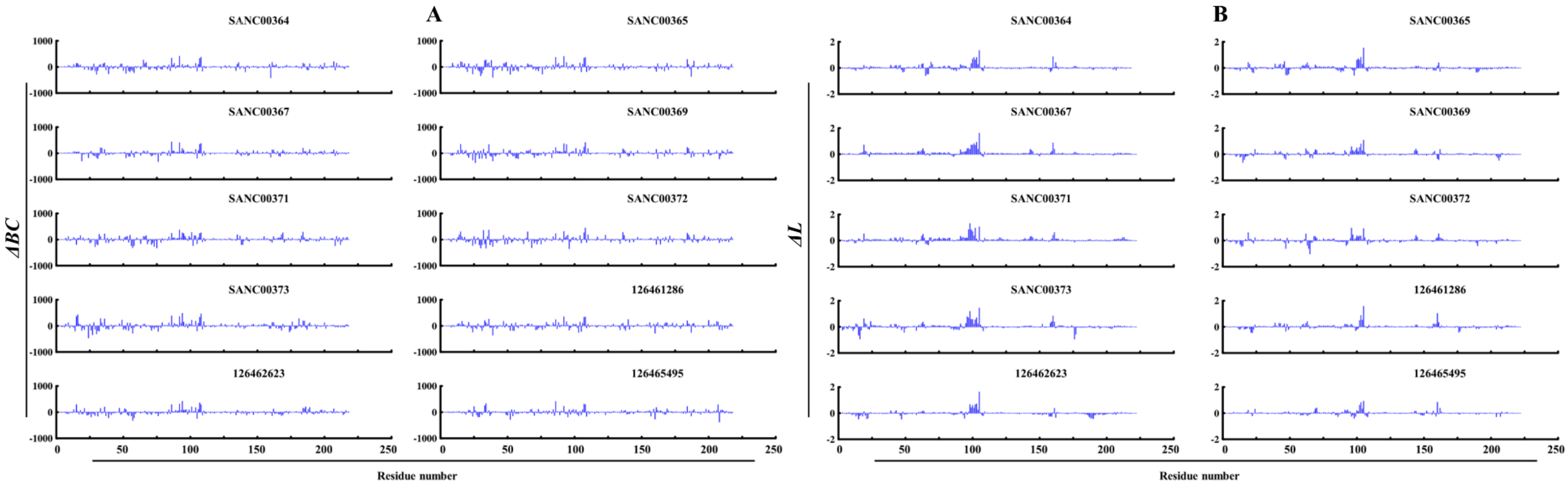

Figure S41: Plots showing Cat-S residues with significant changes in *average* a) *BC* and B) *L* upon ligand binding.

Table S1. Position of the catalytic domain of all proteins used and the corresponding domain numbering.

Protein	Position in whole sequence	Catalytic domain numbering	Sequence accession number	PDB ID
**FP-2**	244-484	1-243	PF3D7_1115700	2OUL
**FP-3**	250-492	1-242	PF3D7_1115400	3BWK
**VP-2**	246-487	1-242	PVX_091415	-
**VP-3**	253-493	1-241	PVX_091410	-
**KP-2**	252-495	1-244	PKH_091250	-
**KP-3**	240-479	1-240	PKH_091260	-
**BP-2**	228-468	1-241	PBANKA_093240	-
**CP-2**	231-471	1-241	PCHAS_091190	-
**YP-2**	232-472	1-241	PY00783	-
**Cat-K**	115-329	1-215	gi | 157830076	3OVZ
**Cat-L**	113-333	1-221	gi | 313754424	3OF8
**Cat-S**	115-331	1-217	gi | 30749675	1NPZ

Table S2. Residue interaction fingerprint between the different proteins and ligands. Shown in red, pink, green and cyan are residues interacting with S1, S2, S3 and S1’ respectively. In black represent non-subsite residues. Residues are numbered according to the catalytic domain (**Table S1**).

	**Protein**	**S1, S2,S3, S1’ and non-subsite interactions**
SANC00364	FP-2	C39,G40,A157,H174,W206,D35,C42,K37,N38,G171,S205,W210
	FP-3	Q38,G42,Y83,A159,H176,W208,D37,A39,L40,D211,W212
	VP-2	Q37,Y82,A158,H175,W207,D36,A38,N39,S210,W211
	VP-3	C39,G40,D81,V157,H174,W206,S205,W210
	KP-2	Q37,C81,D82,T158,H175,W207,C43
	KP-3	G39,C79,D80,A156,H173,W205,D34,S208,W209
	BP-2	Q37,E158,W207,D36,S206,D210,W211
	CP-2	Q37,C40,Q158,H175,W207,D36,K38,A210,W211
	YP-2	Q37,C40,A41,E158,H175,W207,D36,Q38,K39,C43
	Cat-K	Q19,C22,G23,Q143,H162,W184,N18,S24,C25,N187
	Cat-L	Q20,C23,G24,N67,H164,W190N19,W27,S189,E193,W194
	Cat-S	Q19,G23,R141,F146,H164,W186,Y18,A185,N189,F190
SANC00365	FP-2	Q36,C39,G40,A157,H174,W206,D35,K37,N38,G171,S205,Q209,W210
	FP-3	Q38,G42,Y83,A159,H176,W208,D37,A39,L40,C44,D211,W212
	VP-2	Q37,Y82,A158,H175,W207,D36,A38,N39,S210,W211
	VP-3	C39,G40,D81,V157,H174,W206,S205,W210
	KP-2	Q37,C81,D82,A153,T158,H175,W207,C43
	KP-3	Q35,C79,D80,A156,H173,W205,D34,W209
	BP-2	Q37,E158,W207,D36,S206,D210,W211
	CP-2	Q37,C40,Q158,H175,W207,D36,K38,A210,W211
	YP-2	Q37,C40,A41,E158,H175,W207,D36,Q38,K39,C43
	Cat-K	Q19,C22,G23,Q143,H162,W184,N18,S24,C25,N187
	Cat-L	Q20,C23,G24,N67,H164,W190N19,W27,S189,E193,W194
	Cat-S	Q19,G23,C66,R141,F146,H164,W186,Y18,A185,N189,F190
SANC003	FP-2	Q36,C39,G40,N173,G82,G83,A157,H174,W206,D35,K37,N38,G171,Q209,W210
	FP-3	Q38,G42,Y83,N175,G84,G85,A159,H176,W208,D37,A39,L40,D211,W212
	VP-2	Q37,C40,Y82,N174,G83,G84,V153,A158,H175,W207,D36,A38,N39,W211
	VP-3	Q36,C39,D81,N173,G82,G83,A152,V157,H174,W206,K37,N38,W43,S205,W210
	KP-2	Q37,C81,D82,N174,A176,G83,G84,I151,A153,N154,T158,H175,W207,C43,A45
	KP-3	Q35,C38,D80,N172,G81,A156,H173,W205,D34,G36,S204,S208,W209
	BP-2	Q37,C40,A41,G83,G84,N174,V153,D154,E158,W207,D36,Q38,K39,D210,W211
	CP-2	Q37,C40,A41,A173,N174,G83,G84,Q158,H175,W207,D36,R39,S206,A210,W211

67	YP-2	Q37,C40,A41,A173,N174,G83,G84,V153,E158,H175,W207,D36,Q38,K39,Y172
	Cat-K	Q19,C22,G64,N161,G65,G66,Q143,H162,W184,N18,G20,Q21,S24,N187,W188
	Cat-L	Q20,C23,G24,N67,D163,G68,G69,H164,W190N19,G21,Q22,W27,E193,W194
	Cat-S	Q19,G23,N67,N163,G68,R141,F146,H164,W186,Y18,G20,C25,N189,F190
SANC00369	FP-2	Q36,C39,G40,N173,G82,G83,A157,H174,W206,D35,K37,N38,C42,S205,Q209,W210
	FP-3	Q38,C41,Y83,N175,G84,G85,A159,H176,W208,D37,A39,L40,C44,D211,W212
	VP-2	Q37,C40,G41,Y82,N174,G83,G84,V153,H175,W207,D36,A38,N39,C43,S210,W211
	VP-3	Q36,C39,G40,D81,N173,G82,G83,A152,V157,H174,W206,K37,N38,C42,W43,S205,W210
	KP-2	Q37,C81,D82,N174,A176,G83,G84,I151,A153,T158,H175,W207,C43,W44
	KP-3	Q35,C38,G39,C79,D80,N172,G81,A156,H173,W205,D34,G36,C41,S204,S208,W209
	BP-2	Q37,N174,G83,G84,V153,D154,W207,D36,Q38,K39,D155,D210,W211
	CP-2	Q37,C40,A41,A173,N174,G83,G84,Q158,H175,W207,D36,K38,R39,A210,W211
	YP-2	Q37,C40,A41,A173,N174,G83,G84,V153,H175,W207,D36,Q38,K39,C43,Y172
	Cat-K	Q19,C22,G23,G64,N161,G65,G66,Q143,H162,W184,N18,G20,C25,N187,W188
	Cat-L	Q20,C23,G24,N67,D163,G68,G69,H164,W190,N19,G21,Q22,C26,W27,S189,E193,W194
	Cat-S	Q19,G23,C66,N67,N163,G68,R141,F146,H164,W186,Y18,G20,C25,A185,N189,F190
SANC00371	FP-2	Q36,C39,G40,N173,G82,G83,H174,W206,D35,K37,N38,C42,S205,Q209,W210
	FP-3	Q38,C41,G42,Y83,N175,G84,G85,A159,H176,W208,D37,A39,L40,C44,D211,W212
	VP-2	Q37,C40,G41,Y82,N174,G83,G84,V153,H175,W207,D36,A38,N39,S206,S210,W211
	VP-3	Q36,C39,G40,D81,N173,G82,G83,A152,V157,H174,W206,K37,N38,W43,S205,W210
	KP-2	Q37,C81,D82,N174,A176,G83,G84,N154,T158,H175,W207,C43,A45
	KP-3	Q35,C38,G39,C79,D80,N172,G81,A156,H173,W205,D34,G36,C41,S204,S208,W209
	BP-2	Q37,C40,A41,N174,G83,G84,V153,D154,E158,W207,D36,Q38,K39,T170,S206,D210,W211
	CP-2	Q37,C40,A41,A173,N174,G83,G84,Q158,H175,W207,D36,K38,R39,C43,S206,A210,W211
	YP-2	Q37,C40,A41,I85,A173,N174,G83,G84,V153,E158,H175,W207,D36,Q38,K39,C43,Y172
	Cat-K	Q19,G23,G64,N161,G65,G66,Q143,H162,W184,N18,G20,Q21,N187,W188
	Cat-L	Q20,C23,G24,N67,D163,G68,G69,H164,W190,N19,G21,Q22,S189,E193,W194
	Cat-S	Q19,G23,C66,N67,N163,G68,R141,F146,H164,W186,Y18,G20,C25,A185,N189,F190
SANC00372	FP-2	Q36,C39,G40,N173,G82,G83,A157,H174,W206,D35,K37,N38,C42,G171,S205,Q209,W210
	FP-3	Q38,C41,G42,Y83,N175,G84,G85,A159,H176,W208,D37,A39,L40,C44,D211,W212
	VP-2	Q37,C40,G41,Y82,N174,G83,G84,V153,A158,H175,W207,D36,A38,S206,S210,W211
	VP-3	Q36,C39,G40,D81,N173,G82,G83,A152,V157,H174,W206,K37,N38,W43,S205,W210
	KP-2	Q37,C81,D82,N174,A176,G83,G84,I151,A153,N154,T158,H175,W207,C43,W44,A45
	KP-3	Q35,C38,G39,C79,D80,N172,G81,A156,H173,W205,D34,G36,C41,S204,S208,W209
	BP-2	Q37,A41,N174,G83,G84,V153,D154,E158,W207,D36,Q38,K39,D155,S206,D210,W211
	CP-2	Q37,C40,A41,A173,N174,G83,G84,Q158,H175,W207,D36,K38,R39,C43,S206,A210,W211
	YP-2	Q37,C40,A41,I85,A173,N174,G83,G84,V153,E158,H175,W207,D36,Q38,K39,C43,Y172

	Cat-K	Q19,C22,G23,G64,N161,G65,G66,Q143,H162,W184,N18,G20,Q21,S24,C25,N187,W188
	Cat-L	Q20,C23,G24,N67,D163,G68,G69,H164,W190,N19,G21,Q22,C26,W27,S189,E193,W194
	Cat-S	Q19,G23,C66,N67,N163,G68,R141,F146,H164,W186,Y18,G20,C25,A185,N189,F190
SANC00373	FP-2	Q36,C39,G40,N173,G82,G83,A157,H174,W206,D35,K37,N38,C42,G171,S205,Q209,W210
	FP-3	Q38,C41,G42,Y83,N175,G84,G85,A159,H176,W208,D37,A39,L40,C44,D211,W212
	VP-2	Q37,C40,G41,Y82,N174,G83,G84,V153,A158,H175,W207,D36,A38,N39,R161,S206,S210,W211
	VP-3	Q36,C39,G40,D81,N173,G82,G83,A152,V157,H174,W206,K37,N38,C42,W43,S205,W210
	KP-2	Q37,C81,D82,N174,A176,G83,G84,I151,A153,N154,T158,H175,W207,C43
	KP-3	Q35,C38,G39,C79,D80,N172,G81,A156,H173,W205,D34,G36,C41,S204,S208,W209
	BP-2	Q37,C40,A41,N174,G83,G84,V153,D154,E158,W207,D36,Q38,K39,D155,T170,S206,D210,W211
	CP-2	Q37,C40,A41,A173,N174,G83,G84,Q158,H175,W207,D36,K38,R39,C43,S206,A210,W211
	YP-2	Q37,C40,A41,I85,A173,N174,G83,G84,V153,E158,H175,W207,D36,Q38,K39,C43,Y172
	Cat-K	Q19,C22,G23,G64,N161,G65,G66,Q143,H162,W184,N18,G20,Q21,S24,C25,N187,W188
	Cat-L	Q20,C23,G24,N67,D163,G68,G69,H164,W190N19,G21,Q22,C26,W27,S189,E193,W194
	Cat-S	Q19,G23,C66,N67,N163,G68,R141,F146,H164,W186,Y18,G20,C25,A185,N189,F190
126461286	FP-2	Q36,C39,G40,C80,N81,L84,I85,S149,L172,N173,A175,D234,G82,G83,V152,A157,H174,W206,D35,K37,C42,W43,D154
	FP-3	Q38,C41,G42,C82,Y86,I87,S51,P174,N175,E236,G84,G85,A159,H176,W208,D37,A39,L40,C44,F160,R162
	VP-2	Q37,C40,G41,Y82,N84,F85,S149,P173,N174,E235,T79,G83,G84,A158,H175,W207,A38,N39,C43,D44,D211
	VP-3	Q36,C39,G40,D81,N84,S149,P172,N173,A175,Q234,Y78,G82,G83,V157,H174,W206,K37,N38,C42,W43,W210
	KP-2	Q37,C40,G41,L85,S150,P173,N174,A176,E235,G83,G84,I151,T158,H175,W207,D36,K38,N39,C43,W44,K210
	KP-3	Q35,C38,G39,D80,F83,N148,T171,N172,A174,E233,G81,G82,A156,H173,W205,D34,G36,D37,C41,W42,D153,S204
	BP-2	Q37,C40,A41,E82,I85,L86,A150,A173,N174,A176,F79,G83,G84,V151,V153,E158,W207,Q38,C43,W44,W211
	CP-2	Q37,C40,A41,E82,I85,L86,A150,A173,N174,A176,G83,G84,A153,S154,Q158,H175,W207,C43,D44,E155,F157
	YP-2	Q37,C40,A41,D82,I85,A150,A173,N174,A176,F79,G83,G84,V151,E158,H175,W207,D36,Q38,K39,C43,W44,Y172
	Cat-K	Q19,C22,G23,C63,Y67,L160,N161,D61,G66,G65,Q143, H162,W184,G20,Q21,C25,D26,W188
	Cat-L	Q20,C23,G24,N67,L70,D163,G165,E64,G68,G69,L145,H164,W190,G21,Q22,C26,W27,W194
	Cat-S	Q19,G23,C66,N67,F70,N163,V162,G165,G68,G69,V138,A140,R141,F146,H164,C25,W26,H142,P143
126462623	FP-2	Q36,C39,G40,C80,N81,L84,I85,S149,L172,N173,A175,D234,G82,G83,V152,A157,H174,W206,K37,N38,C42,W43,D154
	FP-3	Q38,C41,G42,C82,Y86,I87,S51,P174,N175,E236,G84,G85,A159,H176,W208,D37,A39,L40,C44,F160,R162
	VP-2	Q37,C40,G41,Y82,F85,S149,P173,N174,E235,T79,G83,G84,A158,H175,W207,A38,N39,C43,D44,D211
	VP-3	Q36,,C39,G40,D81,N84,S149,P172,N173,A175Q234,Y78,G82,G83,V157,H174,W206,K37,N38,C42,W43,W210
	KP-2	Q37,C40,G41,L85,S150,P173,N174,A176,E235,G83,G84,I151,T158,H175,W207,D36,K38,N39,C43,W44,K210
	KP-3	Q35,C38,G39,D80,F83,N148,T171,N172,A174,E233,G81,G82,A156,H173,W205,D34,G36,D37,C41,W42,D153,S204
	BP-2	Q37,C40,A41,E82,I85,L86,A150,A173,N174,A176,F79,G83,G84,V151,V153,E158,W207,Q38,C43,W44,W211
	CP-2	Q37,C40,A41,E82,I85,L86,A150,A173,N174,A176,G83,G84,A153,S154,Q158,H175,W207,C43,D44,E155,F157
	YP-2	Q37,C40,A41,D82,I85,A150,A173,N174,A176,F79,G83,G84,V151,E158,H175,W207,D36,Q38,K39,C43,W44,Y172
	Cat-K	Q19,C22,G23,C63,Q143, H162,W184,G20,Q21,C25,D26,W188

	Cat-L	Q20,C23,G24,N67,L70,G68,G69,L145,H164,W190,G21,Q22,C26,W27,W194
	Cat-S	Q19,G23,C66,N67,F70,N163,G68,G69,V138,A140,R141,F146,H164,C25,W26,H142,P143
126465495	FP-2	Q36,C39,G40,C80,N81,L84,I85,S149,L172,N173,A175,D234,G82,G83,V152,A157,H174,W206,D35,N38,C42,W43,D154
	FP-3	Q38,C41,G42,C82,Y86,I87,S51,P174,N175,E236,G84,G85,A159,H176,W208,D37,A39,L40,C44,F160,R162
	VP-2	Q37,C40,G41,Y82,N84,F85,S149,P173,N174,E235,T79,G83,G84,A158,H175,W207,A38,N39,C43,D44,D211
	VP-3	Q36,,C39,G40,D81,N84,S149,P172,N173,A175Q234,Y78,G82,G83,V157,H174,W206,K37,N38,C42,W43,W210
	KP-2	Q37,C40,G41,L85,S150,P173,N174,A176,E235,G83,G84,I151,T158,H175,W207,D36,K38,N39,C43,W44,K210
	KP-3	Q35,C38,G39,D80,F83,N148,T171,N172,A174,E233,G81,G82,A156,H173,W205,D34,G36,D37,C41,W42,D153,S204
	BP-2	Q37,C40,A41,E82,I85,L86,A150,A173,N174,A176,F79,G83,G84,V151,V153,E158,W207,Q38,C43,W44,W211
	CP-2	Q37,C40,A41,E82,I85,L86,A150,A173,N174,A176,G83,G84,A153,S154,Q158,H175,W207,C43,D44,E155,F157
	YP-2	Q37,C40,A41,D82,I85,A150,A173,N174,A176,F79,G83,G84,V151,E158,H175,W207,D36,Q38,K39,C43,W44,Y172
	Cat-K	Q19,C22,G23,C63,Y67,L160,N161,D61,G66,G65,Q143, H162,W184,G20,Q21,C25,D26,W188
	Cat-L	Q20,C23,G24,N67,L70,D163,G165,E64,G68,G69,L145,H164,W190,G21,Q22,C26,W27,W194
	Cat-S	Q19,G23,C66,N67,F70,N163,V162,G165,G68,G69,V138,A140,R141,F146,H164,C25,W26,H142,P143

Table S3. RMSD distribution statistics. Apo and ligand bound RMSD means were compared using the z-test with $\alpha = 0.05$ and a H_0 of $\mu 1 - \mu 2 = 0$. The two-sample KS-test was used to compare the shapes of the distributions between the apo and ligand bound systems μ = Mean, σ = Standard deviation and σ^2 = Variance.

Protein	Ligand	μ	σ	σ^2	Z-test	P	KS	P
**FP-2**	Apo	0.23	0.04	0.00E+00	-	-	-	-
	SANC00364	0.26	0.02	0.00E+00	-154.62	0.00E+00	0.44	0.00E+00
	SANC00365	0.28	0.04	0.00E+00	-183.30	0.00E+00	0.46	0.00E+00
	SANC00367	0.24	0.01	0.00E+00	-81.74	0.00E+00	0.39	0.00E+00
	SANC00369	0.30	0.02	0.00E+00	-206.21	0.00E+00	0.55	0.00E+00
	SANC00371	0.23	0.02	0.00E+00	-64.90	0.00E+00	0.35	0.00E+00
	SANC00372	0.23	0.02	0.00E+00	-46.98	0.00E+00	0.31	0.00E+00
	SANC00373	0.28	0.03	0.00E+00	-218.92	0.00E+00	0.55	0.00E+00
	126461286	0.24	0.02	0.00E+00	-94.70	0.00E+00	0.39	0.00E+00
	126462623	0.22	0.02	0.00E+00	-13.80	0.00E+00	0.20	0.00E+00
	126465495	0.23	0.02	0.00E+00	-72.20	0.00E+00	0.35	0.00E+00
**FP-3**	Apo	0.21	0.03	0.00E+00	-	-	-	-
	SANC00364	0.25	0.02	0.00E+00	-202.76	0.00E+00	0.52	0.00E+00
	SANC00365	0.24	0.02	0.00E+00	-208.70	0.00E+00	0.59	0.00E+00
	SANC00367	0.24	0.02	0.00E+00	-201.48	0.00E+00	0.60	0.00E+00
	SANC00369	0.24	0.01	0.00E+00	-227.98	0.00E+00	0.66	0.00E+00
	SANC00371	0.24	0.02	0.00E+00	-187.86	0.00E+00	0.51	0.00E+00
	SANC00372	0.22	0.01	0.00E+00	-93.33	0.00E+00	0.30	0.00E+00
	SANC00373	0.25	0.01	0.00E+00	-217.16	0.00E+00	0.62	0.00E+00
	126461286	0.26	0.03	0.00E+00	-231.8	0.00E+00	0.60	0.00E+00
	126462623	0.23	0.01	0.00E+00	-181.81	0.00E+00	0.57	0.00E+00
	126465495	0.24	0.02	0.00E+00	-168.71	0.00E+00	0.44	0.00E+00
**VP-2**	Apo	0.29	0.04	0.00E+00	-	-	-	-
	SANC00364	0.31	0.02	0.00E+00	-91.98	0.00E+00	0.28	0.00E+00
	SANC00365	0.32	0.02	0.00E+00	-178.04	0.00E+00	0.49	0.00E+00
	SANC00367	0.25	0.02	0.00E+00	60.4	0.00E+00	0.44	0.00E+00
	SANC00369	0.28	0.04	0.00E+00	-3.5	0.00E+00	0.20	0.00E+00

	SANC00371	0.29	0.02	0.00E+00	-21.14	0.00E+00	0.22	0.00E+00
	SANC00372	0.33	0.03	0.00E+00	-116.97	0.00E+00	0.31	0.00E+00
	SANC00373	0.31	0.02	0.00E+00	-87.62	0.00E+00	0.26	0.00E+00
	126461286	0.25	0.02	0.00E+00	94.48	0.00E+00	0.48	0.00E+00
	126462623	0.30	0.02	0.00E+00	-54.51	0.00E+00	0.23	0.00E+00
	126465495	0.27	0.02	0.00E+00	10.86	0.00E+00	0.34	0.00E+00
**VP-3**	Apo	0.22	0.02	0.00E+00	-	-	-	-
	SANC00364	0.29	0.01	0.00E+00	-464.45	0.00E+00	0.86	0.00E+00
	SANC00365	0.28	0.02	0.00E+00	-415.64	0.00E+00	0.85	0.00E+00
	SANC00367	0.26	0.01	0.00E+00	-360.13	0.00E+00	0.79	0.00E+00
	SANC00369	0.28	0.02	0.00E+00	-393.7	0.00E+00	0.78	0.00E+00
	SANC00371	0.34	0.02	0.00E+00	-532.2	0.00E+00	0.88	0.00E+00
	SANC00372	0.31	0.02	0.00E+00	-488.58	0.00E+00	0.85	0.00E+00
	SANC00373	0.25	0.02	0.00E+00	-234.22	0.00E+00	0.61	0.00E+00
	126461286	0.28	0.01	0.00E+00	-465.51	0.00E+00	0.87	0.00E+00
	126462623	0.28	0.02	0.00E+00	-379.77	0.00E+00	0.77	0.00E+00
	126465495	0.28	0.02	0.00E+00	-460.39	0.00E+00	0.89	0.00E+00
**KP-2**	Apo	0.22	0.02	0.00E+00	-	-	-	-
	SANC00364	0.29	0.03	0.00E+00	-360.35	0.00E+00	0.78	0.00E+00
	SANC00365	0.34	0.01	0.00E+00	-578.07	0.00E+00	0.92	0.00E+00
	SANC00367	0.31	0.02	0.00E+00	-474.26	0.00E+00	0.88	0.00E+00
	SANC00369	0.28	0.01	0.00E+00	-379.45	0.00E+00	0.83	0.00E+00
	SANC00371	0.29	0.02	0.00E+00	-350.32	0.00E+00	0.76	0.00E+00
	SANC00372	0.28	0.02	0.00E+00	-320.62	0.00E+00	0.71	0.00E+00
	SANC00373	0.29	0.02	0.00E+00	-388.11	0.00E+00	0.78	0.00E+00
	126461286	0.29	0.02	0.00E+00	-450.16	0.00E+00	0.86	0.00E+00
	126462623	0.31	0.02	0.00E+00	-482.63	0.00E+00	0.86	0.00E+00
	126465495	0.29	0.02	0.00E+00	-408.63	0.00E+00	0.81	0.00E+00
**KP-3**	Apo	0.19	0.03	0.00E+00	-	-	-	-
	SANC00364	0.25	0.02	0.00E+00	-343.66	0.00E+00	0.69	0.00E+00
	SANC00365	0.24	0.02	0.00E+00	-306.66	0.00E+00	0.68	0.00E+00

	SANC00367	0.26	0.01	0.00E+00	-367.48	0.00E+00	0.69	0.00E+00
	SANC00369	0.24	0.01	0.00E+00	-309.22	0.00E+00	0.66	0.00E+00
	SANC00371	0.27	0.02	0.00E+00	-429.2	0.00E+00	0.83	0.00E+00
	SANC00372	0.24	0.03	0.00E+00	-438.07	0.00E+00	0.84	0.00E+00
	SANC00373	0.27	0.02	0.00E+00	-359.83	0.00E+00	0.75	0.00E+00
	126461286	0.25	0.02	0.00E+00	-310.29	0.00E+00	0.64	0.00E+00
	126462623	0.24	0.02	0.00E+00	-463.81	0.00E+00	0.85	0.00E+00
	126465495	0.30	0.02	0.00E+00	-308.78	0.00E+00	0.63	0.00E+00
**BP-2**	Apo	0.24	0.02	0.00E+00	-	-	-	-
	SANC00364	0.29	0.03	0.00E+00	-301.03	0.00E+00	0.69	0.00E+00
	SANC00365	0.31	0.03	0.00E+00	-337.42	0.00E+00	0.78	0.00E+00
	SANC00367	0.30	0.04	0.00E+00	-222.28	0.00E+00	0.60	0.00E+00
	SANC00369	0.29	0.03	0.00E+00	-232.34	0.00E+00	0.51	0.00E+00
	SANC00371	0.32	0.02	0.00E+00	-479.61	0.00E+00	0.87	0.00E+00
	SANC00372	0.31	0.03	0.00E+00	-403.39	0.00E+00	0.84	0.00E+00
	SANC00373	0.30	0.03	0.00E+00	-342.86	0.00E+00	0.70	0.00E+00
	126461286	0.32	0.02	0.00E+00	-493.69	0.00E+00	0.89	0.00E+00
	126462623	0.31	0.03	0.00E+00	-327.49	0.00E+00	0.71	0.00E+00
	126465495	0.29	0.03	0.00E+00	-289.83	0.00E+00	0.69	0.00E+00
**CP-2**	Apo	0.26	0.02	0.00E+00	-	-	-	-
	SANC00364	0.28	0.02	0.00E+00	-110.76	0.00E+00	0.28	0.00E+00
	SANC00365	0.26	0.01	0.00E+00	-21.04	0.00E+00	0.12	0.00E+00
	SANC00367	0.29	0.02	0.00E+00	-409.57	0.00E+00	0.83	0.00E+00
	SANC00369	0.37	0.02	0.00E+00	-198.16	0.00E+00	0.59	0.00E+00
	SANC00371	0.30	0.02	0.00E+00	-209.55	0.00E+00	0.60	0.00E+00
	SANC00372	0.32	0.02	0.00E+00	-243.35	0.00E+00	0.64	0.00E+00
	SANC00373	0.30	0.02	0.00E+00	-126.52	0.00E+00	0.32	0.00E+00
	126461286	0.29	0.04	0.00E+00	-17.66	0.00E+00	0.16	0.00E+00
	126462623	0.25	0.01	0.00E+00	-15.73	0.00E+00	0.12	0.00E+00
	126465495	0.25	0.02	0.00E+00	-34.67	0.00E+00	0.17	0.00E+00
**YP-2**	Apo	0.19	0.02	0.00E+00	-	-	-	-

	SANC00364	0.31	0.02	0.00E+00	-583.12	0.00E+00	0.90	0.00E+00
	SANC00365	0.26	0.01	0.00E+00	-647.93	0.00E+00	0.96	0.00E+00
	SANC00367	0.28	0.02	0.00E+00	-615.2	0.00E+00	0.95	0.00E+00
	SANC00369	0.31	0.03	0.00E+00	-615.81	0.00E+00	0.95	0.00E+00
	SANC00371	0.28	0.02	0.00E+00	-562.32	0.00E+00	0.87	0.00E+00
	SANC00372	0.31	0.02	0.00E+00	-487.67	0.00E+00	0.89	0.00E+00
	SANC00373	0.32	0.03	0.00E+00	-437.87	0.00E+00	0.87	0.00E+00
	126461286	0.29	0.01	0.00E+00	-478.65	0.00E+00	0.78	0.00E+00
	126462623	0.28	0.01	0.00E+00	-465.45	0.00E+00	0.87	0.00E+00
	126465495	0.32	0.03	0.00E+00	-435.56	0.00E+00	0.89	0.00E+00
**Cat-K**	Apo	0.23	0.02	0.00E+00	-	-	-	-
	SANC00364	0.22	0.02	0.00E+00	13.88	0.00E+00	0.12	0.00E+00
	SANC00365	0.20	0.01	0.00E+00	103.75	0.00E+00	0.54	0.00E+00
	SANC00367	0.22	0.01	0.00E+00	18.09	0.00E+00	0.76	0.00E+00
	SANC00369	0.22	0.01	0.00E+00	-7.23	0.00E+00	0.45	0.00E+00
	SANC00371	0.22	0.01	0.00E+00	-18.23	0.00E+00	0.56	0.00E+00
	SANC00372	0.23	0.02	0.00E+00	-30.41	0.00E+00	0.59	0.00E+00
	SANC00373	0.22	0.02	0.00E+00	27.79	0.00E+00	0.45	0.00E+00
	126461286	0.24	0.01	0.00E+00	-10.55	0.00E+00	0.78	0.00E+00
	126462623	0.22	0.01	0.00E+00	25.77	0.00E+00	0.81	0.00E+00
	126465495	0.22	0.02	0.00E+00	-17.78	0.00E+00	0.78	0.00E+00
**Cat-L**	Apo	0.17	0.02	0.00E+00	-	0.00E+00	-	0.00E+00
	SANC00364	0.20	0.01	0.00E+00	-226.70	0.00E+00	0.73	0.00E+00
	SANC00365	0.26	0.02	0.00E+00	-435.27	0.00E+00	0.56	0.00E+00
	SANC00367	0.22	0.01	0.00E+00	-312.34	0.00E+00	0.67	0.00E+00
	SANC00369	0.22	0.02	0.00E+00	-329.78	0.00E+00	0.78	0.00E+00
	SANC00371	0.21	0.01	0.00E+00	-335.45	0.00E+00	0.78	0.00E+00
	SANC00372	0.27	0.01	0.00E+00	-356.65	0.00E+00	0.78	0.00E+00
	SANC00373	0.23	0.01	0.00E+00	-323.45	0.00E+00	0.78	0.00E+00
	126461286	0.31	0.02	0.00E+00	-336.45	0.00E+00	0.84	0.00E+00
	126462623	0.22	0.01	0.00E+00	-336.45	0.00E+00	0.92	0.00E+00

	126465495	0.21	0.02	0.00E+00	-336.67	0.00E+00	0.65	0.00E+00
	Apo	0.21	0.02	0.00E+00	-	-	-	0.00E+00
	SANC00364	0.24	0.02	0.00E+00	-184.67	0.00E+00	0.91	0.00E+00
	SANC00365	0.26	0.01	0.00E+00	-295.56	0.00E+00	0.67	0.00E+00
	SANC00367	0.22	0.02	0.00E+00	-70.16	0.00E+00	0.78	0.00E+00
	SANC00369	0.25	0.01	0.00E+00	-272.67	0.00E+00	0.45	0.00E+00
**Cat-S**	SANC00371	0.24	0.02	0.00E+00	-180.98	0.00E+00	0.89	0.00E+00
	SANC00372	0.33	0.02	0.00E+00	-368.78	0.00E+00	0.67	0.00E+00
	SANC00373	0.26	0.01	0.00E+00	-266.67	0.00E+00	0.87	0.00E+00
	126461286	0.27	0.01	0.00E+00	-381.78	0.00E+00	0.98	0.00E+00
	126462623	0.26	0.03	0.00E+00	-232.56	0.00E+00	0.78	0.00E+00
	126465495	0.25	0.02	0.00E+00	-204.56	0.00E+00	0.98	0.00E+00

Table S4: Ligand-residue interaction fingerprint at different MD simulation time steps. VDR and HBR indicate the number of residues involved in van der Waals (hydrophobic) interactions and those involved in hydrogen (HBR) bonding respectively. Residue are numbered based on the catalytic domain length of each protein (**Table S1**).

	Compound										
**Protein**	**Time (ns)**	**Type**	**SANC00364**	**SANC00365**	**SANC00367**	**SANC00369**	**SANC00371**	**SANC00372**	**126461286**	**126462623**	**126465495**
**Cat-K**	**0**	**HBR**	W184	W184	Q19,W184	Q19,W184	Q19,W184	Q19,W184	Q19,W184	Q19,W184	Q19
		**VDR**	8	9	13	12	15	17	20	20	23
	**20**	**HBR**	W184	W184	W184	Q19	W184	W184	W184	W184	Q19,N161
		**VDR**	10	8	12	13	13	16	20	21	22
	**40**	**HBR**	W184	W184	W184	Q19	Q19,W184	W184	W184	Q19	W184
		**VDR**	9	7	13	14	15	17	22	21	22
	**60**	**HBR**	W184	W184	Q19,W184	Q19	Q19	Q19,W184	W184	W184	N161,W184
		**VDR**	10	8	13	12	12	15	19	21	23
	**80**	**HBR**	W184	W184	W184	W184	W184	Q19,W184	W184	W184	G65
		**VDR**	9	11	14	13	14	17	21	21	22
	**100**	**HBR**	W184	W184	W184	W184	W184	Q19,W184	W184	W184	G65,N161
		**VDR**	11	8	13	12	14	16	20	20	21
**Cat-L**	**0**	**HBR**	Q20	Q20	N19,D163	Q20	G21	Q20	G21,W190	Q20,G21	Q20,W190
		**VDR**	9	12	15	13	15	16	20	22	23
	**20**	**HBR**	Q20	Q20	Q20	Q20	G21,W190	Q20	G21	Q20,W190	W190
		**VDR**	10	7	14	12	13	15	23	23	22
	**40**	**HBR**	Q20	W190	N19,W190	Q20	G21	Q20	W190	W190	W190
		**VDR**	14	8	14	13	12	16	23	20	23
	**60**	**HBR**	-	W190	N19,W190	-	W190	-	W190	W190	Q20
		**VDR**	12	8	13	12	14	17	20	21	22
	**80**	**HBR**	Q20	W190	W190	Q20,D163	W190	W190	G21	W190	Q20,W190
		**VDR**	8	12	14	13	13	616	20	21	24
	**100**	**HBR**	W190	W190	W190	W190	G21,W190	W190	W190	W190	Q20
		**VDR**	9	8	15	12	13	15	20	15	22
**Cat-S**	**0**	**HBR**	Q19	Q19	Q19,W186	W186	Q19	Q19	W186	Q19	Q19
		**VDR**	10	11	15	13	14	16	23	23	22
	**20**	**HBR**	W186	Q19	Q19	Q19,W186	-	W186	W186	Q19,W186	Q19,W186
		**VDR**	8	11	14	12	13	17	20	21	23
	**40**	**HBR**	W186	Q19	Q19,W186	W186	W186	Q19	Q19	Q19,W186	Q19
		**VDR**	9	10	13	12	13	15	22	21	21
	**60**	**HBR**	W186	Q19	Q19	Q19	Q19	-	W186	Q19,W186	W186
		**VDR**	10	10	12	14	13	15	21	21	22
	**80**	**HBR**	W186	Q19	Q19	Q19,W186	W186	W186	W186	Q19	Q19
		**VDR**	8	11	13	13	12	16	20	23	20
	**100**	**HBR**	W186	Q19	Q19,W186	Q19,W186	W186	Q19	W186	Q19,W186	W186
		**VDR**	10	11	12	13	13	16	19	21	19

**FP-2**	**0**	**HBR**	C42,W206	C42,W206	K37,C39,W207	C39,W207	Q36,N38,W206	Q36,W206	Q36,N81,G83,W206	Q36,N81,G83,N173, W206	Q36,N81,G83,N173, W206
		**VDR**	11	11	13	12	12	17	22	22	25
	**20**	**HBR**	W206	W206	K37	C39,W207	Q36,N38,W206	Q36,N38,W206	Q36,G83,W206	Q36,N81,G83,N173, W206	Q36,G83,N173,W206
		**VDR**	12	10	14	13	13	16	22	24	24
	**40**	**HBR**	C42,W206	C42,W206	C39,W207	C39,W207	0	Q36,N38,W206	Q36,N81,G83,N173, W206	Q36,N81,G83,N173, W206	Q36,N81,G83,N173, W206
		**VDR**	10	12	12	12	13	15	23	25	26
	**60**	**HBR**	C42	C42	K37,C39	C39,W207	Q36,N38,W206	Q36,N38,W206	Q36,G83,N173,W206	Q36,N81,G83,N173, W206	Q36,N81,G83,N173, W206
		**VDR**	9	12	12	12	14	15	19	24	22
	**80**	**HBR**	C42,W206	C42	K37,C39	C39,W207	Q36,W206	Q36,N38,W206	G83	Q36,N81,G83,N173, W206	Q36,N81,G83,N173, W206
		**VDR**	12	9	13	13	13	16	22	24	24
	**100**	**HBR**	C42,W206	C42,W206	K37,C39	C39,W207	Q36,N38,W206	0	G83,N173	Q36,N81,G83,N173, W206	Q36,N81,G83,N173, W206
		**VDR**	12	10	13	12	13	16	23	26	25
**FP-3**	**0**	**HBR**	G42,W208	G42,W208	G84,W208	Q38,W208	G40,G85,W208	G85,W208	G42,G85,N175,W208	G42,G85,N175,W208	0
		**VDR**	10	11	14	12	13	15	20	21	27
	**20**	**HBR**	G42	G42	G84,W208	Q38,W208	G40,W208	G85,W208	G42,G85,N175,W208	G42,G85,N175,W208	G42,G85,N175,W208
		**VDR**	8	9	14	12	13	17	21	23	28
	**40**	**HBR**	G42,W208	W208	G84,W208	Q38,W208	G40,G85,W208	G85,W208	G85,N175,W208	G42,N175,W208	G42,G85,N175,W208
		**VDR**	12	10	13	13	14	18	23	24	23
	**60**	**HBR**	G42	G42	G84,W208	G84,W208	G40,W208	G85,W208	G42,G85,N175,W208	G42,G85,N175,W208	G85,N175,W208
		**VDR**	11	11	14	13	14	16	24	25	25
	**80**	**HBR**	G42,W208	G42	G84,W208	G84,W208	G40,G85,W208	G85,W208	G42,G85,N175,W208	G42,G85,N175,W208	G42,G85,N175,W208
		**VDR**	12	10	13	13	14	16	25	22	27
	**100**	**HBR**	G42	W208	G84,W208	G84,W208	G40,G85,W208	G85,W208	G42,G85,N175,W208	G42,G85,N175,W208	G42,G85,N175,W208
		**VDR**	12	10	13	12	13	17	21	22	22
**VP-2**	**0**	**HBR**	D36,W207	D36,W207	A38,H175,W207	A38,H175,W207	Q37,A38,W207	G41,Y82	Q37,G84,N174,W207	Q37,G84,N174,W207	Q37,G84,N174,W207
		**VDR**	11	10	13	13	13	15	24	25	23
	**20**	**HBR**	D36	W207	A38,W207	A38,H175,W207	Q37,W207	Q37,A38,W207	Q37,N174,W207	Q37,G84,N174,W207	Q37,G84,N174,W207
		**VDR**	10	10	14	12	14	14	24	22	24
	**40**	**HBR**	W207	D36,W207	A38,W207	A38,W207	Q37,A38,W207	Q37,A38,W207	Q37,G84,N174,W207	Q37,N174,W207	Q37,G84,N174,W207
		**VDR**	11	12	13	11	13	16	22	24	24
	**60**	**HBR**	D36,W207	D36,W207	A38,H175,W207	A38,H175,W207	Q37,A38,W207	Q37,A38,W207	Q37,N174,W207	Q37,N174,W207	Q37,G84,N174,W207

		**VDR**	12	8	14	12	13	17	24	23	25
	**80**	**HBR**	W207	D36,W207	A38,W207	A38,H175,W207	Q37,A38,W207	Q37,A38,W207	Q37,G84,N174,W207	Q37,G84,N174,W207	G84,N174,W207
		**VDR**	15	12	11	12	12	16	20	22	25
	**100**	**HBR**	D36,W207	D36,W207	A38,H175,W207	0	Q37,A38,W207	Q37,A38,W207	N174	Q37,G84,N174,W207	G84,N174,W207
		**VDR**	11	13	13	12	12	16	21	25	24
**VP-3**	**0**	**HBR**	D81,W206	D81,W206	Q36,W206	Q36,W206	Q36,W206	G40,G83,W206	G40,N173,Q234,W206	G40,N173,Q234,W206	G83,N173
		**VDR**	11	12	13	13	13	17	16	22	26
	**20**	**HBR**	D81,W206	D81,W206	Q36,W206	W206	Q36,W206	G40,G83,W206	G40,N173,Q234,W206	G40,N173,Q234,W206	G83,Q234,W206
		**VDR**	12	11	12	12	12	16	22	24	22
	**40**	**HBR**	D81,W206	D81	W206	Q36,W206	W206	G40,W206	G40,N173,Q234,W206	G40,N173,Q234,W206	G83,Q234,W206
		**VDR**	13	11	12	12	12	17	23	24	22
	**60**	**HBR**	W206	W206	Q36,W206	Q36	Q36,W206	G40,G83,W206	G40,N173,Q234,W206	G40,N173,Q234,W206	G83,Q234,W206
		**VDR**	11	10	13	11	13	17	24	25	21
	**80**	**HBR**	D81,W206	D81,W206	Q36,W206	W206	Q36,W206	G40,G83,W206	G40,N173,Q234,W206	G40,N173,Q234,W206	G83,Q234,W206
		**VDR**	11	11	13	13	13	16	23	23	23
	**100**	**HBR**	W206	D81,W206	Q36,W206	Q36,W206	Q36,W206	G40,W206	0	0	G83,Q234,W206
		**VDR**	11	10	13	13	14	16	22	22	21
**KP-2**	**0**	**HBR**	Q37,W207	D36,W207	Q37,W207	W207	Q37,G84,W207	Q37,G84,W207	C40,G84,E235,W207	Q37,T158	G84,T158,N174
		**VDR**	10	13	12	12	13	16	23	24	22
	**20**	**HBR**	Q37	Q37	Q37,W207	Q37,W207	Q37,G84,W207	Q37,G84,W207	C40,G84,E235,W207	C40,G84,E235,W207	C40,G84,E235,W207
		**VDR**	11	11	12	13	12	16	19	23	24
	**40**	**HBR**	Q37,W207	Q37	Q37	Q37,W207	W207	G40,W206	C40,G84,E235,W207	C40,E235,W207	C40,G84,E235,W207
		**VDR**	11	12	13	13	13	18	23	24	22
	**60**	**HBR**	Q37	Q37,W207	Q37,W207	Q37,W207	Q37,G84,W207	G40,W206	C40,E235,W207	C40,G84,E235,W207	C40,G84,E235,W207
		**VDR**	10	11	12	12	15	16	24	22	22
	**80**	**HBR**	Q37,W207	Q37	Q37,W207	Q37,W207	Q37,W207	G40,W206	C40,G84,E235,W207	C40,G84,E235,W207	C40,G84,E235,W207
		**VDR**	12	11	12	13	14	17	24	24	23
	**100**	**HBR**	Q37,W207	Q37,W207	Q37,W207	Q37,W207	Q37,G84,W207	G40,W206	G84,E235,W207	C40,G84,E235,W207	C40,G84,E235,W207
		**VDR**	11	12	13	12	13	16	23	23	23
**KP-3**	**0**	**HBR**	D34,W205	W205	D34,C38,W205	D34,C38,W205	D34,G39,W205	D34,G39,W205	D34,G82,W205,E233	N172	N172
		**VDR**	11	11	13	12	13	18	22	24	22
	**20**	**HBR**	D34	D34,W205	D34,W205	D34,W205	D34,W205	D34,G39,W205	D34,G82,W205,E233	D34,G82,W205,E233	D34,G82,W205,E233

		**VDR**	12	11	12	12	14	15	21	22	24
	**40**	**HBR**	D34,W205	D34,W205	D34,C38,W205	D34,W205	G39,W205	D34,W205	D34,G82,W205,E233	D34,G82,W205,E233	D34,G82,W205,E233
		**VDR**	11	11	12	12	13	17	20	22	24
	**60**	**HBR**	W205	D34,W205	D34,C38,W205	D34,C38,W205	D34,G39,W205	D34,G39,W205	D34,G82,W205,E233	D34,G82,W205,E233	D34,G82,W205,E233
		**VDR**	12	14	13	13	13	16	21	24	26
	**80**	**HBR**	D34,W205	D34,W205	D34,C38,W205	D34,C38,W205	D34,W205	D34,W205	D34,G82,W205,E233	D34,G82,W205,E233	D34,G82,W205,E233
		**VDR**	11	12	12	12	13	16	19	23	22
	**100**	**HBR**	D34,W205	D34,W205	D34,C38,W205	D34,C38,W205	D34,G39,W205	D34,G39,W205	D34,G82,W205,E233	D34,G82,W205,E233	D34,G82,W205,E233
		**VDR**	13	13	12	12	12	17	22	24	21
**BP-2**	**0**	**HBR**	Q37,W207	Q37,W207	Q37,E82,W207	Q37,G84,W207	Q37,G84,W207	Q37,W207	Q37,G82,W205,E233	Q37,G82,W205,E233	Q37,G82,W205,E233
		**VDR**	11	7	13	13	14	17	23	22	22
	**20**	**HBR**	Q37,W207	W207	Q37,E82,W207	Q37,E82,W207	Q37,G84,W207	G84,W207	Q37,G82,W205,E233	Q37,G84,W205,E233	Q37,G82,W205,E233
		**VDR**	9	9	12	13	13	15	23	24	24
	**40**	**HBR**	Q37,207	Q37	Q37,E82,W207	Q37,W207	Q37,G84,W207	Q37,G84,W207	Q37,G82,W205,E233	Q37,G84,N174,E233	Q37,G84,W205,E233
		**VDR**	10	9	12	12	13	17	22	24	21
	**60**	**HBR**	Q37	Q37,W207	Q37,G84,W207	Q37,G84,W207	Q37,G84,W207	Q37,W207	Q37,G82,W205,E233	Q37,G84,N174,E233	Q37,G84,W205,E233
		**VDR**	11	10	13	13	13	16	24	23	11
	**80**	**HBR**	Q37,W207	Q37	W207	Q37,G84,W207	Q37,G84,W207	Q37,G84,W207	Q37,G82,W205,E233	Q37,G84,N174,E233	Q37,G84,W205,E233
		**VDR**	10	10	12	12	12	17	23	22	24
	**100**	**HBR**	Q37,W207	Q37,W207	G84,W207	Q37,G84,W207	Q37,G84,W207	Q37,G84,W207	Q37,G82,W205,E233	Q37,G84,N174,E233	Q37,G84,W205,E233
		**VDR**	12	9	14	14	13	16	24	22	23
**CP-2**	**0**	**HBR**	W207	Q37	Q37,W207	Q37,W207	C43,G84,N174	C43,G84,N174	Q37,E82,N174,W207	Q37,E82,N174,W207	Q37,E82,N174,W207
		**VDR**	11	8	12	12	13	17	22	23	25
	**20**	**HBR**	Q37,W207	Q37,C40	W207	Q37,W207	C43,G84,N174	C43,G84,N174	Q37,E82,N174,W207	Q37,E82,N174,W207	Q37,E82,N174,W207
		**VDR**	11	7	13	12	12	16	24	22	24
	**40**	**HBR**	Q37	Q37,W207	Q37,W207	-	H175	C43,G84,N174	Q37,E82,N174,W207	Q37,E82,N174,W207	Q37,E82,N174,W207
		**VDR**	12	11	12	13	13	16	23	21	22
	**60**	**HBR**	Q37,W207	Q37,C40	Q37,W207	Q37,W207	C43,G84,N174	C43,G84,N174	Q37,E82,N174,W207	Q37,E82,N174,W207	Q37,E82,N174,W207
		**VDR**	13	12	12	12	12	17	21	22	24
	**80**	**HBR**	Q37	Q37,W207	Q37	Q37,W207	C43,G84,N174	C43,G84,N174	Q37,E82,N174,W207	Q37,E82,N174,W207	Q37,E82,N174,W207
		**VDR**	9	10	13	12	13	18	22	22	23

	**100**	**HBR**	Q37,W207	Q37,W207	Q37	Q37,W207	C43,H175	C43,G84,N174	Q37,E82,N174,W207	Q37,E82,N174,W207	Q37,E82,N174,W207
		**VDR**	8	9	12	12	13	18	20	24	24
**YP-2**	**0**	**HBR**	Q37,W207	Q37,W207	Q37,G84,W207	Q37,G84,W207	Q37,G84,W207	Q37,W207	G84,N174,W207	G84,N174	G84
		**VDR**	11	10	12	12	12	17	23	24	22
	**20**	**HBR**	Q37	Q37	Q37,G84,W207	Q37,G84,W207	Q37,G84,W207	Q37,G84,W207	Q37,N174	G84,N174,W207	G84,N174,W207
		**VDR**	10	9	12	11	10	18	24	24	24
	**40**	**HBR**	W207	Q37,W207	Q37,W207	0	Q37,W207	Q37,W207	G84,N174,W207	N174	G84,N174,W207
		**VDR**	10	7	12	13	14	18	23	22	24
	**60**	**HBR**	Q37,W207	Q37,W207	Q37,G84,W207	Q37,G84,W207	Q37,G84,W207	Q37,G84,W207	G84,N174,W207	G84,N174,W207	N174
		**VDR**	11	9	12	12	13	17	21	22	22
	**80**	**HBR**	Q37,W207	Q37	Q37,W207	Q37,G84,W207	Q37,G84,W207	Q37,G84,W207	G84,N174	N174	G84,N174,W207
		**VDR**	12	10	12	12	12	16	21	22	22
	**100**	**HBR**	Q37,W207	Q37,W207	Q37,W207	0	Q37,W207	Q37,G84,W207	G84,N174,W207	G84,N174,W207	G84,N174,W207
		**VDR**	11	11	12	12	Q37,G84,W207	16	22	23	23

Table S5: Protein-ligand complex binding free energy terms in kJ/mol as determined by molecular mechanics Poisson-Boltzmann surface area (MM-PBSA) analysis. vdW=van der Waals forces, ele=electrostatics, PB=polar solvation energy, SASA=Soluble Accessible Surface Area, bind=binding free energy.

		**Compound**								
**Protein**	**Energy**	**SANC00364**	**SANC00365**	**SANC00367**	**SANC00369**	**SANC00371**	**SANC00372**	**126461286**	**126462623**	**126465495**
**Cat S**	ΔG_{vdW}	-90.7±0.3	-90.1±0.2	-117.4±0.2	-112.4±0.4	-112.8±0.2	-113.2±0.2	-134.8±0.2	-131.8±0.3	-132.1±0.2
	ΔE_{ele}	-1.1±0.2	-2.3±0.3	-9.5±0.3	-10.4±0.3	-10.3±0.2	-10.8±0.3	-14.9±0.3	-19.3±0.2	-17.3±0.2
	ΔG_{PB}	62.5±0.2	54.8±0.2	65.8±0.2	65.5±0.3	67.5±0.5	68.8±0.2	38.6±0.2	35.5±0.2	43.5±0.3
	ΔG_{SASA}	-6.1±0.3	-5.5±0.2	-7.4±0.3	-8.3±0.2	-10.3±0.4	-7.4±0.3	-13.6±0.2	-17.7±0.2	-15.9±0.2
	ΔG_{bind}	**-35.5±0.2**	**-43.1±0.2**	**-68.4±0.2**	**-65.6±0.2**	**-66.0±0.2**	**-62.6±0.2**	**-124.6±0.5**	**-133.4±0.2**	**-121.8±0.4**
**Cat K**	ΔG_{vdW}	-88.7±0.3	-90.4±0.2	-116.1±0.2	-112.1±0.2	-121.7±0.2	-121.1±0.4	-130.6±0.4	-136.9±0.2	-137.5±0.2
	ΔE_{ele}	-2.5±0.3	-3.7±0.2	-9.5±0.2	-7.9±0.2	-9.8±0.2	-9.7±0.2	-17.8±0.4	-17.8±0.2	-18.2±0.2
	ΔG_{PB}	68.0±0.2	55.5±0.2	70.8±0.2	64.0±0.3	63.6±0.2	70.0±0.2	43.4±0.2	36.5±0.2	49.5±0.2
	ΔG_{SASA}	-7.0±0.2	-6.2±0.4	-7.9±0.2	-8.2±0.2	-9.4±0.4	-8.9±0.2	-16.3±0.4	-16.3±0.2	-16.7±0.4
	ΔG_{bind}	**-30.2±0.4**	**-45.8±0.2**	**-62.7±0.2**	**-64.2±0.2**	**-77.3±0.2**	**-70.1±0.2**	**-121.3±0.2**	**-134.6±0.2**	**-122.9±0.2**
**Cat L**	ΔG_{vdW}	-90.5±0.3	-90.9±0.4	-116.1±0.2	-110.1±0.2	-117.9±0.2	-117.4±0.2	-132.9±0.3	-130.7±0.2	-134.2±0.2
	ΔE_{ele}	-2.3±0.3	-4.3±0.2	-8.5±0.4	6.6±0.2	-9.8±0.3	-12.6±0.2	-19.5±0.2	-19.7±0.3	-16.6±0.3
	ΔG_{PB}	63.5±0.2	64.6±0.4	67.6±0.5	69.7±0.2	70.5±0.2	63.5±0.2	45.4±0.2	37.5±0.2	46.5±0.2
	ΔG_{SASA}	-7.7±0.2	-5.8±0.2	-8.6±0.4	-9.4±0.2	-8.8±0.2	-9.8±0.2	-17.9±0.2	-18.1±0.2	-15.2±0.2
	ΔG_{bind}	**-37.1±0.2**	**-36.5±0.2**	**-65.6±0.5**	**-56.6±0.2**	**-65.9±0.2**	**-76.8±0.2**	**-124.1±0.2**	**-131.0±0.2**	**-119.6±0.2**
**FP-2**	ΔG_{vdW}	-90.0±0.2	-93.3±0.2	-113.4±0.2	-117.3±2.7	-126.5±0.2	-125.2±0.2	-137.9±0.2	-143.3±0.2	-140.6±0.2
	ΔE_{ele}	-4.9±0.2	-3.3±0.2	-8.5±0.3	-10.5±1.7	-9.9±0.3	-10.5±0.3	-17.4±0.3	-19.4±0.3	-17.3±0.3
	ΔG_{PB}	54.7±0.2	53.6±0.2	63.6±0.2	59.0±3.1	68.6±0.2	57.7±0.2	40.3±0.2	38.7±0.2	41.5±0.2
	ΔG_{SASA}	-6.7±0.2	-5.9±0.2	-8.8±0.3	-7.8±1.7	-7.3±0.3	-8.7±0.3	-16.0±0.3	-17.8±0.3	-15.9±0.3
	ΔG_{bind}	**-46.8±0.2**	**-49.0±0.2**	**-67.3±0.2**	**-76.6±2.7**	**-75.1±0.2**	**-86.7±0.2**	**-132.0±0.2**	**-141.9±0.2**	**-132.3±0.2**
**FP-3**	ΔG_{vdW}	-94.2±0.2	-91.5±0.2	-115.6±0.3	-113.6±0.2	-124.6±0.2	-129.3±0.6	-137.5±0.2	-142.1±0.2	-137.5±0.2
	ΔE_{ele}	-4.3±0.3	-3.5±0.2	-7.9±0.3	-9.7±0.3	-9.8±0.3	-9.9±0.3	-17.4±0.3	-19.4±0.3	-17.3±0.2
	ΔG_{PB}	57.9±0.2	54.9±0.2	68.3±0.2	60.6±0.2	63.5±0.4	64.6±0.4	42.5±0.4	34.9±0.2	43.5±0.2
	ΔG_{SASA}	-7.0±0.2	-6.4±0.2	-8.0±0.2	-7.8±0.2	-7.6±0.2	-8.7±0.4	-16.0±0.2	-17.8±0.2	-15.9±0.2
	ΔG_{bind}	**-47.6±0.2**	**-46.5±0.2**	**-63.0±0.2**	**-70.1±0.2**	**-78.5±0.2**	**-83.3±0.4**	**-128.4±0.2**	**-144.4±0.2**	**-127.2±0.2**
**VP-2**	ΔG_{vdW}	-87.4±0.2	-87..7±0.2	-112.2±0.2	-114.2±0.2	-130.0±0.4	-125.6±0.2	-144.2±0.2	-137.9±0.2	-137.5±0.2
	ΔE_{ele}	-5.8±0.3	-3.9±0.3	-5.6±0.3	-9.6±0.3	-10.5±0.4	-10.5±0.3	-18.4±0.3	-18.3±0.2	-18.0±0.3
	ΔG_{PB}	49.9±0.2	49.7±0.2	70.7±0.2	56.5±0.2	68.4±0.5	65.7±0.2	42.9±0.2	28.3±0.2	45.6±0.2
	ΔG_{SASA}	-5.8±0.2	-4.4±0.3	-9.4±0.2	-7.2±0.2	-7.0±0.4	-9.2±0.02	-16.9±0.2	-16.8±0.2	-16.5±0.2
	ΔG_{bind}	**-49.2±0.2**	**-46.4±0.2**	**-56.5±0.2**	**-73.4±0.2**	**-78.9±0.6**	**-79.5±0.2**	**-136.6±0.2**	**-144.2±0.2**	**-126.4±0.2**
**VP-3**	ΔG_{vdW}	-91.6±0.2	-87.7±0.2	-118.3±0.2	-114.7±0.2	-123.6±0.2	-126.0±0.2	-137.2±0.2	-138.6±0.2	-138.1±0.2

	**ΔE $_{ele}$**	-2.3±0.2	-2.5±0.3	-9.9±0.4	-10.6±0.2	-11.3±0.3	-11.3±0.2	-17.8±0.3	-17.6±0.3	-16.7±0.3
	**ΔG $_{PB}$**	58.9±0.2	53.3±0.2	65.6±0.5	48.8±0.2	63.9±0.2	64.9±0.5	38.7±0.2	30.5±0.2	47.5±0.2
	**ΔG $_{SASA}$**	-6.0±0.2	-5.4±0.2	-7.7±0.2	-8.3±0.3	-9.3±0.2	-8.9±0.2	-16.3±0.2	-16.2±0.3	-15.3±0.2
	**ΔG $_{bind}$**	**-41.1±0.2**	**-42.3±0.2**	**-70.4±0.5**	**-84.8±0.2**	**-80.3±0.2**	**-81.3±0.2**	**-132.7±0.2**	**-141.9±0.2**	**-122.6±0.2**
**KP-2**	**ΔG $_{vdW}$**	-87.6±0.2	-93.7±0.2	109.75±0.2	-113.7±0.5	-128.3±0.2	-123.7±0.2	-136.8±0.2	-137.0±0.2	-140.4±0.2
	**ΔE $_{ele}$**	-2.2±0.3	-3.0±0.2	-7.0±0.2	-9.6±0.2	8.9±0.3	-9.8±0.3	-18.4±0.3	-21.0±0.2	-15.6±0.3
	**ΔG $_{PB}$**	57.7±0.2	56.8±0.4	-64.8±0.4	56.6±0.5	64.6±0.2	64.6±0.2	46.5±0.2	34.6±0.2	46.5±0.2
	**ΔG $_{SASA}$**	-7.4±0.2	-5.5±0.2	-9.3±0.2	-8.6±0.4	-8.2±0.3	-9.2±0.2	-16.9±0.2	-19.3±0.2	-14.3±0.2
	**ΔG $_{bind}$**	**-39.7±0.2**	**-45.4±0.2**	**-61.2±0.4**	**-75.4±0.2**	**-80.8±0.2**	**-78.1±0.2**	**-125.6±0.2**	**-142.7±0.2**	**-123.7±0.2**
**KP-3**	**ΔG $_{vdW}$**	-89.2±0.2	-93.1±0.3	-112.4±0.2	-113.6±0.2	-127.1±0.2	-123.5±0.2	-136.0±0.2	-140.6±0.2	-142.2±0.2
	**ΔE $_{ele}$**	-3.5±0.2	-3.2±0.2	-7.9±0.2	-10.5±0.2	-9.9±0.2	-11.2±0.3	-18.4±0.3	-17.8±0.3	-18.7±0.3
	**ΔG $_{PB}$**	63.6±0.2	53.5±0.2	64.3±0.2	63.2±0.4	64.7±0.2	63.9±0.2	37.6±0.4	37.4±0.5	45.4±0.4
	**ΔG $_{SASA}$**	-5.8±0.2	-6.0±0.3	-8.6±0.3	-8.8±0.2	-8.2±0.3	-9.2±0.2	-16.7±0.2	-16.3±0.2	-17.2±0.2
	**ΔG $_{bind}$**	**-33.6±0.4**	**-49.0±0.2**	**-64.5±0.2**	**-69.7±0.2**	**-80.6±0.2**	**-80.0±0.2**	**-133.7±0.2**	**-137.3±0.2**	**-132.7±0.2**
**BP-2**	**ΔG $_{vdW}$**	-90.5±0.2	-93.3±0.2	-116.8±0.2	-116.1±0.5	-122.4±0.4	-126.8±0.4	-112±0.2	-148.3±0.2	-141.7±0.2
	**ΔE $_{ele}$**	-3.5±0.3	-2.4±0.2	-9.5±0.2	-9.8±0.2	-9.9±0.2	-10.4±0.2	-16.3±0.2	-17.4±0.3	-18.3±0.2
	**ΔG $_{PB}$**	68.7±0.2	54.3±0.4	62.5±0.2	56.9±0.2	64.6±0.5	62.4±0.2	45.6±0.5	37.4±0.2	48.7±0.2
	**ΔG $_{SASA}$**	-6.0±0.2	-5.6±0.3	-9.6±0.2	-9.4±0.2	-8.6±0.4	-8.2±0.2	-15.0±0.2	-16.0±0.2	-16.8±0.2
	**ΔG $_{bind}$**	**-31.3±0.2**	**-46.7±0.4**	**-73.4±0.4**	**-78.4±0.5**	**-76.3±0.5**	**-83.0±0.2**	**-125.1±0.4**	**-144.3±0.2**	**-128.1±0.2**
**CP-2**	**ΔG $_{vdW}$**	-91.5±0.2	-92.7±0.2	-118.0±0.3	-116.0±0.2	-129.8±0.2	-127.9±0.2	-141.4±0.2	-140.6±0.2	-143.5±0.2
	**ΔE $_{ele}$**	-4.2±0.3	-3.6±0.2	-9.3±0.2	-9.9±0.2	-9.8±0.3	-10.3±0.2	-21.7±0.4	-17.4±0.2	-19.5±0.2
	**ΔG $_{PB}$**	56.7±0.2	57.6±0.2	59.7±0.2	67.3±0.4	70.5±0.2	56.6±0.2	43.6±0.2	35.6±0.2	45.6±0.2
	**ΔG $_{SASA}$**	-5.4±0.2	-6.4±0.2	-7.2±0.3	-9.4±0.2	-10.0±0.2	-10.9±0.2	-20.0±0.2	-16.0±0.2	-17.9±0.2
	**ΔG $_{bind}$**	**-44.5±0.2**	**-45.1±0.2**	**-75.2±0.3**	**-68.0±0.2**	**-79.0±0.2**	**-92.6±0.2**	**-139.6±0.4**	**-138.4±0.2**	**-135.3±0.2**
**YP-2**	**ΔG $_{vdW}$**	-94.1±0.2	-88.9±0.2	-112.2±0.4	-115.8±0.2	-121.7±0.2	-121.1±0.2	-131.8±0.2	-143.0±0.2	-136.8±0.2
	**ΔE $_{ele}$**	-4.8±0.3	-2.9±0.2	-8.6±0.3	-10.3±0.3	-9.0±0.2	-10.6±0.2	-19.4±0.2	-18.5±0.3	-16.8±0.2
	**ΔG $_{PB}$**	60.5±0.2	58.4±0.4	65.7±0.2	64.5±0.2	64.4±0.2	63.6±0.2	39.6±0.4	36.6±0.2	41.6±0.2
	**ΔG $_{SASA}$**	-5.1±0.2	-6.2±0.2	-8.5±0.2	-8.6±0.3	-9.4±0.2	-9.7±0.2	-17.8±0.2	-17.0±0.2	-15.4±0.2
	**ΔG $_{bind}$**	**-43.4±0.2**	**-39.5±0.2**	**-63.6±0.2**	**-70.3±0.2**	**-77.7±0.2**	**-83.4±0.2**	**-129.5±0.2**	**-142.1±0.2**	**-127.6±0.2**

Table S6: Subsite residue composition information (details and position). Residue numbering based on the catalytic domain length of individual proteins as indicated in **Table S1**.

**Subsite**		**Protein**											
		**FP-2**	**FP-3**	**VP-2**	**VP-3**	**KP-2**	**KP-3**	**BP-2**	**CP-2**	**YP-2**	**Cat-K**	**Cat-L**	**Cat-S**
**S1**	a	Q36	Q38	Q37	Q36	Q37	Q35	Q37	Q37	Q37	Q19	Q20	Q19
	b	C39	C41	C40	C39	C40	C38	C40	C40	C40	C22	C23	C22
	c	G40	G42	G41	G40	G41	G39	A41	A41	A41	G23	G24	G23
	d	C80	C82	C81	C80	C81	C79	C81	C81	C81	C63	C66	C66
	e	N81	Y83	Y82	D81	D82	D80	E82	E82	D82	G64	N67	N67
**S2**	a	L84	Y86	F85	N84	L85	F83	I85	I85	I85	Y67	L70	F70
	b	I85	I87	I86	I85	I86	I84	L86	L86	L86	M68	M71	M71
	c	S149	S151	S150	S149	S150	N148	A150	A150	A150	A134	A136	G137
	d	L172	P174	P173	P172	P173	T171	A173	A173	A173	L160	M162	V162
	e	N173	N175	N174	N173	N174	N172	N174	N174	N174	N161	D163	N163
	f	A175	A177	A176	A175	A176	A174	A176	A176	A176	A163	G165	G165
	g	D234	E236	E235	Q234	E235	E233	G235	A235	G235	L209	A215	F211
**S3**	a	K76	K78	Q77	K76	K77	Q75	N77	N77	N77	E59	G62	G62
	b	N77	N79	N78	N77	N78	N76	N78	N78	N78	N60	N63	N63
	c	Y78	N80	T79	Y78	N79	N77	F79	D79	F79	D61	E64	K64
	d	G82	G83	G83	G82	G83	G81	G83	G83	G83	G65	G68	G68
	e	G83	G84	G84	G83	G84	G82	G84	G84	G84	G66	G69	G69
**S1'**	a	V150	I152	I151	I150	I151	I149	V15	V151	V151	I135	I137	V138
	b	A151	A153	A152	C151	N152	A150	G152	G152	G152	D136	D138	D139
	c	V152	A154	V153	A152	A153	V151	V153	A153	V153	A137	A139	A140
	d	S153	S155	S154	N153	N154	S152	D154	S154	A154	S138	G140	R141
	e	A157	A159	A158	V157	T158	A156	E158	Q158	E158	Q143	L145	F146
	f	H174	H176	H175	H174	H175	H173	H175	H175	H175	H162	H164	H164
	g	N204	N206	N205	N204	N205	N203	N205	N205	N205	N182	N188	N184
	h	W206	W208	W207	W206	W207	W205	W207	W207	W207	W184	W190	W186

Table S7. Residues with significant *ΔBC* (A) and *ΔL* (B). Apo systems were used as the reference structures. A cut off value of one and half times standard deviation of the means of the difference with the various ligands used to define residues with significant changes in *average BC* and *average L*. Residue numbering based on the catalytic domain length indicated in **Table S1**.

Protein	Change	Residues
**FP-2**	+ change	W24, L26, V30, T31, S41, A56, I57, N60, C73, G82, P105, S147, A151, M183, I202, K203
	- change	R25, N38, G40, P100, P145, V179, F181, I201, S205, A235
**FP-3**	+ change	W47, G51, S52, L66, S68, V73, S76, D96, S110, C116, N117, L118, I148, L180, V181, A237, P240
	- change	A39, G42, Q70, M94, G98, K128, S132, Y189, S207, G209
**VP-2**	+ change	P13, K14, A21, V49, Q70, A109, V149, 1A52, F182, G183, G212, D222, I223, N224, 2G25, P229, C230, G233
	- change	D19, E53, M71, I111, S148, F165, V180, K197, Y199, I202, V203, S206, W207
**VP-3**	+ change	H22, W24, T31, N38, L65, E69, E101, V107, D108, L113, A152, N178, E184, Y199, K203, Q219, D221
	- change	C42, E52, C99, T110, S147, F164, V179, E185, L202, S230
**KP-2**	+ change	L21, T32, Q55, P106, V135, E139, V180, A186, K197, R198, S206, Q220, T221, D231, I241
	- change	W25, V51, Y56, I141, D166, A176, L179, Y188, Y200, L226, T229, G233
**KP-3**	+ change	S40, S45, T46, V47, V70, D80, P85, V139, V147, I176, Y197, Y199, A234
	- change	G36, G39, C41, V50, A150, G168, Y186, R194, R196, Y198, G236
**BP-2**	+ change	K12, I31, G33, S42, A49, Y56, D73, C74, E113, I141, L144, I147, A176, G181, N205, Y216, T221, I240
	- change	D27, I32, D36, Q38, A41, V52, Q69, F90, S109, P112, P133, L179, R204, N229, P238
**CP-2**	+ change	F23, D24, I32, S42, W44, A45, D110, D135, V151, V180, Y201, I202, S208, K220, C230, V231
	- change	S9, S13, I31, K38, A49, I52, E139, A140, I141, T148, A150, F165, A226, N229, V233
**YP-2**	+ change	N9, N19, I34, A47, L66, S109, P133, T147, G152, V177, I180, S206, C230, L232, I239, I240
	- change	V51, V52, R59, S67, E91, E116, Q142, P146, T148, I149, T170, L179, R204, V231
**Cat-K**	+ change	Y12, C22, V31, N52, D61, G64, G80, C96, K103, C155, L165, A166, A180, K191, N199, K200
	- change	N18, C25, G32, N47, A71, M97, N161, N201, N202, A203, G205, L209
**Cat-L**	+ change	E64, M71, Q76, E87, T111, I137, F153, E160, D163, E177, K187, S189, A203, K204

	- change	T15, T32, L35, G37, L54, D56, L70, P91, S134, K148, G165, L167, E174, S175
**Cat-S**	+ change	T14, E35, V46, D85, P91, K93, S103, R106, A107, K183, H188, C206
	- change	G23, F28, S29, A30, A36, L38, L53, K104, A108, T159, W186, G207
**B)**		
**FP-2**	+ change	M2, A20, R25, L26-S28, D35-G40, K59, L62, G83, Y106-L113, C119, Q208-W210
	- change	N60, G82, G97, C114, D117
**FP-3**	+ change	D20, A39, G98, P107-C116, K119, D136
	- change	L66, Y227, A237
**VP-2**	+ change	K14-H20, G83, G84, Y107-P, F117-I119, G167, D222-R227
	- change	S75, E113, M114, T192
**VP-3**	+ change	G40, E106-E112, Y158, L163, F164, G166-C168, E185-Y198, S230
	- change	T31, S74, L113, C114
**KP-2**	+ change	F18-Y20, D82, L114, C115, Y188-S192, E196, G209, K210, L226, T229
	- change	L21, V64, T158, L164, F165, G167, S168, E235, A236
**KP-3**	+ change	C38, G39, N59, V62, G81, G82, L97, Y105, P110, C118
	- change	R27, E60, V109, K112, D116, R120, F133, F155, S188-K195
**BP-2**	+ change	I2-Y11, R26-Y28, I32, D36-K39, Y107-V111, N118, K119, Y188-L192
	- change	K17, N61, Q62, E113, V231
**CP-2**	+ change	S9, V18-Y20, I31, A33, Q37-R39, L114, S224-A226, N229
	- change	Y11, P14-D16, A30, A33, V34, S42, D110, N118, D135
**YP-2**	+ change	Y11, P14-Q17, I32, P112, E116, T170, Y172, V231
	- change	N19, D33, I34, D36-Q38, N61, L66, C81, N118-C120, H191, C230, L232, Q234
**Cat-K**	+ change	V16-Q19, G43, Q76, K77, M97, N99, G102, E115, G116, S154, K200-G205
	- change	G64, N156-D158
**Cat-L**	+ change	Y101, N102, K104, S159, D179, G211
	- change	G44, I47, G62, E64, G69, E93-E96, T111, F144, E160, D163, T176, E177
**Cat-S**	+ change	Y101-K104, T159, Q160
	- change	G20, S21, V46

Table S8. Association of the various protein dynamic residue network metrics for proteins bound to different compounds as determined by Pearson's correlation coefficient.

		SANC00							PubChem		
**Protein**	**Metric**	**364**	**365**	**367**	**369**	**371**	**372**	**373**	**126461286**	**126462623**	**126465495**
**FP-2**	***BC* vs** L^{-1}	0.71	0.71	0.71	0.72	0.71	0.70	0.70	0.72	0.70	0.70
	***BC* vs** $RMSF^{-1}$	0.67	0.67	0.68	0.63	0.65	0.66	0.65	0.65	0.63	0.61
	***L* vs RMSF**	0.76	0.68	0.78	0.64	0.74	0.77	0.70	0.73	0.77	0.76
**FP-3**	***BC* vs** L^{-1}	0.73	0.72	0.72	0.72	0.73	0.73	0.72	0.72	0.74	0.73
	***BC* vs** $RMSF^{-1}$	0.67	0.64	0.64	0.64	0.65	0.65	0.65	0.64	0.69	0.65
	***L* vs RMSF**	0.81	0.82	0.79	0.81	0.79	0.79	0.80	0.78	0.77	0.78
**VP-2**	***BC* vs** L^{-1}	0.73	0.74	0.75	0.74	0.75	0.73	0.73	0.73	0.74	0.74
	***BC* vs** $RMSF^{-1}$	0.61	0.60	0.69	0.62	0.61	0.61	0.65	0.65	0.63	0.59
	***L* vs RMSF**	0.77	0.75	0.76	0.73	0.77	0.74	0.80	0.77	0.74	0.75
**VP-3**	***BC* vs** L^{-1}	0.74	0.72	0.74	0.72	0.75	0.73	0.73	0.73	0.74	0.74
	***BC* vs** $RMSF^{-1}$	0.63	0.55	0.64	0.60	0.59	0.62	0.58	0.65	0.63	0.59
	***L* vs RMSF**	0.77	0.75	0.78	0.75	0.65	0.66	0.80	0.77	0.74	0.75
**KP-2**	***BC* vs** L^{-1}	0.74	0.77	0.73	0.72	0.75	0.77	0.77	0.78	0.77	0.73
	***BC* vs** $RMSF^{-1}$	0.57	0.62	0.64	0.64	0.59	0.66	0.64	0.63	0.63	0.64
	***L* vs RMSF**	0.75	0.72	0.78	0.78	0.76	0.70	0.72	0.77	0.68	0.80
**KP-3**	***BC* vs** L^{-1}	0.74	0.74	0.74	0.74	0.76	0.74	0.77	0.75	0.72	0.74
	***BC* vs** $RMSF^{-1}$	0.66	0.63	0.62	0.61	0.67	0.63	0.66	0.66	0.65	0.65
	***L* vs RMSF**	0.76	0.79	0.81	0.80	0.72	0.77	0.75	0.76	0.79	0.77
**BP-2**	***BC* vs** L^{-1}	0.76	0.77	0.77	0.76	0.76	0.77	0.76	0.77	0.77	0.76
	***BC* vs** $RMSF^{-1}$	0.59	0.60	0.62	0.58	0.61	0.55	0.56	0.56	0.55	0.53
	***L* vs RMSF**	0.76	0.71	0.73	0.75	0.75	0.72	0.71	0.69	0.68	0.72
**CP-2**	***BC* vs** L^{-1}	0.76	0.75	0.75	0.77	0.76	0.76	0.76	0.76	0.77	0.77
	***BC* vs** $RMSF^{-1}$	0.58	0.56	0.63	0.65	0.61	0.61	0.63	0.61	0.63	0.61
	***L* vs RMSF**	0.74	0.74	0.80	0.67	0.77	0.73	0.76	0.71	0.77	0.73
**YP-2**	***BC* vs** L^{-1}	0.75	0.75	0.76	0.76	0.75	0.78	0.75	0.75	0.75	0.76
	***BC* vs** $RMSF^{-1}$	0.65	0.55	0.62	0.60	0.57	0.65	0.61	0.68	0.64	0.54
	***L* vs RMSF**	0.76	0.73	0.75	0.73	0.72	0.61	0.71	0.76	0.75	0.73

**Cat-K**	***BC* vs *L*$^{-1}$**	0.86	0.86	0.87	0.87	0.86	0.87	0.87	0.88	0.87	0.86
	***BC* vs RMSF^{-1}**	0.61	0.66	0.62	0.64	0.59	0.64	0.66	0.61	0.68	0.64
	***L* vs RMSF**	0.53	0.66	0.62	0.61	0.58	0.56	0.56	0.49	0.57	0.64
**Cat-L**	***BC* vs *L*$^{-1}$**	0.83	0.84	0.83	0.83	0.84	0.84	0.86	0.82	0.84	0.84
	***BC* vs RMSF^{-1}**	0.62	0.52	0.65	0.59	0.65	0.65	0.60	0.59	0.63	0.62
	***L* vs RMSF**	0.69	0.54	0.70	0.63	0.66	0.67	0.68	0.68	0.69	0.72
**Cat-S**	***BC* vs *L*$^{-1}$**	0.86	0.86	0.87	0.87	0.87	0.87	0.87	0.86	0.87	0.84
	***BC* vs RMSF^{-1}**	0.52	0.59	0.54	0.58	0.59	0.58	0.62	0.60	0.59	0.54
	***L* vs RMSF**	0.51	0.58	0.54	0.62	0.62	0.56	0.61	0.59	0.52	0.65

Table S9. Active compounds against FP-2 and decoys from DUD-E used for docking protocol validation.

	ACTIVE		
**Nos**	**Smiles**	**IC50 [µm]**	**2D Structure**
1	CCCCOC(=O)C1C(Oc2c1c(\C=C\C(=O)OC(Cc1ccc(O)c(O)c1)C(O)=O)ccc2O)c1ccc(O)c(O)c1	3.18	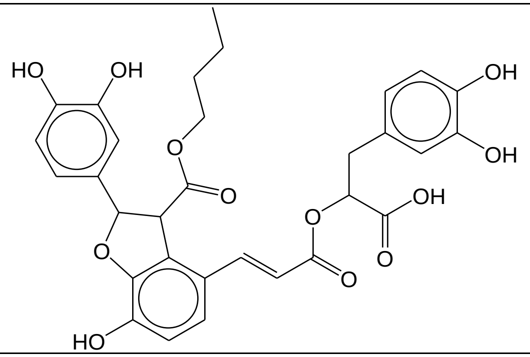
2	COC(=O)C(Cc1ccc(O)c(O)c1)OC(=O)\C=C\c1ccc(O)c2OC(C(C(=O)OC)c12)c1ccc(O)c(O)c1	3.77	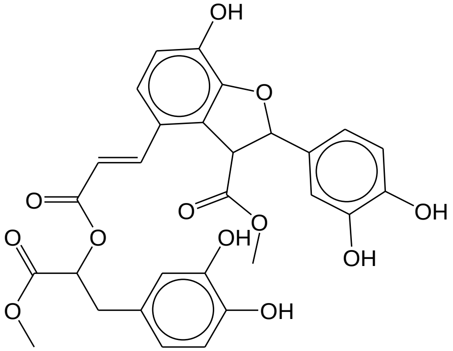

3	Oc1ccc(cc1)C1CCc2c(O)cc3OC4(Oc5cc(O)cc(O)c5C(=O)C4(O)c3c2O1)c1ccc(O)cc1	5.23	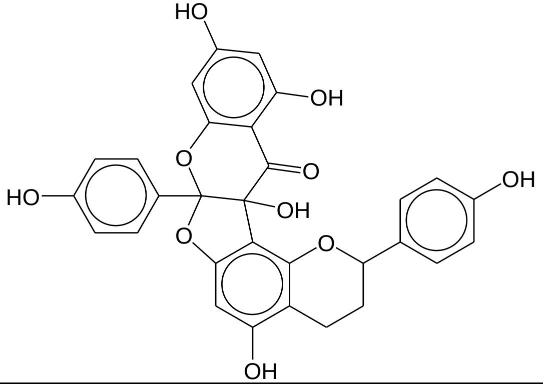
4	CCOC(=O)c1c(O)cc(O)c2CCC(Oc12)c1ccc(O)cc1	9.12	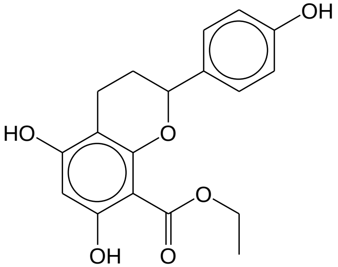
5	CC[C@H](C)[C@H](N(C)C)C(=O)N[C@@H](CC(C)C)C(=O)N[C@@H](CC(C)C)C(=O)N[C@@H](C)\C=C\C(=O)N[C@@H](C)C(=O)N1C[C@@H](O)C[C@H]1C(=O)OC	7.7	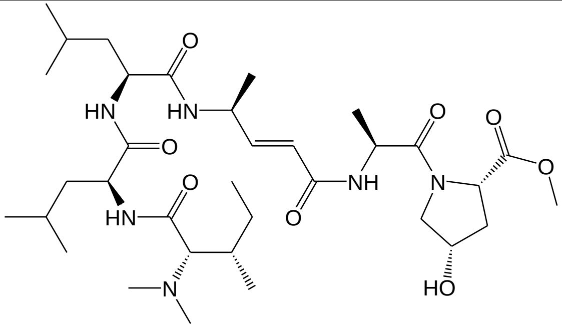

6	CC(C)C[C@H](NC(=O)[C@H](CC(C)C)NC(=O)[C@@H]1CCCN1C)C(=O)N[C@@H](C)\C=C\C(=O)N[C@@H](C)C(=O)NCc1ccccc1	6.0	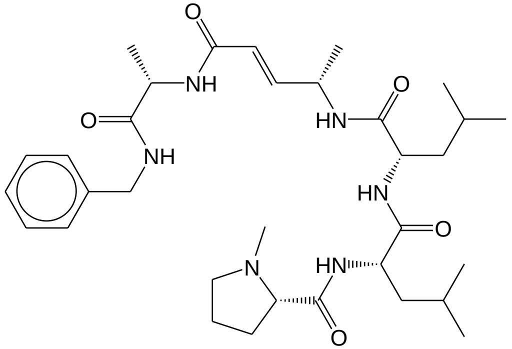
7	COC1=CC(=O)N(C1)C(=O)\C=C\[C@H](C)NC(=O)[C@H](CC(C)C)NC(=O)[C@H](CC(C)C)NC(=O)[C@H](C(C)C)N(C)C	0.02	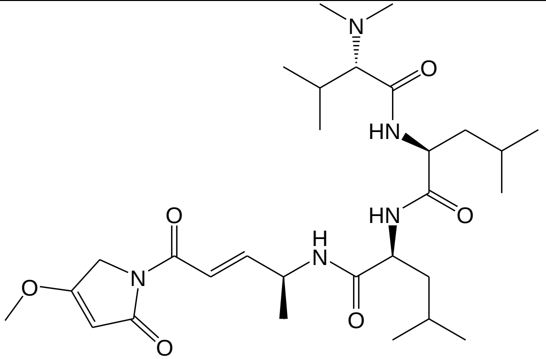
8	COC1=CC(=O)N([C@H]1C(C)C)C(=O)\C=C\[C@H](C)NC(=O)[C@H](CC(C)C)NC(=O)[C@H](CC(C)C)NC(=O)[C@H](C(C)C)N(C)C	0.009	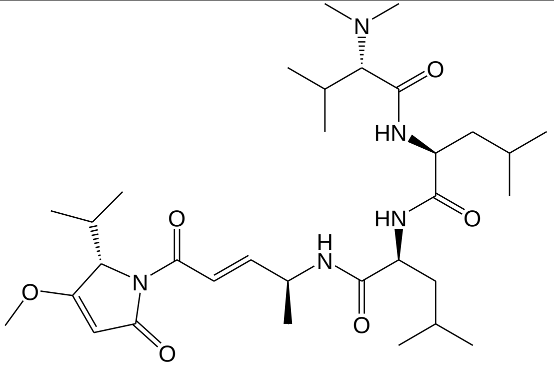

9	COC1=CC(=O)N([C@H]1Cc1ccc(O)cc1)C(=O)\C=C\[C@H](C)NC(=O)[C@H](CC(C)C)NC(=O)[C@H](CC(C)C)NC(=O)[C@H](C(C)C)N(C)C	0.005	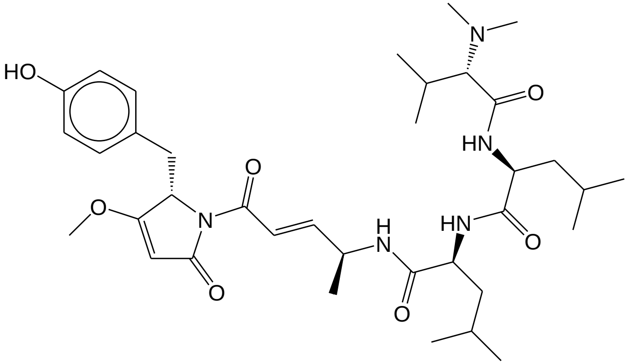
10	COC1=CC(=O)N([C@H]1Cc1c[nH]c2ccccc12)C(=O)\C=C\[C@H](C)NC(=O)[C@H](CC(C)C)NC(=O)[C@H](CC(C)C)NC(=O)[C@H](C(C)C)N(C)C	0.01	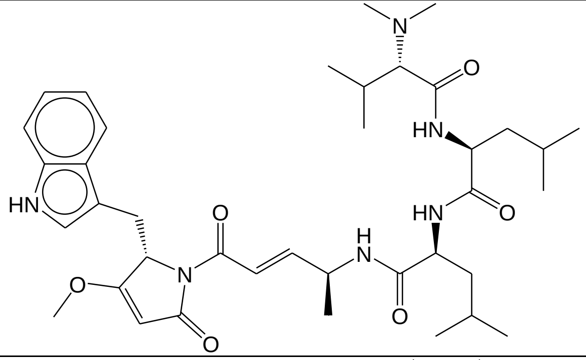
11	CC(C)C[C@H](NC(=O)[C@H](CC(C)C)NC(=O)[C@H](C(C)C)N(C)C)C(=O)N[C@@H](C)\C=C\C(=O)N1CC(OCc2ccccc2)=CC1=O	0.009	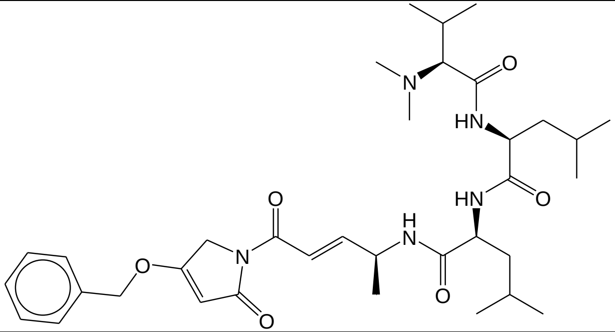

12	CC(C)C[C@H](NC(=O)[C@H](CC(C)C)NC(=O)[C@H](C(C)C)N(C)C)C(=O)N[C@@H](C)\C=C\C(=O)N1[C@@H](C(C)C)C(OCc2ccccc2)=CC1=O	0.006	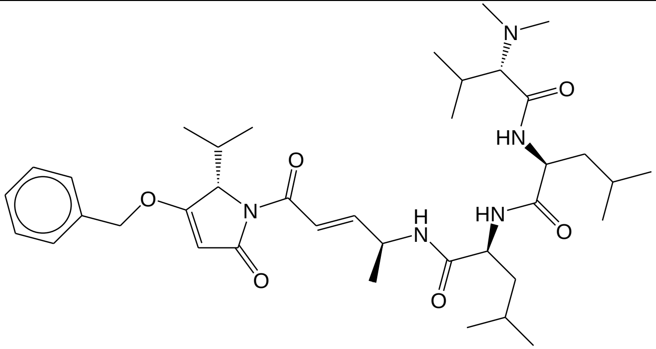
13	CC1CC(NCS(=O)(=O)c2ccccc2)C(=O)N(C\C=C\S(=O)(=O)c2ccccc2)C1	0.14	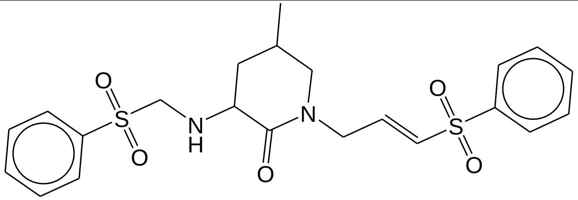
14	CC(C)CC(NCC(=O)N1CCN(C)CC1)C(=O)N[C@@H](CCc1ccccc1)\C=C\S(=O)(=O)NOCc1ccccc1	0.002	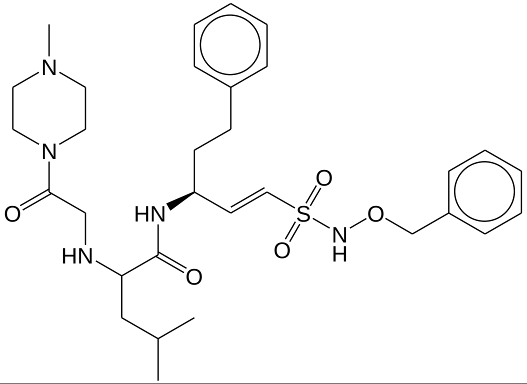

15	CC1CC(NCC(=O)NCCN2CCOCC2)C(=O)N(C1)[C@@H](CCc1ccccc1)\C=C\S(=O)(=O)NOCc1ccccc1	0.014	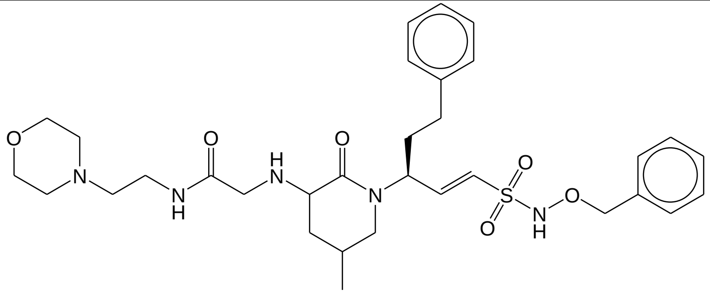
16	CC1CC(NCC(=O)NCCCCCO)C(=O)N(C1)[C@@H](CCc1ccccc1)\C=C\S(=O)(=O)NOCc1ccccc1	0.016	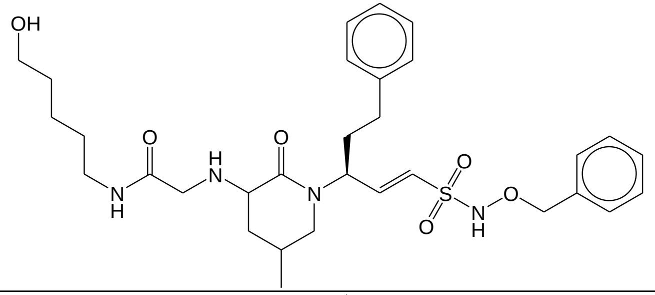
17	CCOC(=O)C1CCN(CC1)C(=O)CNC1CC(C)CN([C@@H](CCc2ccccc2)\C=C\S(=O)(=O)NOCc2ccccc2)C1=O	0.0022	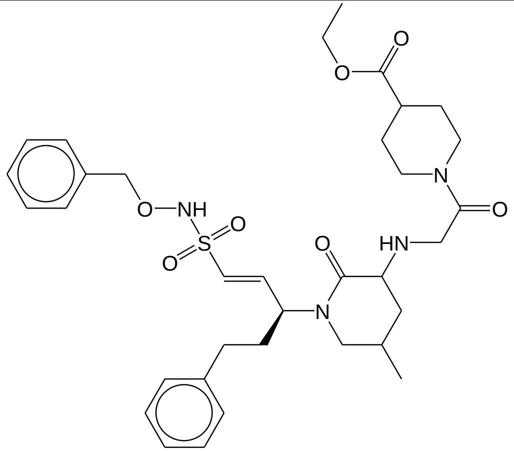

18	CC1CC(NCC(=O)NS(=O)(=O)c2ccccc2)C(=O)N(C1)[C@@H](CCc1ccccc1)\C=C\S(=O)(=O)NOCc1ccccc1	0.016	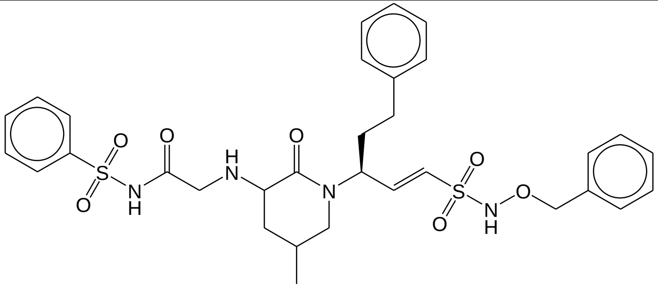
19	CC(C)CC(NC(=O)N1CCOCC1)C(=O)NC(CCc1ccccc1)\C=C\S(=O)(=O)Oc1ccc(F)cc1	0.0007	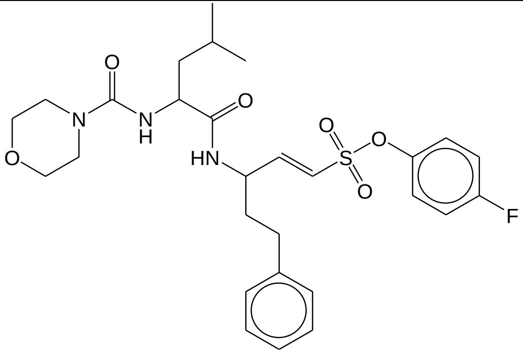
20	CC(C)CC(NC(=O)N1CCN(C)CC1)C(=O)NC(CCc1ccccc1)\C=C\S(=O)(=O)Oc1ccc(F)cc1	0.021	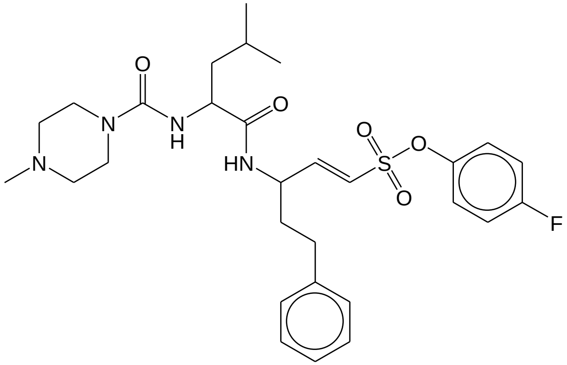

21	CCOC(=O)C1CCN(CC1)C(=O)NC(CC(C)C)C(=O)NC(CCc1ccccc1)\C=C\S(=O)(=O)Oc1ccc(F)cc1	0.0009	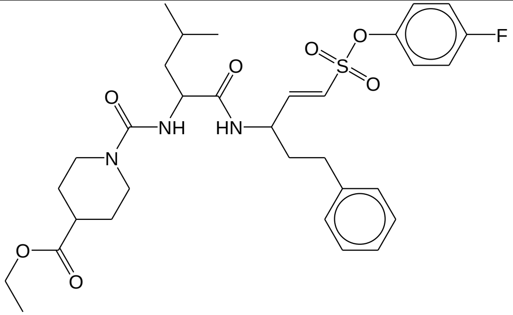
22	CC(C)CC(NC(=O)N1CCOCC1)C(=O)NC(CCc1ccccc1)\C=C\S(=O)(=O)Oc1ccccc1	0.0007	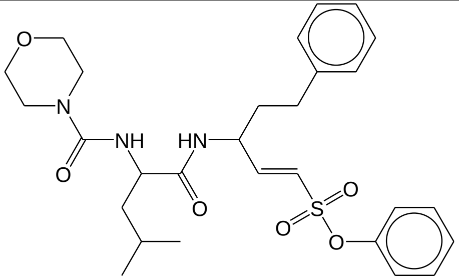
23	CC(C)CC(NC(=O)N1CCN(C)CC1)C(=O)NC(CCc1ccccc1)\C=C\S(=O)(=O)Oc1ccccc1	0.35	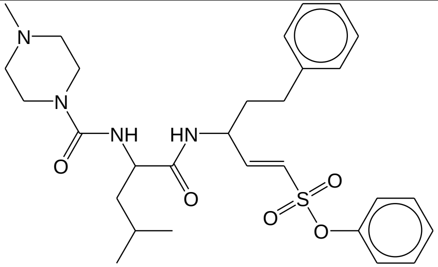
24	CCOC(=O)C1CCN(CC1)C(=O)NC(CC(C)C)C(=O)NC(CCc1ccccc1)\C=C\S(=O)(=O)Oc1ccccc1	0.0007	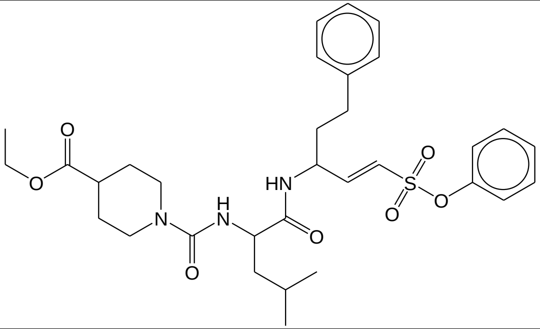

25	CC(C)CC(NC(=O)N1CCCC1CO)C(=O)NC(CCc1ccccc1)\C=C\S(=O)(=O)Oc1ccccc1	0.0008	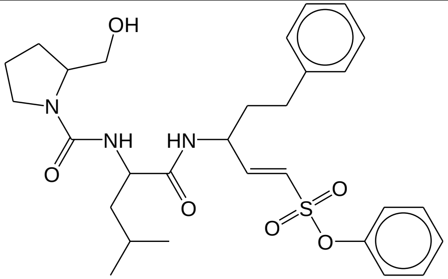
26	CC(C)CC(NC(=O)N1CCCC1CO)C(=O)NC(CCc1ccccc1)\C=C\S(=O)(=O)Oc1ccc(F)cc1	0.0009	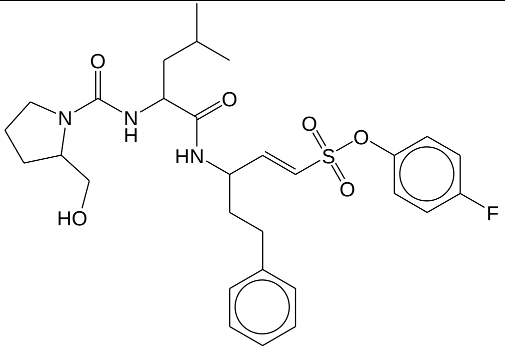
27	COc1ccc(OS(=O)(=O)\C=C\C(CCc2ccccc2)NC(=O)C(CC(C)C)NC(=O)N2CCCC2CO)cc1	0.0009	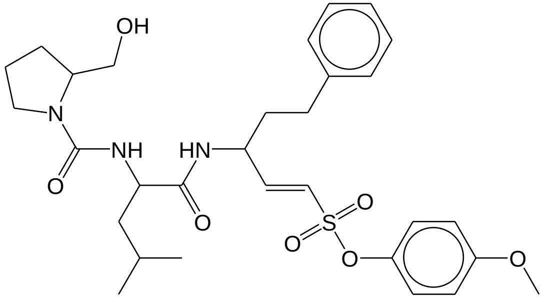
28	[O-][N+](=O)c1cccc(c1)C(=O)\C=C\c1ccc2OCOc2c1	8.5	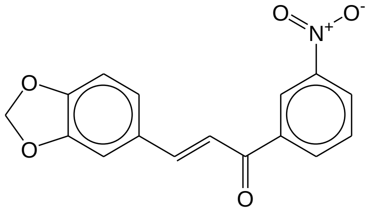

29	COc1cc(OC)c(\C=C\C(=O)c2cccc(c2)[N+]([O-])=O)cc1OC	9.5	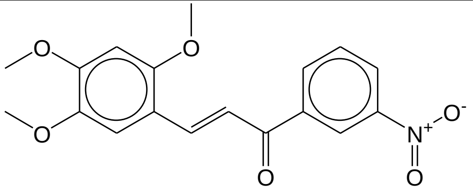
30	CC1CCc2c(C1)nc1ccc(cc1c2NCCCCCCNc1c2CCC(C)Cc2nc2ccc(cc12)[N+]([O-])=O)[N+]([O-])=O	5.2	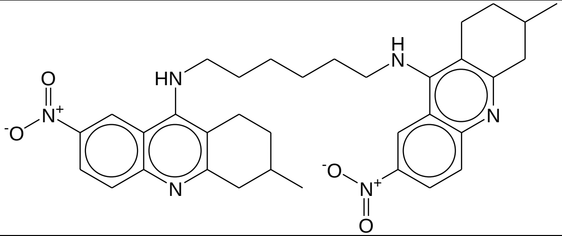
**DUD-E Decoys**			
1			
2	c1cc(ccc1C(=O)OCC(=O)c2ccc(cc2)Cl)NC(=O)CCCC(=O)OCC(=O)c3ccc(cc3)Cl		
3	COc1ccc(cc1OC)CN(CCC2=CCCCC2)S(=O)(=O)c3ccc(cc3)S(=O)(=O)NC4CCCCC4		
4	CCOC(=O)CSc1nnc(n1c2ccc(cc2)OC)CSc3[nH]c(c(n3)c4ccccc4)c5ccccc5		
5	CCOC(=O)c1c(c(sc1NC(=O)CSc2nc3c(c(cs3)c4ccco4)c(=O)n2CC=C)C(=O)OCC)C		
6	Cc1ccccc1OCc2nnc(n2C)SCC(=O)Nc3ccc4c(c3)sc(n4)SCC(=O)Nc5ccc(cc5)C(=O)OC		
7	CC1=NC(=C)C([C@@H]([C@@H]1C(=O)OC[C@H]2CCCO2)c3ccccc3[N+](=O)[O-])C(=O)OCCNC(=O)c4ccccc4O		
8	CC1=NC(=C)C([C@H](C1C(=O)OC[C@H]2CCCO2)c3ccccc3[N+](=O)[O-])C(=O)OCCNC(=O)c4ccccc4O		
9	CCOc1cc(ccc1OCCOc2ccc(cc2)Br)/C=N/Nc3cc(nc(n3)N4CCCC4)N5CCCC5		
10	Cc1cc(nc(n1)SCC(=O)N(Cc2ccc(cc2)F)[C@H](c3ccc(c(c3)OC)OC)C(=O)Nc4ccc(cc4)N(C)C)C		
11	CN(C)c1ccc(cc1)NC(=O)[C@@H](c2cc(c(c(c2)OC)OC)OC)N(Cc3ccccc3)C(=O)Cn4c5ccccc5nn4		
12	COCCOc1cc(c(cc1Cl)Cl)N(CC(=O)Nc2cccc(c2)C(F)(F)F)S(=O)(=O)c3ccc(cc3)OC		
13	COc1cc(cc(c1OC)OC)Nc2nc(nc(n2)OC(C(F)(F)F)C(F)(F)F)OC(C(F)(F)F)C(F)(F)F		
14	CCOc1ccc(cc1OCC)CCNS(=O)(=O)c2cc(ccc2C)C3=NN[C@@H](c4c3cccc4)Nc5ccc6c(c5)OCO6		
15	CC(C)(C)C(=O)OC[C@@H]1[C@H]([C@@H]([C@@H]([C@@H](O1)NC(=O)c2ccccn2)OC(=O)C(C)(C)C)OC(=O)C(C)(C)C)OC(=O)C(C)(C)C		
16	CCCCCCCCCCC[C@]12O[C@@H]3[C@@H]4C5[C@](O5)([C@H]([C@]6([C@@H]([C@@]4(O1)[C@@H](C[C@@]3(O2)C(=C)C)C)C=C(C6=O)C)O)O)CO		
17	CCSCCOC(=O)C1=C(NC2=C([C@@H]1c3ccc(c(c3OC)OC)OC)C(=O)C[C@@H](C2)c4ccccc4OC)C		
18	CCSCCOC(=O)C1=C(NC2=C([C@@H]1c3ccc(c(c3OC)OC)OC)C(=O)C[C@H](C2)c4ccccc4OC)C		
19	C[C@@H](C(=O)c1ccccc1)OC(=O)c2ccc(cc2)NC(=O)CCCC(=O)OCC(=O)c3ccc(cc3)Br		
20	CCOc1ccc(cc1)n2c(nnc2SCC(=O)Nc3ccc(cc3OC)OC)COc4ccc(cc4)Cl		
21	CCN(CC)c1ccc(cc1)NC(=O)CSc2nnc(n2c3ccc(cc3)OCC)COc4ccc(cc4)Cl		
22	CC(=O)Oc1c(cc(cc1OC)/C=N\NC(=O)CSc2nnc(s2)SCc3ccccc3Cl)OC		
23	COCCN(CCOC)S(=O)(=O)c1ccc(cc1)C(=O)Nc2nc(cs2)c3ccc(cc3)Oc4ccccc4		
	COc1ccc(cc1)CCCc2nnc(o2)SCC(=O)N/N=C/c3ccc(c(c3)OC)OCc4cccc5c4cccc5		

24	CCOC(=O)c1ccc(cc1)c2ccc(o2)/C=N/Nc3nc(nc(n3)Nc4ccc(cc4)OC)NCc5ccco5
25	CCCCN(CC)S(=O)(=O)c1ccc(cc1)C(=O)Nc2nnc(o2)c3cc(c(c(c3)OCC)OCC)OCC
26	CC[C@@H]1CCCCN1S(=O)(=O)c2ccc(cc2)C(=O)Nc3nnc(o3)c4cc(c(c(c4)OCC)OCC)OCC
27	CCOc1cc(ccc1OCC=C)[C@@H]2C(=C(C(=O)N2c3nnc(s3)SCc4ccccc4F)O)C(=O)c5ccc6c(c5)C[C@H](O6)C
28	CCCCCCCCCCCCSc1nc2c(n1C[C@H](COc3ccc(cc3)Cl)O)c(=O)[nH]c(=O)n2C
29	CC(C)(C)c1ccc(cc1)c2nnc(n2c3ccccc3)SCC(=O)Nc4ccc5c(c4)sc(n5)SCC(=O)NC[C@@H]6CCCO6
30	c1ccc(cc1)C(=O)Nc2c(cn(c(=O)n2)[C@H]3[C@H]([C@@H]([C@@H](O3)COC(=O)c4ccccc4)OC(=O)c5ccccc5)OC(=O)c6ccccc6)F
31	COc1ccc(cc1)NC(=O)CCCCn2c(=O)c3c(ccs3)nc2SCC(=O)Nc4ccc(cc4OC)OC
32	CCC(C)(C)[C@H]1CCc2c(sc(c2C(=O)OC)NC(=O)CCCS(=O)(=O)c3nc(cc(n3)C(F)(F)F)c4ccccc4OC)C1
33	Cc1ccc(cc1)Cn2ccc(n2)NC(=O)CCCS(=O)(=O)c3nc(cc(n3)C(F)(F)F)c4ccc(c(c4)OC)OC
34	CCCCOc1ccc(c(c1)C)c2c(cn(n2)c3ccccc3)C4C(=C(NC(=C4C(=O)OCCOC)C)C)C(=O)OCCOC
35	CCCOc1ccc(c(c1)C)c2c(cn(n2)c3ccccc3)C4C(=C(NC(=C4C(=O)OCCOC)C)C)C(=O)OCCOC
36	CC[NH+](CC)CCN1[C@H](C(=C(C1=O)[O-])C(=O)c2ccc(cc2)OCC=C)c3cc(c(c(c3)Br)O)OC
37	CCCOP(=O)(c1c(oc(n1)Cc2cccc3c2cccc3)NCCc4ccc(c(c4)OC)OC)OCCC
38	CCCOc1ccc(cc1)C(=O)Oc2ccc(cc2OC)/C=N/NC(=O)[C@H](C)Oc3ccc(cc3)Br
39	CCCOc1ccc(cc1)C(=O)Oc2ccc(cc2OCC)/C=N/NC(=O)C(=O)Nc3ccc(cc3)Br
40	CCOc1cc(ccc1O)/C=N/NC(=O)CSc2nnc(n2c3ccccc3)c4cc(c(c(c4)OC)OC)OC
41	CCCOc1ccc(cc1)C(=O)Oc2ccc(cc2OCC)/C=N/NC(=O)CNC(=O)c3ccc(c(c3)OC)OC
42	CCCOc1ccc(cc1)C(=O)Oc2ccc(cc2OCC)/C=N/NC(=O)C(=O)Nc3ccc(c(c3)Cl)Cl
43	CCCOc1ccc(cc1)C(=O)Oc2ccc(cc2OCC)/C=N/NC(=O)c3ccccc3NS(=O)(=O)c4ccc(cc4)Cl
44	Cc1ccc(cc1C)NC(=O)CN2c3ccccc3/C(=C/4\C(=O)N(C(=S)S4)CCCCCCCCCCC(=O)O)/C2=O
45	CCCCCCCCN1C(=O)/C(=C/2\c3ccccc3N(C2=O)CC(=O)Nc4ccc(cc4OC)OC)/SC1=S
46	CCCCOc1ccc(cc1)C(=O)NCC(=O)N/N=C/c2cc(ccc2OC(=O)c3ccc(cc3)C)Br
47	Cc1ccc(c(c1)c2c(cn(n2)c3ccccc3)C4C(=C(NC(=C4C(=O)OCCOC)C)C)C(=O)OCCOC)C
48	CCCOc1ccc(cc1)C(=O)Oc2ccc(cc2)/C=N/NC(=O)C(=O)Nc3ccccc3C(=O)Nc4cccc(c4)C
49	CC1=C(C(C(=C(N1)C)C(=O)OCC=C)c2cn(nc2c3ccc(c(c3)Cl)OC)c4ccccc4)C(=O)OCC=C
50	COc1ccc(cc1)c2nnc(n2c3ccc(cc3)OC)SCC(=O)N/N=C/c4ccc(c(c4OC)OC)OC
51	CCCN(CCC)S(=O)(=O)c1ccc(cc1)C(=O)Nc2ccc(cc2)S(=O)(=O)Nc3cc(on3)C
52	COc1ccc(cc1OC)c2nnc(n2CC=C)NCC(=O)Nc3ncc(s3)Cc4ccc(cc4)F
53	CCCCC(=O)Nc1nnc(s1)SCC(=O)Nc2c(c(c(s2)C(=O)OCC)C)C(=O)OCC
54	c1ccc(cc1)OCCOc2ccc(cc2)C(=O)NC(=S)Nc3cccc(c3)C(=O)NC4CCCCC4
55	Cn1c(nnc1SCC(=O)Nc2ccc(cc2)OC(F)(F)F)CSCC(=O)Nc3ccccc3
56	CC[C@@H](C)c1ccc(cc1)N([C@H](c2ccco2)C(=O)NC(C)(C)CC)C(=O)CNC(=O)c3cccs3
57	Cc1cc2cc(c(=O)[nH]c2cc1C)CN(Cc3cccnc3)C(=S)NCCc4ccc(c(c4)OC)OC
58	CCOCCn\1c2ccc(cc2s/c1=N\C(=O)CCCSc3ccc(cc3)OC)S(=O)(=O)N
59	CN(c1ccccc1)C(=O)c2cccc(c2)NC(=S)NC(=O)c3cccc(c3)OCCOc4ccccc4
60	c1ccc(cc1)C(=O)Nc2ccc(cc2)NC(=S)NC(=O)c3cccc(c3)OCCOc4ccccc4
	CCOc1cc(cc(c1OCC)OCC)C(=O)N/N=C(/C)\c2ccc(cc2)NC(=O)c3ccc(s3)C

61	CCc1ccc(c(c1)Br)OCC(=O)NNC(=S)NC(=O)COc2ccc(cc2)C(C)(C)C
62	Cc1cc(cc(c1Cl)C)OCC(=O)NNC(=S)NC(=O)CCCOc2ccc(cc2Cl)Cl
63	CCCOC(=O)c1ccc(cc1)OCC(=O)NNC(=S)NC(=O)c2ccc(c(c2)Br)C
64	Cc1cc(nc(n1)N/C(=N/C(=S)Nc2ccc(cc2)Nc3ccccc3)/NCc4ccc(cc4)OC)C
65	CCCOc1ccccc1C(=O)NNC(=S)NC(=O)c2ccc(c(c2)Br)OCC(C)C
66	CC[C@H](C)c1ccc(cc1)OCC(=O)NNC(=S)NC(=O)c2cc(ccc2OCC)Br
67	Cc1ccc(c(c1)C)NS(=O)(=O)c2ccc(cc2)NC(=S)NC(=O)c3ccccc3OCCOC
68	Cc1cccc(c1NS(=O)(=O)c2ccc(cc2)NC(=S)NC(=O)c3ccccc3OCCOC)C
69	COCCOc1ccc(cc1Br)C(=O)NC(=S)Nc2ccc(cc2)OC[C@H]3CCCO3
70	Cc1ccc(c(c1)C)OCC(=O)NNC(=S)NC(=O)c2cc(ccc2OCCOC)Br
71	c1cc(cc(c1)NC(=S)NC(=O)CCCOc2ccc(cc2Cl)Cl)C(=O)NC[C@H]3CCCO3
72	CC1=C([C@@H](C(=C(N1)C)C(=O)OCCOc2ccc(cc2)NC(=O)C)c3ccc(s3)[N+](=O)[O-])C(=O)OC
73	CCCOc1ccc(cc1)C(=O)NC(=S)Nc2ccc(cc2)NC(=O)[C@@H](C)Oc3ccccc3Cl
74	CCCSc1nc2ccc(cc2s1)NC(=O)CSc3nc([nH]n3)N/N=C/C=C/c4ccccc4
75	CCOc1ccc2c(c1)sc(n2)NC(=O)CSc3nc([nH]n3)N/N=C/c4ccc(cc4)C(=O)OC
76	CCCCSc1nnc(s1)NC(=O)CSc2nnc(n2N)N/N=C/c3cccc4c3cccc4
77	C=CCc1cccc(c1O)/C=N/Nc2[nH]nc(n2)SCCC(=O)NC(c3ccccc3)c4ccccc4
78	CCOC(=O)C1=C(NC(=O)N[C@H]1c2ccc(o2)C)COC(=O)CCc3ncc(o3)c4c(cccc4F)F
79	CCCCNC(=O)[C@@H](c1c(cccc1Cl)Cl)N(Cc2ccco2)C(=O)CNC(=O)c3ccccc3
80	CC[C@H](C)NC(=O)Cn1c2ccsc2c(=O)n(c1=O)CCCCCC(=O)NCc3ccccc3Cl
81	CCCOc1ccc(cc1OCC)[C@@H]2c3ccc(cc3OC(=C2C#N)N)OC(=O)COc4ccc(cc4)C
82	CCOc1ccc(cc1)n2c(nnc2SCC(=O)N/N=C/c3cccc(c3O)CC=C)c4ccccc4
83	CCCCCCOc1ccc(cc1)NC(=O)C[C@@H]2C(=O)N(C(=S)N2NC(=O)c3ccc(cc3)Cl)C
84	CCCCSc1ccccc1NC(=O)CSc2nc([nH]n2)N/N=C/c3cccc(c3)Br
85	CN(C)c1ccc(cc1)/C=N/Nc2[nH]nc(n2)SCC(=O)Nc3ccccc3SCc4ccccc4
86	CCCCSc1nnc(s1)NC(=O)CSc2nc([nH]n2)N/N=C/c3ccc(cc3Cl)Cl
87	CCCCSc1ccccc1NC(=O)CSc2nc([nH]n2)N/N=C/c3ccc(cc3)Br
88	COc1ccccc1/C=N/Nc2[nH]nc(n2)SCC(=O)Nc3ccc4c(c3)sc(n4)SCC=C
89	CCOC(=O)c1c(c(c(s1)NC(=O)CSc2nc([nH]n2)N/N=C(/C)\c3ccc(cc3)OC)C#N)C
90	Cc1ccccc1C(=O)NCCc2nnc(n2CC=C)SCC(=O)Nc3nc(cs3)c4ccccc4
91	COC(=O)c1ccccc1NC(=O)CSc2nnc(n2CC=C)CCNC(=O)c3ccccc3Cl
92	CCN(CC)C(=O)c1c(c(c(s1)NC(=S)Nc2ccn(n2)Cc3ccccc3Cl)C(=O)OC)C
93	CCN(CC)C(=O)c1c(c(c(s1)NC(=S)Nc2cccc(c2)Cn3c(cc(n3)C)C)C(=O)OC)C
94	CCN(CC)C(=O)c1c(c(c(s1)NC(=S)Nc2c(cn(n2)Cc3ccccc3)Cl)C(=O)OC)C
95	CCOc1ccccc1NC(=O)CCSc2nnc(n2c3ccccc3)CNc4cccc5c4cccc5
96	CCOc1ccccc1NC(=O)CCSc2nnc(n2c3ccccc3)CNc4ccc(cc4)Cl
97	CCc1ccccc1N(CC(=O)N/N=C/c2ccc(cc2)OCC(=O)Nc3ccc(cc3)C)S(=O)(=O)C
	CCCCOC(=O)c1ccc(cc1)NC(=O)C[C@@H]2C(=O)Nc3n2nc(c3c4ccc(cc4)Cl)COC

98	Cc1ccc(cc1)CN(Cc2ccc(o2)/C=N/NC(=O)C(=O)NCC=C)S(=O)(=O)c3ccc(cc3)C
99	C[C@@H](C(=O)Nc1ccc(cc1)C(=O)C)Sc2nnc(n2c3ccc(cc3)OC)CNc4ccc(cc4)OC
100	C[C@H](C(=O)Nc1ccc(cc1)C(=O)C)Sc2nnc(n2c3ccc(cc3)OC)CNc4ccc(cc4)OC
101	Cc1ccc(cc1C)NC(=O)Cn2c3ccsc3c(=O)n(c2=O)CCCCCC(=O)NCc4ccc(cc4)F
102	Cc1ccc(cc1NC(=O)Cn2c3ccccc3c(=O)n(c2=O)CCCCC(=O)NCc4ccccc4OC)Cl
103	CN(C)c1ccc(cc1)NC(=O)CSC2=Nc3ccccc3C4=N[C@H](C(=O)N42)CCC(=O)NCc5ccccc5OC
104	CCOCCOc1ccc(cc1)C(=O)NC(=S)Nc2ccc(cc2)S(=O)(=O)Nc3cc(cc(c3)C)C
105	CCOCCOc1cccc(c1)C(=O)NC(=S)Nc2ccc(cc2)S(=O)(=O)Nc3ccc(c(c3)C)C
106	CCc1ccc2c(c1)cc(c(=O)[nH]2)CN(CCc3ccc(c(c3)OC)OC)C(=S)Nc4ccc(cc4)OC
107	CCC(C)(C)NC(=O)[C@H](c1ccc(cc1)OC)N(c2ccc(cc2C)C)C(=O)CCC(=O)Nc3cc(on3)C
108	CCC(C)(C)NC(=O)[C@H](c1ccc(cc1)OC)N(Cc2ccc(cc2)C)C(=O)CCC(=O)Nc3cc(on3)C
109	CCC(C)(C)NC(=O)[C@H](c1ccccc1Cl)N(Cc2cccs2)C(=O)CCC(=O)Nc3cc(on3)C
110	Cc1ccc2cc(c(=O)[nH]c2c1)CN(Cc3ccc(cc3)OC)C(=S)NCCc4ccc(c(c4)OC)OC
111	CC[C@@H](C)Oc1ccc(cc1)C(=O)NC(=S)Nc2ccc(cc2)NC(=O)[C@@H](C)Oc3ccccc3Cl
112	CC[C@H](C)Oc1ccc(cc1)C(=O)NC(=S)Nc2ccc(cc2)NC(=O)[C@@H](C)Oc3ccccc3Cl
113	C=CCn1c(nnc1SCCC(=O)Nc2ccc(cc2)S(=O)(=O)NC3CCCCC3)c4cccnc4
114	CCOC(=O)CCCCC(=O)c1c([nH]c(=O)[nH]1)CSc2nc3c(c4cc(ccc4n3C)Br)nn2
115	CCOc1cc(ccc1OC(=O)c2ccco2)/C=N/NC(=O)/C(=C\c3cccs3)/NC(=O)c4ccccc4
116	CCCCCCNC(=O)Cc1csc(n1)NC(=O)CN(CC(C)C)C(=O)c2ccc(cc2Cl)Cl
117	CC[C@@H](C)C(=O)O[C@@H]1C[C@@H]2[C@@]([C@@H]([C@@H]([C@@H]([C@@]23[C@@H](O[C@@H](C3=C1)OC(=O)C)OC(=O)C)O)O)C)(C)CCC<br>(=C)C=C
118	CCCOc1ccc(cc1OCC)[C@@H]2c3ccc(cc3OC(=C2C#N)N)OC(=O)COc4cc(cc(c4)C)C
119	CCCCOc1ccc(cc1OCC)[C@@H]2c3c(n[nH]c3C(=O)N2CCCOC)c4cc(c(cc4O)C)Cl
120	Cc1ccc2cc(c(=O)[nH]c2c1C)CN(Cc3ccc(cc3)OC)C(=S)NCCc4ccc(c(c4)OC)OC
121	CCOc1ccc(cc1)n2c(nnc2SCC(=O)NNC(=C)c3cccc(c3)OC)c4ccc(cc4)Cl
122	CCOc1ccc(cc1)n2c(nnc2SCC(=O)N/N=C/c3cccc(c3O)OCC)c4ccc(cc4)Cl
123	COC(=O)[C@@](c1ccc(cc1)N[C@@](C(=O)OC)(C(F)(F)F)NC(=O)c2ccc(cc2Cl)Cl)(C(F)(F)F)O
124	CC(C)(C)c1ccc(cc1)OCCOc2c(cc(cc2Br)/C=N/NC(=O)Cc3nnc(s3)N)OC
125	CCOc1ccc(cc1)n2c(nnc2SCC(=O)NNC(=C)c3ccc(c(c3)OC)O)c4ccc(cc4)Cl
126	CCCOc1cccc(c1)C(=O)NC(=S)Nc2cc3c(cc2C)nn(n3)c4ccc(cc4C)[NH+](CC)CC
127	CC(C)c1ccc(c(c1)Br)OCC(=O)NNC(=S)NC(=O)c2cc(c(c(c2)OC)OC)OC
128	CCn1c(nnc1SCC(=O)Nc2cccc(c2)SC)[C@@H](CC(C)C)NC(=O)c3ccc(cc3Cl)Cl
129	CCn1c(nnc1SCC(=O)Nc2cccc(c2)C(=O)OC(C)C)CC(=O)Nc3ccc(c(c3)Cl)Cl
130	Cc1c(nc(s1)NC(=O)CSc2nnc(n2CC=C)CC(=O)Nc3cccc(c3)OC)c4ccccc4
131	COc1cc(cc(c1)OC)NC(=S)N[C@H]2CO[C@H]3[C@@H]2OC[C@H]3OC(=O)Nc4ccc(cc4)Oc5ccccc5
132	CCCCCOc1ccc(cc1OCC)[C@@H]2c3c([nH]nc3C(=O)N2CCCOC)c4cc(ccc4O)Cl
133	c1ccc(c(c1)C(F)(F)F)NC(=S)NNC(=O)CSCC(=O)Nc2ccc(cc2)OC(F)(F)F
134	C[C@H](c1ccc2ccccc2c1)NC(=O)CN(CCn3cccn3)CC(=O)N[C@@H](C)c4ccc5ccccc5c4

135	C[C@H](c1ccc2ccccc2c1)NC(=O)CN(CCn3cccn3)CC(=O)N[C@H](C)c4ccc5ccccc5c4
136	Cc1ccc(cc1C)c2csc3c2c(=O)[nH]c(n3)CN(Cc4ccco4)C[C@H](COCc5ccccc5)O
137	CCSCCOC(=O)C1=C(NC2=C([C@@H]1c3cc(c(c(c3)OC)O)[N+](=O)[O-])C(=O)[C@H]([C@@H](C2)C)C(=O)OC)C
138	COC(=O)Cc1c(c(=O)n([nH]1)c2ccccc2)[C@@H](c3ccc(cc3Cl)Cl)c4c(nn(c4O)c5ccccc5)CC(=O)OC
139	CC[NH+](CC)CCCN1[C@@H](C(=C(C1=O)[O-])C(=O)c2c(c([nH]c2C)C(=O)OC)C)c3ccc(cc3)Br
140	CCOc1ccc(cc1)NC(=O)CSc2nnc(n2c3ccccc3OC)CNC(=O)c4ccccc4F
141	CCSc1nnc(s1)NC(=O)CSc2nnc(n2c3cccc(c3)C(F)(F)F)CNC(=O)c4cccc(c4)C
142	Cc1ccc(c(c1)n2c(nnc2SCC(=O)Nc3ccc(cc3)OC)CNC(=O)c4ccc(cc4)S(=O)(=O)N(C)C)C
143	COc1ccc(cc1)NC(=O)CSc2nnc(n2c3cc(ccc3OC)OC)CNC(=O)c4cccs4
144	Cc1ccc(cc1)[C@@H]2C(=C(NC(=C2C(=O)OCC=C)C)SCC(=O)Nc3cc(c(cc3OC)Cl)OC)C#N
145	Cc1ccc(cc1)[C@H]2C(=C(NC(=C2C(=O)OCC=C)C)SCC(=O)Nc3cc(c(cc3OC)Cl)OC)C#N
146	c1cc(cc(c1)F)C(=O)Nc2ccc(cc2)OCCOCCOc3ccc(cc3)NC(=O)c4cccc(c4)F
147	CCOc1ccc(cc1)NC(=O)CSc2nnc(n2c3cccc(c3)C)CNC(=O)c4cccc(c4C)[N+](=O)[O-]
148	CCOc1ccc(cc1)C(=O)Oc2ccc(cc2/C=N/NC(=O)CNC(=O)c3ccc(cc3)OC)Br
149	C[C@@]12CC[C@H]([C@@]([C@@H]1CC[C@H]([C@H]2CC(=O)NCc3ccc4c(c3)OCO4)O)(C)CO)OC(=O)Nc5cc(c(c(c5)OC)OC)OC
150	CCOc1ccc(cc1)N(CC(=O)Nc2ccc(cc2)NC(=O)C)S(=O)(=O)c3ccc(c(c3)OC)OC
151	Cc1ccc(o1)c2csc(n2)NC(=O)CCCCCCCCC(=O)Nc3nc(cs3)c4ccc(o4)C
152	CC(C)(C)c1ccc(cc1)C(=O)Nc2ccc(cc2)C(=O)NNC(=S)NC(=O)CCC(=O)OCCOC
153	Cc1ccc(c(c1)NC(=O)CCC(=O)NNC(=S)NC(=O)CCCOc2ccc(cc2Cl)Cl)C
154	CCCCCCOc1ccc(cc1)OCCOc2ccc(cc2OC)/C=N/NC(=O)Cc3nnc(s3)N
155	CC(C)c1ccc(c(c1)Br)OCC(=O)NNC(=S)NC(=O)c2ccc(cc2)OCCOC
156	CC(C)c1ccc(cc1)OCC(=O)NNC(=S)NC(=O)c2cc(ccc2OCCOC)Br
157	COc1ccccc1NCc2nnc(n2c3ccccc3)SCCC(=O)Nc4ccc(c(c4)OC)Cl
158	CCCN1C(=C([C@@H](NC1=O)c2ccc(cc2)NC(=O)Nc3cc(cc(c3)OC)OC)C(=O)OCC(C)C)C
159	CCCN1C(=C([C@H](NC1=O)c2ccc(cc2)NC(=O)Nc3cc(cc(c3)OC)OC)C(=O)OCC(C)C)C
160	Cc1ccc(o1)CN([C@@H](c2c(cc(cc2O)O)O)C(=O)NC(C)(C)CC(C)(C)C)C(=O)CCC3CCCCC3
161	CCc1ccc(cc1)NC(=O)Cn2c3ccccc3c(=O)n(c2=O)CCCC(=O)NCc4ccccc4OC
162	CCCOc1ccc(cc1)C(=O)Oc2ccc3c(c2)OC(=C([C@H]3c4ccc(c(c4)OC)OCC(C)C)C#N)N
163	CC(C)COc1ccc(cc1OC)[C@@H]2c3ccc(cc3OC(=C2C#N)N)OC(=O)c4ccc(cc4)OCC=C
164	C[C@H](CNC(=O)c1ccc(cc1)C(=O)N(C(C)C)C(C)C)NC(=O)c2ccc(cc2)C(=O)N(C(C)C)C(C)C
165	CCCCCCOc1ccc(cc1OC)[C@H]2c3c(n[nH]c3C(=O)N2Cc4ccco4)c5cc(cc(c5O)C)C
166	CCCCCCOc1ccc(cc1OC)[C@H]2c3c(n[nH]c3C(=O)N2C[C@@H]4CCCO4)c5c(cc(cc5O)C)C
167	CCCOc1ccc(cc1OC)[C@H]2C(=C(Nc3n2nc(n3)SCCC)C)C(=O)Nc4ccccc4OC
168	CCOc1ccc(cc1)/N=C(/Nc2[nH]c(cc(=O)n2)CSc3nnnn3c4ccccc4)\NC(=O)c5ccccc5
169	CCOc1cc(ccc1OCc2ccccc2Cl)/C=N/NC(=O)Cc3nnc(s3)NC(=O)c4ccccc4C
170	CCCCOc1ccc(cc1OCC)[C@@H]2C(=C(Nc3n2nc(n3)SCC)C)C(=O)Nc4cccc(c4C)C
171	CCCCSc1nc2ccc(cc2s1)NC(=O)CSc3nc([nH]n3)N/N=C(/CC)\c4ccccc4
	CCOc1ccc(cc1OCC)CC/C(=N\O[C@@](C(=O)OC)(C(F)(F)F)NC(=O)c2ccc(cc2)C)/N

172	CCN(CC)c1ccc(cc1)n2nc3cc(c(cc3n2)NC(=S)NC(=O)c4cccc(c4)OCC(C)C)C
173	CCOc1ccc(cc1)NCc2nnc(n2c3ccccc3)SCC(=O)Nc4ccc(c(c4)Cl)OC
174	CCOc1ccc(cc1)NC(=O)CSc2nnc(n2CC=C)CNC(=O)c3ccccc3Br
175	CN(C)S(=O)(=O)N(CC(=O)Nc1ccc(cc1)S(=O)(=O)Nc2ccc(cc2OC)OC)c3ccccc3
176	Cc1ccc(cc1)S(=O)(=O)N(CC(=O)Nc2ccc(cc2)S(=O)(=O)Nc3c(cccc3C)C)c4cc(ccc4OC)OC
177	CC[C@H](C)C(=O)O[C@@H]1C[C@@H]2[C@@]([C@@H]([C@@H]([C@@H]([C@@]23[C@@H](O[C@@H](C3=C1)OC(=O)C)OC(=O)C)O)O)C)(C)C/C=C(
178	\C)/C=C
179	CCOc1cc(ccc1OCCC(C)C)[C@H]2c3c([nH]nc3C(=O)N2CCCOC)c4cc(ccc4O)Cl
180	CCOc1cc(ccc1OCC=C)[C@@H]2c3c([nH]nc3C(=O)N2CCCOC(C)C)c4cc(ccc4O)Cl
181	CC(C)(C(=O)O[C@H]1[C@H]([C@@H]([C@@H]([C@H](O1)C(=O)OCC=C)O)O)O)Oc2ccc(cc2)C(=O)c3ccc(cc3)Cl
182	Cc1cnc(nc1Nc2cccc(c2)S(=O)(=O)NC(C)(C)C)Nc3ccc(cc3)OCCN4CCCC4
183	CC(C)OC[C@H](CN(Cc1ccccc1)Cc2[nH]c(=O)c3c(csc3n2)c4ccc(c(c4)OC)OC)O
184	CCN(CC)CCNC(=O)/C(=C\c1cn(c2c1cccc2)S(=O)(=O)N(C)C)/NC(=O)c3ccccc3F
185	c1ccc(cc1)c2ccc(cc2)OCC(=O)NNC(=S)NC(=O)c3ccccc3OCCOc4ccccc4
186	CCOc1cc(cc(c1OCC)OCC)C(=O)Nc2nnc(s2)SCC(=O)Nc3ccc(cc3C)C
187	c1cc(c(cc1/C=N/Nc2c3c([nH]cn3)ncn2)OC([C@@H](C(F)(F)F)F)(F)F)OC([C@@H](C(F)(F)F)F)(F)F
188	CC[C@H](C(=O)Nc1cccc(c1)C(=O)C)Sc2nnc(n2CC)CC(=O)Nc3cccc(c3)C(F)(F)F
189	COc1c(cnc(n1)OC)c2ccc3c(c2)[nH+]cc(c3Nc4cc(cc(c4)Oc5cc(cc(c5)F)F)C(=O)[O-])S(=O)(=O)NC6CC6
190	CCOc1cc(ccc1OC(=O)c2ccccc2)/C=N/NC(=O)c3ccc(cc3)NS(=O)(=O)c4ccc(cc4)C
191	CCCOc1ccc(cc1)C(=O)Oc2ccc(cc2)/C=N/NC(=O)CNC(=O)c3ccc(c(c3)Cl)Cl
192	CCN(CC)c1ccc(c(c1)O)/C=N/NC(=O)CSc2nnc(n2c3ccc(cc3)Cl)c4ccc(cc4)OC
193	CCCOc1ccc(cc1)C(=O)Oc2ccc(cc2/C=N/NC(=O)CNC(=O)c3ccccc3Cl)Br
194	CCCCCCCCOc1ccc(cc1)c2cc(n[nH]2)C(=O)N/N=C/c3cc(c(c(c3)OC)OC(=O)C)OC
195	CCCCOc1ccc(cc1)C(=O)Oc2ccc(cc2)/C=N/NC(=O)C(=O)Nc3c(c4c(s3)CCCC4)C#N
196	CCOc1ccc(cc1)NC(=O)c2ccccc2NC(=O)C(=O)N/N=C/c3ccc(cc3)OC(=O)c4ccc(cc4)Br
197	c1ccc(cc1)NC(=O)c2ccccc2NC(=O)CCCCC(=O)Nc3ccccc3C(=O)Nc4ccccc4
198	COc1ccc(cc1)CC(=O)Nc2ccc(cc2)C(=O)N/N=C\c3ccc(c(c3)CSc4nc5ccccc5o4)OC
199	CCC(C)(C)n1c(nnn1)[C@@H](c2cc3ccc(cc3[nH]c2=O)C)[NH+](CCc4ccccc4)Cc5cccs5
200	CCC(C)(C)n1c(nnn1)[C@H](c2cc3cc(ccc3[nH]c2=O)C)[NH+](CCc4ccccc4)Cc5cccs5
201	CCc1ccc2c(c1)cc(c(=O)[nH]2)[C@@H](c3nnnn3C(C)(C)C)[NH+](CCc4ccccc4)Cc5cccs5
202	CCc1ccc2c(c1)cc(c(=O)[nH]2)[C@H](c3nnnn3C(C)(C)CC)[NH+](CCc4ccccc4)Cc5ccccc5
203	COc1ccc(cc1)OCC(=O)O[C@H]2C[C@@H]3C[NH+]4CCc5c6ccc(cc6[nH]c5[C@H]4C[C@@H]3[C@@H]([C@H]2OC)C(=O)OC)OC
204	CCCCN(CCCC)S(=O)(=O)c1ccc(cc1)C(=O)Nc2c(c3c(s2)C([NH2+]C(C3)(C)C)(C)C)C#N
205	CCOc1ccc(cc1)N([C@H](c2cc[nH+]cc2)C(=O)NC(C)(C)C)C(=O)Cn3nc(nn3)c4ccc(cc4)C
206	CCc1ccc2c(c1)cc(c(=O)[nH]2)C[NH+](Cc3cccs3)[C@@H](CC)c4nnnn4Cc5ccc(cc5)OC
207	CCOc1ccc(cc1Cl)/C(=C/2\[C@H](N(C(=O)C2=O)CCCn3cc[nH+]c3)c4cc(ccc4OC)OC)/O
208	CC[C@H](c1nnnn1CCc2ccccc2)[NH+](CCc3cccc(c3)C)Cc4cc5cc(cc(c5[nH]c4=O)C)C
	CC[C@@H](c1nnnn1C2CCCC2)[NH+](CCc3ccc(c(c3)OC)OC)Cc4cc5cc(ccc5[nH]c4=O)C

209	Cc1cc(ccc1OC)CNc2cc3c(c(n(c3[nH+]c2)CCc4ccccn4)C(=O)OC)NC(=O)C5CCC5
210	CC[NH+](CC)CCCN1[C@@H](C(=C(c2ccc(cc2)OCC)O)C(=O)C1=O)c3cccc(c3)Oc4ccccc4
211	CC[NH+](CC)CCCN1[C@H](C(=C(c2ccc(cc2)OCC)O)C(=O)C1=O)c3cccc(c3)Oc4ccccc4
212	CCOc1ccc(cc1Cl)/C(=C/2\[C@@H](N(C(=O)C2=O)CCC[NH+](C)C)c3cc(c(c(c3)OC)OC)OC)/O
213	CCCCCOc1ccc(cc1)[C@H]2/C(=C(\c3ccc4c(c3)OCCO4)/O)/C(=O)C(=O)N2CCCn5cc[nH+]c5
214	COc1ccc(c(c1OC)OC)/C=N\NC(=O)CSc2[nH+]c3ccccc3n2Cc4ccc(cc4)Cl
215	c1ccc(cc1)C(c2ccccc2)[NH+]3CCN(CC3)CCCn4cc(c5c4cc(cc5)C(=O)[O-])Cn6cc[nH+]c6
216	C[C@@H]1Cc2cc(ccc2O1)C(=O)C3=C(C(=O)N([C@H]3c4ccc(c(c4)OC)OCCC(C)C)CCC[NH+](C)C)O
217	CC[NH+](CC)CCN1[C@@H](C(=C(C1=O)O)C(=O)c2c(nc(s2)C)C)c3cc(c(c(c3)C(C)(C)C)O)C(C)(C)C
218	CCCC[NH+](C)CCN1[C@@H]([C@@]23C=C[C@@H](O2)[C@@H]([C@@H]3C1=O)C(=O)Nc4cccc(c4)OC)C(=O)NC5CCCCC5
219	Cc1ccc(cc1)CN2[C@@H]([C@@H](OC2=O)c3ccc(cc3)NC(=O)c4ccccc4OC)C(=O)NCC[NH+](C)C
220	CC[C@@H](C)[NH+](Cc1c(nn(c1Oc2ccccc2OC)c3ccccc3)C)C[C@@H](COCc4ccccc4)O
221	CC(C)N(c1ccccc1)C(=O)Cn2c3ccccc3[nH+]c2CCNC(=O)c4cc(c(c(c4)OC)OC)OC
222	CC[C@@H](c1nnnn1Cc2ccc(cc2)OC)[NH+](Cc3ccc(cc3)F)Cc4cc5ccc(cc5[nH]c4=O)OC
223	CC[C@@H](c1nnnn1Cc2ccc(cc2)OC)[NH+](Cc3ccc4c(c3)OCO4)Cc5cc6c(cc(cc6[nH]c5=O)C)C
224	Cc1ccc(c2c1cc(c(=O)[nH]2)C[NH+](CCc3ccc(c(c3)OC)OC)Cc4nnnn4Cc5ccc(cc5)F)C
225	CC[C@H](c1nnnn1Cc2ccc(cc2)F)[NH+](Cc3ccc(cc3)OC)Cc4cc5cc(ccc5[nH]c4=O)OC
226	Cc1cc(c2cc(c(=O)[nH]c2c1)[C@@H](c3nnnn3CCc4ccccc4)N(Cc5ccc(cc5)OC)Cc6ccc[nH+]c6)C
227	COc1ccccc1N2CC[NH+](CC2)Cc3ccc(cc3)C(=O)N/N=C/c4ccc(cc4)OCC(=O)Nc5cccc(c5)Cl
228	CC(C)(C)c1cc(c(c(c1)/C=[NH+]/CC/[NH+]=C/c2cc(cc(c2[O-])CN3CCOCC3)C(C)(C)C)O)CN4CCOCC4
229	C[C@@]\1(C[NH+]([C@@H](C/C1=N/CCc2ccc(c(c2)OC)OC)c3ccccc3)CCc4ccc(c(c4)OC)OC)O
230	C[C@@H]1Cc2cc(ccc2O1)C(=O)C3=C(C(=O)N([C@@H]3c4cccc(c4)OCCC(C)C)CCCn5cc[nH+]c5)O
231	Cc1cc2c(cc1OC)[nH]c3c2CC[NH+]4[C@@H]3C[C@@H]5[C@@H](C4)C[C@H]([C@@H]([C@H]5C(=O)OC)OC)OC(=O)c6cc(c(c(c6)OC)OC)OC
232	CC[C@@H]([C@@H]1C(=NN(C1=O)c2nc3ccccc3s2)CSc4ccc(cc4)Cl)[NH2+]CCc5c[nH]cn5
233	COc1ccc(cc1OC)CC[NH+](Cc2ccc(cc2)Cl)Cc3nc(cs3)C(=O)NCc4ccco4
234	CCCCCCNC(=O)c1csc(n1)C[NH+](Cc2ccc3c(c2)OCO3)Cc4ccc(cc4OC)OC
235	CCCC(=O)OCc1cnc(c2c1cc(/c(=[NH+]/c3ccc(cc3)C)/o2)C(=O)Nc4ccc(cc4OC)OC)C
236	COc1ccc(cc1)N/C(=[NH+]/CCc2ccc(c(c2)OC)OC)/S[C@@H]3CC(=O)N(C3=O)c4cccc(c4)Cl
237	Cc1ccc(cc1)C(=O)N/C(=C\c2cn(nc2c3ccc(cc3)Cl)c4ccccc4)/C(=O)NCCCn5cc[nH+]c5
238	CC(=O)Oc1cccc(c1)/C=[NH+]/c2cc(ccc2O)S(=O)(=O)c3ccc(c(c3)/N=C/c4cccc(c4)OC(=O)C)O
239	c1ccc(cc1)C(=O)Oc2ccc(cc2)c3csc([n+]3CCc4ccc(cc4)S(=O)(=O)N)Nc5cccc(c5)C(F)(F)F
240	Cc1cccc(c1)COc2ccc(cc2)C(=O)C3=C(C(=O)N([C@@H]3c4ccc(cc4)N(C)C)CCCn5cc[nH+]c5)O
241	COc1cc2cc[nH+]c(c2cc1OC)Cc3cc(c(cc3NC(=O)COc4ccc5ccccc5c4)OC)OC
242	CCOC(=O)C1=C(NC(=O)N[C@@H]1c2cccc(c2)F)CN(Cc3ccc[nH+]c3)C(=O)Cc4ccc(cc4)Cl
243	C[NH+](C)CCNC(=O)c1cccc(c1)Nc2c(cnn(c2=O)c3ccc(cc3)OC)Oc4ccc5c(c4)CCC5
244	CCc1cc(nc([nH+]1)SCc2ccc(o2)C(=O)NCc3ccco3)N4CCN(CC4)c5ccc(cc5)OC
245	COCc1cc(nc(n1)SCc2ccc(cc2)C(=O)NCc3cc[nH+]cc3)N4CCN(CC4)c5ccccc5F
	COc1cc2cc[nH+]c(c2cc1OC)Cc3cc(c(cc3NC(=O)CSc4nc5ccccc5s4)OC)OC

246	COc1cc2cc[nH+]c(c2cc1OC)Cc3cc(c(cc3NC(=O)CSc4nnc(o4)c5cccs5)OC)OC
247	CCN(CC)S(=O)(=O)c1cccc(c1)c2nnc(o2)SCC(=O)Nc3cc(c(cc3OC)Cl)C
248	CCc1ccc(cc1)N([C@H](c2cc(c(c(c2)OC)OC)OC)C(=O)NC3CCCCC3)C(=O)c4cnccn4
249	CCOc1ccc(cc1)NC(=O)CN2C(=O)/C(=C\c3cc(c(c(c3)Cl)OCc4ccccc4C#N)OC)/SC2=O
250	[H]/N=C\1/C(=C/c2cc(c(c(c2)Cl)OCCOc3ccc(cc3)C)OC)/C(=O)N=C4N1N=C(S4)COc5ccccc5
251	CC(C)[C@@H](C(=O)Nc1ccc(cc1)N(C)C)N(Cc2nc(cs2)c3ccccc3)C(=O)CN4c5ccccc5C(=O)C4=O
252	CN(C)c1ccc(cc1)NC(=O)[C@@H](c2cccs2)N(Cc3ccc(cc3)OC)C(=O)Cn4c5ccccc5nn4
253	Cc1cc(nc(n1)SCC(=O)N(Cc2ccccc2Cl)[C@H](c3ccc(cc3)OC)C(=O)Nc4ccc(cc4)OC)C
254	CCOc1ccc(cc1OCC)CCNC(=O)Cn2cnc3c(c2=O)c(c(s3)C)c4ccc(cc4)Cl
255	CCc1ccccc1NC(=O)CSC2=NC(=C([C@@H](C2C#N)c3ccccc3Cl)C(=O)OCCOC)C
256	CCN(CC)c1ccc(cc1)NC(=O)CSc2nnc(n2Cc3ccco3)COc4ccc(cc4)Cl
257	Cc1ccc(o1)c2nnn(n2)CC(=O)N(c3ccc(cc3)Cl)[C@@H](c4cccs4)C(=O)NCCC(C)C
258	CCC(C)(C)NC(=O)[C@H](c1ccc(cc1)OC)N(c2ccc(cc2)C(=O)C)C(=O)Cn3c4ccccc4nn3
259	CCOCCOC(=O)C1=C(NC2=C([C@H]1c3cc(c(cc3OC)OC)OC)C(=O)C[C@@H](C2)c4cccs4)C
260	CCCCN(CC)S(=O)(=O)c1ccc(cc1)C(=O)Nc2cc(nn2c3nc4ccc(cc4s3)OC)C
261	CCCCCNC(=O)c1csc(n1)CN(CCc2ccc(c(c2)OC)OC)Cc3ccc4c(c3)OCO4
262	CC[NH+](CC)CCN(c1nc2ccccc2s1)C(=O)c3ccccc3[N-]S(=O)(=O)c4ccc(cc4)F
263	CCCOc1cccc(c1)[C@@H]2c3c(n[nH]c3C(=O)N2CCc4ccc(c(c4)OC)OC)c5ccc(cc5)OC
264	CCCCSc1nc2n(n1)[C@H](C(=C(N2)C)C(=O)OC(C)C)c3cc(c(c(c3)Br)OC)OC
265	CCCCOC(=O)C1=C(Nc2nc(nn2[C@H]1c3cc(c(c(c3)Br)OC)OCC)SCC)C
266	CCOc1ccc(cc1)[C@H](C(=O)NC2CCCC2)N(c3cccc4c3CCCC4)C(=O)Cn5nc(nn5)c6cccs6
267	Cc1ccc(cc1)c2nnn(n2)CC(=O)N(CCc3ccccc3C)[C@@H](c4ccncc4)C(=O)NC5CCCC5
268	COc1ccccc1c2cc(nc(n2)S(=O)(=O)CCCC(=O)Nc3ccc(c(c3)Cl)n4cccc4)C(F)(F)F
269	CC[C@@H](c1nnnn1CCc2ccccc2)N(Cc3ccc4c(c3)OCO4)Cc5cc6ccc(cc6[nH]c5=O)C
270	CC[C@@H](c1nnnn1Cc2ccc(cc2)OC)N(Cc3cccs3)Cc4cc5cc(c(cc5[nH]c4=O)C)C
271	CC[C@@H](c1nnnn1CCc2ccccc2)N(Cc3cccs3)Cc4cc5cc6c(cc5[nH]c4=O)OCO6
272	CCOc1ccc(cc1)n2c(nnc2SCC(=O)N/N=C/c3cc(ccc3OC)Br)c4ccccc4
273	CCOc1ccc(cc1OC)[C@@H]2C(=C(Nc3n2nc(n3)SCc4ccccc4Cl)C)C(=O)OC(C)C
274	CN(C)c1ccc(cc1)c2nnc(n2CC=C)SCC(=O)Nc3c(c4c(s3)CCCCC4)C(=O)OC
275	Cc1ccc(c(c1)OCc2nnc(n2C)SCC(=O)Nc3c(c4c(s3)CCCC4)C(=O)OC(C)C)C
276	COc1ccccc1OCc2nnc(n2CC=C)SCC(=O)Nc3ccc(cc3)I
277	CC1=C([C@H](C2=C(N1)C[C@@H](CC2=O)c3ccc(cc3)OC)c4cc(c(c(c4)OC)OC)OC)C(=O)OCCOC
278	[H]/N=C\1/C(=C/c2cc(c(c(c2)Cl)OCCOc3ccc(cc3)C)OCC)/C(=O)N=C4N1N=C(S4)CCC
279	[H]/N=C\1/C(=C/c2cc(c(c(c2)Cl)OCCOc3ccc(cc3)C)OC)/C(=O)N=C4N1N=C(S4)CCCC
280	C[C@H](CCC(=O)O)[C@@H]1CC[C@@H]2[C@@]1([C@H](C[C@H]3[C@H]2[C@@H](C[C@@H]4[C@@]3(CC[C@H](C4)OC(=O)C)C)OC(=O)C)OC(=O)C)C
281	C[C@@H](CCC(=O)O[C@H]1C[C@H]2[C@@H]3CC=C4C[C@@H](CC[C@@]4([C@H]3CC[C@@]2([C@H]1NC(=O)C)C)C)OC(=O)C)COC(=O)C
282	CC[C@@H](C)[C@@H](C(=O)Oc1ccc2c(cc(=O)oc2c1)c3ccc(cc3)OC)NS(=O)(=O)c4ccc(cc4)C

283	Cc1ccc(cc1)S(=O)(=O)Nc2ccc3c(c2)c(c(n3S(=O)(=O)c4ccc(cc4)C)C)C(=O)OCCOC
284	CC1=C([C@@H](C2=C(N1)C[C@H](CC2=O)c3cccs3)c4ccc(c(c4)OC)OC)C(=O)OCCOc5ccccc5
285	Cc1ccc(cc1)OCc2nnc(n2c3ccc(cc3)OC)SCC(=O)Nc4cc(c(cc4OC)Cl)C
286	CC[C@@H](C)c1ccc(cc1)OCCOc2c(cc(cc2Cl)/C=C\3/C(=O)NC(=O)N(C3=O)c4ccc(cc4)C)OC
287	CCOc1cc(ccc1OCCOc2ccccc2OC)/C=C\3/C(=O)NC(=O)N(C3=O)c4ccc(cc4)Br
288	[H]/N=C\1/C(=C/c2cn(c3c2cccc3)CCOc4ccccc4CC=C)/C(=O)N=C5N1N=C(S5)COc6ccccc6C
289	[H]/N=C\1/C(=C/c2cn(c3c2cccc3)CCCOc4cccc(c4)OC)/C(=O)N=C5N1N=C(S5)COc6ccccc6C
290	CC(C)c1ccc(c(c1)S(=O)(=O)NCCN2CCN(CC2)S(=O)(=O)c3cc(ccc3OC)C(C)C)OC
291	Cc1ccccc1CSc2nnc(n2c3ccc(cc3)[N+](=O)[O-])[C@H](C)NC(=O)COc4ccc(cc4)Cl
292	Cc1cccc(c1)C(=O)N(Cc2ccco2)Cc3cc(ccc3N(C)C)NS(=O)(=O)c4ccc(cc4)OC
293	CCCOc1ccc(cc1C)C(=O)C2=C(C(=O)N([C@H]2c3cccc(c3)Oc4ccccc4)Cc5cccnc5)O
294	CCCOc1ccccc1CN(Cc2ccco2)c3cnc(nc3C(=O)Nc4ccc(cc4)C)S(=O)(=O)C
295	CC(=C)Cn1c(nnc1SCC(=O)N/N=C/c2ccc(c(c2)OC)OC)c3ccc(cc3)Br
296	COc1cc(cc(c1OC)OC)/C=N/NC(=O)CSc2nnc(n2c3ccccc3)c4ccc(cc4)Br
297	COc1ccc(cc1)Nc2nc(nc(n2)N/N=C/c3cc(c(c(c3)I)OCC#C)OC)NCc4ccco4
298	Cc1ccc(cc1[N+](=O)[O-])S(=O)(=O)N(CC(=O)NCCSCc2ccc(cc2Cl)Cl)c3ccc(cc3)OC
299	Cc1ccc(cc1C)OCc2nnc(n2c3ccccc3)SCC(=O)Nc4ccc5c(c4)sc(n5)SCC(=O)Nc6ccccc6
300	CC(=O)O[C@H]([C@@H]([C@@H](C(=O)Nc1cccc2c1cccc2)OC(=O)C)OC(=O)C)[C@@H](C(=O)Nc3cccc4c3cccc4)OC(=O)C
301	Cc1c(c(sc1C(=O)OC(C)C)NC(=O)CCCC(=O)Nc2c(c(c(s2)C(=O)OC(C)C)C)C(=O)OC(C)C)C(=O)OC(C)C
302	CCCOc1c(cccc1OC)[C@H]2C(=C(N=c3n2c(=O)/c(=C\c4ccc(c(c4)Br)OCC(=O)N)/s3)C)C(=O)OCC
303	COc1cc(nc(n1)OC)NS(=O)(=O)c2ccc(cc2)NC(=S)NC(=C(C(F)(F)F)C(F)(F)F)C(C(F)(F)F)(F)F
304	CCc1cc(cc(c1)OCCOc2c(cc(cc2Cl)/C=N/Nc3cc(nc(n3)N4CCCC4)N5CCCC5)OC)C
305	CCCCCCOc1ccc(cc1)OCCOc2c(cc(cc2Br)/C=N/NC(=O)c3ccncc3)OCC
306	CCCCCCOc1ccc(cc1)OCCOc2c(cc(cc2I)/C=N/NC(=O)c3ccncc3)OC
307	CCOC(=O)NCc1ccc(cc1)S(=O)(=O)Nc2cc(c(cc2Cc3c4cc(c(cc4ccn3)OC)OC)OC)OC
308	c1ccc(cc1)COP(=O)(OCc2ccccc2)OC[C@@H]3[C@H]([C@H]([C@@H](O3)COP(=O)(OCc4ccccc4)OCc5ccccc5)O)O
309	CC(=O)N[C@@H]1[C@H]([C@@H]([C@H](O[C@H]1Sc2nc3ccccc3n2CCCOc4ccc5ccccc5c4)COC(=O)C)OC(=O)C)OC(=O)C
310	CC[NH+](CC)c1ccc(cc1)NC(=O)CSc2c(c(sn2)SCC(=O)Nc3ccc(cc3)N(CC)CC)C#N
311	CCCOc1ccc(cc1)NC(=O)C[C@H]2C(=O)N(C(=S)N2CCCn3c(c(c(n3)C)[N+](=O)[O-])C)c4ccc(cc4)Cl
312	[H]/N=C\1/C(=C/c2ccc(c(c2)OCC)OCCOc3ccc(cc3)Br)/C(=O)N=C4N1N=C(S4)CCCCCCC
313	[H]/N=C\1/C(=C/c2cc(c(c(c2)Br)OCCOc3ccc(cc3)C(C)(C)C)OC)/C(=O)N=C4N1N=C(S4)CCCCCCC
314	CCOc1cc(ccc1OCCSc2cccc3c2cccc3)/C=N/Nc4cc(nc(n4)N5CCOCC5)N6CCOCC6
315	COc1cccc(c1OC)[C@@H]2CC(=NN2C(=O)CSc3nnc(n3c4cccc(c4)C(F)(F)F)CNC(=O)c5ccco5)c6cccs6
316	CCOc1ccc(cc1)n2c(nnc2SCC(=O)N3[C@@H](CC(=N3)c4cccs4)c5cccc(c5OC)OC)CNC(=O)c6ccco6
317	CCN(Cc1ccccc1)S(=O)(=O)c2ccc(cc2)C(=O)Nc3nnc(o3)c4cc(c(c(c4)OCC)OCC)OCC
318	CCC/C(=N\OC(C(F)(F)F)(C(F)(F)F)NC(=O)Nc1cccc(c1)[C@](C(=O)OCC)(C(F)(F)F)O)/Cl
319	CCCOc1ccc(cc1OCC)[C@@H]2C(=C(C(=O)N2c3nnc(s3)SCc4ccccc4F)O)C(=O)c5ccc6c(c5)OCCO6
	CC[NH+](CC)CCCN1[C@@H](C(=C(C1=O)[O-])C(=O)c2ccc3c(c2)OCCO3)c4ccc(c(c4)OCC)OCCC(C)C

320	CCOc1cc(ccc1OCC=C)[C@H]2C(=C(C(=O)N2c3nnc(s3)SCc4ccccc4F)O)C(=O)c5ccc6c(c5)C[C@@H](O6)C
321	CCOc1cc(ccc1OCCC(C)C)[C@@H]2C(=C(C(=O)N2c3nnc(s3)SCc4ccccc4F)O)C(=O)c5ccc6c(c5)C[C@H](O6)C
322	CCOc1cc(cc(c1OCC)OCC)C(=O)NCc2nnc(n2c3cc(ccc3OC)OC)SCC(=O)N4CCc5c4cccc5
323	c1ccc(cc1)c2nc(c(o2)COC(=O)[C@@H](c3ccccc3)NC(=O)c4ccccc4)COC(=O)[C@@H](c5ccccc5)NC(=O)c6ccccc6
324	CCOc1ccc(cc1OCC)Cn2c3c(nc2SCC(=O)Nc4ccc(cc4)OC(F)(F)F)n(c(=O)n(c3=O)C)C
325	CC(C)(C)c1c2c(n(n1)c3ccc(cc3)OC)N(C(=O)CS[C@H]2c4ccc(c(c4)OC)OC)CC(=O)NCCCOC
326	Cc1ccc(c(c1)C)n2c(cc(n2)C(C)(C)C)NC(=O)CN(CCCOC)C(=O)Nc3c(cccc3C(C)C)C(C)C
327	COc1ccc(cc1OC)CCN(Cc2cccs2)C(=O)CN(C[C@@H]3CCCO3)C(=O)Nc4c(cccc4Cl)Cl
328	CCOCCCN(CC(=O)N(CCc1ccc(c(c1)OC)OC)Cc2ccc(s2)C)C(=O)Nc3ccc(c(c3)C)C
329	CCOc1ccc(cc1)NC(=O)N(CCOC)CC(=O)N(CCc2ccc(c(c2)OC)OC)Cc3ccc(s3)C
330	CCOc1ccc(cc1F)c2c(cn(n2)c3ccccc3)C4C(=C(NC(=C4C(=O)OCCOC)C)C)C(=O)OCCOC
331	CCN(CC)c1ccc(cc1)/C=N/NC(=O)CSc2nnc(n2CC=C)CNc3ccc(cc3)I
332	CC1=C(C(C(=C(N1)C)C(=O)OCC(C)C)c2cn(nc2c3ccc(c(c3)F)OC)c4ccccc4)C(=O)OCC(C)C
333	CCCCOc1ccc(cc1)C(=O)C2=C(C(=O)N([C@H]2c3ccc(c(c3)OC)OCC=C)c4nnc(s4)SCc5ccccc5Cl)O
334	COc1ccc(cc1OC)C(=O)C2=C(C(=O)N([C@H]2c3ccc(cc3)OCC=C)c4nnc(s4)SCc5cccc6c5cccc6)O
335	CCCOc1ccc(cc1)C(=O)Oc2ccc(cc2/C=N/NC(=O)CNC(=O)c3ccc(cc3)OC)Br
336	Cc1ccc(cc1)n2c(nnc2SCC(=O)Nc3ccccc3)Cc4nnc(n4c5ccc(cc5)C)SCC(=O)Nc6ccccc6
337	Cc1ccc(c(c1)C)n2c(nnc2SCC(=O)Nc3ccccc3)Cc4nnc(n4c5ccc(cc5C)C)SCC(=O)Nc6ccccc6
338	CCCCCCOc1ccc(cc1OC)[C@@H]2C(=C(C(=O)N2c3nc(c(s3)C(=O)OC)C)O)C(=O)c4ccc(c(c4)F)C
339	CCCOc1ccc(cc1)C(=O)Oc2ccc(cc2OCC)/C=N/NC(=O)COc3ccc4ccccc4c3Br
340	CC1=C(C(C(=C(N1)C)C(=O)OCCOC)c2cn(nc2c3ccc4ccccc4c3)c5ccccc5)C(=O)OCCOC
341	c1ccc2c(c1)/C(=C/3\C(=O)N(C(=S)S3)CCCCCCCCCCC(=O)O)/C(=O)N2CC(=O)Nc4cccc(c4)C(F)(F)F
342	CCCCOc1ccc(cc1)C(=O)Oc2ccc(cc2OC)/C=N/NC(=O)CNC(=O)c3cc(c(c(c3)OC)OC)OC
343	CCOc1ccc(cc1)NC(=O)c2ccccc2NC(=O)C(=O)N/N=C/c3cc(ccc3OC(=O)/C=C/c4ccccc4)Br
344	CC1=C(C(C(=C(N1)C)C(=O)OCC=C)c2cn(nc2c3ccc(cc3)S(=O)(=O)N(C)C)c4ccccc4)C(=O)OCC=C
345	C=CC(=O)OC[C@@H](COc1ccc(cc1)C2(c3ccccc3-c4c2cccc4)c5ccc(cc5)OC[C@@H](COC(=O)C=C)O)O
346	COc1ccc(cc1)NC(=O)CSC2=Nc3ccccc3C4=N[C@@H](C(=O)N42)CCC(=O)NCc5cccs5
347	CCOc1cc(ccc1OC(=O)c2ccc(cc2)Cl)/C=N/NC(=O)CNC(=O)c3ccccc3Cl
348	CCOc1ccc(cc1)C(=O)NCC(=O)N/N=C/c2ccc(cc2)OC(=O)c3ccc(cc3Cl)Cl
349	Cc1ccc(cc1)C(=O)N/C(=C/c2cn(nc2c3ccc(cc3)Cl)c4ccccc4)/C(=O)NCCCn5ccnc5
350	CCOC(=O)CC[C@H](C(=O)OCC)NC(=O)c1ccc(cc1)Nc2cnc3cc(ccc3n2)C(F)(F)F
351	CCN(CC)S(=O)(=O)c1cc(ccc1C)c2c3ccccc3c(nn2)Nc4ccc(cc4)OCC(=O)NC
352	CCCN(CCC)S(=O)(=O)c1ccc(cc1)C(=O)NNC(=O)c2c(onc2c3ccccc3Cl)C
353	CCOc1ccc(cc1)S(=O)(=O)Nc2ccc(c3c2cccc3)NS(=O)(=O)c4ccc(cc4)OCC
354	CCOc1ccccc1N(CC(=O)Nc2ccc(cc2)NC(=O)C)S(=O)(=O)c3ccc(cc3)SC
355	Cc1cccc(c1)n2c(nnc2SCC(=O)Nc3ccc(cc3)OC)CNC(=O)c4cccc(c4)OC
356	CCOc1ccc(cc1)NC(=O)CSc2nnc(n2c3cccc(c3)C)CNC(=O)c4ccc(c(c4)C)C
	CCc1nnc(n1NC(=S)NC(=O)c2ccc(c(c2)[N+](=O)[O-])N3CCCC3)SCc4ccccc4

357	CCOc1ccc(cc1)n2c(nnc2SCC(=O)Nc3cc(ccc3C)C)CNC(=O)c4cccc(c4)C
358	CCOc1cc(cc(c1OCC)OCC)C(=O)Nc2nnc(s2)SCC(=O)Nc3ccccc3F
359	Cc1cccc(c1)NC(=O)CSc2nnc(n2c3cccc(c3C)C)CNC(=O)Cc4ccc(cc4)OC
360	CCc1ccc2c(c1)cc(c(=O)[nH]2)CN(CCc3ccc(c(c3)OC)OC)C(=O)Nc4ccc(cc4)OC
361	CCN(CC)S(=O)(=O)c1cc(ccc1C)NC(=O)[C@@H](C)Sc2nnc(s2)Nc3ccccc3F
362	CCOC(=O)Nc1ccc2c(cc(=O)oc2c1)COC(=O)[C@H](CCSC)NC(=O)c3cccc(c3)C
363	CCCCOC(=O)C[C@@H]1C(=O)NCCN1C(=S)NC(=O)c2cc(ccc2OCCC)Br
364	CCCCCOC(=O)C[C@H]1C(=O)NCCN1C(=S)NC(=O)c2cc(ccc2OCC)Br
365	Cc1cc(ccc1/C(=N/NC(=O)c2cc(ccc2O)Cl)/N=N/c3ccccc3)N(CCC#N)CCC#N
366	CCOc1ccc(cc1)NC(=O)C[C@H]2C(=O)N(C(=S)N2CCNC(=O)C)c3ccc(c(c3)Cl)OC
367	CC(C)(C)c1nnc(n1CC=C)SCC(=O)Nc2ccc(cc2)S(=O)(=O)Nc3cccc(c3)Cl
368	Cc1ccccc1C(=O)Nc2ccc(c(c2)C(=O)NCCCOC)N3CCN(CC3)c4ccccc4OC
369	CCCCCCN1[C@@H]([C@@]23C=C[C@@H](O2)[C@@H]([C@@H]3C1=O)C(=O)Nc4cccc(c4)OC)C(=O)N[C@@H]5CCCC[C@@H]5C
370	C/C(=N/NC(=O)CSc1nnc(n1CC=C)CNc2ccc(cc2)Cl)/c3ccc(cc3OC)OC
371	CCCCCOc1c(cc(cc1Cl)C(=O)Nc2ccc(cc2)S(=O)(=O)Nc3nccc(n3)C)Cl
372	c1cc(ncc1)N/N=C/c2ccc(cc2)O[C@H]3OCCO[C@H]3Oc4ccc(cc4)/C=N/Nc5ncccc5
373	CC[C@H](C(=O)Nc1ccc(cc1)S(=O)(=O)NCc2ccco2)Sc3cc(nc(n3)C)c4ccccc4
374	CCCCCOc1ccc(cc1OC)[C@H]2c3c(n[nH]c3C(=O)N2Cc4cccnc4)c5c(cc(cc5O)C)C
375	Cc1cc(nc(n1)C)NS(=O)(=O)c2ccc(cc2)NC(=O)COc3ccc(cc3)C(C)(C)CC(C)(C)C
376	CCOc1ccccc1NC(=O)C2=C(Nc3nc(nn3[C@@H]2c4ccc(c(c4)OC)OC(C)C)SCC)C
377	CC[C@@H](C(=O)Nc1cccc(c1)C)SC2=Nc3ccccc3C4=N[C@@H](C(=O)N42)CCC(=O)NCc5cccs5
378	Cc1c(c(nc(c1C#N)SCc2nnc(n2N)CSc3c(c(c(n3)C)CC=C)C)C#N)C)CC=C
379	Cc1ccccc1C(=O)NCCc2nnc(n2C)SCC(=O)Nc3ccc(cc3)/N=N/c4ccccc4
380	C[C@@H](C(=O)N/N=C/c1cc(ccc1OC)OC)Sc2nnc(n2CC=C)CNc3ccc(cc3)Cl
381	CCOc1ccc(cc1)NCc2nnc(n2c3ccccc3)SCCC(=O)Nc4ccc(cc4)OCC
382	c1cc(ncc1)N/N=C\c2ccc(cc2)O[C@H]3OCCO[C@H]3Oc4ccc(cc4)/C=N\Nc5ncccc5
383	Cc1cc(ccc1NC(=O)CSc2nnc(n2CC=C)[C@@H](C)NC(=O)c3ccc(cc3Cl)Cl)[N+](=O)[O-]
384	CC(=O)Nc1ccc(cc1)S(=O)(=O)N(CC(=O)N/N=C/c2ccccc2C(F)(F)F)c3ccc(cc3)OC
385	CC(C)c1ccc(cc1)NC(=O)Cn2c3ccccc3c(=O)n(c2=O)CCCCC(=O)Nc4ccccc4
386	CCOc1ccccc1CNC(=O)CN(C)c2nn3c(c(nc3s2)c4cccc(c4)F)NC(C)(C)C
387	COc1c(nccn1)NS(=O)(=O)c2ccc(cc2)NC(=O)CCCOc3ccc(cc3Cl)Cl
388	CS(=O)(=O)CC(=O)NCCn1ccc2c1c(ncn2)Nc3ccc(c(c3)Cl)Oc4cccc(c4)C(F)(F)F
389	CCNc1nc(nc(n1)N(C(=O)COc2ccc(cc2Cl)Cl)S(=O)(=O)c3ccc(cc3)C)NCC
390	CC(=O)Nc1ccc(cc1)S(=O)(=O)Oc2ccccc2CN(Cc3ccco3)C(=O)Nc4ccccc4OC
391	c1cc(ccc1NC(=O)CSc2nnc(s2)SCC(=O)Nc3ccc(cc3)SC#N)SC#N
392	Cc1ccc(c(c1)c2c3c([nH]n2)C(=O)N([C@@H]3c4cc(c(c(c4)OC)OC)OC)CCc5ccc(c(c5)OC)OC)O
393	CCOc1cc(ccc1OC(=O)c2ccc(cc2)OC)/C=N/NC(=O)C(=O)Nc3c(c4c(s3)CCCC4)C#N
	CCOCCOc1nc(n(n1)c2cccc(c2)NC(=O)Nc3ccc(cc3F)F)c4ccc(cc4)OC

394	CC[C@H]1CN(CC[C@H]1CC(=O)N(CC)C(=O)NCCC[NH+](C)C)C(=O)Nc2cccc(c2)C(F)(F)F
395	CCC[NH+]1CCc2c(sc(c2C(=O)NC)NC(=O)c3ccc(cc3)S(=O)(=O)N(CCC)CCC)C1
396	CCc1ccc2c(c1)cc(c(=O)[nH]2)C[NH+](Cc3ccccc3OC)Cc4nnnn4Cc5ccc(cc5)OC
397	CCc1ccc2c(c1)cc(c(=O)[nH]2)C[NH+](Cc3ccccc3OC)Cc4nnnn4Cc5ccc(cc5)F
398	CCc1ccc2c(c1)cc(c(=O)[nH]2)C[NH+](Cc3ccc(cc3)C)Cc4nnnn4Cc5ccc(cc5)OC
399	CCc1ccc2c(c1)cc(c(=O)[nH]2)C[NH+](Cc3ccc4c(c3)OCO4)[C@H](CC)c5nnnn5C(C)(C)CC
400	CCc1ccc2c(c1)cc(c(=O)[nH]2)C[NH+](Cc3ccccc3OC)[C@@H](CC)c4nnnn4C5CCCCC5
401	CC[C@@H](c1nnnn1Cc2ccccc2)[NH+](Cc3ccc(cc3)OC)Cc4cc5ccc(cc5[nH]c4=O)C
402	CC[C@H](c1nnnn1C(C)(C)CC)[NH+](Cc2ccc(cc2)F)Cc3cc4cc(c(cc4[nH]c3=O)OC)OC
403	Cc1ccccc1COc2ccc(cc2)/C(=C/3\[C@H](N(C(=O)C3=O)CCC[NH+](C)C)c4cccc(c4)OC)/O
404	CC[NH+](CC)CCNC(=O)c1ccc(cc1)NC(=S)NC(=O)c2cc(ccc2OC)Br
405	CCCCCOc1ccc(cc1OC)[C@@H]2C(=C(c3ccc4c(c3)OCCO4)O)C(=O)C(=O)N2CC[NH+](C)C
406	CCOc1cc(ccc1OCC=C)[C@H]2/C(=C(/c3ccc4c(c3)C[C@H](O4)C)\O)/C(=O)C(=O)N2CCC[NH+](C)C
407	COc1cc(cc(c1OC[C@@H](c2ccc3c(c2)OCO3)O)Br)C[NH2+]C[C@H](c4ccccc4)O
408	CCCOc1cccc(c1)C(=O)C2=C(C(=O)N([C@@H]2c3cccc(c3)Oc4ccccc4)Cc5cc[nH+]cc5)O
409	CCOc1cccc(c1)C(=O)C2=C(C(=O)N([C@@H]2c3cccc(c3)Oc4ccccc4)CCCn5cc[nH+]c5)O
410	COc1cccc2c1oc(c2)C(=O)C3=C(C(=O)N([C@H]3c4ccc(cc4)OCC=C)CCCn5cc[nH+]c5)O
411	C[NH+](C)CCCNC(=O)/C(=C\c1ccc(o1)c2cccc(c2)C(F)(F)F)/NC(=O)c3ccc(cc3)OC
412	CCCOc1ccc(cc1)[C@@H]2C(=C(C(=O)N2CCC[NH+](C)C)O)C(=O)c3c(nc(s3)c4ccccc4)C
413	Cc1c(sc(n1)c2ccccc2)C(=O)C3=C(C(=O)N([C@@H]3c4ccc(c(c4)OC)OC)CCC[NH+](C)C)O
414	CC[NH+](CC)CCN1[C@@H](C(=C(C1=O)O)C(=O)c2cc3cccc(c3o2)OC)c4ccc(cc4OC)OC
415	CC[NH+](CC)CCCN1[C@@H](C(=C(C1=O)O)C(=O)c2c(n3cccc(c3n2)C)C)c4ccc(cc4)C(C)(C)C
416	CCCCCCOc1ccc(cc1)[C@H]2C(=C(C(=O)N2CCCn3cc[nH+]c3)O)C(=O)c4c(nc(s4)C)C
417	CCNC(=S)N(CC[NH+](C)C)Cc1cnc(n1Cc2ccccc2)S(=O)(=O)Cc3ccccc3C
418	Cc1c([nH+]c(nc1N(C)CCc2ccc(c(c2)OC)OC)SCc3ccc(cc3)C(=O)NCC(C)C)C
419	CC(C)[NH+](Cc1[nH]c(=O)c2c(csc2n1)c3ccccc3OC)C[C@H](COc4ccc(cc4)Cl)O
420	CCc1c(c(n(n1)c2ccccc2)Oc3ccccc3)C[NH+](C[C@@H]4CCCO4)C[C@@H](COC(C)(C)C)O
421	CCc1c(c(n(n1)c2ccc(cc2)F)Oc3ccccc3)C[NH+](Cc4ccco4)C[C@@H](COC(C)(C)C)O
422	CC(C)(C)OC[C@@H](C[NH+](Cc1c(nn(c1Oc2cccc(c2)F)C)c3ccccc3)C[C@@H]4CCCO4)O
423	CCOc1ccc(cc1)c2nc(c(o2)NCCC[NH+](C)C)S(=O)(=O)c3ccc(cc3)Br
424	Cc1ccc(c(c1)C)OCCCCn2c3ccccc3[nH+]c2[C@H](C)NC(=O)Cc4ccc(c(c4)OC)OC
425	C[C@@H](c1[nH+]c2ccccc2n1CCCCOc3ccccc3OC)NC(=O)Cc4ccc(c(c4)OC)OC
426	COc1cc(cc(c1OC)OC)C(=O)NCCCc2[nH+]c3ccccc3n2Cc4ccc5ccccc5c4
427	CC(C)(C)c1ccc(cc1)OCCCCn2c3ccccc3[nH+]c2CNC(=O)c4ccc(c(c4)OC)OC
428	CCCOc1ccc(cc1)[C@@H](CNC(=O)c2ccc(o2)S(=O)(=O)c3ccc(cc3)Cl)[NH+](CC)CC
429	CCCCCOc1ccc(cc1)[C@H]2c3c(n[nH]c3C(=O)N2CC[NH+]4CCOCC4)c5ccc(cc5)Cl
430	CCCCOc1ccc(cc1OCC)[C@H]2c3c(n[nH]c3C(=O)N2Cc4cc[nH+]cc4)c5ccc(cc5)OC
	Cc1ccc2cc(c(=O)[nH]c2c1C)C[NH+](CCc3ccc(c(c3)OC)OC)Cc4nnnn4Cc5ccccc5

431	CC[NH+](CC)CCC[C@@H](C)NC(=O)c1cc(nc2c1c(nn2[C@@H]3CCS(=O)(=O)C3)C)c4ccc(cc4)C
432	CC[NH+](CC)CCC[C@@H](C)NC(=O)c1cc(nc2c1c(nn2[C@H]3CCS(=O)(=O)C3)C)c4ccc(cc4)C
433	COc1cc2cc[nH+]c(c2cc1OC)Cc3cc(c(cc3NS(=O)(=O)c4ccc(cc4)N5CCCC5=O)OC)OC
434	CCCOc1ccc(cc1OC)[C@H]2CN3C(=O)CN(C(=O)[C@@]3([C@H]4C2=c5ccccc5=[NH+]4)C)CCCCCO
435	COc1ccccc1[C@@H](CNC(=O)c2ccc(cc2)S(=O)(=O)Nc3ccccc3OC)[NH+]4CCCC4
436	Cc1cnc(nc1Nc2cccc(c2)S(=O)(=O)NC(C)(C)C)Nc3ccc(cc3)OCC[NH+]4CCCC4
437	CCOc1cc(ccc1OCc2ccccc2C#N)/C=N\Nc3[nH+]c(nc(n3)N4CCCC4)N5CCCC5
438	Cc1ccc(cc1Nc2nccc(n2)c3cccnc3)NC(=O)/C=C/c4ccc(cc4)OCCC[NH+](C)C
439	Cc1cccc(c1)CS(=O)(=O)c2ncc(n2C3CCCCC3)CN(CC[NH+](C)C)C(=O)NC(C)(C)C
440	CCOc1cccc(c1)c2ccc3[nH+]c(c(n3c2)CCC(=O)NCCc4ccccn4)c5ccc(cc5)OC
441	c1cc[nH+]c(c1)N/N=C\c2ccc(cc2)O[C@@H]3[C@@H](OCCO3)Oc4ccc(cc4)/C=N/Nc5ccccn5
442	CCOc1ccc(cc1)n2c(nnc2SCC(=O)Nc3cccc4c3cccc4)COc5ccccc5
443	C[C@@H]1Cc2cc(ccc2O1)C(=C3[C@H](N(C(=O)C3=O)CCOC)c4ccc(c(c4)OC)OCCC(C)C)O
444	[H]/N=C\1/C(=C/c2cn(c3c2cccc3)CCOc4cccc(c4)C)/C(=O)N=C5N1N=C(S5)CCCCCCC
445	[H]/N=C/1\C(=C\c2ccc(c(c2)OC)OCCCOc3ccc(cc3)CC)\C(=O)N=C4N1C(=CS4)c5ccc(cc5)F
446	CCOc1ccccc1[C@H]2[C@@H]3CCCC[C@@]3(CCN2C(=O)c4cc(c(c(c4)OCC)OCC)OCC)O
447	CCCCN(CCCC)S(=O)(=O)c1ccc(cc1)NS(=O)(=O)c2ccc(cc2)OC(F)(F)F
448	CCCCCOC(=O)c1c2c(nc3ccccc3n2)n(c1NC(=O)c4ccc(o4)Br)CC=C
449	Cc1ccc(cc1)[C@H](C(=O)NCCC(C)C)N(c2ccc(cc2)C(=O)C)C(=O)Cn3c4ccccc4nn3
450	CC1=C([C@H](C2=C(N1)CC(CC2=O)(C)C)c3cc(c(c(c3)OC)OC)OC)C(=O)OCCOc4ccccc4
451	CCCOC(=O)C1=C(NC2=C([C@@H]1c3cc(c(c(c3)OC)OC)OC)C(=O)C[C@@H](C2)c4ccccc4OC)C
452	CC[C@@H](C)OC(=O)C1=C(NC2=C([C@H]1c3ccccc3OCC)C(=O)C[C@H](C2)c4ccc(c(c4)OC)OC)C
453	CC1=C([C@H](C2=C(N1)C[C@@H](CC2=O)c3ccccc3)c4cc(c(c(c4)OC)OC)OC)C(=O)OCCOC
454	CCC(C)(C)n1c(nnn1)[C@@H](c2cc3cccc(c3[nH]c2=O)C)N(CCc4ccccc4)Cc5ccccc5
455	CC[C@@H](c1nnnn1CCc2ccccc2)N(Cc3ccccc3Cl)Cc4cc5cc(ccc5[nH]c4=O)C
456	Cc1ccc(cc1)SCCNC(=O)CN(c2cc(cc(c2)C)C)S(=O)(=O)c3ccc(c(c3)OC)OC
457	CCCOc1cccc(c1)[C@@H]2C(=C(C(=O)N2CCC[NH+](CC)CC)[O-])C(=O)c3ccc(cc3)OC(C)C
458	CCN(CC)c1ccc(c(c1)C)NC(=O)c2cnc3c(c(nn3c2C)C)c4ccc(c(c4)OCC)OCC
459	CC(C)c1cc(c(s1)NC(=O)/C(=C\c2ccc(o2)COc3cccc(c3)C(F)(F)F)/C#N)C(=O)OC
460	CCCOC(=O)C1=C(NC2=C([C@@H]1c3ccc(c(c3OC)OC)OC)C(=O)C[C@H](C2)c4ccccc4OC)C
461	COc1cccc(c1)c2c(cn(n2)c3ccccc3)/C=C/4\C(=O)N(C(=S)S4)CCCCCC(=O)O
462	CCOc1ccc(cc1)N(CC(=O)NCc2ccc(cc2)OC(C)C)S(=O)(=O)c3ccc(cc3)Cl
463	COc1ccc(cc1)CNC(=O)c2ccc(cc2)Cn3c4cnccc4nc3SCc5ccc(cc5)OC
464	CCOCCOc1nc(n(n1)c2ccc(cc2)NC(=O)c3ccc(cc3)C)c4cccc(c4)C(F)(F)F
465	CCOCCOc1nc(n(n1)c2ccc(cc2)NC(=O)c3cccc(c3)F)c4cccc(c4)C(F)(F)F
466	CC(C)CN(CC(=O)Nc1nc(cn1c2ccc(c(c2)OC)OC)c3ccc(cc3)Cl)C(=O)C4CC4
467	CC(C)(C)OC[C@@H](CN(Cc1ccco1)Cc2c(nn(c2Oc3ccc(cc3)F)C)c4ccccc4)O
	C[C@@H]1CCCCN1c2c(c(no2)c3ccccc3)CN(CCCOC)C[C@@H](COCc4ccccc4)O

468	CC(C)(C)CC(C)(C)NC(=O)[C@H](c1ccc(cc1)N(C)C)N(Cc2ccco2)C(=O)c3ccccc3OC
469	CCCN([C@H](c1ccc(cc1)C(F)(F)F)C(=O)NCS(=O)(=O)c2ccc(cc2)C)C(=O)c3ccco3
470	COCCNC(=O)c1ccc(cc1)CSc2nnc(n2c3ccc(cc3)OC)c4ccccc4Cl
471	CCc1ccc(cc1)C(=O)c2cn(c3c(c2=O)ccc(n3)C)CC(=O)NCCc4ccc(c(c4)OC)OC
472	CC(=O)c1ccc(cc1)OC[C@@H](CN(Cc2ccc(o2)c3cccc(c3)Cl)[C@@H]4CCS(=O)(=O)C4)O
473	CCOCCCN(CC(=O)N(Cc1ccccc1)Cc2cccn2C)C(=O)Nc3ccc(cc3)SC
474	CCOc1cc(cc(c1OCc2cccc(c2)C)Cl)CNCCSc3nnnn3c4ccccc4
475	[H]\N=C\1/C(=C/c2cc(c(c(c2)OC)OCCOc3ccc(cc3)[C@H](C)CC)OC)/C(=O)N=C4N1C=CS4
476	CC[C@H](c1nnnn1Cc2ccc(cc2)F)N(Cc3ccc(cc3)OC)Cc4cc5cc(ccc5[nH]c4=O)C
477	CCOc1ccc(cc1)N(CC(=O)N/N=C(\C)/c2ccc(cc2)OC)S(=O)(=O)c3ccc(cc3)Cl
478	Cc1cc(cc(c1)N(CC(=O)N/N=C/c2ccc(cc2)SC)S(=O)(=O)c3ccc(c(c3)OC)OC)C
479	C[C@H](C(=O)Nc1ccc(cc1OC)OC)Sc2nc(cc(n2)C(F)(F)F)c3ccc(c(c3)OC)OC
480	CC(C)CCOP(=O)([C@](C(=O)OC)(C(F)(F)F)Nc1nc2ccccc2s1)OCCC(C)C
481	CC[C@@H](C)c1ccc(cc1)OCCn2cc(c3c2cccc3)/C=N\NC(=O)CSc4nnc(s4)C
482	CCCOc1ccc(cc1)NC(=O)[C@@H]2CC(=O)N(/C(=N/c3cccc(c3)C(F)(F)F)/S2)CC(=O)OC
483	CCOCCOc1nc(n(n1)c2cccc(c2)NC(=O)[C@@]3(C[C@@H]3F)Cl)c4cccc(c4)C(F)(F)F
484	CN(C)c1ccc(cc1CN(CCOC)S(=O)(=O)c2cccc(c2)Cl)NC(=O)c3cccs3
485	COCCNC(=O)c1ccccc1CSc2nnc(n2c3cccc(c3)Cl)c4cccc(c4)OC
486	CC(C)(C)NC(=O)N(CCOC)Cc1ccc(c(c1)OS(=O)(=O)c2ccc(c(c2)Cl)Cl)OC
487	COc1ccc(cc1)c2cc(c(c(n2)SCC(=O)NCc3cc(cc(c3)OC)OC)C#N)c4cccs4
488	CCCOc1ccc(cc1)NC(=O)C[C@@H]2C(=O)N(/C(=N/c3ccc(cc3)OC)/S2)Cc4cccs4
489	CCOc1cc(ccc1OC)/C=c/2\c(=O)n(/c(=C/C(=O)OCC)/s2)CC(=O)N[C@@H]3CCC[C@@H]([C@@H]3C)C
490	CCOc1ccc(cc1F)c2c(cn(n2)c3ccccc3)/C=C\4/C(=O)N(C(=S)S4)CCCCCC(=O)O
491	Cc1ccc(cc1C)Oc2cc(nc(n2)SC)n3cncn3
492	Cc1ccc(cc1)c2nnn(n2)Cc3ccc(cc3)C(=O)OC
493	COc1ccc2c(c1)s/c(=N\C(=O)c3ccno3)/n2CC#C
494	CC(C)c1nnc2n1nc(s2)c3cc(cc(c3)OC)OC
495	Cc1cc(nc(n1)n2c(nc(n2)c3ccccc3OC)Cl)C
496	C[C@@H]1C[C@H](CC(C1)(C)C)Oc2c(n3ccsc3n2)[N+](=O)[O-]
497	C[C@@H]1C[C@@H](CC(C1)(C)C)Oc2c(n3ccsc3n2)[N+](=O)[O-]
498	CC1=C[C@H](N=N1)[C@H]2N=N[C@@H](N2/N=C/C=C/c3ccccc3)S
499	CN(C)c1c(c(ncn1)Sc2ccc(cc2)Cl)[N+](=O)[O-]
500	c1cc(nc(c1[N+](=O)[O-])n2ccc(n2)C(F)(F)F)Cl
501	CCCn1c2ccccc2c3c1nc(nn3)n4c(cc(n4)C)C
502	C[C@H](c1nc(no1)C(C)C)Sc2nnc(o2)c3ccccc3
503	Cc1nc(no1)c2ccc(cc2)Oc3nc4ccccc4o3
504	CCCSc1nnc2n1c3c(c4ccccc4n3CC)nn2
	CN(Cc1ccccc1OC)c2nc(nc(n2)Cl)OC

505	CCOc1nc(nc(n1)Cl)N(C)Cc2ccc(o2)C
506	C1CCN(CC1)c2nc(nc(n2)Cl)OC[C@H]3CCCO3
507	Cc1ccc(cc1C)Oc2nc(nc(n2)Cl)n3cccn3
508	CC(C)Oc1nc(nc(n1)Cl)Oc2ccc(cc2)C#N
509	CC[C@H](C)CN(C)c1nc(nc(n1)Cl)N2CCOCC2
510	CCC[C@H](C)N(C)c1nc(nc(n1)Cl)n2cccn2
511	CC[C@@H]1CCCN(C1)c2nc(nc(n2)Cl)n3cccn3
512	CCCOc1nc(nc(n1)Cl)N(C)C2CCOCC2
513	CCCOc1nc(nc(n1)Cl)N2CCOC[C@H]2CC
514	CC[C@H]1COCCN1c2nc(nc(n2)Cl)OC(C)C
515	CC[C@@H]1CO[C@@H](CN1c2nc(nc(n2)Cl)N(C)C)C
516	CCCOc1nc(nc(n1)Cl)N2CCOCC2(C)C
517	CC(C)Oc1nc(nc(n1)Cl)N(C)C[C@H]2CCCCO2
518	CC(C)N(CC1CC1)c2nc(nc(n2)Cl)n3cccn3
519	C[C@H]1CN(CC(O1)(C)C)c2nc(nc(n2)Cl)OC(C)C
520	CC(C)Oc1nc(nc(n1)Cl)N(C)c2cccc(c2)OC
521	CCCOc1nc(nc(n1)Cl)N(C)[C@@H]2CCCN(C2)C
522	CN(c1ccc(cc1)C#N)c2c(ccc(n2)Cl)[N+](=O)[O-]
523	CC(C)Oc1nc(nc(n1)Cl)N(C)c2cccc(c2)C#N
524	Cc1nc2cc(ccc2o1)c3nc(on3)/C=C/c4ccco4
525	CCn1c2cc(ccc2nn1)c3nc(on3)c4cccs4
526	Cn1cnc2c1cc(cc2)c3nc(on3)c4cccc(c4)OC
527	Cc1ccc(c(c1C)OC(=O)c2nc3nc(cc(n3n2)C)C)C
528	Cc1ccc(cc1)c2nc(on2)Cn3c4ccccc4oc3=O
529	CCCC[C@@H]1C=CCN1C(=O)c2cc(n3c(n2)ncn3)C(C)C
530	c1ccc2c(c1)nc(o2)Sc3nnnn3C4CCCCC4
531	Cc1ccc2c(c1)nc(o2)N3CCC[C@H]3c4nc(on4)C(C)C
532	Cc1ccc(cc1C)OCc2nc(no2)c3ccnc(c3)OC
533	Cc1ccc(cc1OC)c2nc(no2)c3ccnc(c3)OC
534	c1ccc2c(c1)nnn2OCc3cc(on3)c4cccs4
535	CN1CCCC[C@H]1COc2nc(nc(n2)Cl)N3CCCC3
536	COCc1ccc(o1)c2nc(no2)c3cc4ccccc4o3
537	C[C@H](Cn1c2c(ccc(c2Br)OC)cn1)N=[N+]=[N-]
538	CCN(CC)c1nc(nc(n1)Cl)N2CCC[C@H](C2)N(C)C
539	c1ccc2c(c1)cc(o2)c3nnc(o3)SCc4ccon4
540	CCCCn1c(=O)c2ccccc2n3c1nnc3SCC(=O)OC
541	CCO[C@H]([C@@H]1CC(=NO1)c2ccc(cc2)OC)n3c4ccccc4nn3
	COc1ccc(cc1)n2c(nnn2)SCc3cc(ccc3OC)OC

542	Cc1c(cco1)c2nnc(o2)SCC(=O)c3ccc(cc3OC)OC
543	Cc1ccnc2n1nc(n2)C(=O)OCC(=O)c3ccc(cc3)C(C)(C)C
544	c1ccc2c(c1)cc(o2)C(=O)COC(=O)CN3C(=O)c4ccccc4C3=O
545	Cc1cc(ccc1Cl)OCC(=O)Oc2cccc(c2)n3cnnn3
546	COc1cc(ccc1OC(F)F)c2nc(on2)/C(=C/c3ccco3)/C#N
547	CC(C)n1c(nnn1)SCc2coc(n2)c3ccc(cc3)OC
548	CCCOc1ccccc1C(=O)Oc2ccc(cc2)n3cnnn3
549	CCOc1ccc(cc1)c2nc(c(o2)C)CN(C)Cc3c(non3)C
550	CCn1c(nnc1SCc2nc(no2)c3ccco3)c4cccs4
551	CCCn1c(nnn1)CSc2c3ccccc3nc(n2)c4ccco4
552	c1cc(oc1)Cn2c(nnn2)SCc3coc(n3)c4ccc(cc4)F
553	c1ccc(cc1)Cc2nc(on2)CSc3nnc(o3)c4ccco4
554	CC(C)(C)n1c(nnn1)SCc2nc(no2)Cc3ccccc3
555	CC(C)(CCl)c1nc(no1)c2ccc(cc2)OS(=O)(=O)N(C)C
556	COCc1nc(no1)c2ccc(nc2OC)c3ccc(cc3)OC
557	c1ccc(c(c1)OCCOC(=O)c2ccc(cc2)n3cnnn3)Cl
558	c1ccc2c(c1)C(=O)N(C2=O)CCCOc3ccc(cc3)c4nnco4
559	CCOc1ccc(cc1)c2nc(cs2)C(=O)O[C@H](C)c3nc(no3)C
560	COC(=O)c1ccccc1OC(=O)CCc2nc(no2)c3ccsc3
561	CCCN(Cc1ccc(cc1)C#N)S(=O)(=O)c2ccccc2[N+](=O)[O-]
562	COC(=O)c1cccc(c1)OCCSc2nnc(o2)c3cccnc3
563	Cc1ccc(cc1)OCCOc2ccc(cc2OC)/C=N/[C@@H]3N=CN=N3
564	CCOc1cc(ccc1OCc2nc(no2)c3cccc(c3)OC)C#N
565	Cc1ccccc1C(=O)O[C@@H]2CCCC[C@@H]2n3cc(nn3)C(=O)OC
566	CC(C)Cc1nnc2n1nc(s2)Cc3cc(c(c(c3)OC)OC)OC
567	COc1cc(cc(c1)OCc2nc(no2)COc3ccccc3)OC
568	Cc1nc(no1)Cc2nc(on2)CCOc3cccc(c3Cl)Cl
569	COc1ccccc1OCc2ccc(o2)c3nc(no3)c4ccncc4
570	C[C@@H](c1ncc(o1)C(C)(C)C)Sc2nnnn2C[C@@H]3CCCO3
571	Cc1c2cc(sc2n(n1)C)C(=O)OCCOc3cncc(n3)Cl
572	CCO[C@H](C)c1nc(on1)CO/N=C\2/CCCc3c2ccc(c3)OC
573	c1ccc2c(c1)cccc2CCCOC(=O)Cn3c(=O)c4ccnn4cn3
574	CCCCc1ccc(cc1)n2c(nnn2)SCC(=O)c3cnn(c3)C
575	CCO[C@H](C)c1nc(on1)CSc2nnc(n2C)c3ccccc3C
576	CCCn1c(nnn1)CSc2nnc(n2CC)c3ccccc3
577	CCc1c(nn(c(=O)c1C#N)Cc2nc(no2)CCC(C)C)CC
578	CC(C)(C)c1nc(on1)CCC(=O)Oc2ccc3ccc(=O)oc3c2
	CCc1nc(on1)CN(Cc2nc(no2)CC)[C@H]3CCCC[C@@H]3CC

579	COc1ccc(cc1)OCCCc2nc(no2)c3ccc(cn3)C#N
580	CCCOc1ccc(cc1)OCc2nc(no2)c3ccc(cn3)C#N
581	C[C@H](Cc1ccc(cc1)OC)CN(C)c2cc(nc3n2ncn3)COC
582	C=CCn1c(nnc1Sc2ccccc2[N+](=O)[O-])c3ccncc3
583	COC(=O)c1cc(cn(c1=O)CCC23CC4CC(C2)CC(C4)C3)[N+](=O)[O-]
584	CCOc1ccc(cc1OC)C\2=Nc3ncnn3/C2=N\Cc4ccccc4
585	CCCOc1ccc(cc1)OC(=O)c2ccnc(c2)n3cncn3
586	C/C(=C/C(=O)O[C@@H]1CCCC[C@H]1n2cc(nn2)C(=O)OC)/C3CC3
587	COc1ccc2c(c1)c(ccn2)Oc3cc(c(nc3)CC(=O)OC)OC
588	c1ccc(cc1)OCc2nnc(n2Cc3ccco3)SCC(=O)Nc4ccc(cc4)S(=O)(=O)N
589	c1cc(cc(c1)Cl)NC(=S)N/N=C/c2ccc(o2)c3ccc(cc3)S(=O)(=O)Nc4ncccn4
590	CC(C)c1ccc(cc1)/C=C/C(=O)NC(=S)Nc2ccc(cc2)S(=O)(=O)Nc3c(nccn3)OC
591	CCC(=O)Nc1cc(ccc1C)NC(=O)CSc2nnc(n2C)c3ccc(cc3)NS(=O)(=O)C
592	Cc1ccc(cc1)NS(=O)(=O)c2ccc(cc2)NC(=O)CCNS(=O)(=O)c3ccc(cc3)OC
593	CCc1ccccc1Nc2nc(nc(n2)N)CSc3nnc(n3C[C@H]4CCCO4)c5cccs5
594	CCN(CC)S(=O)(=O)c1cccc(c1)c2nnc(n2N)SCC(=O)Nc3ccc(cn3)Cl
595	Cc1cc(ccc1OCC(=O)NC(=S)Nc2ccc(cc2)S(=O)(=O)Nc3nc(cc(n3)C)C)Cl
596	CCN(CC)S(=O)(=O)c1cccc(c1)NC(=O)CSc2nnc(n2N)c3ccc(cc3)Cl
597	CCc1nc2ccccc2n1CC(=O)NNC(=O)c3ccc(c(c3)S(=O)(=O)NCc4ccco4)Cl
598	CCC[C@@]1(C(=O)N(C(=O)N1)CC(=O)Nc2ccc(cc2)C(=O)Nc3ccccc3OC)c4ccccc4
599	Cc1ccc(cc1)S(=O)(=O)NCCc2nnc(s2)NS(=O)(=O)c3ccc(cc3)NC(=O)C
600	Cc1ccc2c(c1)occ2CC(=O)N(C)CC(=O)N(CC(C)C)c3c(n(c(=O)[nH]c3=O)CC(C)C)N
601	c1ccc2c(c1)cccc2Cn3cc(cn3)NC(=S)Nc4ccc(cc4)S(=O)(=O)Nc5ncccn5
602	COc1cc(cc(c1)OC)NC(=O)CCSc2nc([nH]n2)N/N=C/c3cc(cc(c3[O-])Cl)Cl
603	Cc1cccc(c1)[N-]S(=O)(=O)c2ccc(cc2)NC(=O)CCSc3nnc(n3N)C(F)(F)F
604	Cc1c(c(n(n1)C(C)C)C)/C=N/Nc2[nH]nc(n2)SCC(=O)NCc3ccc(c(c3)Cl)Cl
605	CC[C@H](C)Sc1nnc(s1)NC(=O)CSc2nc([nH]n2)N/N=C/c3cccc4c3cccc4
606	COC(=O)c1c2c(sc1NC(=O)CCSc3nc([nH]n3)N/N=C/c4cccc5c4cccc5)CCC2
607	COCc1nnc(n1N)SCCC(=O)Nc2ccc(cc2)S(=O)(=O)[N-]c3cccc(c3)Cl
608	CCCSc1nnc(s1)/N=C(/CSc2nnc(n2N)N/N=C/c3c4ccccc4ccc3O)\[O-]
609	CCSc1nnc(s1)/N=C(/CSc2nnc(n2N)N/N=C/c3c4ccccc4ccc3[O-])\[O-]
610	CC[C@H](C)Sc1nnc(s1)NC(=O)CSc2nc([nH]n2)N/N=C/c3ccc(cc3Cl)Cl
611	CC[C@@H](C)Sc1nnc(s1)NC(=O)CSc2nc([nH]n2)N/N=C/c3cccc(c3Cl)Cl
612	CCCCn1c(=O)c2c(c(sc2nc1SCC(=O)c3c(n(c(=O)[nH]c3=O)CCOC)N)C)C
613	c1ccc2c(c1)C(=O)N(C2=O)CCCCCC(=O)Nc3nc4c(c(n3)[O-])ncn4[C@H]5[C@H]([C@@H]([C@@H](O5)CO)O)O
614	CCOCCCNC(=O)c1ccc(c(c1)NC(=O)Nc2cccc(c2)OC)N3C[C@H]4C[C@@H](C3)c5cccc(=O)n5C4
615	Cc1c(sc(n1)C)C(=O)C2=C(C(=O)N([C@H]2c3ccc(cc3)OCC(=O)N)CCCn4cc[nH+]c4)[O-]
	CC(=O)OC[C@@H]([C@H]1CC[C@@]2([C@@]1(CC[C@@H]3[C@@H]2CC[C@@]4([C@@]3(CC[C@@H](C4)OC(=O)C)COC(=O)C)O)C)O)O

616	Cc1ccccc1CN(c2ccc(cc2)C(=O)N/N=C/c3ccc(cc3)OCC(=O)N)S(=O)(=O)C
617	CC(C)CN(CC(C)C)S(=O)(=O)c1ccc(cc1)C(=O)Nc2c(ccs2)C(=O)NNC(=O)C
618	Cc1cc(nc(n1)N/C(=N\CCc2c[nH]c3c2cc(cc3)Cl)/NC(=O)CSc4nnc(s4)C)C
619	c1cc(oc1)/C=N/Nc2[nH]nc(n2)SCC(=O)Nc3nnc(s3)SCc4ccc(cc4)Cl
620	C/C(=N/Nc1[nH]nc(n1)SCC(=O)Nc2cccc(c2)Br)/c3ccc(cc3OC)OC
621	CCOCSc1nnc(s1)NC(=O)CSc2nnc(n2N)c3cccc(c3)Br
622	CCOCSc1nnc(s1)NC(=O)CSc2nnc(n2N)c3ccc(cc3)Br
623	C/C(=N/Nc1[nH]nc(n1)SCC(=O)Nc2ccc3c(c2)sc(n3)SCC=C)/c4cccnc4
624	Cc1cc(nc(n1)NS(=O)(=O)c2ccc(cc2)NC(=S)NC(=O)COc3ccc(cc3Cl)Cl)C
625	Cc1cc(c(c(c1NS(=O)(=O)C)C)S(=O)(=O)Nc2ccc(cc2)CC(=O)NCC(=O)OC)C
626	CC(C)CC[C@]1(C(=O)NC(=NC1=O)[O-])Cc2ccccc2CC3(C(=O)NC(=O)NC3=O)CCC(C)C
627	c1cc[nH]/c(=N\S(=O)(=O)c2ccc(cc2)/N=N/c3cc(c(c(c3)C(=O)[O-])O)c4ccc(cc4)S(=O)(=O)Nc5ccccn5)/c1
628	C[C@H](CCc1cc(cc(c1O)C[C@H](C(=C)C)O)c2c(c(=O)c3c(o2)cc(c(c3[O-])OC)O)OC)CO
629	CC(C)[C@H](C(=O)N[C@@H](CC(=O)[O-])[C@@H]1CCOC(C1)(C)C)NC(=O)CO/N=C/2\CC[C@@]3([C@@H]4CC[C@]5([C@@H]([C@@H]4CCC3=C2)CC[C@@]5(C#C)O)C)C
630	CC(C)[C@@H](C(=O)N[C@@H](CC(=O)[O-])CS)NC(=O)CO/N=C\1/CC[C@@]2([C@@H]3CC[C@]4([C@@H]([C@@H]3CCC2=C1)CC[C@@]4(C#C)O)C)C
631	c1ccc(c(c1)c2nnn(n2)CC(=O)NNC(=S)Nc3cc(cc(c3)Cl)Cl)Oc4ccc(cc4Cl)Cl
632	CCOc1cc(cc(c1OCC(=O)N(C)C)Br)/C=N\Nc2ccc(cc2)S(=O)(=O)N
633	Cc1cc(ccc1OCC(=O)NC(=S)Nc2ccc(cc2)S(=O)(=O)Nc3cc(on3)C)Br
634	Cc1ccc(cc1)C(=O)Oc2ccc(cc2)/C=N/NC(=O)C(=O)Nc3ccccc3C(=O)Nc4ccc(cc4)C
635	COc1ccc(cc1)c2c(cn(n2)c3ccccc3)/C=N/NC(=O)C(=O)Nc4ccccc4C(=O)Nc5ccc(cc5)Br
636	c1cc[nH+]c(c1)CN(CCNc2ccc(c(c2)C(=O)[O-])c3c4ccc(cc4oc-5cc(=O)ccc35)O)Cc6ccccn6
637	c1cc(cc(c1)Br)Nc2nc(nc(n2)NN)Nc3cccc(c3)Br
638	c1ccc(cc1)n2c(c(c(n2)/C(=C/c3ccc(cc3)/C=C(\C#N)/c4c(c(n(n4)c5ccccc5)N)C#N)/C#N)C#N)N
639	C[C@@]12CC[C@H]([C@@]([C@H]1Cc3c(nc(s3)NC(=O)C(C)(C)C)[C@H]2CC(=O)NC4CCCC4)(C)CO)O
640	c1cc2c(cc1NC(=S)NC(=O)/C=C/c3ccc(o3)c4ccc(cc4Cl)Cl)[nH]c(=O)[nH]2
641	Cc1cc(no1)NS(=O)(=O)c2ccc(cc2)Nc3nc(cs3)c4ccc(c(c4)O)O
642	Cc1cccc(c1C)n2c3c(cn2)c(nc(n3)SCC(=O)Nc4ccc(cc4)C(=O)N)O
643	Cc1ccccc1Nc2nc(nc(n2)N)CN3C(=O)[C@](NC3=O)(C)c4ccc5ccccc5c4
644	c1cc(ccc1c2c(c[nH]n2)[C@@H]3c4c(n[nH]c4OC(=C3C#N)N)c5ccc(c(c5)Cl)Cl)F
645	CCc1ccccc1Nc2nc(nc(n2)N)CN3C(=O)[C@](NC3=O)(C)c4ccc5ccccc5c4
646	c1ccc(cc1)Nc2c3c(c4c(n2)c(ns4)O)OC(=C([C@H]3c5ccc(cc5)Br)C#N)N
647	CCc1c(c(c(c(=O)n1c2ccccc2N)Sc3nnnn3C)N)c4ccc(cc4)Cl
648	Cc1ccc(cc1Cl)N/C(=N/c2[nH]c(cc(=O)n2)CSc3nc4ccccc4s3)/N
649	Cc1cccc(c1NC(=O)Cn2c(c(nn2)C(=O)Nc3ccc(cc3)Br)N)C
650	Cn1c2c(nc1Sc3nnc(n3N)N/N=C/c4c5ccccc5ccc4O)n(c(=O)n(c2=O)C)C
651	c1ccc(c(c1)CSc2nnc(n2N)N/N=C/c3cc(ccc3O)Br)Cl
652	Cc1cc(c(c(c1)C)NC(=O)Cn2c(c(c(n2)NC)c3nc(no3)c4cccc(c4)Cl)N)C

653<br>654<br>655<br>656<br>657<br>658<br>659<br>660<br>661<br>662<br>663<br>664<br>665<br>666<br>667<br>668<br>669<br>670<br>671<br>672<br>673<br>674<br>675<br>676<br>677<br>678<br>679<br>680<br>681<br>682<br>683<br>684<br>685<br>686<br>687<br>688<br>689	CCN(CC)c1c(c2c3c(nc(c2c(n1)N)N)N(C(=O)C3)c4ccc(c(c4)OC)OC)C#N<br>CCN(CC)c1ccc2c(c1)Oc3cc(ccc3C24c5ccc(c(c5C(=O)O4)N)N)N(CC)CC<br>C[C@]12CC[C@H]3[C@H]([C@]1(CC[C@@H]2C4=CC(=O)OC4)O)CC[C@@]5([C@@]3(CC[C@H](C5)O)/C=N/Nc6ccccc6)O<br>C[C@H](C(=O)N/[NH+]=C/c1cc(cc(c1[O-])Br)Br)Nc2c(=O)[nH]c(nn2)[O-]<br>Cc1cc(c(cc1NS(=O)(=O)c2ccc3c4c2cccc4C(=O)N3)Sc5[nH]ncn5)O<br>C[C@@H]1CCC[C@@H](N1c2[nH]c(=O)c3c(n2)NC(=O)C[C@@H]3C(=O)Nc4ccc(cc4)NC(=O)C)C<br>COc1ccc(cc1)C2=C[C@H](N=N2)[C@@H]3NN[C@@H](N3N)SCc4c(cccc4Cl)Cl<br>c1cc(cc(c1)Cl)Nc2[nH]c(=O)c3c(n2)NC(=O)C[C@@H]3C(=O)Nc4ccc(cc4Cl)Cl<br>Cc1cc(cc(c1)NC(=O)[C@@H]2CC(=O)Nc3c2c(=O)[nH]c(n3)Nc4cc(cc(c4)Cl)Cl)C<br>Cc1ccc(c(c1)NC(=O)[C@H]2CC(=O)Nc3c2c(=O)[nH]c(n3)Nc4ccc(cc4Cl)Cl)C<br>Cc1cccc(c1O)c2cc(n[nH]2)C(=O)Nc3ccc(cc3)S(=O)(=O)/N=c/4\[nH]ccs4<br>Cc1cc(c(c(c1)O)c2cc(n[nH]2)C(=O)Nc3ccc(cc3)S(=O)(=O)Nc4c(c(no4)C)C)C<br>Cc1ccc(nc1)Nc2c(c(ncn2)Nc3cc(c(cc3C)[C@H](C#N)c4ccc(cc4)Cl)Cl)N<br>c1cc(ccc1C(=O)NNc2c(c(ncn2)Nc3ccc(cc3F)Br)N)Br<br>c1cc2cccnc2c(c1)Sc3c(c(ncn3)NNC(=O)c4ccc(cc4)Br)N<br>c1cc(ccc1C(=O)Nc2nc3c(s2)CCC[C@@H]3C(=O)Nc4nc(cs4)CC(=O)N)Cl<br>c1cc(c(cc1NC(=O)[C@H]2CC(=O)Nc3c2c(nc(n3)N4CCCCC4)N)C(F)(F)F)Cl<br>c1cc(ccc1N/N=C\2/[C@@H](OC(=O)/C2=N\Nc3ccc(cc3)Br)[C@@H](CO)O)Br<br>CC1=C(C(=O)O[C@@H](C1)[C@@H](C)[C@H]2[C@@H](C[C@@H]3[C@@]2(CC[C@@H]4[C@@H]3[C@H]5[C@H](O5)[C@@]6([C@@]4([C@H](C[C@@<br>H](C6)O)O)C)O)C)O)C<br>C[C@]12CC[C@@H]([C@]([C@@H]1CC[C@@]3([C@H]2CC=C4[C@]3(CC[C@@]5([C@H]4CC(C[C@H]5O)(C)C)C)C)C)(C)CO)O[C@H]6[C@@H]([C@H]([<br>C@@H]([C@H](O6)C(=O)[O-])O)O)O<br>Cc1c(c(n[nH]1)O)[C@@H](c2ccc(c(c2)Br)OCc3cccc4c3cccc4)c5c(n[nH]c5O)C<br>Cn1c(c(c(=O)[nH]c1=O)c2c3c([nH]c2c4ccc(cc4)C5CCCCC5)n(c(=O)nc3[O-])C)N<br>C[C@@H]1[C@H]([C@@H]([C@@H]([C@@H](O1)O[C@H]2CC[C@]3([C@@H](C2)CC[C@@H]4[C@@H]3CC[C@]5([C@@]4(CC[C@@H]5C6=CC(=O)OC<br>6)O)C)C)O)O)O<br>C[C@]1(C(=O)N(C(=O)N1)CC(=O)Nc2ccc(cc2)S(=O)(=O)N)c3cccc(c3)Br<br>C[C@]12CC[C@@H](C([C@H]1CC[C@@]3([C@@H]2C=C[C@@H]4[C@]3(CC[C@@]5([C@@H]4CC(CC5)(C)C)C(=O)O)C)C)(C)C)O[C@@H]6[C@H]([C@<br>@H]([C@H](O6)CO)O)O<br>CCOc1ccc(cc1)c2c3c([nH]n2)OC(=C([C@@]34c5c(ccc(c5Br)C)NC4=O)C#N)N<br>C/C(=N/NC(=O)c1ccc(c(c1)S(=O)(=O)N)Cl)/c2ccc3c(c2)Nc4ccccc4S3<br>Cc1ccc2c(c1Br)[C@@]3(c4c(n[nH]c4OC(=C3C#N)N)c5cc(c(c(c5)OC)OC)OC)C(=O)N2<br>c1ccc(cc1)n2c(=O)c3c([nH]2)NC4=C([C@H]3c5ccc(c(c5)O)O)C(=O)C[C@@H](C4)c6cccs6<br>Cc1c(c(n[nH]1)O)[C@@H](c2cc(c(c(c2)I)OC(C)C)I)c3c(n[nH]c3O)C<br>c1ccc(cc1)c2cc(n[nH]2)c3nnc(n3N)SCC(=O)Nc4ccc(c(c4)Cl)Cl<br>C[C@@H]([C@H]1CC[C@@H]2[C@@]1(CC[C@@H]3[C@@H]2CC[C@@H]4[C@@]3(CC[C@H](C4)O[C@H]5[C@@H]([C@H]([C@@H]([C@@H](O5)C(=<br>O)[O-])O)O)O)C)C)O<br>c1cc(cc(c1)Cl)NC(=O)CSc2nnc(n2N)c3cc(n[nH]3)c4ccc(cc4)Cl

690	c1ccc2c(c1)cccc2NC(=O)CSc3nnc(n3N)c4cc(n[nH]4)c5ccc(cc5)Cl
691	Cc1ccc(cc1)/C=N/n2c(c(c3c2nc4ccccc4n3)C(=O)Nc5cc(ccc5C)C)N
692	CC1([C@@H](N(C(=S)S1)NC(=O)Nc2ccc(cc2)Cl)N(C(=O)Nc3cccc4c3cccc4)[O-])C
693	c1ccc2c(c1)c(cc(n2)O)C(=O)NNC(=O)c3ccc(cc3)[N-]S(=O)(=O)c4cccs4
694	Cc1c2c([nH]n1)OC(=C([C@@H]2c3ccc(c(c3)COc4ccc(cc4Cl)Cl)OC)C#N)N
695	CC1=C([C@@H](C(=C(N1)SCC(=O)N)C#N)c2ccco2)/C(=N/c3nc4ccccc4s3)/[O-]
696	c1cc(c(nc1)NC(=O)c2ccc(cc2)NS(=O)(=O)c3ccc(s3)Br)O
697	CC(=O)Nc1ccc(cc1)[C@H](/C(=N\NC(=O)c2ccncc2)/c3c(nc4cc(ccc4n3)Cl)[O-])O
698	Cc1ccc2c(c1)c(cc(n2)c3ccc(cc3)NC(=O)C)C(=O)Nc4ccc(cc4)NC(=O)C
699	Cc1c(c(n(n1)c2ccccc2)O)[C@H](c3ccc(c(c3)O)O)c4c(nn(c4[O-])c5ccccc5)C
700	C/C=C/1\C(=O)N[C@H](C(=O)O[C@H]\2CC(=O)N[C@@H](C(=O)N[C@H](CSSCC/C=C2)\C(=N1)\[O-])C(C)C)C(C)C
701	Cc1ccc(c(c1)[C@H](c2c([nH]c(=S)n(c2=O)C(C)C)[O-])[C@H]3[C@@H](N(C(=S)NC3=O)C(C)C)O)N(C)C
702	c1cc(c(cc1Cl)c2ccc(o2)[C@H]3c4c([nH]c(=O)[nH]c4=O)NC5=C3C(=O)CCC5)Cl
703	c1cc(oc1c2cc(cc(c2)Cl)Cl)[C@H]3c4c([nH]c(=O)[nH]c4=O)NC5=C3C(=O)CCC5
704	c1ccc(c(c1)C(=O)N)[N-]C2=NC(=O)/C(=C/3\c4cc(ccc4NC3=O)Br)/S2
705	c1cc(c(cc1Cl)Cl)Nc2nc3c(c(n2)[O-])[C@H](CC(=O)N3)C(=O)NC4CCCCC4
706	[H]/N=C/1\Nc2nc3ccccc3n2[C@H](N1)c4ccc(cc4)N5[C@@H](CC(=N5)c6ccccc6)c7ccccc7
707	CCCc1c(nnn1c2c(non2)N)C(=O)N/N=C\3/c4cc(ccc4N=C3[O-])Br
708	CC1(CC2=C(C(=O)C1)[C@@]3(c4ccccc4NC3=O)N=C(N2)Nc5nc6ccccc6s5)C
709	c1cc(ccc1N2CCN(CC2)c3c(c(ncn3)Nc4c(cc(cn4)C(F)(F)F)Cl)N)F
710	Cc1c2c([nH]n1)OC(=C([C@H]2c3ccc(c(c3)CSc4nc5ccccc5s4)OC)C#N)N
711	c1cc(ccc1c2nnc(n2N)SCC(=O)N/N=C/c3cc(ccc3[O-])Cl)Br
712	CCCN1c2ccccc2[C@@H](C(=C1[O-])/C(=N/N=C(\C)/[C@@H]3C(=c4ccccc4=[NH+]C3=O)O)/[O-])O
713	CC[C@H]1CC2=c3ccccc3=[NH+][C@H]2[C@@H](N1)c4c([nH]c(=S)n(c4=O)c5ccc(cc5)OC)[O-]
714	c1ccc(cc1)C(=O)N[C@H]2Nc3cc(ccc3S2)NC(=O)[C@H]4C=C(N=N4)c5cccs5
715	C[C@@H]1CC[C@@]2(CC[C@@]3(C(=CC[C@@H]4[C@]3(CC[C@H]5[C@@]4(CC[C@@H](C5(C)C)O[C@H]6[C@@H]([C@H]([C@@H]([C@H](O6)C)O)O
716	)O)C)C)[C@@H]2[C@H]1C)C(=O)[O-])C(=O)[O-]
717	C[C@@H]1[C@H]([C@@H]([C@H]([C@@H](O1)O[C@H]2CC[C@]3([C@@H](C2(C)C)CC[C@@]4([C@H]3CC=C5[C@]4(CC[C@@]6([C@@H]5CC(CC6)(C
718	)C)C(=O)[O-])C(=O)[O-])C)C)O)O)O
719	C[C@@H]1CC[C@@]2(CC[C@@]3(C(=CC[C@@H]4[C@]3(CC[C@H]5[C@@]4(CC[C@@H](C5(C)C)O[C@H]6[C@@H]([C@H]([C@H]([C@H](O6)C)O)O)O
720	)C)C)[C@@H]2[C@H]1C)C(=O)[O-])C(=O)[O-]
721	C[C@@H]1CC[C@@]2(CC[C@@]3(C4=CC[C@H]5[C@@]([C@H]4CC[C@]3([C@@H]2[C@H]1C)C)(CC[C@@H](C5(C)C)O[C@H]6[C@@H]([C@H]([C@@
722	H]([C@H](O6)C(=O)[O-])O)O)O)C)C)C(=O)O
723	C[C@]12CC[C@@H]([C@@]([C@H]1CC[C@@]3([C@H]2CC=C4[C@]3(C[C@H]([C@@]5([C@H]4CC(CC5)(C)C)C(=O)[O-])O)C)C)(C)C(=O)[O-
724	])O[C@H]6[C@@H]([C@H]([C@@H](CO6)O)O)O
725	C[C@@H]1CC[C@@]2(CC[C@@]3(C(=CC[C@H]4[C@]3(CC[C@@H]5[C@@]4(CC[C@@H](C5(C)C)O[C@H]6[C@@H]([C@@H]([C@H]([C@@H](O6)C)O
726	)O)O)C)C)[C@@H]2[C@H]1C)C(=O)[O-])C(=O)O
	COc1ccc2c(c1)c3c([nH]2)[C@@H](NCC3)c4c(=O)[nH]c(=O)n(c4[O-])c5ccc(cc5)I

727	c1ccc2c(c1)N/C(=C\3/C(=O)NC(=O)N(C3=O)c4ccc(cc4)Cl)/C[C@H](S2)c5cccc(c5)O
728	CSc1ccc(cc1)c2ccc3c(c2)C(=O)N4CC[C@@H](C[C@H]4C(=O)N3)NC(=O)[C@@H]5CSCN5
729	COc1cccc(c1OC)[C@@H]2c3c(n[nH]c3OC(=C2C#N)N)c4ccc(cc4)Br
730	C[C@]1(C(=O)NC(=O)N1)c2cccc(c2)NC(=O)c3c(nc(s3)c4ccccc4)c5ccccc5
731	c1cc(cc(c1)Br)S(=O)(=O)Nc2cc(cc(c2)C(F)(F)F)c3[nH]c(=O)[nH]n3
732	COc1ccc(c(c1)[C@H]2c3c(n[nH]c3OC(=C2C#N)N)c4ccc(c(c4)Cl)Cl)OC
733	Cc1cccc(c1)N2C3=NC(=O)[C@](C3=C(NC2=O)[O-])(C(F)(F)F)NS(=O)(=O)c4ccc(cc4)N
734	c1ccc(c(c1)N2C3=NC(=O)[C@](C3=C(NC2=O)[O-])(C(F)(F)F)[N-]S(=O)(=O)c4ccc(cc4)N)F
735	c1ccc(c(c1)N2C3=NC(=O)[C@@](C3=C(NC2=O)[O-])(C(F)(F)F)[N-]S(=O)(=O)c4ccc(cc4)N)F
736	Cc1nc(n(n1)CC(=O)Nc2c(c3c(s2)CCC3)C(=O)N)c4ccc(c(c4)Cl)Cl
737	c1cc(sc1)S(=O)(=O)[N-]c2ccc(cc2)C(=O)NNC(=O)c3cc(ccc3O)Cl
738	c1ccc2cc(ccc2c1)c3cc(n[nH]3)C(=O)N/N=C/4\c5cc(ccc5NC4=O)Br
739	c1ccc(cc1)[C@H]2CC3=C([C@@H](C(=C(N3c4[nH]ncn4)N)C#N)c5ccc(cc5)Br)C(=O)C2
740	CC\1=NNC(=O)/C1=N/Nc2cc(ccc2Cl)C(=O)Nc3cc(c(cc3Cl)Cl)Cl
741	c1c(cc(c(c1C(=O)Nc2cc([nH]n2)C(=O)[O-])O)I)I
742	Cc1ccc2c(c1)[C@]3(c4c(nc(nc4[O-])SCc5cccc(c5)F)NC(=C3C#N)N)C(=O)N2
743	COc1ccc(cc1OC)[C@@H]2CC3=C([C@@H](c4c([nH]nc4O)N3)c5ccc(cc5Cl)Cl)C(=O)C2
744	COc1ccc(cc1OC)[C@@H]2CC3=C([C@H](c4c([nH]nc4O)N3)c5ccc(cc5)SC)C(=O)C2
745	C[C@H]1CCCCN1Cc2c(nnn2c3c(non3)N)C(=O)N/N=C\c4cc(c(c(c4)Br)[O-])Br
746	c1ccc(cc1)C(=O)C(CCc2nnc(s2)N)(CCc3nnc(s3)N)CCc4nnc(s4)N
747	c1cc(oc1)CNS(=O)(=O)c2ccc-3c(c2)C(=NNC(=S)N)c4c3ccc(c4)S(=O)(=O)NCc5ccco5
748	CCC(C)(C)NC(=O)[C@@H](c1ccc(cc1)OCC)N(Cc2ccco2)C(=O)c3c(c(ns3)C(=O)N)N
749	CCOC(=O)CNC(=O)[C@@H](c1cccs1)N(c2cccc(c2C)C)C(=O)c3c(c(ns3)C(=O)N)N
750	CCOC(=O)CNC(=O)[C@H](c1cccs1)N(c2ccccc2OC)C(=O)c3c(c(ns3)C(=O)N)N
751	CCc1ccc(cc1)N([C@H](c2ccc(cc2)N(C)C)C(=O)NCCOC)C(=O)c3c(c(ns3)C(=O)N)N
752	CCc1ccccc1N([C@@H](c2ccc(o2)C)C(=O)NCC(=O)OCC)C(=O)c3c(c(ns3)C(=O)N)N
753	CN(C)c1ccc(cc1)[C@@H](C(=O)NCCOC)N(c2ccccc2F)C(=O)c3c(c(ns3)C(=O)N)N
754	COCCNC(=O)[C@@H](c1ccc(cc1)OC)N(Cc2cccs2)C(=O)c3c(c(ns3)C(=O)N)N
755	Cc1ccc(cc1)N([C@H](c2ccc(cc2)N(C)C)C(=O)NCCOC)C(=O)c3c(c(ns3)C(=O)N)N
756	Cc1cccc(c1C)N([C@H](c2ccc(cc2)N(C)C)C(=O)NCCOC)C(=O)c3c(c(ns3)C(=O)N)N
757	Cc1ccc(cc1)[C@@H](C(=O)NC(C)(C)C)N(c2ccc(c(c2)OC)OC)C(=O)c3c(c(ns3)C(=O)N)N
758	Cc1ccc2c(c1)/C(=N\O[C@@H]3[C@H]([C@@H]([C@H]([C@H](O3)CO)O)O)NC(=O)C)/C(=O)N2CCOc4ccccc4C
759	Cc1ccc(c(c1)C)NS(=O)(=O)c2cccc(c2)C(=O)NNS(=O)(=O)c3ccc(cc3)NC(=O)C
760	c1cc2cccc3c2c(c1)C(=O)N(C3=O)CCCC(=O)OCC(=O)Nc4cc(cc(c4)C(=O)N)C(=O)N
761	CCN(CC)c1ccc(cc1)/C=N/Nc2nnc(n2N)SCC(=O)Nc3cc(ccc3OC)Cl
762	Cc1cccc(c1)NS(=O)(=O)c2ccc(cc2)NC(=O)CCSc3nnc(n3N)c4cccnc4
763	Cn1cc(cn1)/C=N/Nc2[nH]nc(n2)SCC(=O)Nc3ccc(cc3)S(=O)(=O)NC4CCCCC4
	COCCSc1nnc(s1)NC(=O)CSc2nc([nH]n2)N/N=C/c3c4ccccc4ccc3O

764	Cc1c(c(n(n1)C(C)C)C)/C=N/Nc2nnc(n2N)SCC(=O)Nc3nnc(s3)SCC(C)C
765	Cc1c(c(n(n1)C(C)C)C)/C=N/Nc2nnc(n2N)SCCC(=O)Nc3ccc(cc3)Br
766	Cc1c(c(n(n1)C(C)C)C)/C=N/Nc2nnc(n2N)SCCC(=O)Nc3c(cccc3Cl)Cl
767	c1ccc2c(c1)cccc2/C=N/Nc3nnc(n3N)SCCC(=O)Nc4cccc(c4)N5CCCC5=O
768	c1ccc2c(c1)cccc2/C=N/Nc3nnc(n3N)SCCC(=O)Nc4ccc(cc4)S(=O)(=O)N
769	Cc1cc(ccc1NC(=O)CSc2nnc(n2N)N/N=C/c3ccc(cc3)C(=O)OC)Br
770	COC(=O)c1ccc(cc1)/C=N/Nc2nnc(n2N)SCC(=O)NCc3ccc(c(c3)Cl)Cl
771	CC(=O)Nc1cccc(c1)NC(=O)CCSc2nnc(n2N)N/N=C/c3c4ccccc4ccc3O
772	Cc1ccc(cc1)/C=N/Nc2nnc(n2N)SCC(=O)Nc3ccc(cc3)S(=O)(=O)NC(C)(C)C
773	COc1cc(ccc1Cl)NC(=O)CSc2nnc(n2N)N/N=C/c3cc(c(c(c3)OC)OC)OC
774	COc1cc(cc(c1OC)OC)/C=N/Nc2nnc(n2N)SCCC(=O)Nc3ccc(cc3)Cl
775	Cc1ccc(cc1)NS(=O)(=O)c2ccc(cc2)NC(=O)CSc3nc([nH]n3)N/N=C/c4ccccc4
776	c1cc(ccc1NC(=O)CSc2nnc(n2N)N/N=C/c3cc(ccc3O)Cl)OC(F)(F)F
777	CCOc1ccc(cc1)NC(=O)CSc2nnc(n2N)N/N=C/c3cc(ccc3OC)Br
778	C/C(=N\Nc1nnc(n1N)SCC(=O)Nc2ccccc2[N+](=O)[O-])/c3ccc(cc3)Br
779	CCN(CC)c1ccc(c(c1)O)/C=N/Nc2nnc(n2N)SCC(=O)Nc3nc4ccccc4s3
780	c1ccc(c(c1)CSc2nnc(s2)NC(=O)CSc3nnc(n3N)NN=C4CCCC4)Cl
781	CC(=O)OC[C@@]12[C@H](C[C@@H](C[C@@]1(CC[C@@H]3[C@@H]2[C@@H](C[C@@]4([C@@]3(CC[C@@H]4C(=O)O)O)C)OC(=O)C)O)OC(=O)C)O
782	CC(=O)OC[C@]12[C@H](C[C@@H](C[C@@]1(CC[C@@H]3[C@@H]2[C@@H](C[C@@]4([C@@]3(CC[C@@H]4C(=O)O)O)C)OC(=O)C)O)OC(=O)C)O
783	Cc1ccc(o1)[C@H](C(=O)NC[C@H]2CCCO2)N(c3ccc(cc3)C(C)C)C(=O)c4c(c(ns4)C(=O)N)N
784	CC1=C[C@](N(N1)C(=O)CSc2nnc(s2)SCC(=O)N3[C@@](CC(=C)N3)(c4ccccc4)O)(c5ccccc5)O
785	c1cc(ccc1c2[nH]c3ccc(cc3n2)NC(=O)CSc4cn[nH]n4)NC(=O)CSc5cn[nH]n5
786	Cc1cc(no1)NS(=O)(=O)c2ccc(cc2)N/N=C(\C#N)/C(=O)Nc3ccc(cc3)S(=O)(=O)N
787	C/C(=C\CO[C@@H]1[C@@H]([C@@H]([C@@H](CO1)OC)O)O)/CC[C@@H]2C(=C)CC[C@@H]3[C@@]2(CC[C@@H](C3(C)C)O[C@@H]4[C@@H]([C@
788	@H]([C@@H](O4)CO)O)O)C
789	C[C@H]1[C@H]([C@@H](C[C@@H](O1)O[C@@H]2CC[C@@]3([C@H]4CC[C@@]5([C@@H](CC[C@@]5([C@@H]4CC[C@@]3(C2)O)O)C6=CC(=O)OC6
790	)C)/C=N/CCCO)OC)O
791	CCCCCCCCC[C@@H]1[C@H]([C@@H]([C@@H](C(=O)N[C@@H](C(=O)N[C@@H](C(=O)N[C@@H](C(=O)O1)CO)[C@@H](C)O)C(C)C)C)O)C
792	COc1c/c(=N/S(=O)(=O)c2ccc(cc2)NC(=S)Nc3ccc(cc3)S(=O)(=O)N)/[nH]c(n1)OC
793	COc1cc(cc(c1)OC)NC(=O)c2c(c3c(c(sc3s2)C(=O)Nc4cc(cc(c4)OC)OC)N)N
794	CC1=C([C@@H](C(=C(N1)SCC(=O)Nc2nc3ccccc3s2)C#N)c4ccco4)C(=O)Nc5ccc(cc5)S(=O)(=O)N
795	CC1=C([C@@H](C(=C(N1)SCC(=O)NC2CCCCC2)C#N)c3ccco3)C(=O)Nc4ccc(cc4)S(=O)(=O)N
796	c1cc(ccc1Cc2ccc(cc2)NC(=O)NNC(=O)c3ccncc3)NC(=O)NNC(=O)c4ccncc4
797	c1ccc(cc1)NC(=O)C(=O)N/N=C/c2c3ccccc3ccc2O
798	c1cc(cc(c1)O)N2C(=O)c3ccc(cc3C2=O)Oc4ccc(cc4)N
799	c1cc(cc(c1)O)/C=N/NC(=O)c2cc(n[nH]2)c3ccc(cc3)Cl
800	Cc1ccc(c(c1)C)OCC(=O)NNC(=O)Nc2ccc(cc2)Cl
	Cc1ccc(cc1)NC(=O)Nc2nc(cs2)CC(=O)NC(C)(C)C

801	c1ccc(cc1)Nc2nc(nc(n2)N)/C(=C/c3ccccc3Cl)/C#N
802	Cc1cc(ccc1O[C@@H](C)C(=O)Nc2ccc3c(c2)[nH]c(=O)[nH]3)Cl
803	Cc1ccc(cc1)c2nnc(n2N)SCc3[nH]c4ccccc4n3
804	Cc1cc(cc(c1)NC(=O)NNC(=O)c2ccc(cc2)n3cccc3)C
805	c1cc(ccc1CSc2nnc(n2N)NN=C3CCCC3)Cl
806	Cc1ccnc(c1)Nc2c(c(ncn2)Oc3cc(cc(c3)C)C)N
807	c1ccc2cc(ccc2c1)Oc3c(c(ncn3)NCc4ccco4)N
808	CCN(c1ccccc1)c2c(c(ncn2)Nc3ccc(cn3)C)N
809	Cc1cccc2c1nc(s2)Nc3c(c(ncn3)N4CCCC4)N
810	Cc1ccc2cccc(c2n1)Nc3c(c(ncn3)N4CCCC[C@@H]4C)N
811	CC[C@H]1CCCCN1c2c(c(ncn2)Nc3nc(c(s3)C)C)N
812	c1cc2cccnc2c(c1)Oc3c(c(ncn3)NC4CCCCCC4)N
813	Cc1ccc(c(c1)C)Nc2c(c(ncn2)n3cnc4c3cccc4)N
814	Cc1ccc2cccc(c2n1)Nc3c(c(ncn3)Oc4ccccc4)N
815	c1ccc2c(c1)ncn2c3c(c(ncn3)Nc4ccc(cc4)Cl)N
816	c1ccc2c(c1)CCCN2c3c(c(ncn3)Nc4nccs4)N
817	c1ccc2c(c1)CCCN2c3c(c(ncn3)Nc4ccccn4)N
818	Cc1ccc(cc1)Oc2c(c(ncn2)Nc3cccnc3Cl)N
819	CCNc1c2ccccc2nc(n1)Nc3ccc(cc3)NC(=O)C
820	C[C@@H]1CCCC[C@@H]1NC(=O)Nc2ccc(cc2)NC(=O)c3ccco3
821	Cc1ccc(cc1)[C@H]2C[C@@H](n3c(nc(n3)N)N2)c4ccc(cc4)OC
822	C[C@H]1C(=C(NN1C(C)(C)C)C)[C@@H]2[NH+]=c3ccc(cc3=[NH+]2)C(=O)[O-]
823	Cc1ccc(cc1)Nc2c(n[nH]n2)C(=O)Nc3c(cccc3C)C
824	Cc1cccc(c1Oc2c(cccn2)CNc3cc(nc(n3)N)C)C
825	C[C@H](c1ccc(s1)Cl)NC(=O)Nc2ccc3c(c2)C(=O)NC3=O
826	Cc1c(c(n[nH]1)O)CCC(=O)N/N=C\c2ccc(c(c2)Cl)Cl
827	COc1ccc(cn1)c2cc(c(c(n2)N)C#N)c3ccc4c(c3)cc[nH]4
828	Cc1c(c(on1)C)[C@@H](C)NC(=O)NNc2ccc(cc2Cl)Cl
829	Cc1cc(nc(n1)c2ccccc2O)N/N=C\c3ccccc3O
830	Cc1cc(nc(n1)SCc2cc(=O)oc3c2ccc(c3C)O)N
831	CC(C)c1ccc(cc1)S(=O)(=O)Nc2ccc3c(c2)[nH]c(=O)[nH]3
832	Cc1csc2c1c(=O)[nH]c(n2)c3cccc(c3)NC(=O)NC(C)C
833	CC(C)(C)c1cs/c(=N\S(=O)(=O)c2cc(c(nc2)N)Cl)/[nH]1
834	c1cc(cnc1)c2c(cn[nH]2)CNc3cc([nH]n3)C4CCCCCC4
835	c1ccc(cc1)NC(=O)Nc2ccc(cc2)c3ccnc4c3cn[nH]4
836	Cc1ccc(cc1NC(=O)NCc2cc3ccccc3[nH]2)c4ncco4
837	c1ccc2c(c1)c(ccn2)NNC(=O)c3cc(n[nH]3)C4CCCCC4
	Cc1cc(c2cc([nH]c2c1)C(=O)NNc3cc(cc(n3)Cl)C#N)C

838	CC(C)(C)c1c(scn1)NC(=O)Nc2ccc3c(c2)CC(=O)N3
839	c1ccc2c(c1)c(nc(n2)c3ccco3)Nc4ccc(cc4)C(=O)N
840	c1ccc2c(c1)ccc(c2/C=N\NC(=O)c3c4c([nH]n3)CCC4)O
841	c1ccc(cc1)Nc2nc(nc(n2)N)/C(=C\c3cccc(c3)F)/C#N
842	Cc1cccc2c1oc(c2C)C(=O)Nc3c4ccccc4oc3C(=O)N
843	Cc1ccc(cc1F)Cc2cnc(s2)NC(=O)Nc3c[nH]nc3
844	CCNc1c(c(nc(n1)N)Cl)/N=N/c2ccc(cc2)Cl
845	CCOc1ccc(cc1)S(=O)(=O)NCCC(=O)OCC(=O)Nc2c(c(c(cn2)Cl)C)Cl
846	COc1cc(cc(c1OC)OC)/C=C/C(=O)Nc2nnc(s2)SCC(=O)NC[C@H]3CCCO3
847	C=CCN(c1ccccc1)S(=O)(=O)c2cccc(c2)C(=O)OCC(=O)NC(=O)NCc3ccco3
848	CCSc1nc(c2cnn(c2n1)CCNC(=O)c3cc(c(c(c3)OC)OC)OC)NC(C)C
849	CCSc1nc(c2cnn(c2n1)CCNC(=O)COc3ccc(cc3)Cl)NCCOC
850	CCSc1nc(c2cnn(c2n1)CCNC(=O)[C@H]3CC(=O)N(C3)c4ccc(cc4)C)NCCOC
851	CCOC(=O)Cc1csc(n1)NC(=O)CSc2c3c(n(c(=O)n2)CCCO)CCCC3
852	COCCCn1c(nnc1SCCC(=O)NC(=O)NCc2ccccc2)c3ccccc3Cl
853	CCOc1ccc(cc1)NC(=O)CSc2nnc(o2)CNC(=O)COc3ccc(cc3)Cl
854	CNC(=O)c1c2c(sc1NC(=O)c3ccc(cc3)S(=O)(=O)N(CCOC)CCOC)CCCCC2
855	CCOc1ccc(cc1)NC(=S)N(Cc2ccco2)Cc3cc4cc(c(cc4[nH]c3=O)OC)OC
856	COCCNC(=O)c1c2c(sc1NC(=O)COC(=O)COc3ccc4ccccc4c3)CCCC2
857	CCCOc1ccc(cc1C(=O)NC(=S)N2CCNC(=O)[C@@H]2CC(=O)OCCC)Br
858	CCCCOC(=O)C[C@H]1C(=O)NCCN1C(=S)NC(=O)c2cc(ccc2OCC)Br
859	CSCCCNc1cc(cc(c1)S(=O)(=O)N2CCCC2)C(=O)NCCCn3ccnc3
860	CCCCCOC(=O)C[C@H]1C(=O)NCCN1C(=S)NC(=O)c2ccc(c(c2)Br)OC(C)C
861	CCCCCOC(=O)C[C@H]1C(=O)NCCN1C(=S)NC(=O)c2ccccc2OCCOC
862	CC(C)n1cc(cn1)/C=N/NC(=O)CSc2nnc(n2CC=C)CNc3ccc(cc3)Cl
863	CCCCCCc1nc2c(c(n1)S[C@@H](C)C(=O)NC(=O)NC3CC3)c(=O)n(c(=O)n2CCC)C
864	CCOc1ccc(cc1OCC)C(=O)NCC(=O)O[C@H](C)C(=O)Nc2ccc(cc2)C(F)(F)F
865	CN(CCc1nc(on1)c2ccc(cc2)C(=O)NCCCOC)C(=O)Nc3ccccc3OC
866	CCN(Cc1[nH]c(=O)c2cc(c(cc2n1)OC)OC)CC(=O)NCc3ccccc3OC(F)(F)F
867	CCOCCOc1nc(n(n1)c2cccc(c2)NC[C@@H](COC(C)C)O)c3ccc(cc3)OC
868	CCOC(=O)c1c(c2c(nc(nc2s1)C[NH+](CCCOC)C[C@@H](COC(C)(C)C)O)[O-])C
869	CC(C)C[NH+](Cc1nc(c2c(csc2n1)c3ccc(c(c3)OC)OC)[O-])C[C@H](COC(C)C)O
870	CC(C)[NH+](Cc1nc(c2c(csc2n1)c3ccc(c(c3)OC)OC)[O-])C[C@@H](COCC#C)O
871	CC(C)[NH+](Cc1nc(c2c(csc2n1)c3ccc(c(c3)OC)OC)[O-])C[C@H](COCC#C)O
872	COc1ccc(cc1OC)c2csc3c2c(=O)[nH]c(n3)CN(C[C@H](COc4ccccc4)O)C5CC5
873	COc1ccc(cc1)Nc2nc(cs2)C(=O)N(CCC(=O)NC[C@@H]3CCCO3)Cc4ccco4
874	COCCNC(=O)CCN(Cc1ccco1)C(=O)c2csc(n2)Nc3cccc(c3Cl)Cl
	CCC(=O)Nc1ccc(cc1OCC)C(=O)CSc2nnc(o2)CNc3ccc(cc3)Cl

875	C[C@@H](c1nnc(n1CC=C)SCC(=O)Nc2cccc(c2)C(=O)OC)NC(=O)c3cccs3
876	Cc1ccc(cc1)N([C@H](c2ccc(cc2)F)C(=O)NCCOC)C(=O)CCC(=O)Nc3nccs3
877	CCOc1ccc(cc1OCC)C(=O)NCCn2c3c(cn2)c(ncn3)NCc4ccc(cc4)F
878	CCCNC(=O)CSc1nnc(s1)NC(=O)c2c(cn(n2)c3ccc(cc3)F)OCCC
879	Cc1cccc(c1)N2CCN(CC2)CCCCNC(=O)c3c(c(c([nH]3)C)C(=O)OCCOC)C
880	CCCC(=O)Nc1ccc(c(c1)C(=O)C)OCC(=O)NCc2ccccc2S(=O)(=O)N(C)C
881	CCOc1ccc(cc1)[C@H](CCC(=O)N/N=C\c2ccc(c(c2)Br)OCC(=O)OC)O
882	Cc1ccc(cc1)C(=O)NCCc2nnc(n2CC=C)SCC(=O)Nc3ccc(cc3)OC
883	CC[C@H](C)Sc1nnc(s1)NC(=O)CSc2nnc(n2CC=C)CNc3ccccc3
884	CCN(CC)C(=O)c1c(c(c(s1)NC(=S)Nc2ncn(n2)Cc3ccccc3C)C(=O)OC)C
885	CCN(CC)S(=O)(=O)c1ccc(c(c1)/N=C(/C[NH+](C)CC(=O)Nc2ccc(cc2)OC)\[O-])C
886	CCOc1ccc(cc1OCC)S(=O)(=O)N2CCN(CC2)CCOc3ccc(cc3)S(=O)(=O)N
887	CCn1c(c(c(n1)C)CN/C(=N\C(=O)c2cc(c(c(c2)OC)OC)OC)/Nc3ccc(cc3)OC)C
888	CC[NH+](CC)CCCN1[C@H](C(=C(C1=O)[O-])C(=O)c2c(c([nH]c2C)C(=O)OC)C)c3ccccc3F
889	CCOc1ccc(cc1)[C@@H]2C(=C(C(=O)N2CCCn3cc[nH+]c3)[O-])C(=O)c4c(c([nH]c4C)C(=O)OC)C
890	CCCN(CCC)C(=O)CCC(=O)Nc1ccc(c(c1)Cl)NC(=O)[C@@H](C)n2c(cc(n2)C)C
891	Cc1ccccc1OC[C@@H](CN(CCOC)Cc2[nH]c(=O)c3c(c(sc3n2)C(=O)OC)C)O
892	COc1ccccc1c2csc3c2c(=O)[nH]c(n3)CN(Cc4ccco4)C[C@H](COCC=C)O
893	CCCC(=O)OC[C@H](CN(CC=C)Cc1[nH]c(=O)c2c(c(sc2n1)C(=O)OC(C)C)C)O
894	CCCCC(=O)NC(C(F)(F)F)(C(F)(F)F)Nc1ccc(cc1)S(=O)(=O)Nc2c(con2)C
895	CCOc1ccc(cc1OCC)C(=O)Nc2nnc(s2)SCC(=O)Nc3cccc(c3)NC(=O)C
896	CCOc1ccc(cc1OCC)NC(=O)COC(=O)[C@@H]([C@H](C)O)NS(=O)(=O)c2ccc(cc2)C
897	CCOCCOc1ccc(cc1)C(=O)NC(=S)Nc2ccccc2C(=O)Nc3ccc(c(c3)C)C
898	CCOc1ccc(cc1)NS(=O)(=O)c2ccc(cc2)NC(=S)NC(=O)COc3cccc(c3)C
899	c1ccc2c(c1)cccc2OCC(=O)NNC(=S)NC(=O)CCCOc3ccc(cc3Cl)Cl
900	Cc1cccc(c1)c2nnc(n2CC(=O)N)SCC(=O)NCCC(c3ccccc3)c4ccccc4
901	CC(C)Oc1ccc(cc1Br)C(=O)NC(=S)Nc2ccccc2C(=O)NCCCOC
902	CCOCCOc1ccc(cc1)C(=O)NC(=S)Nc2cccc(c2)C(=O)Nc3cccc(c3)Cl
903	CC(C)(C)c1ccc(cc1)C(=O)Nc2ccc(cc2)NC(=S)NC(=O)c3ccc(cc3)OCCOC
904	CCCOc1ccc(cc1)C(=O)NC(=S)NNC(=O)COc2ccc(cc2Br)CC
905	CC(C)CCOc1ccc(cc1)C(=O)NC(=S)NNC(=O)COc2ccc(cc2)Br
906	CCOc1cc(cc(c1OCC)OCC)C(=O)NC(=S)Nc2ccc(cc2)Cc3c([nH]nc3C)C
907	CCCCOc1ccc(cc1C(=O)NC(=S)NNC(=O)COc2ccccc2C)Br
908	CCCNC(=O)c1ccccc1NC(=S)NC(=O)c2cc(ccc2OCCOC)Br
909	CCCCC(=O)Nc1ccc(cc1)C(=O)NNC(=O)COc2ccc(cc2Br)[C@H](C)CC
910	CCOc1cc(cc(c1OCC)OCC)C(=O)NC(=S)Nc2ccc(cc2)NC(=O)c3ccco3
911	CCCCC(=O)Nc1ccc(cc1)C(=O)Nc2nnc(s2)CCNS(=O)(=O)c3ccc(cc3)C
	CCCCn1c(c(c(=O)[nH]c1=O)N(CCOC)C(=O)COC(=O)c2cc(cnc2)Br)N

912<br>913<br>914<br>915<br>916<br>917<br>918<br>919<br>920<br>921<br>922<br>923<br>924<br>925<br>926<br>927<br>928<br>929<br>930<br>931<br>932<br>933<br>934<br>935<br>936<br>937<br>938<br>939<br>940<br>941<br>942<br>943<br>944<br>945<br>946<br>947<br>948	CCCN(CCC)Cc1c(nnn1c2c(non2)N)C(=O)N/N=C/c3ccc(cc3[O-])N(CC)CC<br>c1cc(ccc1COc2ccc(o2)/C=N/Nc3[nH]nc(n3)SCC(=O)Nc4ccc(cc4)[N+](=O)[O-])F<br>C=CCn1c(nnc1SCC(=O)Nc2nnc(s2)SCC(=O)N)Cn3c4ccccc4nn3<br>COC(=O)c1ccc(cc1)/C=N/Nc2[nH]nc(n2)SCCC(=O)Nc3ccc(cc3)C(F)(F)F<br>CCCCSc1ccccc1NC(=O)CSc2nc([nH]n2)N/N=C/c3c4ccccc4ccc3O<br>CC[C@H](C)Sc1nnc(s1)/N=C(/CSc2nnc(n2N)N/N=C/c3c(nn(c3C)C(C)C)C)\[O-]<br>CCCCSc1nnc(s1)/N=C(/CSc2nnc(n2N)N/N=C/c3cnn(c3)C(C)C)\[O-]<br>CN(C)c1ccc(cc1)/C=N/Nc2[nH]nc(n2)SCC(=O)Nc3nnc(s3)SCCOC<br>CCCCOc1cccc(c1)NC(=O)CSc2nc([nH]n2)N/N=C/c3cccc(c3Cl)Cl<br>CC[C@H](C)Sc1nnc(s1)/N=C(/CSc2nc([nH]n2)N/N=C/c3cccc(c3O)OC)\[O-]<br>CCNS(=O)(=O)c1ccc(cc1)NC(=O)CSc2nc([nH]n2)N/N=C/c3cc(ccc3[O-])OC<br>CC(C)OC(=O)c1c2c(sc1NC(=O)CSc3nnc(n3CCC(=O)N)C4CC4)CCCC2<br>CCCc1ccc(cc1)c2csc(n2)NC(=O)CSc3nnc(n3CCC(=O)N)c4ccco4<br>CCc1ccccc1Nc2nc(nc(n2)N)CSc3nnc(n3CC=C)COc4ccccc4F<br>CCCN(CCC)S(=O)(=O)c1ccc2c(c1)c(c[nH]2)/C=N\NC(=S)Nc3cccc(c3C)Cl<br>Cc1cc(ccc1C(=O)C2=C(C(=O)N([C@H]2c3ccc(cc3)OCC(=O)N)CC[NH+](C)C)[O-])OCC=C<br>CC(=O)c1ccc(cc1)NC(=S)N[C@@H](C(Cl)(Cl)Cl)NC(=O)COc2ccc(cc2)OC<br>CCCCOc1ccccc1C(=O)NC(=S)NNC(=O)c2ccc(cc2)NC(=O)c3ccccc3<br>Cc1ccc2c(c1)occ2CC(=O)OCC(=O)N(CCOC)c3c(n(c(=O)[nH]c3=O)Cc4ccccc4)N<br>C[C@]12C[C@H]([C@@H]3[C@H]([C@@H]1CC[C@@]2(C(=O)COC(=O)CCC(=O)N[C@@H](CC(=O)O)C(=O)[O-])O)CCC4=CC(=O)C=C[C@]34C)O<br>CCOc1ccc(cc1)NC[C@H](COc2ccc(cc2)OC[C@H](CNc3ccc(cc3)OCC)O)O<br>Cc1cc(no1)[N-]S(=O)(=O)c2ccc(cc2)NC(=O)CSc3nnc(n3CC=C)Cc4csc(n4)N<br>CCOc1cc(ccc1O)/C=N/Nc2[nH]nc(n2)SCC(=O)NCc3cccc(c3)C(F)(F)F<br>CC(C)(CNC(=S)Nc1ccc(cc1OC)OC)CNC(=S)Nc2ccc(cc2OC)OC<br>Cc1c(c(sc1C(=O)N)NC(=O)CSc2nnc(n2CC=C)c3cccc(c3)N(C)C)C(=O)OC<br>c1cc(ccc1NC(=S)Nc2cc(cc(c2)OCC(F)(F)F)OCC(F)(F)F)S(=O)(=O)N<br>CCc1cc(c(s1)NC(=S)Nc2ccc(cc2)S(=O)(=O)Nc3cc(ncn3)OC)C(=O)OC<br>COc1ccc(c(c1)/C=N/Nc2[nH]nc(n2)SCC(=O)Nc3nnc(s3)SCC=C)OC<br>CCCCC/C=C\C/C=C\C=C\C=C\[C@@H]([C@H](CCCC(=O)[O-])O)SC[C@@H](C(=O)NCC(=O)[O-])[NH3+]<br>c1cc(ccc1c2ccc([n+](n2)CCCC(=O)[O-])N)NC(=S)Nc3ccc(c(c3)C(=O)[O-])c4c5ccc(cc5oc-6cc(=O)ccc46)O<br>CCN(CC)c1ccc(cc1)CNC(=O)/C=C(\C)/NNC(=O)COc2ccc(cc2C)Br<br>c1cc-2c(cc1S(=O)(=O)N(CCO)CCO)C(=C(C#N)C#N)c3c2ccc(c3)S(=O)(=O)N(CCO)CCO<br>CC(C)Oc1ccc(cc1)[C@]2(C(=O)N(C(=O)N2)CCCCCCN3C(=O)[C@](NC3=O)(C)c4ccc(cc4)OC(C)C)C<br>CC1=NC(=C)C([C@@H](C1C(=O)OCC[NH+](C)C)c2ccccc2[N+](=O)[O-])C(=O)OCCNC(=O)c3ccccc3O<br>CCOC(=O)Oc1c(cc(cc1OC)C(=O)O[C@H]2C[C@@H]3C[NH+]4CCc5c6ccc(cc6[nH]c5[C@@H]4C[C@@H]3[C@@H]([C@@H]2OC)C(=O)OC)OC)OC<br>CC1(C(=O)N(C(=O)N1CN2C(=O)N(C(=O)C2(C)C)C[C@@H](CNc3ccccc3)O)C[C@@H](CNc4ccccc4)O)C<br>CC[NH+](CC)CCOC(=O)c1ccc(cc1)NC(=O)CCCN2C(=O)/C(=C/c3ccc(cc3)OC)/SC2=S<br>CC(=O)C(=C1NC(=C(C(=O)C)C(=O)OC(C)(C)C)NC(=C(C(=O)C)C(=O)OC(C)(C)C)N1)C(=O)OC(C)(C)C

949	Cc1cc(n[nH]1)NC(=O)CSc2nc3ccsc3c(=O)n2CCCC(=O)NCCc4ccc(cc4)S(=O)(=O)N
950	CCCC[NH+](C)CCCN1[C@H]([C@@]23C=C[C@@H](O2)[C@@H]([C@@H]3C1=O)C(=O)Nc4cccc(c4)OC)C(=O)N[C@@H]5CCCC[C@@H]5C
951	COCCNC(=O)c1ccc2c(c1)nc(n(c2=O)Cc3ccco3)SCC(=O)Nc4ccc(cc4OC)OC
952	c1cc[nH+]c(c1)N2CC[NH+](CC2)C[C@H](COc3ccc(cc3)OC[C@@H](CN4CCN(CC4)c5cccc[nH+]5)O)O
953	Cc1ccc2cc(c(=O)[nH]c2c1C)C[NH+](CCc3ccc(c(c3)OC)OC)Cc4nnnn4CCc5ccccc5
954	Cc1nc(c(cn1)CN(/C(=C(/SS/C(=C(/N(C=O)Cc2c(nc(nc2)C)N)\C)/CCO)\CCO)/C)C=O)N
955	CCOc1ccccc1N(CC(=O)Nc2ccccc2C(=O)NCCOC)S(=O)(=O)c3ccc(cc3)SC
956	CCCN(CCC)S(=O)(=O)c1ccc(cc1)C(=O)Nc2ccc(cc2)S(=O)(=O)Nc3cc(ncn3)OC
957	CCOC(=O)C1=C(NC(=C([C@H]1c2ccccc2Cl)C(=O)OC)C)COCCNC(=O)c3ccccc3C(=O)[O-]
958	c1ccc(c(c1)C(=O)N[C@@H]2C=C(C[C@H]([C@@H]2OC(=O)NCCCl)OC(=O)NCCCl)C(=O)NCC(=O)N)Br
959	CC[C@H](C)/C=C(\C)/C=C/C1=CC2=C(C(=O)[C@@](C(=O)C2=CN1[C@@H](CCCNC(=[NH2+])N)C(=O)[O-])(C)OC(=O)C)Cl
960	CCO[C@@H](C)SCC(=O)NC[C@H](CCC(=O)Oc1c(c(cc(c1F)F)F)F)NC(=O)CS[C@H](C)OCC
961	C/C=C(\C[C@H](N(Cc1cnc(nc1N)C)C=O)O)/SS/C(=C\C)/C[C@H](N(Cc2cnc(nc2N)C)C=O)O
962	C/C=C(/C[C@@H](N(Cc1c[nH+]c(nc1N)C)C=O)O)\SS/C(=C/C)/C[C@@H](N(Cc2cnc(nc2N)C)C=O)O
963	CC[NH+](CC)c1ccc2c(c1)oc-3cc(=[N+](CC)CC)ccc3c2c4ccc(cc4S(=O)(=O)[O-])S(=O)(=O)NCCSS(=O)(=O)C
964	Cn1c2ccc(cc2nc1CNc3ccc(cc3)C(=[NH2+])N)C(=O)N(CCC(=O)O[C@H]4[C@@H]([C@H]([C@@H]([C@H](O4)C(=O)[O-])O)O)O)c5ccccn5
965	CCCc1c2c(c(=O)[nH]c(n2)c3cc(ccc3OCC)S(=O)(=O)c4ccc(c(c4)c5[nH]c(=O)c6c(n5)c(nn6C)CCC)OCC)n(n1)C
966	CC(=O)O[C@@H]1[C@H]([C@@H](O[C@@H]([C@H]1OC(=O)C)Oc2ccc3c(c2)CCc4ccccc4N3CCC[NH2+]C)C(=O)OC)OC(=O)C
967	CC(=O)OC[C@@H]1[C@H]([C@@H]([C@@H]([C@H](O1)O[C@H]2[C@@H]([C@H](O[C@@H]([C@H]2OCC=C)OC)CO)O)OC(=O)C)OC(=O)C)OC(=O)C
968	CCC1=C(C(=O)c2c(c(c(c(c2O)Cl)Cl)O)C1=O)O[C@H]3[C@@H]([C@H]([C@@H]([C@H](O3)COC(=O)C)OC(=O)C)OC(=O)C)OC(=O)C
969	CCCCCCCCCC/[NH+]=C/[C@]12CC[C@@H](C[C@]1(CC[C@@H]3[C@@H]2CC[C@]4([C@@]3(CC[C@@H]4C5=CC(=O)OC5)O)C)O)O[C@@H]6C[C@@
970	H]([C@@H]([C@@H](O6)C)O)O
971	C[C@H]1C[C@]([C@@H](C[NH+]1C)C)(CCNC(=O)CCC(=O)OCC(=O)[C@]2(CC[C@@H]3[C@@]2(C[C@@H]([C@H]4[C@H]3CCC5=CC(=O)CC[C@]45C)
972	O)C)O)c6ccc(cc6)OC
973	Cc1c(c(c2c(c1OC(=O)C)C3=C(OCOC3=C(C2=O)C)/C(=C/C(=O)C)/C)O)NC(=O)/C(=C/C=C\[C@H](C)[C@H]([C@@H](C)[C@@H]4[C@@H]([C@@H]([C@@
974	H]([C@@H](O4)O)C)O)C(=O)OC)O)/C
975	Cc1c(c(sc1C(=O)OC)NC(=O)CCCC(=O)Nc2c(c(c(s2)C(=O)OC)C)C(=O)OC)C(=O)OC
976	CC[C@H](C)c1ccc(cc1)NC(=S)NNC(=O)CCCCC(=O)NNC(=S)Nc2ccc(cc2)[C@@H](C)CC
977	Cc1c(c(sc1C(=O)OC(C)C)NC(=O)CCSCCC(=O)Nc2c(c(c(s2)C(=O)OC(C)C)C)C#N)C#N
978	CCOc1cc(ccc1OCC=C)[C@@H]2C(=C(C(=O)N2CCCn3cc[nH+]c3)O)C(=O)c4ccc5c(c4)OCCO5
979	CCCN(CCC)S(=O)(=O)c1ccc(cc1)C(=O)Nc2ccc(cc2)S(=O)(=O)/N=c\3/cc(nc([nH]3)OC)OC
980	CC1(Cc2c(c(sc2C(N1)(C)C)NC(=O)c3ccc(cc3)S(=O)(=O)N(CCOC)CCOC)C(=O)OC)C
981	CC[NH+](CC)Cc1c(nnn1c2c(non2)N)C(=O)N/N=C/c3cc(c(c(c3)Br)OCc4ccccc4C)OC
982	CCN(CC)Cc1c(nnn1c2c(non2)N)C(=O)N/N=C/c3ccc(c(c3)OC)OC(=O)c4ccc(cc4)Cl
983	CC(C)CCOc1ccc(cc1OC)[C@H]2C(=C(C(=O)N2CCCn3cc[nH+]c3)O)C(=O)c4ccc5c(c4)OCCO5
984	COc1ccc2c(c1)/C(=N\NC(=O)CCCCCCCCC(=O)N/N=C\3/c4cc(ccc4NC3=O)OC)/C(=O)N2
985	CCN(CC)S(=O)(=O)c1ccc(cc1)C(=O)NCc2nnc(n2c3cc(ccc3OC)OC)SCC(=O)Nc4ccc(cc4)OC
	c1cc[nH+]c(c1)N2CCN(CC2)C[C@@H](COc3ccc(cc3)OC[C@@H](CN4CCN(CC4)c5ccccn5)O)O

986	c1cnc(nc1)N2CC[NH+](CC2)C[C@H](COc3ccc(cc3)OC[C@@H](CN4CCN(CC4)c5ncccn5)O)O
987	CCOc1ccc(cc1)n2c(nnc2SCC(=O)Nc3nccs3)CNC(=O)c4cc(c(c(c4)OC)OC)OC
988	CCCCCCCCCCCCCCCCN(C)S(=O)(=O)c1cccc(c1)S(=O)(=O)NNc2[n+](c3cc(ccc3s2)S(=O)(=O)[O-])C
989	C[C@H](COc1ccc(cc1)C(C)(C)c2ccc(cc2)OC[C@H](C)OC[C@@H](COC(=O)C=C)O)OC[C@@H](COC(=O)C=C)O
990	CC(=O)OC[C@H]([C@]1(CC[C@H]2[C@@]1(CC(=O)[C@@H]3[C@@H]2CC[C@@H]4[C@@]3(CC[C@H](C4)O[C@H]5[C@@H]([C@H]([C@@H]([C@@H]
991	(O5)C(=O)OC)OC(=O)C)OC(=O)C)OC(=O)C)C)C)O)O
992	CCOC(=O)CNC(=O)CCCCSc1c2c([n-]cn2)nc(n1)SCCCCC(=O)NCC(=O)OCC
993	Cc1c(scn1)/C=C\C2=C(N3[C@H]([C@H](C3=O)NC(=O)/C(=N/OC)/c4csc(n4)N)SC2)C(=O)OCOC(=O)C(C)(C)C
994	Cc1c(cccc1NC(=O)CCC(=O)N/N=C/c2ccc(cc2)/C=N/NC(=O)CCC(=O)Nc3c(c(ccc3)Cl)C)Cl
995	CCOCCCN([C@H](c1ccc(c(c1)OC)OC)C(=O)NC(C)(C)C)C(=O)c2c(c(ns2)C(=O)N)N
996	CCOC(=O)c1c(c(sc1NC(=O)CSc2nnc(s2)NC(=O)CC(C)C)C(=O)OCC)C
997	CC[NH+](CC)CCOC(=O)c1ccc(cc1)NC(=O)CCN2C(=O)/C(=C\c3ccc(cc3)OC)/SC2=O
998	CCOc1cc(cc(c1OCC)OCC)C(=O)Nc2nnc(s2)SCC(=O)Nc3cccc(c3)C
999	CCOCCOc1ccc(cc1)C(=O)NC(=S)NNC(=O)COc2ccc(cc2Br)C
1000	CCCCNC(=O)c1ccccc1NC(=O)c2ccc(cc2)C(=O)Nc3ccccc3C(=O)NCCCC
1001	CC(=O)NCCOc1ccc(cc1OCCNC(=O)C)NS(=O)(=O)c2ccc(cc2)N3CCCC3=O
1002	CCC(C)(C)NC(=O)[C@@H](c1ccc(cc1)OC)N(CCCOCC)C(=O)CCC(=O)Nc2cc(on2)C
1003	CC[C@H](c1nnnn1CC(=O)OCC)[NH+](Cc2ccc(cc2)OC)Cc3cc4ccc(c(c4[nH]c3=O)C)C
1004	CC[C@@H](c1nnnn1CC(=O)OCC)[NH+](Cc2ccccc2OC)Cc3cc4c(ccc(c4[nH]c3=O)C)C
1005	COC(=O)Cn1c(=O)c(c(n(c1=O)Cc2ccccc2)N)C(=O)COC(=O)CCC(=O)c3cccs3
1006	CC[NH+](CC)CCN1[C@@H]([C@@H](C(=O)C1=O)C(=O)c2c(c([nH]c2C)C(=O)OC)C)c3ccc(cc3)C(=O)OC
1007	CCOCCOc1cccc(c1)C(=O)NC(=S)Nc2ccc(cc2)S(=O)(=O)N(C)c3ccccc3
1008	CCN(CC)S(=O)(=O)c1ccc2c(c1)nc(n2C)CCC(=O)OCC(=O)NC(=O)NCCC(C)C
1009	CCCOC(=O)c1ccc(cc1)OCC(=O)NNC(=O)c2ccc(c(c2)Br)OCC(C)C
1010	CCCOC(=O)C[C@H]1C(=O)NCCN1C(=S)NC(=O)c2cc(ccc2OCCOC)Br
1011	CC[NH+](CC)CCCN1[C@@H](/C(=C(/c2ccc(cc2)OC(C)C)\O)/C(=O)C1=O)c3ccc(c(c3)OC)OCC
1012	COc1ccc(cc1)COc2ccc(o2)/C=N/Nc3[nH]nc(n3)SCC(=O)Nc4cccc(c4)OC
1013	COc1cc(cc(c1)OC)NC(=O)CSc2nnc(n2N)N/N=C/c3cc(c(c(c3)OC)OC)OC
1014	Cc1c(c(n(n1)C(C)C)C)/C=N/Nc2[nH]nc(n2)SCC(=O)Nc3nnc(s3)SCCOC
1015	COc1cc(cc(c1)OC)NC(=O)CCSc2nc([nH]n2)N/N=C/c3cc(c(c(c3)OC)OC)OC
1016	Cc1c(c(n(n1)C(C)C)C)/C=N/Nc2nnc(n2N)SCC(=O)Nc3nnc(s3)SCCOC
1017	COc1cc(cc(c1OC)OC)NC(=O)CSc2nnc(n2N)N/N=C/c3ccc(cc3)C(=O)OC
1018	CC[C@H](C)Sc1nnc(s1)NC(=O)CSc2nc([nH]n2)N/N=C(\C)/c3ccc(c(c3)OC)OC
1019	Cc1cc(nc(n1)N/C(=N/C(=S)Nc2cc(c(c(c2)OC)OC)OC)/NCCc3ccc(cc3)F)C
1020	C1CS(=O)(=O)C[C@@H]1NCCOC(=O)NCCCCCCNC(=O)OCCN[C@H]2CCS(=O)(=O)C2
1021	CC[NH+](CC)CCN1[C@H](C(=C(C1=O)O)C(=O)c2cnn(c2C)c3ccccc3)c4cccc(c4)OCC=C
1022	CCCOc1ccc(cc1OCC)[C@H]2C(=C(C(=O)N2CCC[NH+](C)C)O)C(=O)c3c(nc4n3cccc4)C
	CCCCCCOc1ccc(cc1)[C@@H]2C(=C(C(=O)N2CCC[NH+](C)C)O)C(=O)c3c(nc4n3cccc4)C

1023	CCOc1ccc(cc1)C(=O)C2=C(C(=O)N([C@@H]2c3ccc(c(c3)OCC)OCC=C)Cc4cc[nH+]cc4)O
1024	CC/C=C\C/C=C\C/C=C\CCCCCCCC(=O)OC[C@H](CO[C@H]1[C@@H]([C@@H]([C@@H]([C@H](O1)CO)O)O)O)O
1025	CCCOc1ccc(cc1OCC)[C@@H]2C(=C(C(=O)N2CCCn3cc[nH+]c3)O)C(=O)c4c(nc(s4)C)C
1026	CC[C@H](C)NC(=O)c1csc(n1)C[NH+](CCc2ccc(c(c2)OC)OC)Cc3ccc4c(c3)OCO4
1027	Cc1c(c(n(n1)c2ccccc2)Oc3ccc(cc3)OC)C[NH+](Cc4ccco4)C[C@@H](COC(C)(C)C)O
1028	CC(C)OC[C@@H](C[NH+](Cc1c(nn(c1Oc2cccc(c2)OC)C)c3ccccc3)C[C@@H]4CCCO4)O
1029	Cc1ccc(cc1)C(=O)CCC(=O)OCC(=O)N(CCOC)c2c(n(c(=O)[nH]c2=O)Cc3ccccc3)N
1030	CCOc1cc(cc(c1OCC)OCC)C(=O)NCCn2c3c(cn2)c(ncn3)NCc4ccc(cc4)F
1031	C[NH+](C)CCCN1[C@H](C(=C(C1=O)O)C(=O)c2ccc(cc2)OCC=C)c3cc(c(c(c3)OC)OC)OC
1032	CC(=O)N[C@@H]1[C@@H]([C@@H]([C@@H](O[C@@H]1OC(=O)CNC(=O)c2ccccc2)COC(=O)C)OC(=O)C)OC(=O)C
1033	Cc1ccc(c(c1)C)OCCCn2c3ccccc3[nH+]c2CCNC(=O)c4cc(c(c(c4)OC)OC)OC
1034	CC(C)c1ccccc1OCCn2c3ccccc3[nH+]c2CCNC(=O)c4cc(c(c(c4)OC)OC)OC
1035	COc1cc(cc(c1OC)OC)C(=O)NCCCc2[nH+]c3ccccc3n2CCOc4ccccc4Cl
1036	Cc1cc(cc(c1)OCCn2c3ccccc3[nH+]c2CCCNC(=O)c4cc(c(c(c4)OC)OC)OC)C
1037	COc1cc(cc(c1OC)OC)C(=O)NCc2[nH+]c3ccccc3n2CCCCOc4ccccc4Cl
1038	CCOC(=O)c1cccc(c1)NC(=O)CSc2nnc(n2CC=C)CCNC(=O)c3ccc(cc3)C
1039	CCN(CC)S(=O)(=O)c1ccc(cc1)NC(=O)CSc2nnc(n2CC=C)CNc3ccccc3
1040	CC[C@@H](C)NS(=O)(=O)c1ccc(cc1)NC(=O)CSc2nnc(n2CC=C)CNc3ccccc3
1041	COc1cc2c(cc1OC)[nH]c(=O)n(c2=O)CCCCC(=O)NCCOc3cccc(c3)C(F)(F)F
1042	COCCOc1ccccc1C(=O)NC(=S)Nc2ccc(cc2)S(=O)(=O)Nc3cc(ncn3)OC
1043	CCC[NH+](Cc1nc(c2c(c(sc2n1)C(=O)OC(C)C)C)[O-])C[C@@H](COc3ccc(cc3)Cl)O
1044	CCOc1ccc(cc1)[C@@H](C(=O)NC(C)(C)C)N(Cc2ccc(cc2)OC)C(=O)CCn3c(cc(n3)C)C
1045	CCCCOc1ccc(cc1)C(=O)Nc2ccc(cc2)c3nnc(n3C)SCCOc4ccc(cc4)C
1046	CC[C@H](C)c1ccc(cc1)OCC(=O)N/N=C/c2ccc(cc2)OC(=O)c3cc(c(c(c3)OC)OC)OC
1047	CC(C)COP(=O)([C@](C(=O)OC)(C(F)(F)F)Nc1nc2ccc(cc2s1)Cl)OCC(C)C
1048	CCOc1ccc(cc1)/C(=C/2\[C@@H](N(C(=O)C2=O)CCCn3ccnc3)c4ccc(c(c4)OCC)OCC)/O
1049	CC[NH+](CC)CCCN1[C@H](C(=C(C1=O)[O-])C(=O)c2ccc(cc2)OCC)c3ccc(c(c3)OCC)OCC
1050	CC[NH+](CC)CCCN1[C@H](C(=C(C1=O)[O-])C(=O)c2ccc3c(c2)C[C@H](O3)C)c4ccc(c(c4)OCC)OCC
1051	CCCCSc1nnc(s1)/N=C(/CSc2nnc(n2N)N/N=C/c3c(nn(c3C)C(C)(C)C)\[O-]
1052	CC[C@@H](c1ccc(cc1)OC)NC(=O)CN(c2ccc(cc2)OCC)S(=O)(=O)c3ccc(cc3)SC
1053	CCCC[NH+](CCCC)CCCN1[C@H](C(=C(C1=O)[O-])C(=O)c2ccc(cc2)C)c3ccc(cc3)C(=O)OC
1054	CC1=C(C(C(=C(N1)C)C(=O)OCC=C)c2ccc(cc2)OC(=O)COc3ccccc3F)C(=O)OCC=C
1055	CCCCSCCCNC(=O)c1c[nH]c2ccc(cc2c1=O)S(=O)(=O)[N-]c3cccc(c3)C(F)(F)F
1056	CC[NH+](CC)CCCN1[C@H](C(=C(C1=O)[O-])C(=O)c2cc3cc(ccc3o2)C)c4ccc(c(c4)OC)OCC
1057	CCOc1ccc(cc1OCC)[C@@H]2C(=C(C(=O)N2CCC[NH+](C)C)[O-])C(=O)c3cc4cccc(c4o3)OC
1058	CCCOc1ccc(cc1)C(=O)C2=C(C(=O)N([C@@H]2c3ccc(c(c3)OCC)OCC)CC[NH+](CC)CC)[O-]
1059	CC[NH+](CC)CCCN1[C@H](C(=C(C1=O)[O-])C(=O)c2cccc(c2)OCC)c3cccc(c3)Oc4ccccc4
	CCOc1cc(ccc1OCC=C)[C@@H]2C(=C(C(=O)N2CCCn3cc[nH+]c3)[O-])C(=O)c4ccc(cc4)OC

1060	CC[NH+](CC)CCN1[C@H](C(=C(C1=O)[O-])C(=O)c2ccc(cc2C)OCC=C)c3ccc(c(c3)OC)OCC=C
1061	CC[NH+](CC)CCCN1[C@H](C(=C(C1=O)[O-])C(=O)c2c(nc3n2cccc3)C)c4ccc(c(c4)OC)OCC
1062	CCCOc1ccc(cc1)/C=N\Nc2nc3c(n2C[C@H](COc4ccc(cc4)CC)O)c(nc(=O)n3C)[O-]
1063	Cc1c(c(n(n1)c2ccccc2)Oc3ccc(cc3)OC)CN(Cc4ccco4)C[C@H](COCC(C)C)O
1064	CCCCOC[C@H](CN(Cc1ccco1)Cc2c(nn(c2Oc3cccc(c3)OC)C)c4ccccc4)O
1065	Cc1ccccc1OC[C@@H](CN(Cc2c(nn(c2Oc3cccc(c3)OC)C)c4ccccc4)CC(C)C)O
1066	CCCCOC[C@H](CN1CCN(CC1)c2c(c(nc(n2)c3ccccc3)C)Cc4ccc(cc4)[N+](=O)[O-])O
1067	CCC(CC)[C@@H](C(=O)NCS(=O)(=O)c1ccc(cc1)C)N(Cc2ccco2)C(=O)Cc3ccccc3C
1068	CCCOc1cccc(c1)[C@@H]2C(=C(C(=O)N2CC[NH+](CC)CC)[O-])C(=O)c3cnn(c3C)c4ccccc4
1069	CC[NH+](CC)CCCN1[C@H](C(=C(C1=O)[O-])C(=O)c2cnn(c2C)c3ccccc3)c4cccc(c4)OCC
1070	CCCCN(CCCC)S(=O)(=O)c1ccc(cc1)C(=O)Nc2nnc(o2)c3cccc(c3)SC(C)C
1071	Cc1ccc(c(c1)OCCCn2c3ccccc3nc2[C@H](C)NC(=O)Cc4ccc(c(c4)OC)OC)C(C)C
1072	CCCCCCOc1ccc(cc1OC)[C@@H]2c3c(n[nH]c3C(=O)N2CCCO)c4cc(c(cc4[O-])C)Cl
1073	CCCOc1ccc(cc1OCC)[C@H]2c3c([nH]nc3C(=O)N2CCCOC(C)C)c4cc(cc(c4[O-])C)C
1074	CCCCOc1ccc(cc1OC)[C@H]2c3c([nH]nc3C(=O)N2CCCOC(C)C)c4c(cc(cc4[O-])C)C
1075	CCCCCCOc1ccc(cc1OC)[C@H]2c3c([nH]nc3C(=O)N2CCCOC)c4c(cc(cc4[O-])C)C
1076	CC(C)(C)c1ccc(cc1)OCCCc2nnc(n2CC=C)SCC(=O)Nc3cccc(c3)C(=O)OC
1077	CCOc1cc(ccc1OCc2ccccc2C)[C@@H]3C(=C(Nc4n3nc(n4)SCC)C)C(=O)OCC=C
1078	CC[C@@H](c1nnnn1CCc2ccccc2)N(Cc3cccs3)Cc4cc5cc(c(cc5[nH]c4=O)OC)OC
1079	CCCCCOc1ccc(cc1)[C@H]2C(=C(Nc3n2nc(n3)SCc4ccccc4Cl)C)C(=O)OCC
1080	CCCCOc1ccccc1[C@H]2C(=C(Nc3n2nc(n3)SCc4ccccc4Cl)C)C(=O)OCC=C
1081	[H]/N=C\1/C(=C/c2ccc(c(c2)OC)OCCOCCOc3ccc(cc3)C)/C(=O)N=C4N1N=C(S4)CCC
1082	Cc1cc(ccc1OCCCc2nnc(n2CC=C)SCC(=O)Nc3cc(ccc3OC)OC)Cl
1083	C=CCn1c(nnc1SCC(=O)Nc2ncc(s2)Cc3cccc(c3)C(F)(F)F)COc4ccccc4
1084	CC(=O)Oc1c(cc2c(c1O)c(=O)cc(o2)c3ccccc3)O[C@@H]4[C@H]([C@@H]([C@H]([C@@H](O4)C(=O)[O-])OC(=O)C)OC(=O)C)OC(=O)C
1085	CCCCCCOc1ccc(cc1OC)/C=C(\C#N)/C(=O)Nc2nc(cs2)c3ccc(cc3)OC(C)C
1086	CC[C@H](C)N(Cc1ccc(cc1OS(=O)(=O)c2ccc(c(c2)Cl)Cl)[NH+](CC)CC)C(=O)COC
1087	CC[NH+](CC)c1ccc(c(c1)OS(=O)(=O)c2cccc(c2)C(F)(F)F)CN(CC(C)C)C(=O)COC
1088	CCCCCOP(=O)([C@@](C(=O)OCC)(C(F)(F)F)NS(=O)(=O)c1ccc(cc1)C)OCCCCC
1089	CCCCOc1ccc(cc1C)C(=O)C2=C(C(=O)N([C@@H]2c3cccc(c3)Oc4ccccc4)CCC[NH+](C)C)[O-]
1090	CC[NH+](CC)CCN1[C@H](C(=C(C1=O)[O-])C(=O)c2ccc(c(c2)C)OCC(C)C)c3cccc(c3)Oc4ccccc4
1091	CCO/C(=C(\C=C)/OC)/C=C\CCN1c2ccccc2/C(=N\NC(=O)COc3cccc4c3cccc4)/C1=O
1092	CCCCOc1ccc(cc1)c2cc(c(c(n2)SCC(=O)Nc3ccc(cc3)C(=O)OCC)C#N)C(F)(F)F
1093	CCc1nnc(s1)NS(=O)(=O)c2ccc(cc2)NC(=O)c3ccc(cc3)S(=O)(=O)N(CCC#N)CCC#N
1094	Cc1ccc2c(c1)n(c(=O)n2C[C@H]([C@@H]3[C@@H]([C@H]4[C@H](O3)OC(O4)(C)C)OCC=C)O)C[C@H]([C@@H]5[C@@H]([C@@H]6[C@H](O5)OC(O6)(C)C)OCC=C)O
1095	CC[NH+]1CCN(CC1)c2cccc3c2CN(C3=O)[C@H](CCCNS(=O)(=O)c4cccs4)c5ccc(c(c5)OC)OC
1096	CC(C)C(=O)Nc1[nH]c(=O)c2c(n1)n(cn2)[C@@H]3C[C@@H]([C@@H](O3)O[P@@](=O)([O-])Oc4ccc(cc4)Cl)OC(=O)CCC(=O)C

```
Cc1c2cc3[nH+]c(cc4c(c(c([nH]4)cc5[nH+]c(cc(c1CCC(=O)O)[nH]2)C(=C5CCC(=O)[O-])C)C)CCC(=O)[O-])C(=C3CCC(=O)[O-])C
c1cnn(c1)c2ccc(cc2)NC(=S)NC[C@H]3C[C@@H]4CCN3C[C@@H]4C[NH+]5CCN(CC5)C(=S)Nc6ccc(cc6)n7cccn7
C/C(=C\CC[C@](C)(C=C)O[C@H]1[C@@H]([C@H]([C@@H]([C@H](O1)CO)OC(=O)/C(=C/CC[C@](C)(C=C)O)/C)OC(=O)/C(=C/CC[C@@](C)(C=C)O)/C)O)/C(=O)[O-]
C/C(=C\CC[C@@](C)(C=C)O[C@H]1[C@@H]([C@H]([C@@H]([C@H](O1)COC(=O)/C(=C/CC[C@](C)(C=C)O)/C)O)OC(=O)/C(=C/CC[C@@](C)(C=C)O)/C)O)/C(=O)[O-]
C/C(=C\CC[C@@](C)(C=C)O[C@H]1[C@@H]([C@H]([C@@H]([C@H](O1)CO)OC(=O)/C(=C/CC[C@@](C)(C=C)O)/C)OC(=O)/C(=C/CC[C@@](C)(C=C)O)/C)O)/C(=O)O
C/C(=C\CC[C@@](C)(C=C)O[C@H]1[C@@H]([C@H]([C@@H]([C@H](O1)COC(=O)/C(=C/CC[C@@](C)(C=C)O)/C)O)OC(=O)/C(=C/CC[C@@](C)(C=C)O)/C)O)/C(=O)O
c1c(cc(cc1CO)OCc2cc(cc(c2)COc3cc(cc(c3)CO)CO)COc4cc(cc(c4)CO)CO)CO
CC(=O)O[C@H]1[C@@H]([C@@H](O[C@@H]([C@@H]1O)O[C@@H](/C=N\C23CC4CC(C2)CC(C4)C3)[C@@H](CO)O[C@@H](/C=N/C56CC7CC(C5)CC(C7)C6)O)CO)O
CCCO[C@@H]1[C@@H]2[C@H](O[C@@H]1[C@@H](CO[C@H]3[C@@H]([C@H]([C@@H]([C@H](O3)COC(=O)C)OC(=O)C)OC(=O)C)NC(=O)C)O)OC(O2)(C)C
C[C@]12CCC(=O)C=C1CC[C@@H]3[C@@H]2C(=O)C[C@]4([C@H]3CC[C@@]4(C(=O)COC(=O)CCC(=O)N[C@@H](CCSC)C(=O)[O-])O)C
c1cc(c(cc1CC[NH2+]CCC(=O)N(CCNCCc2ccc(c3c2OCC(=O)N3)O)C4CCCCC4)Cl)Cl
c1ccc(cc1)COc2c3c(n/c(=N/CNc4[nH+]c(c5c(n4)n(cn5)COCCO)OCc6ccccc6)/[nH]2)n(cn3)COCCO
COc1ccccc1OCC[NH2+]C[C@H](COc2cccc3c2c4ccccc4n3C[C@@H](COc5cccc6c5c7ccccc7[nH]6)O)O
c1ccc2c(c1)c3c([nH]2)cccc3OC[C@H](C[NH2+]CCOc4ccccc4OCC[NH2+]C[C@@H](COc5cccc6c5c7ccccc7[nH]6)O)O
CC(=O)OC[C@@H]1[C@H]([C@@H]([C@H]([C@@H](O1)Nc2ccc(cc2)Oc3ccnc(c3)C(=O)NC)OC(=O)C)OC(=O)C)OC(=O)C
CCCCCCCCCC/N=C/[C@]12CC[C@@H](C[C@]1(CC[C@@H]3[C@@H]2CC[C@]4([C@@]3(CC[C@@H]4C5=CC(=O)OC5)O)C)O)O[C@@H]6C[C@@H]([C@@H]([C@@H](O6)C)O)O
CCCCCCCCCCN=C[C@]12CC[C@@H](C[C@]1(CC[C@@H]3[C@@H]2CC[C@]4([C@@]3(CC[C@@H]4C5=CC(=O)OC5)O)C)O)O[C@H]6C[C@H]([C@@H]([C@@H](O6)C)O)O
C[C@@H]1C[C@]([C@@H](C[NH+]1C)C)(CCNC(=O)CCC(=O)OCC(=O)[C@]2(CC[C@@H]3[C@@]2(C[C@@H]([C@H]4[C@H]3CCC5=CC(=O)CC[C@]45C)O)C)O)c6ccc(cc6)OC
CC[C@H]([C@H]1CC[C@@H]([C@@H](O1)[C@@H](C)[C@@H]([C@H](C)C(=O)[C@H](CC)[C@@H]2[C@H](C[C@H]([C@]3(O2)CC[C@H]([C@@]4(O3)CC[C@@](O4)(C)[C@H]5CC[C@@]([C@@H](O5)C)(CC)O)O)C)C)O)C)C(=O)[O-]
C[C@]12CC[C@@H]3c4ccc(cc4C[C@H]([C@H]3[C@@H]1CC[C@@H]2O)CCCCCCCCC[S@@](=O)CCCC(C(F)(F)F)(F)F)O[C@H]5[C@@H]([C@H]([C@@H]([C@H](O5)C(=O)[O-])O)O)O
CC1=C2[C@H](C(=O)[C@]3([C@H]([C@@H]([C@@](C2(C)C)(C[C@@H]1OC(=O)[C@@H]([C@H](c4ccccc4)NC(=O)OC(C)(C)C)O)O)OC(=O)c5ccccc5)[C@@]6(CO[C@@H]6[C@H]7[C@@H]3O7)OC(=O)C)C)[O-]
CC1=C2[C@H](C(=O)[C@@]3([C@H](C[C@@H]4[C@]([C@@H]3[C@@H]([C@@](C2(C)C)(C[C@@H]1OC(=O)[C@@H]([C@H](c5ccccc5)/N=C(/O)\OC(C)(C)C)O)O)OC(=O)c6ccccc6)(CO4)OC(=O)C)O)C)O
CSCC[C@@H](C(=O)N[N-]S(=O)(=O)c1ccc(c(c1)Cl)Cl)NS(=O)(=O)c2cc(cc(c2)C(F)(F)F)C(F)(F)F
CCOC(=O)Cc1csc(n1)NC(=O)CSc2nnc(s2)SCC(=O)Nc3nc(cs3)CC(=O)OCC
```

1134	CC[C@@H](C(=O)NC/C=C/C=C(\C)/[C@@H]([C@@H](C)[C@@H]1[C@@H]([C@@H]([C@@H](O1)/C=C/C=C/C=C(\C)/C(=O)c2c(cc[nH]c2=O)[O-
1135	])O)O)OC)[C@@]3([C@@H]([C@@H](C([C@@H](O3)/C=C/C=C/C)(C)C)O)O)O
1136	Cc1cc(cc(c1)NS(=O)(=O)c2ccc(cc2)NC(=O)CCCC(=O)Nc3ccc(cc3)S(=O)(=O)[N-]c4cc(cc(c4)C)C)C
1137	CCN(CC)c1ccc(cc1)NC(=O)CSc2c(c(sn2)SCC(=O)Nc3ccc(cc3)N(CC)CC)C#N
1138	CCOc1cc(cc(c1OCC)OCC)C(=O)NCc2nnc(n2c3cccc(c3C)C)SCC(=O)Nc4nnc(s4)C
1139	CC(=O)CCC(=O)O[C@@H]1C[C@@H](O[C@@H]1O[P@@](=O)([O-])Oc2ccc(cc2)Cl)n3cnc4c3N=CN[C@@H]4NC(=O)c5ccccc5
1140	CC[NH+](CC)c1ccc(c(c1)OCc2c(cccc2Cl)F)/C=N/NC(=O)c3c(n(nn3)c4c(non4)N)C[NH+]5CCC(CC5)C
1141	CCSc1nnc(s1)NC(=O)CSc2nnc(n2c3ccc(cc3)Br)CNC(=O)c4ccc(cc4)S(=O)(=O)N5CCCC5
1142	c1ccc(cc1)c2nc(c(o2)COC(=O)C[C@H](c3ccccc3)NC(=O)c4ccccc4)COC(=O)C[C@@H](c5ccccc5)NC(=O)c6ccccc6
1143	CCCCSc1nnc(s1)NC(=O)CSc2nnc3n2nc(s3)SCC(=O)Nc4nnc(s4)SCCCC
1144	CCOc1ccc(cc1OCC)CCNS(=O)(=O)c2cc(ccc2C)c3c4ccccc4c(n[nH+]3)Nc5ccc(cc5)C(=O)N
1145	c1cc(ccc1c2ccc(cc2)NC(=O)CCCC(=O)OCC(C(F)F)(F)F)NC(=O)CCCC(=O)OCC(C(F)F)(F)F
1146	C=CCc1cccc(c1O)/C=N/NC(=O)CSc2nnc(n2CC=C)CNc3ccc(cc3)I
1147	COc1ccc(c(c1)n2c(nnc2SCC(=O)Nc3nccs3)CNC(=O)c4cc(cc(c4)OC)OC)OC
1148	CCc1nnc(s1)NC(=O)CCCCCN2C(=O)/C(=C\3/C(=O)N(C(=S)S3)CCCCCC(=O)Nc4nnc(s4)CC)/SC2=S
1149	CCCCCCCCCCCCCCCCS(=O)(=O)c1ccc(cc1C(=O)[O-])S(=O)(=O)NNc2[n+](c3ccccc3s2)C
1150	CCCOc1ccc(cc1)C(=O)Oc2ccc(cc2)/C=N/NC(=O)C(=O)Nc3ccccc3C(=O)Nc4ccc(cc4)C
1151	CC(=O)O[C@H]1[C@@H]([C@H](O[C@H]([C@@H]1OC(=O)C)O[C@@H]2CC[C@@]3([C@@H](C2)CC[C@@H]4[C@@H]3C(=O)C[C@@]5([C@@H]4CC[
1152	C@@H]5[C@@H](CO)O)C)C)C(=O)OC)OC(=O)C
1153	CC(=O)O[C@@H]1[C@H]([C@H](O[C@@H]([C@H]1OC(=O)C)OCC(=O)[C@]2(CC[C@H]3[C@@]2(C[C@@H]([C@@H]4[C@@H]3CCC5=CC(=O)C=C[C@
1154	@]45C)O)C)O)C(=O)OC)OC(=O)C
1155	Cc1c(sc(n1)n2c(cc(n2)c3ccccc3)c4ccccc4)C(=O)/C=C(\C(=O)Nc5ccccc5C(=O)C)/NNC(=O)c6ccccc6S(=O)(=O)N
1156	CC(C)CN(C[C@@H]([C@@H](c1ccccc1)c2c(c3ccccc3oc2=O)[O-])/C(=N/NC(=O)c4ccccc4S(=O)(=O)N)/C)CC(C)C
1157	c1ccn2c(c1)nc(c(c2=O)/C=C\3/C(=O)N(C(=S)S3)CCCCCCCCCCC(=O)[O-])NCCOCCO
1158	Cc1c(cccc1NC(=O)CCC(=O)N/N=C\c2ccc(cc2)/C=N\NC(=O)CCC(=O)Nc3c(c(ccc3)Cl)C)Cl
1159	CCCNc1c(c(=O)n2ccccc2n1)/C=C\3/C(=O)N(C(=S)S3)C[C@@H]4CCCO4
1160	Cc1cc(c(n1[C@H]2CCS(=O)(=O)C2)C)C(=O)CSc3[nH]c(nn3)c4cccs4
1161	Cc1c(cnn1c2ccccc2)C(=O)NCCN3C(=O)/C(=C/c4ccccc4)/SC3=O
1162	Cc1c(c2c(cn1)CN(CC2)C(=O)c3cccc(c3)N4CCCC4=O)CNC(=O)Cc5ccsc5
1163	Cc1c(c2c(cn1)CN(CC2)C(=O)c3csc(n3)SC)CNS(=O)(=O)c4ccsc4
1164	CN([C@H]1CCc2c1cccc2)C(=O)c3ccc(cc3)NC(=O)C[C@@H]4C(=O)N=C(S4)N5CCCC5
1165	c1cc(cc(c1)S(=O)(=O)N2CCCC2)NC(=O)c3ccc(cc3)S(=O)(=O)C(F)F
1166	COC(=O)C[C@H](c1ccc(cc1)Cl)NC(=O)c2ccc(cc2)CN3C(=O)CCC3=O
1167	Cc1nccn1CCC(=O)NCC2CCC(CC2)c3cc(c4ccccc4n3)C(=O)N(C)C
1168	Cc1ccc(nc1)c2ccc3c(c2)C[C@@H](O3)CNC(=O)CCN(C)[C@@H]4CCS(=O)(=O)C4
1169	Cc1c(cnc(n1)C)C(=O)C2=C(C(=O)N([C@@H]2c3cccc(c3)Cl)CCC[NH+](C)C)[O-]
1170	c1cc(c(cc1S(=O)(=O)NC2CC2)C(=O)N3CCN(CC3)CCN4CCCC4)Br
	c1cc(cc(c1)S(=O)(=O)N2CCCC2)NC(=O)CN3CCN(CC3)Cc4ccsc4

1171	Cc1c(sc2c1c(=O)n(cn2)CCC(=O)NCc3ccc(o3)c4csc(n4)C)C
1172	Cn1ccnc1[C@@](CC(=O)N2CCCN(CC2)c3ccc(cn3)C(F)(F)F)(C(F)(F)F)O
1173	c1ccc(cc1)S(=O)(=O)N2CCN(CC2)Cc3nc4ccc(cc4n3CCO)Cl
1174	c1ccc2c(c1)CCN2C(=O)CSc3ccccc3C(=O)NCc4nnc5n4CCC5
1175	CCN(CC)C1=NC(=O)[C@@H](S1)CC(=O)Nc2cccc(c2)C(=O)N(C)[C@@H](C)C3CC3
1176	CCN1CCN(CC1)CCCNC(=O)c2cc(ccc2Cl)S(=O)(=O)N3CCSCC3
1177	c1cc(ccc1/C=C/2\C(=O)N(C(=S)S2)CCCC(=O)N[C@H]3CS(=O)(=O)C=C3)F
1178	Cc1c(sc(n1)C)C(=O)C2=C(C(=O)N([C@H]2c3ccccc3OCC=C)CC[NH+](C)C)[O-]
1179	Cc1c(sc(n1)C)C(=O)C2=C(C(=O)N([C@H]2c3ccc(cc3)N(C)C)CCCOC)O
1180	Cc1c(sc(n1)C)C(=O)C2=C(C(=O)N([C@H]2c3cccs3)CCCn4cc[nH+]c4)[O-]
1181	c1cc(cc(c1)Cl)N2CCN(CC2)Cc3nc(cs3)C(=O)NCc4cccnc4
1182	CC[C@@H](C)/C=C/C1=CC2=C(C(=O)[C@]3([C@@H](C2=CO1)[C@@H](C(=O)O3)C(=O)[C@@H](C)[C@@H](C)O)C)Cl
1183	Cc1c(sc(n1)C)C(=O)C2=C(C(=O)N([C@H]2c3cccc(c3)OCC=C)CC[NH+](C)C)[O-]
1184	c1cc(sc1)C2=NN[C@@H](N2C3CC3)SCC(=O)C4=CC(=NC4)C(=O)N5CCCC5
1185	C[C@H](Cn1ccnc1)NC(=O)c2cccc(c2)S(=O)(=O)N3CCc4c(ccs4)C3
1186	C[C@]1(CC1(Cl)Cl)C(=O)NCCS(=O)(=O)N2CCN(CC2)C(=O)[C@@]3(CC3(Cl)Cl)C
1187	c1cc(cc(c1)Br)NC(=O)COc2coc(cc2=O)CN3CCSCC3
1188	CS(=O)(=O)c1ccc(cc1)NC(=O)CS(=O)(=O)Cc2cccc(c2)Br
1189	Cn1ccnc1[C@@H](c2ccccc2N3CCN(CC3)Cc4ccc(c(c4)OC)OCC#C)O
1190	c1ccc(c(c1)CN(CCS(=O)(=O)N2CCNCC2)C(=O)Cc3cccs3)Cl
1191	CN(C[C@H]1CC=C(S1)Cl)C(=O)CCc2[nH]c(=O)c3cnn(c3n2)c4ccccc4
1192	c1cc(sc1)CNS(=O)(=O)c2ccc3c(c2)CCN3C(=O)CCC(=O)N4CCCC4
1193	CCN1CCC[C@H]1CN(CC(=O)N2CCNCC2)S(=O)(=O)c3c(cc(cc3C)C)C
1194	Cc1cccc(c1NC(=O)CN(C)C(=O)c2cccnc2SCc3c(noc3C)C)C
1195	Cc1c(nnn1c2cccc3c2CCN(C3)Cc4cccnc4)C(=O)NCC(F)(F)F
1196	CCN(CC)c1ccc(cc1)NC(=O)CCCN2C(=O)c3ccccc3N4[C@@]2(CCC4=O)C
1197	Cc1nccn1C[C@@H](C)CNC(=O)CCn2cnc3ccc(cc3c2=O)Br
1198	CCN1CCN(CC1)[C@@H](C)CNc2c3c(c(sc3nc(n2)CN4CCOCC4)C)C
1199	COC(=O)C[C@@H](c1cccc(c1)Br)NC(=O)c2ccc(cc2)N3CCCCC3=O
1200	Cn1c(c2ccc(cc2nc1=S)C(=O)N(CC[NH+](C)C)Cc3ccc(cc3F)F)[O-]
1201	c1cc(ccc1C(=O)N2CSC[C@H]2C(=O)Nc3ccn(n3)CCc4ccncc4)Cl
1202	CCn1cc(c(=O)c2c1ccc(c2)S(=O)(=O)N(C)C)C(=O)NCCc3ccc(cc3)Cl
1203	CCn1cc(c(=O)c2c1ccc(c2)S(=O)(=O)N(C)C)C(=O)NCCc3ccc(cc3)C
1204	C[C@@H](C(=O)Nc1ccc(c(c1)Cl)Cl)Sc2c(on[n+]2c3ccc(cc3)OC)[O-]
1205	Cn1c(cc(=O)c(c1CN2CCc3ccccc3C2)O)CN4CCc5cc(c(cc5C4)OC)OC
1206	CCC(=O)NCc1nc2c(n1C)CN(CC2)S(=O)(=O)c3cccc(c3)C(F)(F)F
1207	Cc1cc(c(n1c2ccc(cc2)C#N)C)C(=O)N(CCO)Cc3cc(ccc3OC)OC
	CCCCCCCCCCCCCC(=O)O[C@@H]1[C@H]([C@]2([C@@H](C=C(C[C@]3([C@H]2C=C(C3=O)C)O)CO)[C@H]4[C@@]1(C4(C)C)OC(=O)C)O)C

1208	CCOc1cc(cc(c1OCC)OCC)C(=O)Nc2ccc(cc2NC(=O)c3cc(c(c(c3)OCC)OCC)OCC)C
1209	Cc1ccc(cc1)S(=O)(=O)n2ccc3c2cccc3OCCOCCSC[C@H](C(=O)[O-])NC(=O)OC(C)(C)C
1210	CCCCCCCCCCCCCC(=O)O[C@@H]1[C@H]([C@]2([C@@H](C=C(C[C@]3([C@H]2C=C(C3=O)C)OC)CO)[C@H]4[C@@]1(C4(C)C)OC(=O)C)O)C
1211	CCc1c(cc(c(c1CC(=O)N(CCOC)CCOC)C(=O)c2ccc(c(c2)OC)OCCN3CCOCC3)[O-])O
1212	CC[C@H](C)[C@H](C(=O)[O-])NC(=O)CCC(=O)OCC(=O)[C@]1(CC[C@@H]2[C@@]1(CC[C@H]3[C@H]2CCC4=CC(=O)CC[C@]34C)C)OC(=O)C
1213	CCCCCC(=O)OCC(=O)[C@]1([C@@H](C[C@@H]2[C@@]1(C[C@@H]([C@]3([C@H]2CCC4=CC(=O)C=C[C@@]43C)F)OC(=O)CCCCC)C)C)O
1214	CCc1ccc([nH+]c1)CCOc2ccc(cc2)C[C@H](C(=O)[O-])SS[C@H](Cc3ccc(cc3)OCCc4ccc(cn4)CC)C(=O)[O-]
1215	CCc1ccc([nH+]c1)CCOc2ccc(cc2)C[C@H](C(=O)OCC)SS[C@H](Cc3ccc(cc3)OCCc4ccc(cn4)CC)C(=O)OCC
1216	CCC/C(=N\[C@@H](C)CN/C(=C\1/C(=O)CC([C@H](C1=O)C(=O)OC)(C)C)/CCC)/C2=C([C@@H](C(CC2=O)(C)C)C(=O)OC)[O-]
1217	CCCCCCCCCCCCCCCCCCCCCC(=O)Nc1ccn(c(=O)n1)[C@H]2[C@@H]([C@@H]([C@@H](O2)CO)O)O
1218	CCC/C(=C(/CC)\C(=O)CCC(=O)N(Cc1ccccc1)Cc2ccccc2)/NNC(=O)c3cc4ccccc4cc3O
1219	CCc1ccc([nH+]c1)CCOc2ccc(cc2)c3cc(ccc3OCCc4ccc(cn4)CC)C[C@@H]5C(=O)NC(=O)S5
1220	CC(=O)O[C@@]1(CC[C@@H]2[C@@]1(CC[C@H]3[C@H]2CCC4=CC(=O)CC[C@]34C)C)C(=O)COC(=O)CCC(=O)N[C@H](CC(=O)[O-<br>])[C@@H]5CCOC(C5)(C)C
1221	Cc1c(ccc2c1oc(=O)cc2c3ccccc3)OC[C@H](CN(Cc4ccco4)C[C@@H](COc5ccc6c(cc(=O)oc6c5C)c7ccccc7)O)O
1222	C[C@H]1CCC(=[NH+]C1)[C@@H](C)[C@H]2[C@@H](C[C@H]3[C@@]2(CC[C@@H]4[C@H]3CC=C5[C@@]4(CC[C@@H](C5)O[C@@H]6[C@@H]([C@H]<br>([C@@H]([C@H](O6)COC(=O)C)OC(=O)C)OC(=O)C)OC(=O)C)C)C)OC(=O)C
1223	Cc1cc(nc(n1)[N-]S(=O)(=O)c2ccc(cc2)NC(=O)[C@@](C(F)(F)F)(OC([C@@](C(F)(F)F)(OC(C(C(F)(F)F)(F)F)(F)F)F)(F)F)F)C
1224	c1ccc(cc1)C(=O)N[C@@H]2[C@H]([C@@H]([C@H](O[C@@H]2OC(=O)c3ccccc3)COC(=O)c4ccccc4)OC(=O)c5ccccc5)OC(=O)c6ccccc6
1225	Cc1cc(cc(c1O)C(C)(C)C)CCC(=O)OCCOCCOCCOC(=O)CCc2cc(c(c(c2)C(C)(C)C)O)C
1226	CCCC[NH+](CCCC)CN1c2ccc(cc2/C(=N\NC(=O)COc3cc(ccc3C(C)C)C)/C1=O)Br
1227	c1ccc(cc1)/C(=[NH+]\CCC[NH+](CCC/[NH+]=C(/c2ccccc2)\c3ccccc3O)CCC/N=C(/c4ccccc4)\c5ccccc5O)/c6ccccc6O
1228	CCOc1ccc(cc1C)c2c(cn(n2)c3ccccc3)/C=C\4/C(=O)N(C(=S)S4)CCCCCCCCCCC(=O)O
1229	CCN(CC)c1ccc(cc1)/C=C(\C(=O)N/N=C/c2ccc(c(c2)OCC)OC(=O)c3ccccc3Cl)/NC(=O)c4ccccc4Cl
1230	[H]/N=C\1/C(=C/c2cn(c3c2cccc3)CCOc4ccccc4[C@H](C)CC)/C(=O)N=C5N1N=C(S5)CCCCCCC
1231	Cc1cc(c(c(c1S(=O)(=O)/N=C(/N)\NCCC[C@H](C(=O)On2c(=O)c3ccccc3nn2)NC(=O)OCC4c5ccccc5-c6c4cccc6)C)C)OC
1232	CCOc1cc(cc(c1OCC)OCC)C(=O)Nc2nnc(s2)SCC(=O)Nc3ccccc3C(F)(F)F
1233	CCOc1cc(cc(c1OCC)OCC)C(=O)Nc2nnc(s2)SCC(=O)Nc3cc(ccc3Cl)C(F)(F)F
1234	c1ccc2c(c1)/C(=C/3\C(=O)N(C(=S)S3)CCCCCCCCCCC(=O)[O-])/C(=O)N2CC(=O)Nc4ccc(cc4)F
1235	CC[NH+](CC)c1ccc(c(c1)OCc2ccc(cc2Cl)Cl)/C=N/Nc3nc(nc(n3)Nc4ccc(cc4)OC)NCc5ccco5
1236	CCC/C(=N\OC(C(F)(F)F)(C(F)(F)F)NC(=O)Nc1cccc(c1)[C@@](C(=O)OCC)(C(F)(F)F)O)/Cl
1237	CCCCCCCCCCOc1ccc(cc1)C(=O)Nc2ccc3c(c2)sc(n3)SCC(=O)NC[C@@H]4CCCO4
1238	CCOc1ccc(cc1OCC)CCNS(=O)(=O)c2cc(ccc2C)c3c4ccccc4c(n[nH+]3)Nc5ccc(cc5)OC
1239	CCCCCCCCCC(=O)N(CCc1ccc(c(c1)OC)OC)CCC(=O)Nc2nnc(s2)c3ccc(cc3)Cl
1240	CCOC(=O)[C@](c1ccc(cc1)NC(=O)N[C@@](C(=O)OCC)(C(F)(F)F)OCC(F)(F)F)(C(F)(F)F)O
1241	c1cc(oc1)c2cc(nc(n2)SCCC(=O)Nc3cc(cc(c3)OCC(F)(F)F)OCC(F)(F)F)C(F)(F)F
1242	c1cc2c(ccnc2cc1Cl)NCCC[NH+]3CCN(CC3)CCCCCCC[NH+]4CCN(CC4)CCCNc5ccnc6c5ccc(c6)Cl
1243	CCC(C)(C)c1ccc(c(c1)C(C)(C)CC)OCCCC(=O)Nc2ccc(c(c2)N/C(=C(\C(=O)c3ccc(cc3)OC)/n4c(c[nH]c4=O)C(=O)OC(C)C)/[O-])OC
1244	

1245	CCCCOc1ccc(cc1)C(=O)Oc2ccc(cc2)/C=N/NC(=O)c3ccccc3[N-]S(=O)(=O)c4ccc(cc4)Cl
1246	CCCCCCCCCCCC(=O)NCC(=O)N/N=C/c1ccc(c(c1)OC)OC(=O)c2ccc(cc2Cl)Cl
1247	CCOc1ccc(cc1)C(=O)Oc2ccc(cc2OCC)/C=N/NC(=O)c3ccc(cc3)[N-]S(=O)(=O)c4ccc(cc4)Cl
1248	Cc1cc(ccc1OCC(C)C)c2c(cn(n2)c3ccccc3)/C=C\4/C(=O)N(C(=S)S4)CCCCCCCCCCC(=O)O
1249	CC(C)(C)CC(C)(C)c1ccc(cc1)OCCOc2ccc(cc2N)C(=O)NCCNC(=O)c3ccc4ccccc4c3O
1250	CCCCCCCCCCCCCCCCOc1ccc(c(c1)S(=O)(=O)[N-]Nc2[n+](c3ccccc3s2)C)C(=O)[O-]
1251	CC1([C@@H]2CC[C@]1([C@]([C@@H]2OCc3ccc(cc3)OC)(C=C[C@]4(COC(O4)(C)C)[C@@H](C=C)OCc5ccc(cc5)OC)O)C=C)C
1252	CC1([C@@H]2CC[C@]1([C@]([C@@H]2OCc3ccc(cc3)OC)(C=C[C@]4(COC(O4)(C)C)[C@@H](C=C)OCc5ccc(cc5)OC)O)/C=C\OCc6ccc(cc6)OC)C
1253	CCCCOc1ccc(cc1)C(=O)Oc2ccc(cc2OC)/C=N/NC(=O)COc3cc(c(cc3C(C)C)Br)C
1254	COc1ccc(cc1)NC(=O)CN2c3ccccc3/C(=C/4\C(=O)N(C(=S)S4)CCCCCCCCCCC(=O)[O-])/C2=O
1255	CCCCCCCC[C@H](c1ccc2c(c1)OCCOCCOc3ccc(cc3OCCOCCO2)[C@H](CCCCCCCC)O)O
1256	Cc1cn(c(=O)[nH]c1=O)[C@H]2C[C@@H]([C@H](O2)COC(=O)OC[C@@H](C)c3ccccc3[N+](=O)[O-])O[P@@](N(C(C)C)C(C)C)OCCC#N
1257	Cc1cn(c(=O)[nH]c1=O)[C@H]2C[C@@H]([C@H](O2)COC(=O)OC[C@@H](C)c3ccccc3[N+](=O)[O-])O[P@](N(C(C)C)C(C)C)OCCC#N
1258	CCCOc1ccc(cc1OCC)/C=N/NC(=O)c2ccc(cc2)N(Cc3ccccc3Cl)S(=O)(=O)C
1259	CC[C@H](C)c1ccc(cc1)OCCOc2ccc(cc2OCC)/C=N/NC(=O)CSc3nnc(s3)C
1260	CCN(CC)c1ccc(cc1)n2nc3cc(c(cc3n2)NC(=O)c4cc(c(c(c4)OCC)OCC)OCC)C
1261	CCOC(=O)CC[C@@H](C(=O)OCC)NC(=O)c1ccc(cc1)NCc2nc3c(n2C)c(=O)n(c(=O)n3C)C
1262	CC[NH+](CC)CCN1[C@H]([C@@H](C(=O)C1=O)C(=O)c2ccc3c(c2)C[C@H](O3)C)c4ccc(c(c4)OCC)OCC=C
1263	CC[C@@H](c1nnnn1Cc2ccco2)N(Cc3ccccc3OC)Cc4cc5cc(ccc5[nH]c4=O)OCC
1264	Cc1cc(c(c(c1OC(=O)C)C)C)OC[C@H](CNCCNC(=O)c2ccc(c(c2)S(=O)(=O)N)Cl)O
1265	CCN(CC)S(=O)(=O)c1ccc(cc1)C(=O)C2=C(C(=O)N([C@H]2c3ccc(cc3)C(C)C)CCCOC)O
1266	CCCOc1ccc(cc1)[C@@H]2C(=C(C(=O)N2CCC[NH+](CC)CC)[O-])C(=O)c3cnn(c3C)c4ccccc4
1267	CCN(CC)CCOC(=O)c1ccc(cc1)NC(=O)c2cc3c(nn(c3s2)C)c4ccc(c(c4)OC)OC
1268	CCCOc1ccc(cc1)C(=O)C2=C(C(=O)N([C@H]2c3cccc(c3)OCCC(C)C)CCCn4cc[nH+]c4)[O-]
1269	CCCOc1ccc(cc1OCC)[C@H]2C(=C(C(=O)N2CCC[NH+](C)C)[O-])C(=O)c3c(n4cccc(c4n3)C)C
1270	CCOc1cc(ccc1OCC=C)[C@@H]2C(=C(C(=O)N2CCC[NH+](C)C)[O-])C(=O)c3c(n4cccc(c4n3)C)C
1271	CCCCCCOc1ccc(cc1OC)[C@H]2C(=C(C(=O)N2CCC[NH+](C)C)[O-])C(=O)c3c(nc(s3)C)C
1272	CC[NH+](CC)CCN1[C@@H](C(=C(C1=O)[O-])C(=O)c2c(nc(s2)C)C)c3ccc(c(c3)OC)OCCC(C)C
1273	CCOc1ccc(cc1)CN(C[C@H]2CCCO2)[C@@H](c3cc4cc(ccc4[nH]c3=O)C)c5nnnn5CCOC
1274	CC[C@H](c1nnnn1C[C@H]2CCCO2)N(Cc3ccccc3OC)Cc4cc5cc(ccc5[nH]c4=O)OCC
1275	CCCN(CCC)S(=O)(=O)c1ccc(cc1)C(=O)N(CC[NH+](C)C)c2nc3c(cccc3s2)OCC
1276	CC[NH+](CC)CCCN1[C@@H](C(=C(C1=O)[O-])C(=O)c2cnn(c2C)c3ccccc3)c4ccc(cc4)OCC=C
1277	CCOc1ccc(cc1)CC(=O)N/N=C/C(=N/NC(=O)Cc2ccc(cc2)OCC)/[C@@H]([C@@H]([C@@H](CO)O)O)[O-]
1278	C[NH+](C)CCCN(c1nc2cc(ccc2s1)OC)C(=O)c3ccc(cc3)S(=O)(=O)N(CC=C)CC=C
1279	CCN(CC)CCNc1nc2c(n1C)c(=O)n(c(=O)n2C)CCOC(=O)c3cc(c(c(c3)OC)OC)OC
1280	CCOc1ccc(cc1)CC(=O)N/N=C/C(=N\NC(=O)Cc2ccc(cc2)OCC)/[C@H]([C@@H]([C@@H](CO)O)O)[O-]
1281	CCOc1ccc(cc1)CC(=O)N/N=C/C(=N\NC(=O)Cc2ccc(cc2)OCC)/[C@H]([C@H]([C@@H](CO)O)O)[O-]
	CCCCOc1ccc(cc1OC)[C@@H]2c3c(n[nH]c3C(=O)N2CCCN4CCOCC4)c5ccc(cc5)OC

1282	CCOc1cc(ccc1OC(=O)CCCOc2ccc(cc2)C(C)(C)C)/C=C(\C#N)/C(=O)NCc3ccco3
1283	CCOc1ccc2c(c1)cc(c(=O)[nH]2)CN(Cc3ccccc3OC)[C@H](c4nnnn4Cc5ccco5)C(C)C
1284	Cc1ccc2cc(c(=O)[nH]c2c1)CN(CCc3ccc(c(c3)OC)OC)Cc4nnnn4CCc5ccccc5
1285	CC[C@H](c1nnnn1Cc2ccco2)N(CCc3ccc(c(c3)OC)OC)Cc4cc5cccc(c5[nH]c4=O)C
1286	CCOc1ccc(cc1OCC)CCNC(=S)N(Cc2cc(c(c(c2)OC)OC)OC)C[C@@H]3CCCO3
1287	CCOC(=O)c1ccc(cc1)NC(=O)CSc2nnc(n2CC=C)CCCOc3ccc(cc3C)Cl
1288	CCCN1c2ccc(cc2/C(=N\O[C@@H]3[C@H]([C@@H]([C@@H]([C@@H](O3)COC(=O)C)OC(=O)C)OC(=O)C)NC(=O)C)/C1=O)C
1289	CC(=O)N[C@@H]1[C@@H]([C@@H]([C@@H](O[C@@H]1OC(=O)COc2ccc(cc2Cl)Cl)COC(=O)C)OC(=O)C)OC(=O)C
1290	CCCCN(CCCC)S(=O)(=O)c1ccc(cc1)C(=O)Nc2nnc(o2)Cc3ccc(cc3)SCC
1291	CCOc1cc(cc(c1[O-])[N+](=O)[O-])[C@@H]2C3=C(C[C@H]([C@@H](C3=O)C(=O)OC)C)NC(=C2C(=O)OCCSCC)C
1292	CC1(CC(=O)[C@H](C(=O)[C@@H]1C(=O)OC)/C=N\CCCCCCN/C=C/2\C(=O)CC([C@@H](C2=O)C(=O)OC)(C)C)C
1293	[H]/N=C(\NCCC[C@@H](C(=O)[O-])NC(=O)OC(C)(C)C)/NS(=O)(=O)c1c(c(c2c(c1C)CCC(O2)(C)C)C)C
1294	[H]/N=C(/NCCC[C@@H](C(=O)[O-])NC(=O)OCC1c2ccccc2-c3c1cccc3)\NS(=O)(=O)c4c(cc(c(c4C)C)OC)C
1295	CCN(CC)S(=O)(=O)c1ccc(cc1)NC(=O)COC(=O)Cc2ccc(cc2)OCc3c(noc3C)C
1296	CC[NH+](CC)CCCN1[C@H](C(=C(C1=O)[O-])C(=O)c2ccc3c(c2)C[C@H](O3)C)c4ccc(c(c4)OCC)OCC=C
1297	[H]/N=C\1/C(=C\c2cc(c(c(c2)OC)OCCOc3ccccc3C)OC)/C(=O)N=C4N1N=C(S4)CCCCC
1298	CCCCN(C)S(=O)(=O)c1ccc(cc1)C(=O)Nc2nnc(o2)c3cc(c(c(c3)OCC)OCC)OCC
1299	CC(=O)N[C@@H]1[C@@H]([C@@H]([C@@H](O[C@@H]1Oc2c3ccccc3ccc2C(=O)OC)COC(=O)C)OC(=O)C)OC(=O)C
1300	CCC(=O)Nc1nnc(s1)SCc2cc(=O)c(co2)OC(=O)c3cc(c(c(c3)OCC)OCC)OCC
1301	CCC(CC)C(=O)Nc1nnc(s1)SCc2cc(=O)c(co2)OC(=O)c3ccc(c(c3)OCC)OCC
1302	CCOCCCN(CC(=O)N(Cc1ccccc1)Cc2c(ccs2)C)C(=O)Nc3cc(cc(c3)OC)OC
1303	COCCN(CC(=O)N(CCc1ccc(c(c1)OC)OC)Cc2cccs2)C(=O)Nc3cccc(c3)OC
1304	CC(C)CCOc1ccc(cc1OC)[C@H]2C(=C(C(=O)N2CCC[NH+](C)C)[O-])C(=O)c3ccc4c(c3)OCCO4
1305	COc1ccc(cc1)c2nnc(n2c3ccc(cc3)OC)SCC(=O)N/N=C/c4ccccc4OCC(=O)[O-]
1306	CCCOc1ccc(cc1)C(=O)Oc2ccc(cc2)/C=N/NC(=O)CSc3nnc(n3c4ccccc4)C
1307	Cc1ncc(n1CCOC(=O)N[C@@H](C(Cl)(Cl)Cl)[NH+](Cc2ccccc2)Cc3ccccc3)[N+](=O)[O-]
1308	CCOCCCN([C@H](c1ccc(cc1)O)C(=O)NC(C)(C)C)C(=O)Cn2nc(nn2)c3ccc(cc3)Cl
1309	CCOc1ccc(cc1)C[NH+](Cc2ccco2)[C@@H](c3cc4ccc(cc4[nH]c3=O)C)c5nnnn5CCOC
1310	CCOC(=O)c1ccccc1NC(=O)Cn2c3ccccc3c(=O)n(c2=O)CCC(=O)NCc4ccccc4OC
1311	CC/C=C(\c1csc(n1)N)/C(=O)N[C@@H]2[C@H]3N(C2=O)C(=C(CS3)COC(=O)N)C(=O)OCOC(=O)C(C)(C)C
1312	CCc1ccc(cc1)N([C@@H](c2ccc(cc2)OC)C(=O)NC(C)(C)CC)C(=O)CCC(=O)Nc3cc(on3)C
1313	CC[NH+](CC)CCCN1[C@@H](/C(=C(/c2ccc(c(c2)Cl)OCC)\O)/C(=O)C1=O)c3cc(ccc3OC)OC
1314	CC(C)(C)NC(=O)c1ccc(cc1)NC(=O)CCCCCCCC(=O)Nc2ccc(cc2)C(=O)NC(C)(C)C
1315	CCCN1C(=O)c2c([nH+]cn2C[C@]1(C)C(=O)NCCC(C)C)C(=O)NCc3cc(c(c(c3)OC)OC)OC
1316	CC[NH+]1CCN(CC1)c2ccc(cc2Cl)NC(=S)NC(=O)c3cc(c(c(c3)OCC)OCC)OCC
1317	CCCCOc1ccc(cc1OC)[C@H]2C(=C(c3ccc(cc3)OCC)O)C(=O)C(=O)N2CCC[NH+](CC)CC
1318	CC[NH+](CC)CCN1[C@@H](/C(=C(/c2ccc3c(c2)C[C@@H](O3)C)\O)/C(=O)C1=O)c4ccc(c(c4)OCC)OCC=C
	CC(C)(C)NC(=O)c1cccc(c1)NC(=O)CCCCCCCC(=O)Nc2cccc(c2)C(=O)NC(C)(C)C

1319	Cc1cc(c(c(c1OC(=O)C)C)C)OC[C@H](C[NH2+]CCNC(=O)c2ccc(c(c2)S(=O)(=O)N)Cl)O
1320	Cc1ccc(cc1)C(=O)N/C(=C/c2ccc(cc2)OCCOc3ccccc3OC)/C(=O)NCCC[NH+](C)C
1321	CC(C)Cn1c(c(c(=O)[nH]c1=O)N(CC(C)C)C(=O)CN(C)C(=O)COc2ccc(cc2OC)C(=O)C)N
1322	CCn1cc(c2c1cccc2)/C=C(\C(=O)NCCCn3ccnc3)/NC(=O)c4cc(c(c(c4)OC)OC)OC
1323	CC[NH+](CC)CCCN1[C@H](C(=C(C1=O)O)C(=O)c2cnn(c2C)c3ccccc3)c4cccc(c4)OCC=C
1324	CC[NH+]1CCN(CC1)c2c(c([nH+]n2c3ccccc3)C)CN(CCCOC)C(=O)c4cc(cc(c4)OC)OC
1325	CC[NH+](CC)CCN1[C@@H](C(=C(C1=O)O)C(=O)c2c(nc3n2cccc3)C)c4ccc(c(c4)OCC)OCC=C
1326	CC[NH+](CC)CCN(CC(=O)N1CCN(CC1)c2ccc(n[nH+]2)c3ccc(cc3OC)OC)C(=O)COC
1327	Cc1c(c(n(n1)c2ccccc2)Oc3ccccc3OC)C[NH+](CCOC)C[C@H](COCc4ccccc4)O
1328	CC[C@@H](c1nnnn1C[C@H]2CCCO2)[NH+](CCc3cccc(c3)C)Cc4cc5cc(ccc5[nH]c4=O)OCC
1329	CC[C@H](c1nnnn1C[C@H]2CCCO2)[NH+](Cc3ccccc3OC)Cc4cc5cc(ccc5[nH]c4=O)OCC
1330	Cc1cccc(c1C)OCCCCn2c3ccccc3[nH+]c2CCNC(=O)c4cc(c(c(c4)OC)OC)OC
1331	Cc1ccc(c(c1)C)OCCCCn2c3ccccc3[nH+]c2CCNC(=O)c4cc(c(c(c4)OC)OC)OC
1332	COc1ccc(cc1)OCCCCn2c3ccccc3[nH+]c2CCNC(=O)c4cc(c(c(c4)OC)OC)OC
1333	C[C@H](c1[nH+]c2ccccc2n1CCCCOc3ccccc3OC)NC(=O)c4cc(c(c(c4)OC)OC)OC
1334	CCCCOc1ccc(cc1OCC)[C@@H]2c3c([nH]nc3C(=O)N2CCCn4cc[nH+]c4)c5ccc(cc5)OC
1335	CCOC(=O)Cn1c(nnn1)C[NH+](CCc2ccc(c(c2)OC)OC)Cc3cc4cc(ccc4[nH]c3=O)OC
1336	CC(C)(C)n1c(nnn1)C[NH+](CCc2ccc(c(c2)OC)OC)Cc3cc4cc(c(cc4[nH]c3=O)OC)OC
1337	CC[C@H](c1nnnn1Cc2ccco2)[NH+](CCc3ccc(c(c3)OC)OC)Cc4cc5ccc(cc5[nH]c4=O)C
1338	CC[C@H](c1nnnn1Cc2ccco2)[NH+](CCc3ccc(c(c3)OC)OC)Cc4cc5cccc(c5[nH]c4=O)C
1339	CC/C=C(\c1csc(n1)N)/C(=O)N[C@@H]2[C@@H]3N(C2=O)C(=C(CS3)COC(=O)N)C(=O)OCOC(=O)C(C)(C)C
1340	Cc1c(c([nH]c1C(=O)OC)C)C(=O)C2=C(C(=O)N([C@H]2c3cc(c(c(c3)OC)OC)OC)CCC[NH+](C)C)[O-]
1341	CC1(CC(=O)C(=C([C@H]1C(=O)OC)O)/C=N/CCCCCCN/C=C/2\C(=O)CC([C@H](C2=O)C(=O)OC)(C)C)C
1342	CC[C@@](C[NH+]1CCCCC1)(C(=O)NC(=O)NC(=O)[C@](CC)(C[NH+]2CCCCC2)C(=O)OCC)C(=O)OCC
1343	CCCN(CCC)S(=O)(=O)c1ccc2c(c1)c(cn2CC)/C=N\NC(=S)Nc3ccccc3OCC
1344	Cc1ccc(c(c1)OCC(=O)NNC(=O)CCCCCCCC(=O)NNC(=O)COc2cc(ccc2C)C)C
1345	Cc1ccccc1[NH+]2CCN(CC2)C[C@@H](COc3ccc(cc3)OC[C@H](CN4CC[NH+](CC4)c5ccccc5C)O)O
1346	CCOC(=O)[C@](c1ccc(c(c1)C)N[C@@H](c2ccc(cc2)OC)P(=O)(OCC)OCC)(C(F)(F)F)O
1347	CCCCOc1ccc(cc1)C(=O)NCc2nnc(n2c3ccccc3)SCC(=O)Nc4nnc(s4)C
1348	CC(C)OCCCNC(=O)C(=O)N/N=C/c1ccc(c(c1)OC)OCC(=O)Nc2ccc(cc2Cl)Cl
1349	CCOc1ccc(cc1)CNc2nc(n(n2)S(=O)(=O)c3ccc(cc3)Cl)NCc4ccc(cc4)OCC
1350	c1ccc(cc1)N2CCN(CC2)C[C@@H](COc3ccc(cc3)OC[C@H](CN4CCN(CC4)c5ccccc5)O)O
1351	CC[C@H](C(=O)NCc1cccnc1)Sc2nc3cc(ccc3c(=O)n2Cc4ccco4)C(=O)NCCOC
1352	CC[C@H](C(=O)NCc1ccc[nH+]c1)Sc2nc3cc(ccc3c(=O)n2Cc4ccco4)C(=O)NCCOC
1353	CCCCOc1ccc(cc1)C(=O)Oc2ccc(cc2OC)/C=N/NC(=O)CNC(=O)c3ccc(cc3)Cl
1354	CCCCCCCCN1C(=O)/C(=C/c2c(nc3c(cccn3c2=O)C)NCCCn4cc[nH+]c4)/SC1=S
1355	CCN(CC)CCCOc1ccc(cc1)/C=C/C(=O)Nc2ccc(c(c2)Nc3nccc(n3)c4cccnc4)C
	COc1cccc(c1)/C=N/NC(=O)CSc2nnc(n2CC=C)CNc3cccc4c3cccc4

1356	CCOc1cc(ccc1OC(=O)c2ccc(cc2Cl)Cl)/C=N/NC(=S)Nc3ccc(cc3)OC
1357	CCN(CC)C(=O)c1c(c(c(s1)NC(=S)N[C@@H](C)c2ccc(cc2)C(C)(C)C)C(=O)OC)C
1358	CCOc1cc(cc(c1OCC)OCC)C(=O)NC(=S)Nc2ccc(c(c2)Cl)N3CCCC3
1359	CCOc1cc(cc(c1OCC)OCC)C(=O)NCC(=O)N/N=C/c2ccc(cc2Cl)Cl
1360	CCCNc1c2cnn(c2nc(n1)SCC)CCNC(=O)C(C)(C)Oc3ccc(cc3)Cl
1361	CC(C)(C)c1ccc(c(c1)NC(=O)COC(=O)CCNS(=O)(=O)c2ccc(cc2)Cl)OC
1362	CCCCOc1ccc(cc1C(=O)NC(=S)Nc2cccc(c2)OC[C@@H]3CCCO3)Br
1363	CC[C@H](C)Oc1ccc(cc1)NC(=S)NC(=O)c2cc(ccc2OCCOC)Br
1364	CCC[C@@H](C(=O)NC(C)(C)C)N(C[C@@H]1CCCO1)C(=O)CNS(=O)(=O)c2ccc(cc2)Cl
1365	CCCCOc1ccc(cc1Br)C(=O)NNC(=O)COc2ccccc2[C@@H](C)CC
1366	CCCCOc1ccc(cc1Br)C(=O)NNC(=O)CCCOc2ccc(cc2Cl)Cl
1367	CCCCCCOc1ccc(cc1Br)C(=O)NNC(=O)COc2ccccc2C(C)C
1368	CCCCCCOc1ccc(cc1Br)C(=O)NNC(=O)COc2ccc(cc2)Cl
1369	CCCCCCOc1ccc(cc1C(=O)NNC(=O)COc2ccc(cc2)Cl)Br
1370	CCCCCCOc1ccc(cc1C(=O)NC(=S)Nc2ccc(c(c2)[N+](=O)[O-])Cl)Br
1371	CCCCOc1ccc(cc1C(=O)NNC(=O)COc2ccccc2[C@@H](C)CC)Br
1372	CC[C@@H](C)c1ccccc1OCC(=O)NNC(=O)c2ccc(c(c2)Br)OCCOC
1373	CCCCCCOc1ccc(cc1)C(=O)NNC(=O)COc2ccc(cc2C(C)(C)C)Br
1374	CCN(CC)C(=O)c1cccc(c1)NC(=S)NC(=O)c2cc(ccc2OCCC(C)C)Br
1375	CCCN(CCC)S(=O)(=O)c1ccc2c(c1)c(cn2C)/C=N/NC(=S)Nc3ccccc3CC
1376	CC[C@H](C)c1ccc(cc1)NC(=O)CCSc2nnc(n2c3ccccc3)CNc4ccccc4
1377	CC[C@@H](C)NS(=O)(=O)c1ccc(cc1)NC(=O)CSc2nnc(n2CC=C)C(F)(F)F
1378	CCCCN(CC)C(=O)c1cc(ccc1N2CCNCC2)NS(=O)(=O)c3ccc(cc3)CCC
1379	c1cc(ccc1[C@@H]2CCC=C2)OC[C@H](CN3CCN(CC3)C[C@@H](COc4ccc(cc4)[C@@H]5CCC=C5)O)O
1380	c1cc(ccc1[C@@H]2CCC=C2)OC[C@@H](CN3CCN(CC3)C[C@H](COc4ccc(cc4)[C@H]5CCC=C5)O)O
1381	CCC(C)(C)c1ccc(cc1)Oc2ccc(cc2)NC(=O)COC(=O)CNC(=O)c3ccc(c(c3)C)C
1382	CCCCCCC(=O)N[C@@H]([C@@H](C)CC)C(=O)Nc1nnc(s1)c2cccc(c2)Br
1383	CC(C)COC[C@H](C[NH+](Cc1nc(c2c(csc2n1)c3ccccc3OC)[O-])C4CCCCC4)O
1384	CCCCNC(=O)[C@@H](c1cc2ccccc2c3c1cccc3)N(CCC)C(=O)CNC(=O)c4ccccc4
1385	CCCCNC(=O)[C@H](c1cc2ccccc2c3c1cccc3)N(CCC)C(=O)CNC(=O)c4ccccc4
1386	CCCCCOc1ccc(cc1)[C@@H]2c3c(n[nH]c3C(=O)N2CCCOC)c4cc(cc(c4O)C)C
1387	CCCCCOc1cccc(c1)[C@H]2c3c(n[nH]c3C(=O)N2CCCOC(C)C)c4cc(c(cc4O)C)Cl
1388	CCCCCOc1cccc(c1)[C@H]2c3ccc(cc3OC(=C2C#N)N)OC(=O)COc4cccc(c4C)C
1389	CCCCCOc1ccc(cc1OCC)[C@@H]2c3c(n[nH]c3C(=O)N2CC=C)c4c(cc(cc4O)C)C
1390	CCCCCOc1ccc(cc1OCC)[C@H]2c3c(n[nH]c3C(=O)N2CC=C)c4c(cc(cc4O)C)C
1391	CCOc1cc(c(cc1NC(=O)CCCOc2ccc(cc2)Cl)OCC)NC(=O)c3ccccc3
1392	CCCCOc1cccc(c1)[C@H]2C(=C(Nc3n2nc(n3)SCCCC)C)C(=O)Nc4ccccc4
	CCc1cccc(c1)N/C(=N\C(=O)c2ccc(c(c2)OCC)OCC)/NCc3c(nn(c3C)C)C

1393	COc1ccc(c(c1)OC)NC(=S)Nc2cc(cc(c2)OCC(F)(F)F)OCC(F)(F)F
1394	CCCCOc1ccc(cc1)NC(=O)CSc2ccc(cc2)NC(=O)c3c(cccc3OC)OC
1395	COC(=O)c1ccccc1NC(=O)CSc2nnc(n2CC=C)CNc3cccc4c3cccc4
1396	COc1ccc(c(c1)OC)NC(=S)NCCCn2c(c(c(n2)C(F)(F)F)Br)C3CC3
1397	CCC[N+]1=C(S/C(=C/c2ccc(cc2OCC)[NH+](CC)CC)/C1=O)Nc3ccc(cc3)OCC
1398	CCCCOc1ccc(cc1)C(=O)Nc2ccc(cc2)c3nnc(n3C)SCC(=O)NC4CC4
1399	CC(C)CCOc1cccc(c1)[C@@H]2c3c([nH]nc3C(=O)N2CCCOC(C)C)c4cc(ccc4O)Cl
1400	CCOc1ccc(cc1)[C@H](CCC(=O)N/N=C\c2cc(c(c(c2)Br)OCC=C)OC)O
1401	Cc1cc(nc(n1)NS(=O)(=O)c2ccc(cc2)NC(=O)C(C(C(C(C(C(C(C(F)(F)F)(F)F)(F)F)(F)F)(F)F)(F)F)(F)F)(F)F)C
1402	Cc1ccc(cc1)NC2=C(C(=O)N(C2=O)CCCOC)Sc3ccc(cc3)NC(=O)CC(C)C
1403	COCCCN(Cc1[nH]c(=O)c2c(csc2n1)c3ccc(cc3)OC)C[C@H](Cc4ccccc4)O
1404	CCOc1ccc(cc1)C(=O)Oc2ccc(cc2OCC)/C=N/NC(=O)CNC(=O)c3ccc(cc3)C
1405	CCN(CC)S(=O)(=O)c1cccc(c1)c2nnc(n2N)SCC(=O)c3cc(n(c3C)CCOC)C
1406	CCOC(=O)[C@](C(F)(F)F)(NC(=O)Nc1ccc(cc1)S(=O)(=O)Nc2ccc(nn2)OC)OCC
1407	CCc1ccc(cc1)OCC(=O)N[C@@H](C(Cl)(Cl)Cl)NC(=S)Nc2ccc(cc2)C(=O)OC
1408	CCN(CC)S(=O)(=O)c1ccc(cc1)C(=O)NCCn2c3c(cn2)c(nc(n3)SC)NCC(C)C
1409	CCOc1ccc(cc1)C(=O)NCCn2cc(c3c2cccc3)SCC(=O)Nc4ccccc4OCC
1410	CCCCCOc1ccc(cc1)C(=O)OCC(=O)N(CCOC)c2c(n(c(=O)[nH]c2=O)CC(C)C)N
1411	CCCOc1ccc(cc1Br)C(=O)NC(=S)Nc2ccc(cc2)C(=O)NCCCOC
1412	CCCCOc1ccc(cc1C(=O)NC(=S)Nc2ccc(cc2)C(=O)NCCOC)Br
1413	CC[C@@H](C)c1ccc(cc1)OCC(=O)NNC(=S)NC(=O)CCCOc2ccc(cc2Cl)Cl
1414	CC[C@H](C)c1ccc(cc1)OCC(=O)NNC(=S)NC(=O)CCCOc2ccc(cc2Cl)Cl
1415	CCCNS(=O)(=O)c1ccc(cc1)NC(=S)NC(=O)CCCOc2ccc(cc2Cl)Cl
1416	CCCCCCOc1ccc(cc1)C(=O)NNC(=S)NC(=O)c2ccc(c(c2)Br)OC
1417	CCCCCOc1ccc(cc1)C(=O)NC(=S)Nc2ccc(cc2)S(=O)(=O)N(C)c3ccccc3
1418	CCCCCOc1ccc(cc1)C(=O)NC(=S)Nc2ccc(cc2)S(=O)(=O)Nc3c(cccc3C)C
1419	CCCCCCOc1ccccc1C(=O)Nc2ccc(cc2)S(=O)(=O)Nc3cc(nc(n3)OC)OC
1420	CCCCCCOc1ccccc1C(=O)NC(=S)NNC(=O)COc2ccc(cc2C)Br
1421	CCCCCCOc1ccccc1C(=O)NC(=S)NNC(=O)COc2ccc(cc2)C(=O)OCCC
1422	CCCCCOc1ccccc1C(=O)NC(=S)Nc2ccc(cc2)S(=O)(=O)NC3CCCCC3
1423	CCCCCOc1ccccc1C(=O)NC(=S)Nc2ccc(cc2)S(=O)(=O)N(CC)c3ccccc3
1424	CCCCCCOc1ccc(cc1Br)C(=O)NC(=S)NNC(=O)COc2cccc(c2)C
1425	CCCOc1ccc(cc1C(=O)NC(=S)NNC(=O)COc2ccc(cc2)[C@@H](C)CC)Br
1426	CCCC(=O)Nc1ccc(cc1)NC(=O)CCCCCCCC(=O)Nc2ccc(cc2)NC(=O)CCC
1427	CCOc1cc(cc(c1OCC)OCC)C(=O)NC(=S)Nc2ccc(cc2)Nc3ccc(cc3)OC
1428	c1ccc(cc1)OCCOc2cccc(c2)C(=O)NC(=S)Nc3ccc(cc3)C(=O)NCc4ccco4
1429	CCCC[NH+](CCCC)CCNC(=O)c1cc(nc2c1cc(cc2)S(=O)(=O)N3CCCC[C@@H]3C)[O-]
	COc1cc(cc(c1)OC)NC(=O)CCSc2nnc(n2CC=C)CNc3cccc4c3cccc4

1430	CC[C@@H](C)Sc1nnc(s1)NC(=O)CSc2nc([nH]n2)N/N=C/c3ccc(cc3)C(=O)OC
1431	CCN(CC)c1ccc(cc1)/C=N/Nc2[nH]nc(n2)SCC(=O)NC(c3ccccc3)c4ccccc4
1432	COCCCN(Cc1[nH]c(=O)c2cc(c(cc2n1)OC)OC)Cc3[nH]c(=O)c4cc(c(cc4n3)OC)OC
1433	CCCCOc1ccc(cc1)C(=O)N/C(=C/c2ccc(cc2)N(C)C)/C(=O)Nc3ccc(cc3)C(=O)OC
1434	CCCCCCOc1ccccc1C(=O)NC(=S)Nc2ccc(cc2)S(=O)(=O)Nc3ccccc3
1435	CCCCCNc1c(c(=O)n2cc(ccc2n1)C)/C=C/3\C(=O)N(C(=S)S3)CCCCCC(=O)O
1436	CCCCCOc1cccc(c1)[C@@H]2c3ccc(cc3OC(=C2C#N)N)OC(=O)COc4ccccc4OC
1437	CCCCOc1ccc(cc1OCC)[C@H]2c3c(n[nH]c3C(=O)N2CCCOC)c4cc(cc(c4O)C)C
1438	CCCOc1ccc(cc1OCC)[C@@H]2c3c(n[nH]c3C(=O)N2CCCOC(C)C)c4cc(cc(c4O)C)C
1439	CCOc1cc(ccc1OCCC(C)C)[C@@H]2c3c(n[nH]c3C(=O)N2CCCOC)c4c(cc(cc4O)C)C
1440	C=CCSc1ccccc1NC(=O)CSc2nc([nH]n2)N/N=C/c3cccc(c3)Oc4ccccc4
1441	CC(C)OC(=O)c1cc(cc(c1)NC(=S)Nc2c(c3c(s2)CCCC3)C(=O)OC)C(=O)OC(C)C
1442	CC(C)CSc1nnc(s1)NC(=O)CSc2nc([nH]n2)N/N=C/c3cc(ccc3OC)OC
1443	CCOc1cc(cc(c1OCC)OCC)C(=O)NC(=S)NCc2ccc(cc2)c3nc4ccccc4o3
1444	CC[NH+](CC)c1ccc(cc1)/C=N/NC(=O)c2c(non2)C(=O)N/N=C/c3ccc(cc3)N(CC)CC
1445	CCCCCOc1ccc(cc1OC)[C@H]2c3c(n[nH]c3C(=O)N2CCCOC)c4cc(ccc4O)Cl
1446	CCOc1ccc(cc1)[C@H](CCC(=O)N/N=C/c2cc(c(c(c2)Br)OCC#N)OCC)O
1447	Cc1c2c(=O)[nH]c(nc2sc1C(=O)OC(C)C)CN(CC=C)C[C@H](COc3ccc(cc3)Cl)O
1448	Cc1c(c(n(n1)c2ccccc2)O)C(=O)CCCCCCCCC(=O)c3c(nn(c3O)c4ccccc4)C
1449	CCOC[C@H](CN(CCN1CCOCC1)Cc2[nH]c(=O)c3c(csc3n2)c4cccc(c4)OC)O
1450	CC[NH+](CC)c1ccc(cc1)/C=N/NC(=O)c2ccc(o2)C(=O)N/N=C\c3ccc(cc3)N(CC)CC
1451	CCOc1ccc(cc1OCC)C/C(=N\OC(C(F)(F)F)(C(F)(F)F)NC(=O)c2cccc(c2)C)/N
1452	CC[NH+](CC)c1ccc(cc1)/C=N\Nc2c(nc3ccccc3n2)N/N=C\c4ccc(cc4)N(CC)CC
1453	CC(=O)O[C@H]1[C@H](O[C@@H]([C@@H]([C@@H]1OC(=O)C)OC(=O)C)OC)COC(c2ccccc2)(c3ccccc3)c4ccccc4
1454	CCCCOc1ccc(cc1)C(=O)[C@H]2[C@@H](N(C(=O)C2=O)c3nc(c(s3)C(=O)OC)C)c4ccc(c(c4)OC)OC
1455	CCOCCOC(=O)C1[C@H](C2C(=NC1=C)C[C@@H](CC2=O)c3ccc(c(c3)OC)OC)c4cc(c(cc4Br)OC)OC
1456	CCOc1cccc2c1N(C(C3=C2C4(C(=C(S3)C(=O)OC)C(=O)OC)SC(=C(S4)C(=O)OC)C(=O)OC)(C)C)C(=O)COc5ccccc5
1457	CCOc1ccc2c(c1)C3=C(C(N2C(=O)COc4ccc(cc4)F)(C)C)SC(=C(C35SC(=C(S5)C(=O)OC)C(=O)OC)C(=O)OC)C(=O)OC
1458	CC(=O)OC[C@H]1[C@@H]([C@@H]([C@@H]([C@@H](O1)SC2=N/C(=C\c3cccs3)/C(=O)N2c4ccc(cc4)Cl)OC(=O)C)OC(=O)C)OC(=O)C
1459	CC(=O)OC[C@H]1[C@H]([C@@H]([C@@H]([C@@H](O1)SC2=N/C(=C\c3cccs3)/C(=O)N2c4ccc(cc4)OC)OC(=O)C)OC(=O)C)OC(=O)C
1460	C/C(=N\CCCCCCC/N=C(\C)/C1C(=O)OC(=N1)c2ccc(cc2)Cl)/C3C(=O)OC(=N3)c4ccc(cc4)Cl
1461	CCOCCOC(=O)C1[C@@H](C2C(=NC1=C)C[C@H](CC2=O)c3ccc(c(c3)OC)OC)c4cc(c(c(c4)Br)OC)OCC
1462	CCSCCOC(=O)C1[C@@H](C2C(=NC1=C)C[C@H](CC2=O)c3ccc(c(c3)OC)OC)c4cc(c(c(c4)Br)OC)OC
1463	CC1=C([C@H]([C@H]2C(=N1)C[C@H](CC2=O)c3ccc(c(c3)OC)OC)c4cc(c(c(c4)Br)OC)OC)C(=O)OCCOC(C)C
1464	CCOCCOC(=O)C1[C@H](C2=C(C[C@@H](CC2=O)c3ccc(c(c3)OC)OC)N=C1C)c4cc(c(c(c4)Br)OC)OCC
1465	CCOc1ccc(cc1)N(CC(=O)Nc2ccc(cc2)S(=O)(=O)[N-]c3ccc(cc3)Br)S(=O)(=O)c4ccc(cc4)SC
1466	c1ccc(cc1)C(=O)COC(=O)Cn2c(=O)c3cc4c(cc3c2=O)c(=O)n(c4=O)CC(=O)OCC(=O)c5ccccc5
	CCN(CC)CCN(c1nc2ccc(cc2s1)OCC)C(=O)c3ccc(cc3)S(=O)(=O)N(C)Cc4ccccc4

1467	CCN(CC)CCN(c1nc2c(cccc2s1)OCC)C(=O)c3ccc(cc3)S(=O)(=O)N(C)c4ccccc4
1468	CCN(CC)CCN(c1nc2c(ccc(c2s1)OC)OC)C(=O)c3ccc(cc3)S(=O)(=O)N(C)C4CCCCC4
1469	CCO[P@](=O)(c1ccccc1)c2ccccc2OCCOCCOCCOc3ccccc3[P@@](=O)(c4ccccc4)OCC
1470	CCO[P@@](=O)(c1ccccc1)c2ccccc2OCCOCCOCCOc3ccccc3[P@](=O)(c4ccccc4)OCC
1471	CC(=O)OC[C@@H]1[C@H]([C@@H]([C@H]([C@H](O1)Oc2cc3c(cc2Cl)c(cc(=O)o3)c4ccccc4)OC(=O)C)OC(=O)C)OC(=O)C
1472	CC(=O)OC[C@@H]1[C@H]([C@@H]([C@@H]([C@H](O1)O[C@@]2([C@@H]([C@@H]([C@@H](O2)CCl)OC(=O)C)OC(=O)C)CCl)OC(=O)C)OC(=O)C)Cl
1473	CC(=O)OC[C@@H]1[C@H]([C@H]([C@@H]([C@@H](O1)Oc2ccc(c(c2)[O-])C(=O)COc3ccc(cc3)F)OC(=O)C)OC(=O)C)OC(=O)C
1474	CCCC(=O)[C@H]1C(=CC([C@H](C1=O)C(=O)OC)(C)C)N2CCN(CC2)C3=C(C(=O)[C@@H](C(C3)(C)C)C(=O)OC)C(=O)CCC
1475	CC(=O)OC[C@H]1[C@@H]([C@@H]([C@@H]([C@@H](O1)SCCOc2ccc(cc2)Br)OC(=O)C)OC(=O)C)OC(=O)C
1476	CCCC(=O)[C@@H]1C(=CC([C@H](C1=O)C(=O)OC)(C)C)N2CCN(CC2)C3=CC([C@H](C(=O)[C@H]3C(=O)CCC)C(=O)OC)(C)C
1477	c1ccc(cc1)C(=O)O[C@H]2[C@@H]([C@H](O[C@H]([C@@H]2OC(=O)c3ccccc3)OC(=O)c4ccccc4)C(=O)[O-])OC(=O)c5ccccc5
1478	CCCN(CCC)S(=O)(=O)c1ccc(cc1)C(=O)O/N=C(\C)/c2ccc(c(c2)CSc3ccccn3)OC
1479	C(CCl)OP(=O)(OCCCl)OCC(COP(=O)(OCCCl)OCCCl)(CCl)CCl
1480	CC1=C([C@@H](n2c(=O)/c(=C/c3ccc(c(c3)OC)OC(=O)C)/sc2=N1)c4ccc(c(c4)OC)OC(=O)C)C(=O)OCC(C)C
1481	CCOc1cc(cc(c1OCC)OCC)C(=O)N(c2ccccn2)C(=O)c3cc(c(c(c3)OCC)OCC)OCC
1482	Cc1cc(ccc1OCC=C)C(=O)C2=C(C(=O)N([C@H]2c3ccc(cc3)OCC=C)c4nc(c(s4)C(=O)OC)C)[O-]
1483	CCOc1cc(cc(c1OCC)OCC)C(=O)/N=c/2\c(cc3c(n2CCCOC(C)C)nc4ccccn4c3=O)C#N
1484	CCOCCCn\1c2c(cc(/c1=N\C(=O)c3cc(c(c(c3)OCC)OCC)OCC)C(=O)OCC)c(=O)n4ccccc4n2
1485	CC1([C@H](N(C(=O)N1CCCCC(=O)N/N=C/c2ccc(c(c2)OC)OC)c3cccc(c3)Cl)N(C(=O)Nc4cccc(c4)Cl)[O-])C
1486	CCOc1ccc2c(c1)sc(n2)N3[C@@H](C(=C(C3=O)[O-])C(=O)c4ccc5c(c4)OCCO5)c6ccc(c(c6)OCC)OCCC(C)C
1487	CCCOc1ccc(cc1OCC)[C@H]2C(=C(C(=O)N2c3nnc(s3)SCc4cccc5c4cccc5)[O-])C(=O)c6ccc7c(c6)OCCO7
1488	CCCCCn\1c2c(cc(/c1=N\C(=O)c3cc(c(c(c3)OCC)OCC)OCC)C(=O)OCC)c(=O)n4cccc(c4n2)C
1489	CCOc1cc(ccc1OCC=C)[C@@H]2C(=C(C(=O)N2c3nnc(s3)SCc4ccccc4F)[O-])C(=O)c5ccc6c(c5)C[C@H](O6)C
1490	CCOc1cc(ccc1OCC=C)[C@@H]2C(=C(C(=O)N2c3nnc(s3)SCc4ccc(cc4)C)[O-])C(=O)c5ccc6c(c5)C[C@@H](O6)C
1491	CCOc1ccc2c(c1)C3=C(C(N2C(=O)c4ccc(cc4)C(C)(C)C)(C)C)SC(=C(C35SC(=C(S5)C(=O)OC)C(=O)OC)C(=O)OC)C(=O)OC
1492	c1ccc(cc1)C/N=C(/[C@@H](C2=NC(N=C(O2)c3ccco3)(C(F)(F)F)C(F)(F)F)C(C(F)(F)F)(C(F)(F)F)NC(=O)c4ccco4)\[O-]
1493	CCn1cc(c(=O)c2c1nc(nc2)N3CCN(CC3)C(=S)Nc4c(c(c(s4)C(=O)OC(C)C)C)C(=O)OC(C)C)C(=O)[O-]
1494	COc1ccc(c(c1)OC)C(=O)N(Cc2ccc(c(c2)OS(=O)(=O)c3cccc(c3)C(F)(F)F)OC)Cc4ccco4
1495	CCOCCCN(CC(=O)N(Cc1cccn1Cc2ccccc2)C3CCCCC3)S(=O)(=O)c4cccc5c4nccc5
1496	CCOCCCN(CC(=O)N(CCc1ccc(c(c1)OC)OC)Cc2cccs2)S(=O)(=O)c3ccc(cc3)Br
1497	CC(=O)OC[C@@H]1[C@@H]([C@@H]([C@@H]([C@@H](O1)Oc2ccc3c(cc(=O)oc3c2)c4ccccc4)OC(=O)C)OC(=O)C)OC(=O)C
1498	CCCCOc1ccc(cc1)C(=O)C2=C(C(=O)N([C@@H]2c3cc(c(c(c3)OC)OC)OC)c4nnc(s4)SCc5ccccc5Cl)[O-]
1499	CCOc1cc(ccc1OC(=O)c2ccc(cc2)OC)/C=N/NC(=O)c3ccc(cc3)[N-]S(=O)(=O)c4ccc(cc4)C
1500	CCOc1ccc(cc1)C(=O)Oc2ccc(cc2OC)/C=N/NC(=O)c3ccc(cc3)[N-]S(=O)(=O)c4ccc(cc4)Cl<br>CCN(CC)c1ccc(cc1)/C=c/2\c(=O)n3c(=NC(=C([C@H]3c4ccc(c(c4)OC)OC(=O)C)C(=O)OCC(C)C)C)s2<br>CC1(CN=C(S1)N2CC[NH+](CC2)C[C@@H](COc3ccc(cc3)OC[C@H](C[NH+]4CCN(CC4)C5=NCC(S5)(C)C)O)O)C
